# Asymmetric Synthesis of Bicyclic Pyrazolidinones through Alkaloid‐Catalyzed [3+2]‐Cycloadditions of Ketenes and Azomethine Imines

**DOI:** 10.1002/chem.202104391

**Published:** 2022-03-14

**Authors:** Mukulesh Mondal, Shubhanjan Mitra, Dylan J. Twardy, Manashi Panda, Kraig A. Wheeler, Nessan J. Kerrigan

**Affiliations:** ^1^ Department of Chemistry Oakland University 2200 N. Squirrel Road Rochester MI 48309-4477 USA; ^2^ School of Chemical Sciences Dublin City University Glasnevin Dublin 9 Ireland; ^3^ Department of Chemistry Whitworth University Spokane WA 99251 USA

**Keywords:** azomethine imine, diastereoselectivity, enantioselectivity, ketene, pyrazolidinone

## Abstract

A versatile asymmetric synthesis of bicyclic pyrazolidinones through alkaloid‐catalyzed formal [3+2]‐ and [3+2+2]‐cycloadditions of ketenes with azomethine imines is described. The methodology was found to be tolerant of ketene and a variety of monosubstituted ketenes (R=alkyl, OAc). The products were formed in good to excellent yields (71–99 % for 24 examples, 39 examples in all), with good to excellent diastereoselectivity in many cases (dr 3 : 1 to 27 : 1 for 22 examples), and with excellent enantioselectivity for most examples (≥93 % *ee* for 34 products). In the case of most disubstituted ketenes, the reaction proceeded through a [3+2+2]‐cycloaddition to form structurally interesting bicyclic pyrazolo‐oxadiazepinediones with moderate diastereoselectivity (dr up to 3.7 : 1) and as racemic mixtures (3 examples). The method represents the first unambiguous example of an enantioselective reaction between ketenes and a 1,3‐dipole.

## Graphical abstract

**The catalytic asymmetric synthesis** of bicyclic pyrazolidinones through alkaloid‐catalyzed formal [3+2]‐ and [3+2+2]‐cycloadditions of in situ‐generated and pre‐generated ketenes with azomethine imines is described. The method provides good to excellent yields (71–99 % for 24 examples, 39 examples in all), good to excellent diastereoselectivity (dr 3 : 1 to 27 : 1) and excellent enantioselectivity for most examples (≥93 % *ee* for 34 products).

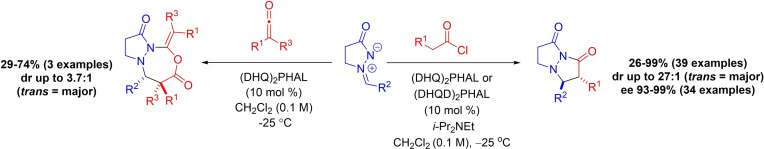

## Introduction

**Scheme 1:**
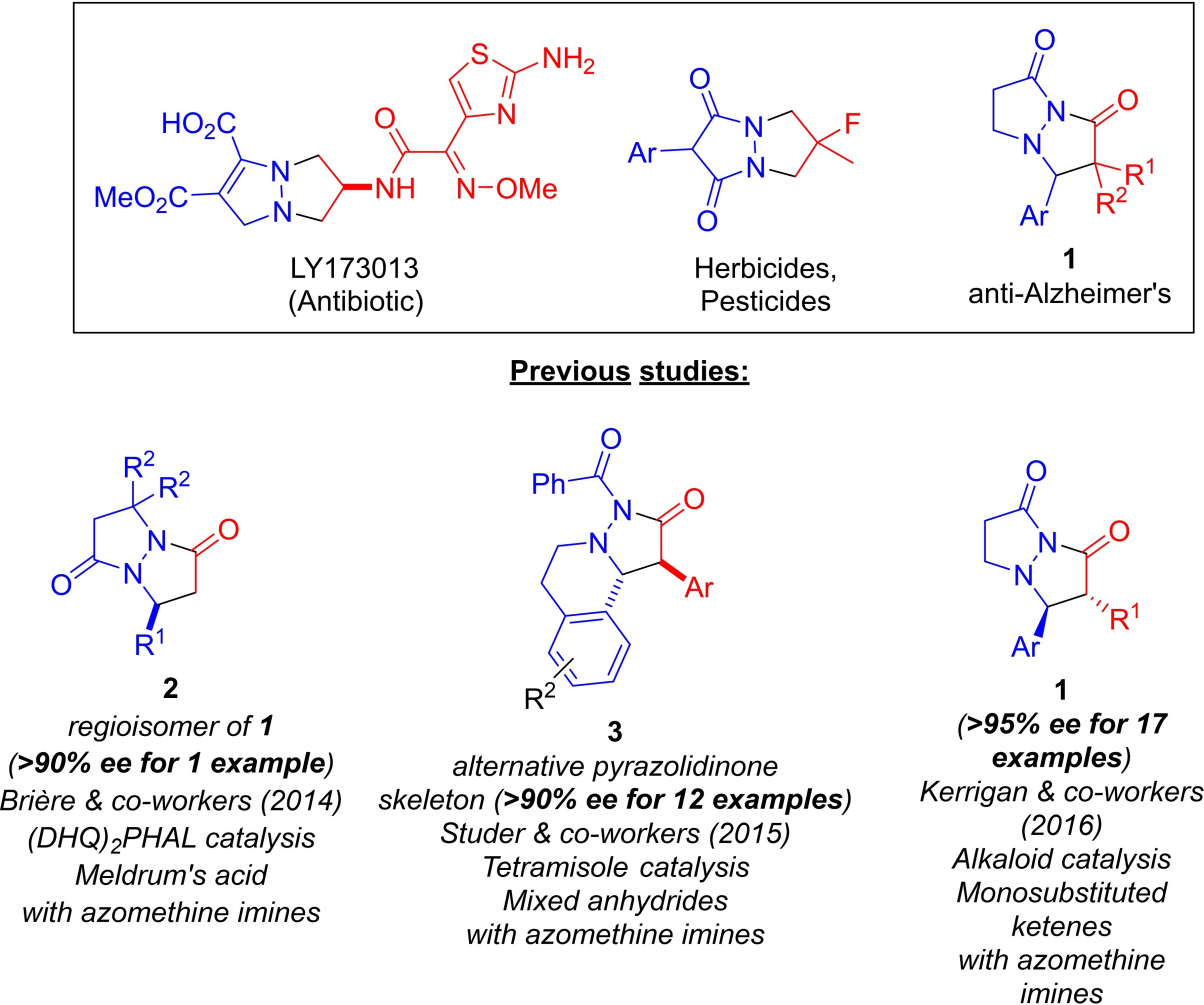
Biologically active pyrazolidinones/pyrazolidinone derivatives and previous studies.

The importance of the bicyclic pyrazolidinone structural motif stems from its presence in many pharmacologically active molecules.^[1]^ These complex molecules exhibit anti‐Alzheimer's activity and related bicyclic pyrazolidinone derivatives have displayed antibiotic, herbicidal, and pesticidal activity (Scheme 1).^[1]^ With this range of biological activity, the development of novel enantioselective methods for the synthesis of bicyclic pyrazolidinones has recently attracted much attention. Despite this activity, catalytic asymmetric entry to bicyclic pyrazolidinones **1** has proven a difficult goal.^[2]^

A number of approaches to the bicyclic pyrazolidinone structure based on a chiral catalyst‐controlled 1,3‐dipolar cycloaddition of an azomethine imine with various dipolarophile reactant partners have been explored.^[3]^ Fu's seminal contribution predicated on a Cu(I)/chiral phosphaferrocene oxazoline‐catalyzed [3+2]‐cycloaddition of azomethine imines with alkynes, to afford the closely related bicyclic pyrazolines, inspired a number of catalytic asymmetric strategic approaches to the pyrazolidinone skeleton.[^4^, ^5^, ^6^, ^7^, ^8^]

In 2014, Brière and co‐workers reported that an unexpected regioisomer of a bicyclic pyrazolidinone (**2**) could be accessed through the (DHQ)_2_PHAL‐catalyzed Knoevenagel‐aza‐Michael cyclocondensation reaction of Meldrum's acid with azomethine imines.^[6]^ However, only one example was formed in ≥90 % *ee*. Additionally, a significant drawback of this method is that it's limited to Meldrum's acid as reactant partner for the azomethine imine, effectively eliminating the possibility of substitution α to the carbonyls in the product. In 2015, Studer and co‐workers demonstrated that a chiral tetramisole‐catalyzed 1,3‐dipolar cycloaddition of azomethine imines with mixed anhydrides could provide access to pyrazolidinones **3**, containing a tetrahydroisoquinoline moiety, with high enantioselectvity (>90 % *ee*) for 12 examples.^[7]^ However, the tetramisole‐catalyzed method is restricted to tetrahydroisoquinoline‐containing imines, and as such is unable to provide access to the bicyclic pyrazolidinones represented by **1** or related fused bicyclic 5‐membered ring systems. In addition, it is also limited to *α*‐aryl substituents (provided by the anhydride) at the stereogenic center adjacent to the imide carbonyl. The tetramisole‐catalyzed reaction was proposed to involve an ammonium enolate intermediate, most likely accessed through deprotonation of an acyl ammonium precursor.^[7]^

Also in 2015, Ye and co‐workers reported the N‐heterocyclic carbene (NHC)‐catalyzed reaction of *α*‐chloroaldehydes with azomethine imines to provide bicyclic pyrazolidinones **3**, with excellent enantiomeric excess obtained in most cases.^[8a]^ A related NHC‐catalyzed oxidative catalytic [3+2] of aldehydes with azomethine imines reported by Ren and co‐workers was also found to be successful with moderate to excellent enantioselectivity (66–98 % *ee*) displayed.^[8b]^

Interestingly, in all of these prior reports of Lewis base‐catalyzed enantioselective 1,3‐dipolar cycloadditions, ketenes had not been demonstrated to be involved as a reactant partner. For a number of years our group has been focused on the development of new enantioselective and diastereoselective reactions involving ketenes, pursuing phosphine, alkaloid, Lewis acid, and transition metal catalysis as a means of creating new methodologies to access important small molecules.[^9^, ^10^] We were particularly attracted to the challenge of developing a new formal [3+2]‐cycloaddition because of the paucity of enantioselective [3+2]‐cycloadditions involving ketenes in the literature.[^10^, ^11^, ^12^]

We, therefore, turned our attention to the development of a new methodology providing access to bicyclic pyrazolidinones from in situ generated ketenes. In 2016 we reported the first unambiguous catalytic enantioselective [3+2]‐cycloaddition of ketenes with azomethine imines.^[13]^ In this current article we describe our complete studies on reaction optimization, the scope of the reaction with respect to ketene (unsubstituted, monosubstituted and disubstituted) and azomethine imine structure (aryl and alkyl substitution), and mechanistic/stereoselectivity analysis.

## Results and Discussion

### Reaction optimization

**Figure 1:**
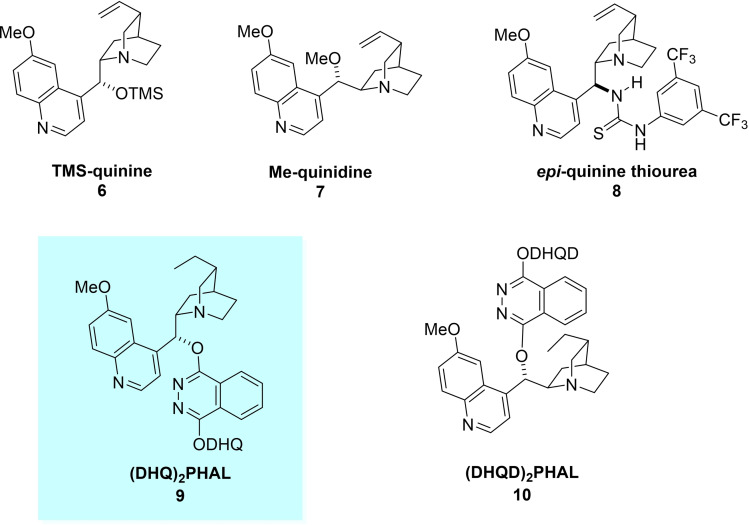
Catalysts investigated for the [3+2]‐cycloaddition.

We began our investigation by examining the reaction of in situ‐generated methylketene with azomethine imine **4 a** (where R^1^=Ph) in the presence of a variety of cinchona alkaloid derivatives (**6**–**10**) in CH_2_Cl_2_ at −25 °C (Table 1, Figure 1). Phosphine catalysts (PMe_3_ and PBu_3_) were also examined in preliminary studies but were found to be ineffective (ca. 5 % conversion with ethylphenylketene). In the alkaloid‐catalyzed reactions, it was found necessary to add propionyl chloride slowly over 10 h to the reaction solution (containing catalyst, azomethine imine, and Hünig's base) in order to maximize the yield of bicyclic pyrazolidinone **1 a**, and limit the formation of methylketene homodimer and aldehyde from azomethine imine decomposition.^[14]^

**Table 1:** Optimization of alkaloid‐catalyzed [3+2] cycloaddition of methylketene with azomethine Imine **4 a**.

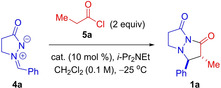						
Entry	Cat.	*T* [°C]	Additive	Yield [%]^[a]^	dr^[b]^	*ee* [%]^[c]^
1	**6**	−25	none	80	1 : 1	98
2	**7**	−25	none	63	1.5 : 1	98
3	**6**	−78	none	(70)	1 : 1	
4	**6**	−25	LiClO_4_	0		
5	**7**	−25	Cu(OTf)_2_	(80)	1 : 1	
6	**7**	−25	Er(OTf)_3_	(75)	1 : 1	
7	**7**	−25	Yb(OTf)_3_	(70)	1 : 1	
8	**7**	−25	none	(40)	1.5 : 1	
9	**8**	−25	none	(25)	2 : 1	
10	**9**	−25	none	90	3 : 1	99
11	**9**	−78	none	61	3 : 1	99
12	**9**	−25	CuI	89	2 : 1	98
13^[d]^	**9**	−25	none	85	3 : 1	99
14^[e]^	**9**	−25	none	45	3 : 1	94

[a] Isolated yield for both diastereomers. Conversion as determined by GC‐MS in parentheses. [b] dr determined by ^1^H NMR or HPLC analysis of crudes. (*R*,*S*)‐isomer=major in most cases. [c] *ee* determined by chiral HPLC or chiral GC analysis for major diastereomer. [d] 2.5 mol% of catalyst used. [e] 1 mol% of catalyst used.

In all cases examined, excellent enantioselectivity in the formation of bicyclic pyrazolidinone **1 a** was observed. However, diastereoselectivity was poor‐to‐modest at first (dr up to 1.5/2 : 1). A number of Lewis acid additives were tested as a means of enhancing diastereoselectivity, but none provided the desired improvement (entries 4–7, 12). It was only after a switch to the dimeric (DHQ)_2_PHAL as catalyst, was promising diastereoselectivity (dr 3 : 1), favoring the *trans*‐diastereomer, achieved (Table 1, entries 10–14).

Catalyst loading (10, 2.5 and 1 mol%) was investigated with a loading of 10 mol% found to be optimal. Only a slight reduction in yield (from 90 % down to 85 %) was obtained at the 2.5 mol% catalyst loading (entry 13), with the same dr and *ee* obtained as for 10 mol% (entry 10). However, with 1 mol% loading there was a significant deterioration in yield (from 90 % down to 45 %), although dr and *ee* remained high (dr 3 : 1, *ee* 94 %, entry 14). It was decided that the substrate scope of the system should be explored using the 10 mol% catalyst loading, rather than the 2.5 mol% loading, due to the cleaner reactions achieved with the higher loading (background formation of aldehyde from the azomethine imine occurred at low catalyst loadings and in the absence of catalyst).

### Reaction scope

Initially, the scope of the methodology was investigated with in situ‐generated monosubstituted ketenes (34 examples, Table 2). Methyl‐, ethyl‐, *n*‐propyl‐, *n*‐hexyl, and acetoxy‐ketene were all found to perform excellently in reaction with aryl‐substituted azomethine imines **4**, with the desired bicyclic pyrazolidinone **1 a**–**1 q** being formed with invariably excellent enantioselectivity (34 examples with *ee* 93–99 % for the major diastereomer). However, the use of in situ‐generated phenylketene, chloroketene, thiophenylketene and isopropylketene resulted in formation of no bicyclic pyrazolidinone.

**Table 2:** Scope of alkaloid‐catalyzed [3+2] cycloaddition of monosubstituted ketenes with azomethine Imines.

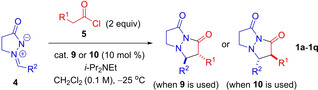
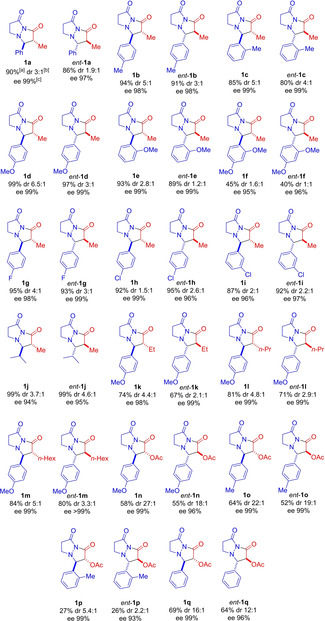

[a] Isolated yield for both diastereomers. [b] dr determined by ^1^H NMR or HPLC analysis of crudes. [c] *ee* determined by chiral HPLC or chiral GC analysis for major diastereomer. (*R*,*S*)‐isomer is the major isomer from most **9**‐catalyzed reactions. (*S*,*R*)‐isomer is the major isomer from most **10**‐catalyzed reactions.

Substitution on the aryl portion of the azomethine imine was flexible, with electron‐donating (MeO− and Me−) and electron‐withdrawing (F−, Cl−) substituents in a variety of positions on the aryl ring being tolerated. *Ortho*‐substitution, however, occasionally led to lower yields (e. g. **1 f**/*ent*‐**1 f**: 40–45 % and **1 p**/*ent*‐**1 p**: 26–27 %). Importantly, an alkyl‐substituted azomethine imine was found to perform excellently with **1 j** and *ent*‐**1 j** being formed in 94–95 % *ee*. However, the reaction was sensitive to the steric bulk of the alkyl substituent, with R^2^=*c*‐hexyl leading to incomplete conversion and poor diastereoselectivity (dr 1 : 1, not shown in Table 2).

Diastereoselectivity in pyrazolidinone formation was generally moderate to excellent, with a dr of up to 27 : 1 being obtained. The *trans*‐diastereomer was formed as the major diastereomer, as determined by X‐ray crystallographic analysis of the major isomer of *ent*‐**1 d**.^[13]^ Generally, (DHQ)_2_PHAL provided higher levels of diastereoselectivity than the pseudoenantiomeric (DHQD)_2_PHAL, for example **1 b** (dr 5 : 1) vs. *ent*‐**1 b** (dr 3 : 1), or **1 n** (dr 27 : 1) vs. *ent*‐**1 n** (dr 18 : 1). Highest diastereoselectivity was observed for ketenes bearing the −OAc substituent, with dr ranging from 12 : 1 to 27 : 1 for 6 examples (**1 n**–**1 q**). Examples **1 p** and *ent*‐**1 p** were notable exceptions giving substantially lower diastereoselectivity, perhaps due to steric interaction with the *ortho*‐methyl aryl group of the azomethine imine. Diastereomerically pure product could be obtained through a single recrystallization as was demonstrated for *ent*‐**1 d** (dr 3 : 1), which was recrystallized from CH_2_Cl_2_/pentane to provide *ent*‐**1 d** in good yield (67 % overall from imine **4 d**) and with excellent diastereomeric purity (dr 37 : 1).^[13]^ The reaction was also found to proceed effectively on a 5 mmol scale from azomethine imine **4 d**, with the desired pyrazolidinone **1 d** being formed in excellent yield (95 %) and with good diastereoselectivity (dr 4 : 1) favoring the *trans*‐isomer (see Supporting Information Experimental for details).

X‐ray crystallographic analysis of *ent*‐**1 d** revealed the absolute configuration, with *ent*‐**1 d** determined to be the (2*S*,3*R*)‐enantiomer.^[13]^ As formation of *ent*‐**1 d** had been through the (DHQD)_2_PHAL **10**‐catalyzed reaction, most pyrazolidinone products of the **10**‐catalyzed reactions were assigned the (2*S*,3*R*)‐configuration. On the other hand, pyrazolidinone products of the **9**‐catalyzed reactions were assigned the (2*R*,3*S*)‐configuration.

We proceeded to explore more difficult cycloadditions such as the reaction of in situ‐generated ketene with azomethine imines (Table 3). The difficulty associated with these reactions lies in the high reactivity of ketene, with competing dimerization as a side reaction, and the absence of a ketene substituent being problematic for attaining high levels of enantiocontrol. Ultimately, catalysts **6**–**9** provided the desired bicyclic pyrazolidinone **1 r**–**1 s** in moderate to excellent yields but with poor to moderate enantioselectivity (10–43 % *ee*) at room temperature. This demonstrated that the presence of a substituent on the ketene was an essential element for attaining good enantiocontrol in the reaction.

**Table 3:** Scope of Alkaloid‐Catalyzed [3+2] cycloaddition of Ketene with Azomethine Imines.

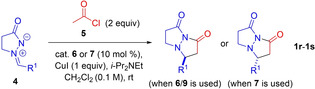					
Entry	Cat.	R^1^	Yield [%]^[a]^	*ee* [%]^[b]^	Product
1	**6**	Ph	23	43	**1 r**
2	**7**	Ph	64	32	*ent*‐**1 r**
3	**9**	Ph	74	10	**1 r**
4^[c]^	**9**	Ph	97	17	**1 r**
5	**6**	4‐FC_6_H_4_	53	25	**1 s**
6	**7**	4‐FC_6_H_4_	59	24	*ent*‐**1 s**

[a] Isolated yield. [b] *ee* determined by chiral HPLC. [c] CuI (1 equiv.) used as additive.

Disubstituted ketenes (methylphenyl‐, diphenyl‐ and dimethyl‐) were then examined as substrates for the alkaloid‐catalyzed reaction (Table 4). Surprisingly, the product that was favored was the result of a [3+2+2]‐cycloaddition for most disubstituted ketenes rather than the earlier seen [3+2]‐cycloaddition. The bicyclic pyrazolo‐oxadiazepinedione products **11 a**–**11 c** formed incorporated two molecules of disubstituted ketene, and were formed with good diastereoselectivity favoring the *trans* (*anti*)‐isomer (dr up to 3.7 : 1, entries 1 and 2, Table 4), albeit with no optical activity. The lack of enantioselectivity using alkaloid catalysts for reactions of disubstituted ketenes is not surprising given poor results observed by other groups.^[15]^ The relative stereochemistry of **11 b** was determined to be *trans* (*anti*) by X‐ray crystal structure analysis. When reactions were run in the absence of the alkaloid catalyst, product **11** was obtained, but as part of a significantly less clean product mixture. Interestingly, when dimethylketene was used as substrate, a product analogous to **11 a**–**11 c**, incorporating two molecules of dimethylketene, was initially formed, but underwent loss of one molecule of dimethylketene to give the usual bicyclic pyrazolidinone product **1 t**. We investigated the synthesis of [3.3.0] fused pyrazolidinones (**1 u**/**1 v)** from **11 a**/**11 b** under a number of reaction conditions (e. g. heating at 40 °C for 5 h or directly from **4 d**, see Supporting Information Experimental) but the desired products were never observed.

**Table 4:** Scope of alkaloid‐catalyzed [3+2+2] cycloaddition of disubstituted ketenes with azomethine imines.

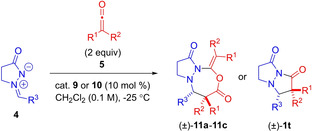							
Entry^[a]^	Cat.	R^1^	R^2^	R^3^	Yield [%]^[a]^	dr^[b]^	Product
1	**9**	Ph	Me	4‐MeOC_6_H_4_	74	3.7 : 1	**11 a**
2	**9**	Ph	Me	4‐MeC_6_H_4_	71	3.1 : 1	**11 b**
3	**9**	Ph	Ph	4‐MeOC_6_H_4_	29	–	**11 c**
4	**9**	Me	Me	4‐MeOC_6_H_4_	13	–	**1 t**
5^[c]^	**9**	Me	Me	4‐MeOC_6_H_4_	52	–	**1 t**
6^[c]^	**10**	Me	Me	4‐MeOC_6_H_4_	55	–	**1 t**
7^[c,d]^	**10**	Me	Me	4‐MeOC_6_H_4_	74	‐	**1 t**

[a] Isolated yield. [b] dr determined by ^1^H NMR or HPLC analysis of crudes. 0 % *ee* for **11 a**–**11 c** and **1 t**. [c] Reaction carried out at −78 °C. [d] CuI (1 equiv.) added.

### Reaction mechanism[^2a^, ^2b^, ^3^, ^9b^, ^11^]

**Scheme 2:**
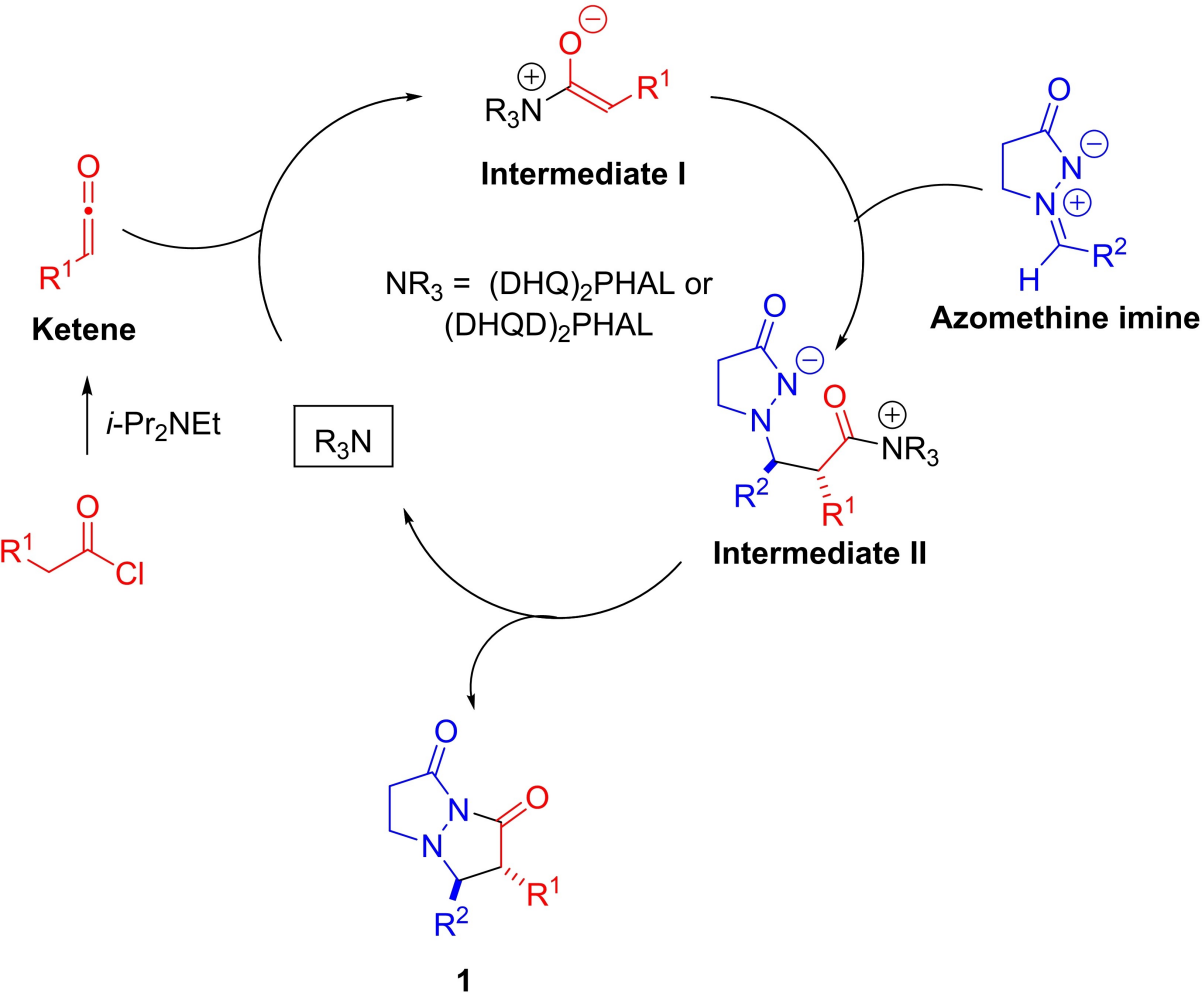
Proposed reaction mechanism for alkaloid‐catalyzed [3+2]‐cycloaddition.

We propose that bicyclic pyrazolidinones **1** are formed through a stepwise process as shown in Scheme 2. The ketene is formed in situ through dehydrohalogenation of acyl chloride by *i*‐Pr_2_NEt. Alkaloid catalyst ((DHQD)_2_PHAL or (DHQ)_2_PHAL) adds to the less sterically hindered side of the monosubstituted ketene (or ketene) to form an ammonium enolate (Intermediate I) stereoselectively, favoring the *Z*‐enolate. Intermediate I then adds to the azomethine imine to form zwitterionic Intermediate II with high enantioselectivity and high diastereoselectivity, favoring the *trans*‐isomer. 5‐Exo‐trig cyclization then leads to the formation of pyrazolidinone **1** with concomitant regeneration of the alkaloid catalyst.

**Scheme 3:**
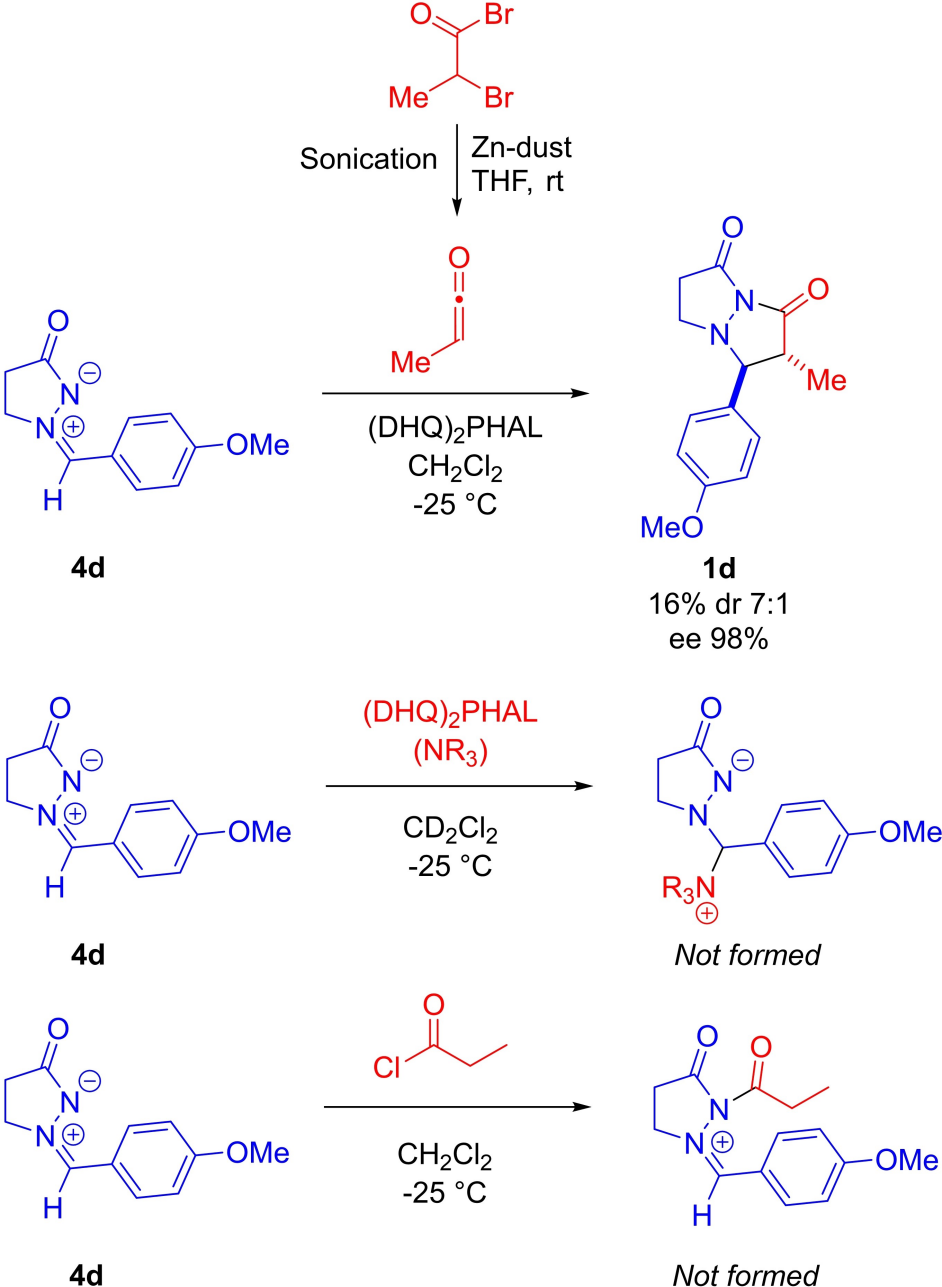
Control and mechanistic experiments.

The involvement of a ketene rather than acyl chloride in reaction with the alkaloid catalyst was supported by the following observations/experimental outcomes (Scheme 3). Independent generation of methylketene through Zn(0)‐mediated dehalogenation of 2‐bromopropionyl bromide and its use in the **9**‐catalyzed reaction with **4 d** resulted in the formation of **1 d**, with almost the same dr (7 : 1 vs. 6.5 : 1 for in situ process) and *ee* (98 % *ee* vs. 99 % for in situ process) as for when propionyl choride and *i*‐Pr_2_NEt were used (for assumed in situ generation of methylketene, Table 2 conditions). Moreover, the same enantiomer and diastereomer was formed as the major isomer under both sets of reactions conditions, i. e. the same sense of selectivity is observed under both pre‐generated ketene and in situ generated ketene reaction conditions. Therefore, we can deduce that ketene is a participant (rather than acyl chloride) in the cycloaddition reactions with azomethine imines.

The possibility of initial addition of the catalyst to the azomethine imine was also explored. An experiment where (DHQ)_2_PHAL and azomethine imine were dissolved in CD_2_Cl_2_ was set up. However, analysis of the reaction mixture by ^1^H NMR spectroscopy revealed no evidence for interaction of (DHQ)_2_PHAL with azomethine imine, and so most likely initial addition of the catalyst to azomethine imine could be ruled out.

The possibility of background reaction between propionyl chloride/ketene with the azomethine imine was also investigated. It was found that when standard reaction conditions, except for the absence of the alkaloid catalyst **9**, were employed for the synthesis of **1 d** that a complex mixture of products resulted. In addition, when azomethine imine was subjected to reaction with propionyl chloride under standard reaction conditions (−25 °C, in CH_2_Cl_2_, but with no catalyst and no Hünig's base), no adduct was formed. This suggested that formation of azomethine imide followed by enolization is unlikely as a reaction pathway for formation of the pyrazolidinone product.

#### [3+2+2] variant with disubstituted ketenes

**Scheme 4:**
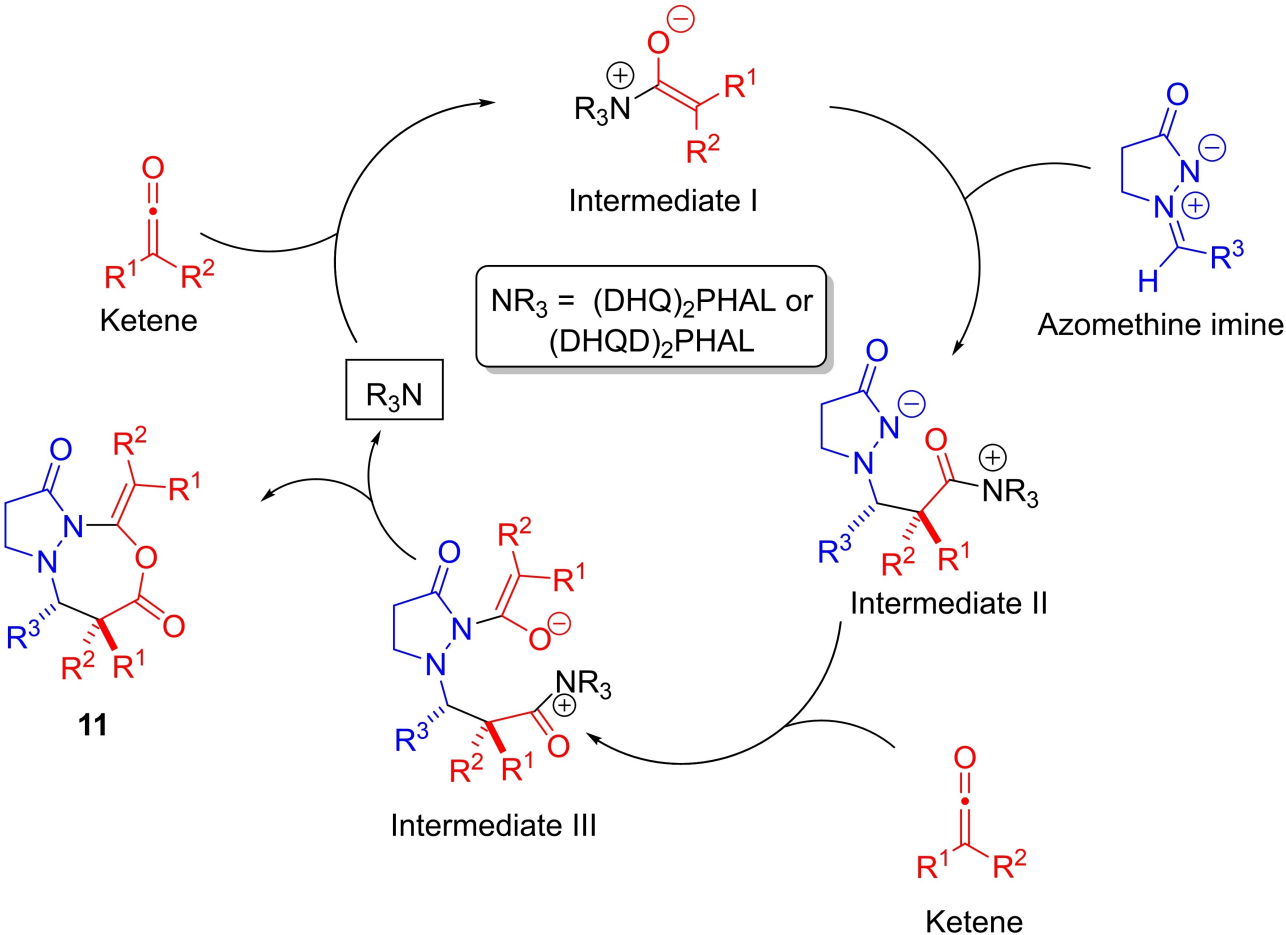
Proposed reaction mechanism for alkaloid‐catalyzed [3+2+2]‐cycloaddition.

Our proposed mechanism for the alkaloid‐catalyzed formation of bicyclic pyrazolo‐oxadiazepinedione products **11 a**–**11 c** involves addition of the alkaloid nucleophilic catalyst to the ketene as before, albeit with a lower equilibrium amount of ammonium enolate formed due to lower electrophilicity of the disubstituted ketene (Scheme 4). The ammonium enolate (intermediate I) would then add to the azomethine imine as before to give an intermediate II with moderate diastereoselectivity (dr up to 3.7 : 1), albeit without any enantioselectivity. However, before cyclization of intermediate II occurs, addition of the azomethine imine anionic nitrogen to another molecule of ketene leads to the formation of an enolate (intermediate III) which adds to the pendant acyl ammonium, leading to the formation of the seven‐membered ring (through a formal [3+2+2]‐cycloaddition).

### Reaction diastereoselectivity rationale

**Scheme 5:**
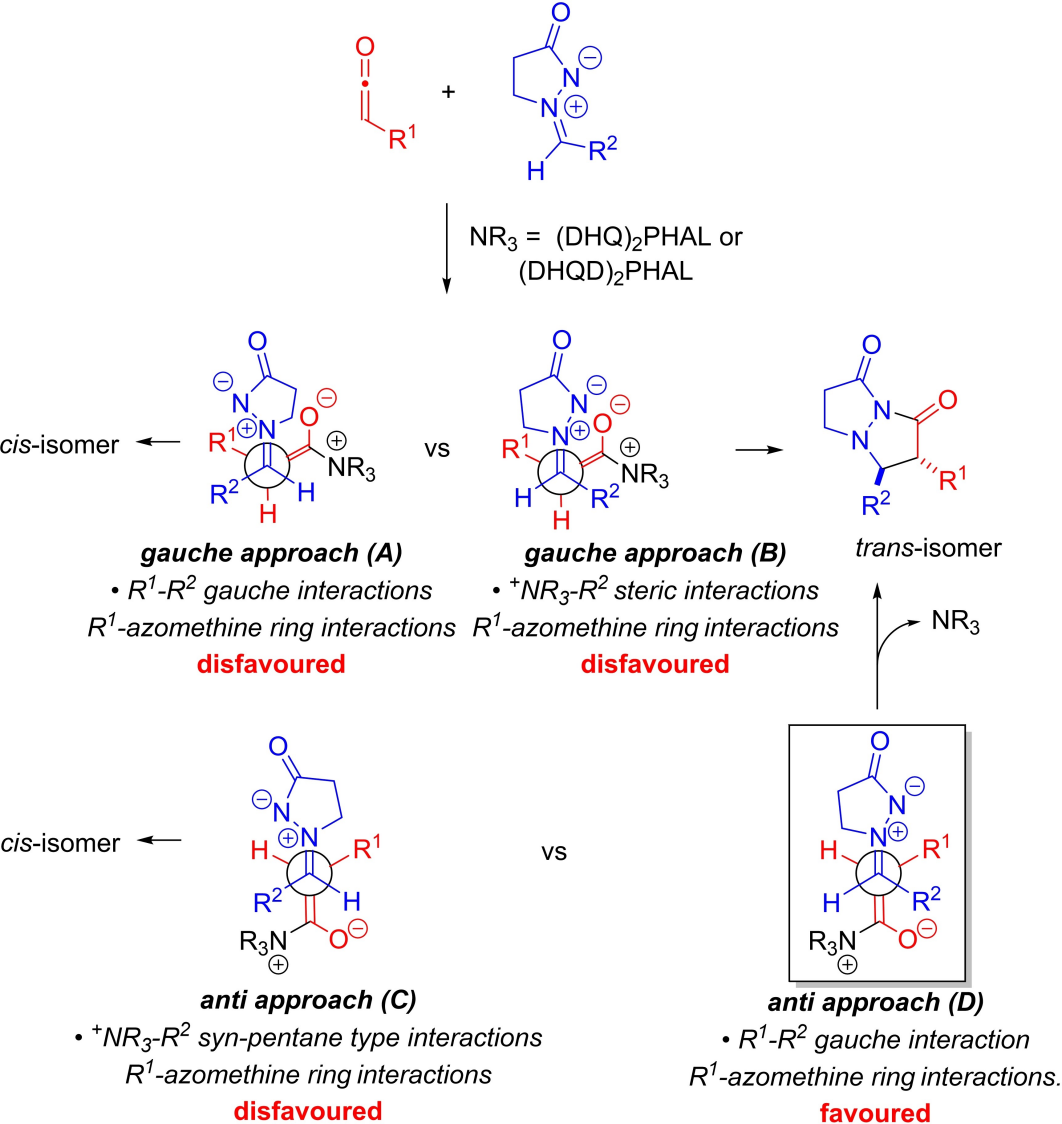
Rationale for diastereoselectivity in formal [3+2]‐cycloaddition (NR_3_=(DHQD)_2_PHAL or (DHQ)_2_PHAL).

Diastereoselectivity in the [3+2]‐cycloaddition depends on the steric interactions between substituents (R^1^, R^2^) on the azomethine imine and the ketene‐derived ammonium enolate (Scheme 5). Gauche approach B of the ammonium enolate to the azomethine imine predicts formation of the *trans‐*diastereomer. However, although it relieves steric interactions between R^1^ and R^2^, it would suffer from severe steric interactions between ^+^NR_3_ and R^2^. Antiperiplanar approach D of the ammonium enolate to the azomethine imine would also result in the formation of the *trans*‐diastereomer. Analysis of the anti‐approach transition state D reveals gauche steric interactions between R^1^ and R^2^, but no significant interactions between ^+^NR_3_ and other substituents (in contrast to B and C). Therefore, it is predicted that antiperiplanar approach D is favored.

Best levels of diastereoselectivity (very good to excellent, dr 12 : 1 to 27 : 1 for 6 examples) were generally observed with the ‐OAc substituent on the ketene (Table 2, **1 n**–**1 q**). In the latter cases (in contrast to R^1^=alkyl), we surmise that a favorable interaction between lone pairs on the ‐OAc group and the positively charged nitrogen of the azomethine imine further stabilizes the putative *anti* approach D leading to a further preference for formation of the *trans*‐isomer (Scheme 5).^[16]^

To investigate whether the preference for the *trans*‐diastereomer was also influenced by equilibration under the reaction conditions we considered the results of earlier experiments (Scheme 3) and carried out a number of other control experiments. One possibility considered was that Hünig's base‐Hünig's base.HCl salt mediated equilibration could be responsible for the high *trans*‐diastereoselectivity observed in some cases. However comparison of the diastereoselectivity (dr 6.5 : 1) in the synthesis of **1 d**, where Hünig's base mediated the in situ ketene generation, with the diastereoselectivity (dr 7 : 1) of a reaction carried out in the absence of Hünig's base, i. e. through Zn(0)‐mediated dehalogenation of 2‐bromopropionyl bromide, showed virtually identical dr values (Scheme 3). This suggested that Hünig's base‐Hünig's base.HCl salt does not play an important role in determining diastereoselectivity. The effect of the Zn(II) salt byproduct, ZnBr_2_, on dr was also explored. Exposure of *ent*‐**1 e** (dr 1.5 : 1) to ZnBr_2_ (2 equiv.) in CH_2_Cl_2_ at −25 °C overnight resulted in only a slight change of dr (to dr 1.1 : 1).

We investigated the possibility that the (DHQ)_2_PHAL catalyst could be responsible for equilibration by exposing *ent*‐**1 e** (dr 1.5 : 1) to the catalyst **9** at −25 °C overnight. However, no significant change (dr 1.2 : 1) in the dr of *ent*‐**1 e** was noted. Exposure to silica gel was also briefly investigated as a means of improving the dr of the pyrazolidinones. However, once again no significant change/improvement in dr (dr 1.6 : 1) was noted after stirring of *ent*‐**1 e** with silica in CH_2_Cl_2_ for 1.5 h at 50–55 °C. Finally, treatment of *ent*‐**1 e** with KO^t^Bu (dr improved to 3.4 : 1) or DBU (dr improved to 2.4 : 1) led to only moderate improvements in dr. To summarize, there is no evidence that isomerization under the reaction conditions is responsible for the relatively high dr (≥6 : 1) observed in many reactions. Indeed, reagents and reaction conditions (e. g. DBU or KO^t^Bu/50 °C) that would usually effect isomerization provided the pyrazolidinone with only moderate diastereoselectivity (dr up to 3.4 : 1).

### Reaction enantioselectivity rationale

**Scheme 6:**
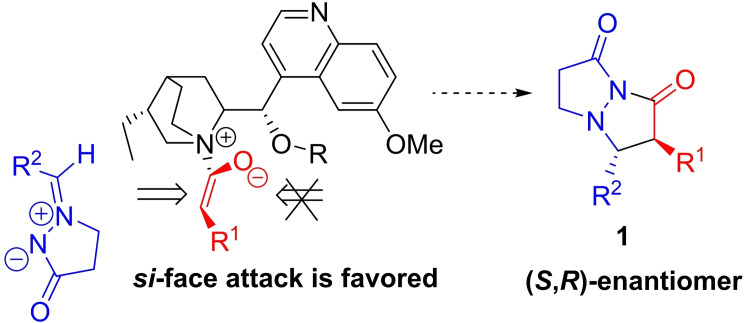
Rationale for enantioselectivity in formal [3+2]‐cycloaddition (catalyst=(DHQD)_2_PHAL).

Enantioselection is determined in the reaction step involving addition of ketene‐derived ammonium enolate (Intermediate I) to azomethine imine to form zwitterionic Intermediate II (Schemes 2 and 6). Approach of the azomethine imine to the *si*‐face of the ammonium enolate, derived from methylketene and (DHQD)_2_PHAL (**10**), is less sterically hindered than approach to the *re*‐face, where the catalyst −OR substituent (containing the phthalazine group) blocks approach. Using the model shown in Scheme 6, the (2*S*,3*R*)‐enantiomer of *ent*‐**1 a** is predicted to be the major enantiomer from the **10**‐catalyzed reaction, in agreement with the absolute configuration established by X‐ray crystallographic analysis for *ent*‐**1 d**.^[13]^ The absolute stereochemical outcome is consistent with models previously advanced by the groups of Calter, Lectka and the authors for related alkaloid‐catalyzed reactions.[^9b^, ^11a^, ^17^]

## Conclusion

To conclude, we have developed an alkaloid‐catalyzed asymmetric synthesis of bicyclic pyrazolidinones from ketenes and azomethine imines. This represents the first unambiguous enantioselective [3+2]‐cycloaddition of ketenes with a 1,3‐dipole. Bicyclic pyrazolidinones were formed from in situ‐generated ketenes and azomethine imines with excellent enantioselectivity in nearly all cases (34 examples ≥93 % *ee*). Interestingly, there was a switch to a [3+2+2]‐cycloaddition in reactions involving most disubstituted ketenes. Future work will involve applications of the reported methodology and the development of other new nucleophile‐catalyzed reactions of ketenes.

## Experimental Section

### General

THF was freshly distilled from benzophenone ketyl radical under nitrogen prior to use, while Hünig's base (diisopropylethylamine) was distilled from calcium hydride.^[18]^ Most anhydrous solvents (dichloromethane and diethyl ether) were obtained by passing through activated alumina columns on a solvent purification system. Hydrazine monohydrate, methyl acrylate, benzaldehyde, *p*‐anisaldehyde, *o*‐tolualdehyde, *p*‐tolualdehyde, 4‐fluorobenzaldehyde, lithium perchlorate, copper(II) triflate, erbium(III) triflate, ytterbium(III) triflate, copper(I) iodide, quinine, quinidine and dinc dust (<10 μm), were purchased from Aldrich Chemical Co. (DHQ)_2_PHAL and (DHQD)_2_PHAL were purchased from AK Scientific, Inc. Propionyl chloride, butyryl chloride, valeroyl chloride, acetoxyacetyl chloride and octanoyl chloride were purchased from Aldrich Chemical Co. and distilled prior to use.^[18]^ TLC plates (Sorbent Technologies, UV254, 250 μM) were used as received. TMS‐quinine, Me‐quinidine and *epi*‐quinine thiourea were synthesized according to literature procedure.^[19]^ Azomethine imines **4 a**–**4 k** (Table 2 graphic, **4 a**: R^2^=Ph; **4 b**: R^2^=4‐MeC_6_H_4_; **4 c**: R^2^=2‐MeC_6_H_4_; **4 d**: R^2^=4‐MeOC_6_H_4_; **4 e**: R^2^=2‐MeOC_6_H_4_; **4 f**: R^2^=2,4‐diMeOC_6_H_4_; **4 g**: R^2^=4‐FC_6_H_4_; **4 h**: R^2^=4‐ClC_6_H_4_; **4 i**: R^2^=3‐ClC_6_H_4_; **4 j**: R^2^=*i*‐Pr; **4 k**: R^2^=*c*‐Hex) were prepared according to literature procedures.[^4^, ^20^] Disubstituted ketenes were prepared according to literature procedures.[^9b^, ^15^, ^21^]

### General procedure for alkaloid‐catalyzed [3+2] cycloaddition of ketene and azomethine imine

To a stirring solution of azomethine imine **4 a**–**4 k** (0.30 mmol, 1 equiv.) and catalyst (0.03 mmol, 0.1 equiv.) in dichloromethane (2.0 mL) at −25 °C, Hünig's base (0.10 mL, 0.60 mmol, 2 equiv.) was added. To this stirring reaction mixture, a solution of acid chloride (0.60 mmol, 2 equiv.) in dichloromethane (1.0 mL) was added over a period of 10 h via syringe pump. The reaction was stirred at this temperature for another 6 h and then poured into cold water (15 mL), extracted with dichloromethane (20 mL×3). The combined organic layers were washed with water (50 mL), and brine (50 mL), and dried over sodium sulfate. The solvent was removed under reduced pressure. The residue was then dissolved in dichloromethane and passed through a plug of regular silica gel (10 g, 2×2 cm) using 10 % EtOAc/dichloromethane as elutant to afford crude product (free from catalyst) for diastereomeric ratio and enantiomeric excess measurement. Pure product was isolated after further regular silica silica gel column chromatographic purification using EtOAc/dichloromethane as eluent (mentioned details below).

### (2S,3R)‐2‐Methyl‐3‐phenyltetrahydro‐1H,7H‐pyrazolo[1,2‐a] pyrazole‐1,7‐dione (ent‐1 a)

Following general procedure, propionyl chloride (0.053 mL, 0.58 mmol) in dichloromethane (1.0 mL) was added over 10 h to a solution of **4 a** (50 mg, 0.29 mmol), Hünig's base (0.10 mL, 0.58 mmol) and (DHQD)_2_PHAL (22 mg, 0.03 mmol) in dichloromethane (1.9 mL) at −25 °C. Elution with 3 % EtOAc/dichloromethane through silica gel column afforded *ent*‐**1 a** as a light yellowish solid (57 mg, 86 %), dr=1.9 : 1 (by ^1^H NMR); HPLC analysis: 97 % *ee* [Daicel Chiralcel AD‐H column; 1.2 mL/min; solvent system: 2 % isopropanol in hexane; retention times: 45.1 min (minor), 54.6 min (major)]; Mp: 136–140 °C; IR (thin film) 2984, 2921, 2885, 1770, 1701, 1455, 1319, 1294, 1275, 699 cm^−1^; ^1^H NMR (400 MHz, CDCl_3_, TMS, Major isomer): δ 7.47‐7.33 (m, 5H), 3.57‐3.48 (m, 2H), 3.10–2.90 (m, 2H), 2.87–2.74 (m, 2H), 1.19 (d, *J*=7.1 Hz, 3H); ^13^C NMR (100 MHz, CDCl_3_, Major isomer): δ 166.7, 165.0, 135.7, 129.2, 129.0, 127.7, 79.0, 52.4, 50.9, 36.6, 11.0; (M^+^H)^+^ HRMS m/z calcd for (C_13_H_15_N_2_O_2_)^+^: 231.1134; found: 231.1131.

### (1R,2R)‐3,5‐Dioxo‐1‐(p‐tolyl)tetrahydro‐1H,5H‐pyrazolo[1,2‐a] pyrazol‐2‐yl acetate (1 p)

Following general procedure, acetoxyacetyl chloride (0.070 mL, 0.64 mmol) in dichloromethane (1.0 mL) was added over 10 h to a solution of **4 c** (60 mg, 0.32 mmol), Hünig's base (0.11 mL, 0.64 mmol) and (DHQ)_2_PHAL (25 mg, 0.03 mmol) in dichloromethane (2.2 mL) at −25 °C. Elution with 3 % EtOAc/dichloromethane through silica gel column afforded **1 p** as a yellowish oil (25 mg, 27 %), dr=5.4 : 1 (by ^1^H NMR and HPLC); HPLC analysis: 99 % *ee* [Daicel Chiralcel OD‐H column; 1.0 mL/min; solvent system: 18 % isopropanol in hexane; retention times: 31.8 min (minor), 34.0 min (major)]; IR (thin film) 2850, 1793, 1748, 1706, 1436, 1420, 1210, 1099, 763 cm^−1^; ^1^H NMR (400 MHz, CDCl_3_, TMS, Major isomer): δ 7.71‐7.65 (m, 1H), 7.32–7.24 (m, 2H), 7.22–7.16 (m, 1H), 5.74 (d, *J*=11.2 Hz, 1H), 4.41 (d, *J*=11.2 Hz, 1H), 3.58 (t, *J*=8.5 Hz, 1H), 3.05–2.93 (m, 1H), 2.89‐2.73 (m, 2H), 2.39 (s, 3H), 2.11 (s, 3H); ^13^C NMR (100 MHz, CDCl_3_, Major isomer): δ 168.7, 165.2, 160.5, 137.0, 131.7, 131.2, 129.2, 127.3, 127.2, 78.4, 71.8, 52.8, 35.5, 20.6, 19.7; (M^+^H)^+^ HRMS m/z calcd for (C_15_H_17_N_2_O_4_)^+^: 289.1188; found: 289.1185.

### 5‐(4‐Methoxyphenyl)‐4‐methyl‐4‐phenyl‐1‐(1‐phenylethylidene)tetrahydro‐1H,3H,9H‐pyrazolo[1,2‐c][1,3,4]oxadiazepine‐3,9‐dione (11 a)

To a stirring solution of **4 d** (60 mg, 0.29 mmol) and (DHQ)_2_PHAL (23 mg, 0.03 mmol) in dichloromethane (2.0 mL) at −25 °C, a solution of methylphenylketene (78 mg, 0.59 mmol) in dichloromethane (1.0 mL) was added over a period of 10 h via syringe pump. The reaction was stirred at this temperature for another 6 h and then poured into cold water (15 mL), and extracted with dichloromethane (20 mL×3). The combined organic layers were washed with water, and brine, and dried over sodium sulfate. Removal of the solvent under reduced pressure followed by plug of regular silica gel column chromatographic purification using 0.5–2 % EtOAc/dichloromethane afforded **11 a** as a yellow sticky solid (102 mg, 74 %), dr=3.7 : 1 (by ^1^H NMR); IR (thin film) 2992, 2932, 2837, 1747, 1710, 1511, 1250, 1117, 1027, 696 cm^−1^; ^1^H NMR (400 MHz, CDCl_3_, TMS, Major isomer): δ 7.35–7.21 (m, 4H), 7.21–7.06 (m, 8H), 6.99–6.90 (m, 2H), 4.36 (s, 1H), 3.82 (s, 3H), 3.75–3.64 (m, 1H), 3.26 (t, *J*=9.9 Hz, 1H), 2.02 (dd, *J*=16.9 & 9.0 Hz, 1H), 1.68 (s, 3H), 1.67–1.56 (m, 1H), 1.20 (s, 3H); ^13^C NMR (100 MHz, CDCl_3_, Major isomer): δ 171.7, 171.0, 160.5, 140.2, 136.5, 130.6, 128.9, 128.6, 128.3, 128.0, 127.83, 127.78, 126.8, 126.4, 126.1, 114.9, 72.5, 57.6, 55.5, 47.9, 31.6, 30.1, 18.3; (M^+^H)^+^ HRMS m/z calcd for (C_29_H_29_N_2_O_4_)^+^: 469.2127; found: 469.2127.

### Isomerization experiment

#### Treatment of crude product (ent‐1 e) with KO^t^Bu

To a solution of *ent‐* **1 e** (23 mg, 0.09 mmol) in THF (37 M) at 0 °C, was added KO^t^Bu (1 M in hexane, 0.022 mL, 0.25 equiv.) dropwise. The mixture was heated to 50 °C, and stirred for 30 min. The reaction was quenched with HCl (0.1 M, ∼5 mL) added dropwise, and extracted into dichloromethane. The solvent was evaporated to afford the crude product for dr measurement by ^1^H NMR (dr 3.4 : 1).

### Determination of absolute and relative stereochemistry

For **1**: X‐ray crystallographic analysis of *ent*‐**1 d** revealed the relative configuration to be *trans* (*anti*), while the absolute configuration, was determined to be the (2*S*,3*R*)‐enantiomer.^[13]^

For **11**: Relative stereochemistry of **11 b** was determined to be *trans* (*anti*) by X‐ray crystallography. Deposition Number 2073137 (for **11 b**) contains the supplementary crystallographic data for this paper. These data are provided free of charge by the joint Cambridge Crystallographic Data Centre and Fachinformationszentrum Karlsruhe Access Structures service.

## Conflict of interest

The authors declare no conflict of interest.

1

## Supporting information

As a service to our authors and readers, this journal provides supporting information supplied by the authors. Such materials are peer reviewed and may be re‐organized for online delivery, but are not copy‐edited or typeset. Technical support issues arising from supporting information (other than missing files) should be addressed to the authors.

Supporting Information

## Data Availability

The data that support the findings of this study are available in the supplementary material of this article.
