# Supplementary material for: Asymmetric Synthesis of Bicyclic Pyrazolidinones through Alkaloid‐Catalyzed [3+2]‐Cycloadditions of Ketenes and Azomethine Imines
Source: Chemistry. 2022 Mar 14;28(21):e202104391. doi: 10.1002/chem.202104391 (PMC9311188; doi:10.1002/chem.202104391)
Supplement: Supplementary file 1 — Supporting Information [file CHEM-28-0-s001.pdf]

Chemistry–A European Journal

Supporting Information

Asymmetric Synthesis of Bicyclic Pyrazolidinones through Alkaloid-Catalyzed [3+2]-Cycloadditions of Ketenes and Azomethine Imines

Mukulesh Mondal, Shubhanjan Mitra, Dylan J. Twardy, Manashi Panda, Kraig A. Wheeler, and Nessan J. Kerrigan*

NMR and HPLC spectra

Table of Contents:

HPLC of (±)-**1a** S4

HPLC of *ent*-**1a** S5

^{1}H & ^{13}C NMR of *ent*-**1a** S6

HPLC of (±)-**1b** S7

HPLC of *ent*-**1b** S8

^{1}H & ^{13}C NMR of *ent*-**1b** S9

HPLC of (±)-**1e** S10

HPLC of **1e** S11

HPLC of *ent*-**1e** S12

^{1}H & ^{13}C NMR of **1e** S13

^{1}H & ^{13}C NMR of *ent*-**1e** S14

HPLC of (±)-**1f** S15

HPLC of **1f** S16

HPLC of *ent*-**1f** S17

^{1}H & ^{13}C NMR of **1f** S18

^{1}H & ^{13}C NMR of *ent*-**1f** S19

HPLC of (±)-**1h** S20

HPLC of **1h** S21

HPLC of *ent*-**1h** S22

^{1}H & ^{13}C NMR of **1h** S23

^{1}H & ^{13}C NMR of *ent*-**1h** S24

HPLC of (±)-**1i**	S25
HPLC of **1i**	S26
HPLC of *ent*-**1i**	S27
^{1}H & ^{13}C NMR of **1i**	S28
^{1}H & ^{13}C NMR of *ent*-**1i**	S29
HPLC of (±)-**1j**	S30
HPLC of **1j**	S31
HPLC of *ent*-**1j**	S32
^{1}H NMR of **1j**	S33
^{13}C NMR of **1j**	S34
^{1}H & ^{13}C NMR of *ent*-**1j**	S35
HPLC of (±)-**1k**	S36
HPLC of *ent*-**1k**	S37
^{1}H & ^{13}C NMR of *ent*-**1k**	S38
HPLC of (±)-**1l**	S39
HPLC of *ent*-**1l**	S40
^{1}H & ^{13}C NMR of *ent*-**1l**	S41
HPLC of (±)-**1m**	S42
HPLC of *ent*-**1m**	S43
^{1}H & ^{13}C NMR of *ent*-**1m**	S44
HPLC of (±)-**1p**	S45
HPLC of **1p**	S46
HPLC of *ent*-**1p**	S47
^{1}H & ^{13}C NMR of **1p**	S48
^{1}H & ^{13}C NMR of *ent*-**1p**	S49
HPLC of (±)-**1r**	S50
HPLC of **1r**	S51
HPLC of *ent*-**1r**	S52
^{1}H & ^{13}C NMR of **1r**	S53
^{1}H & ^{13}C NMR of *ent*-**1r**	S54
HPLC of (±)-**1s**	S55
HPLC of **1s**	S56
HPLC of *ent*-**1s**	S57
^{1}H & ^{13}C NMR of **1s**	S58

^{1}H & ^{13}C NMR of *ent*-**1s** S59
^{1}H & ^{13}C NMR of (±)-**11a** S60
^{1}H & ^{13}C NMR of (±)-**11b** S61
^{1}H & ^{13}C NMR of (±)-**11c** S62
^{1}H & ^{13}C NMR of (±)-**1t** S63
^{1}H NMR of **1d (scale-up experiment)** S64
^{13}C NMR of **1d (scale-up experiment)** S65

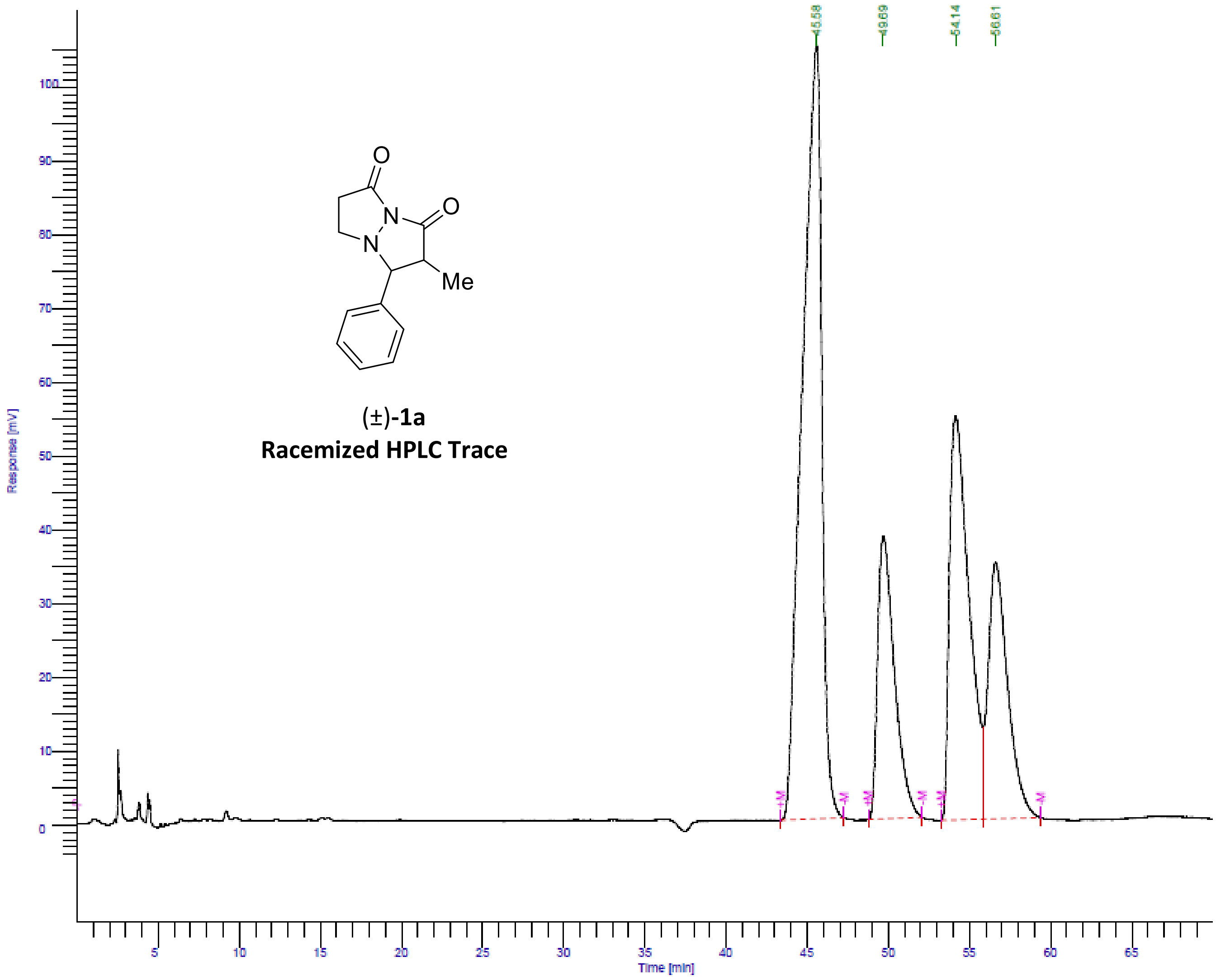

Peak #	Time [min]	Area [µV·s]	Height [µV]	Area [%]	Norm. Area [%]	BL	Area/Height [s]
1	45.579	8679115.68	104901.86	45.76	45.76	*BB	82.7356
2	49.690	2772011.24	38239.60	14.61	14.61	*BB	72.4906
3	54.143	4579552.62	54794.33	24.14	24.14	*BV	83.5771
4	56.611	2937955.14	34764.70	15.49	15.49	*VB	84.5097
		18968634.68	232700.49	100.00	100.00		

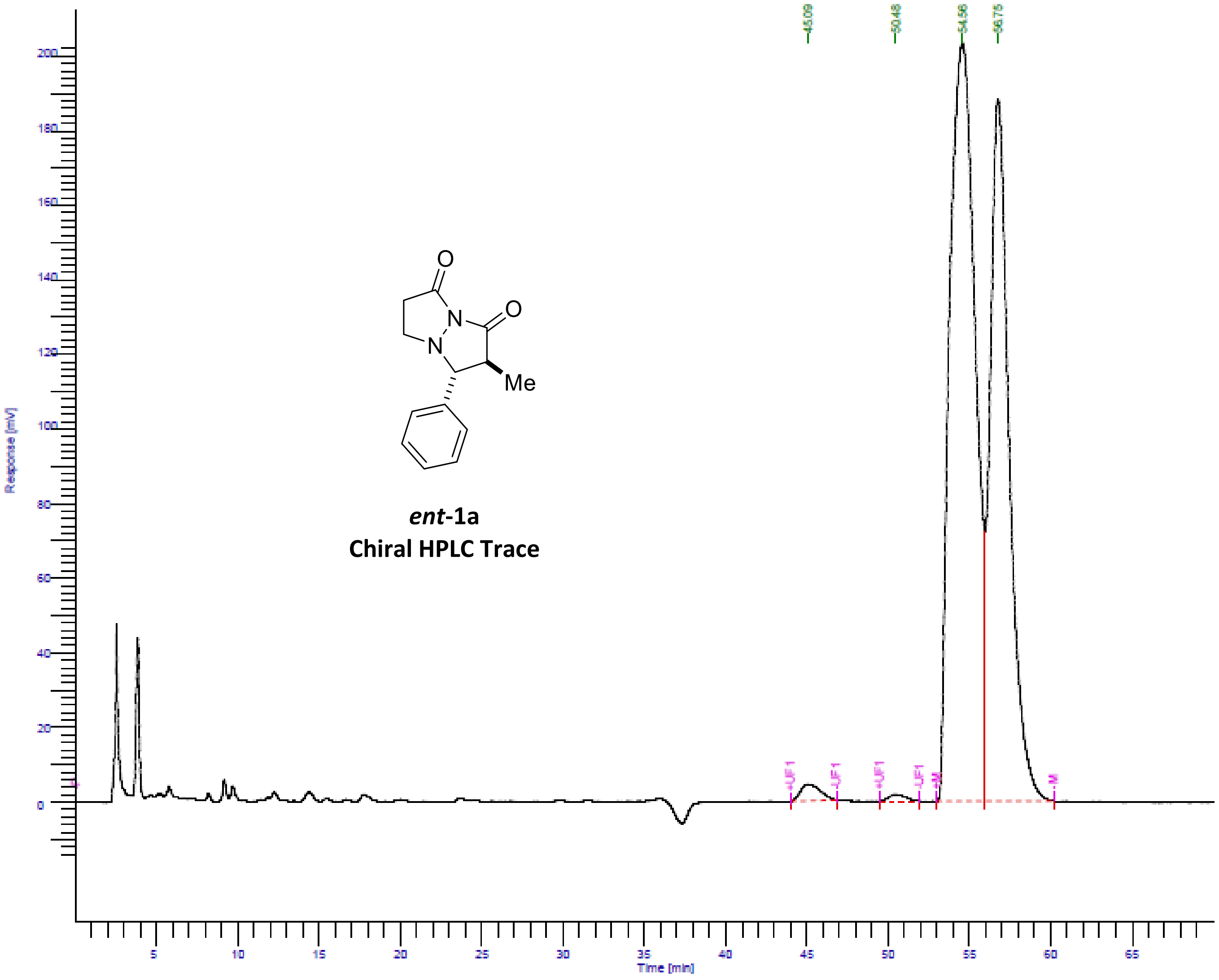

***ent*-1a**
Chiral HPLC Trace

Peak #	Time [min]	Area [µV·s]	Height [µV]	Area [%]	Norm. Area [%]	BL	Area/Height [s]
1	45.085	376755.30	4502.37	0.98	0.98	*MM	83.6794
2	50.475	128366.14	1831.56	0.34	0.34	*MM	70.0857
3	54.560	22338169.50	203068.63	58.32	58.32	*BV	110.0031
4	56.754	15461504.94	188072.72	40.36	40.36	*VB	82.2102
		38304795.88	397475.28	100.00	100.00		

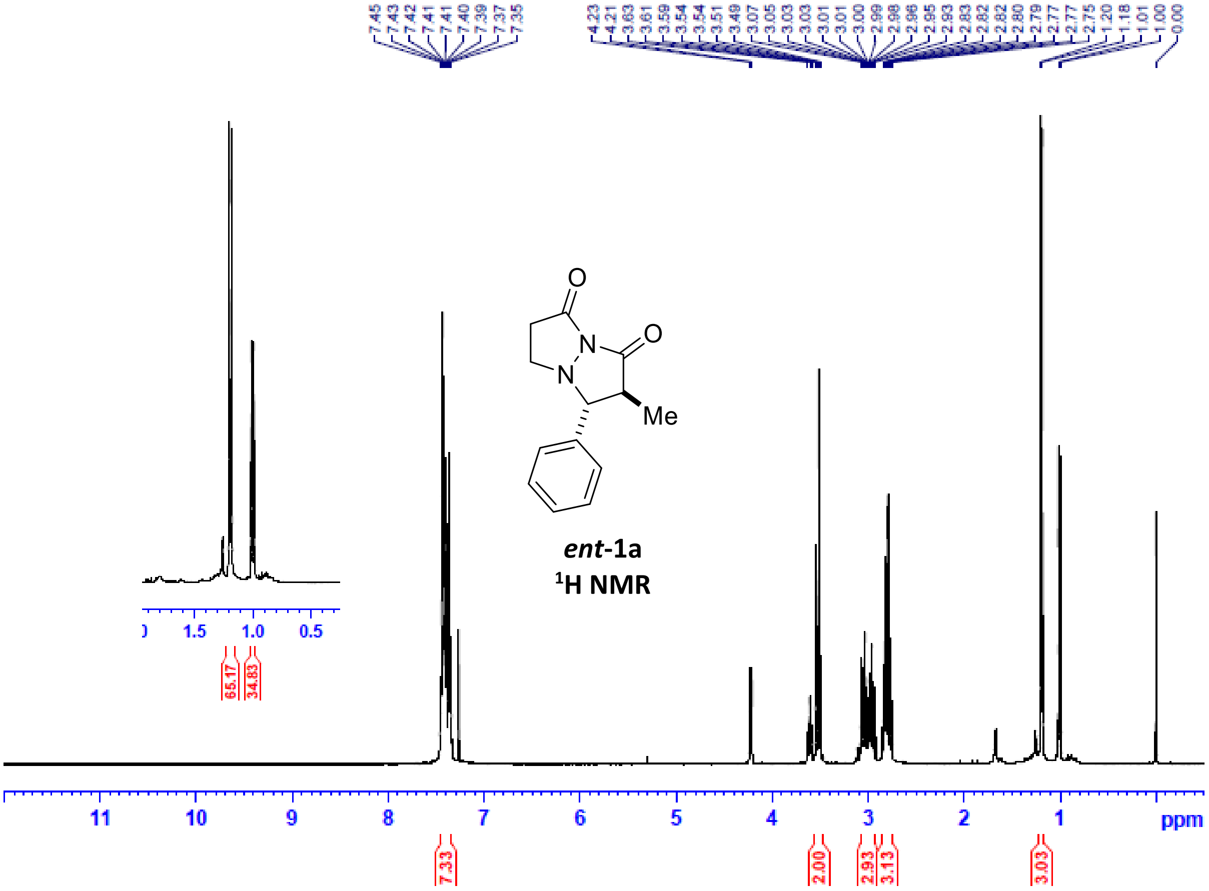
ent-1a
1H NMR
ppm

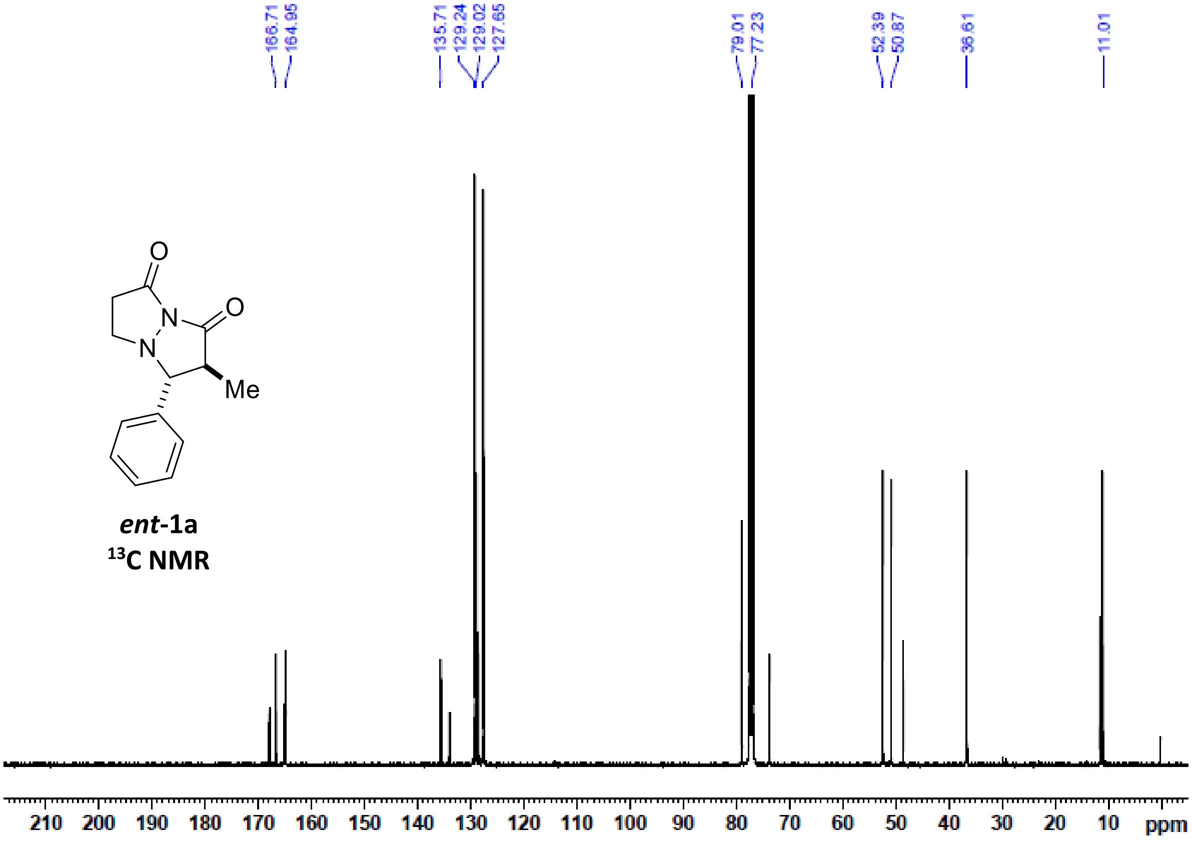
ent-1a
13C NMR
ppm

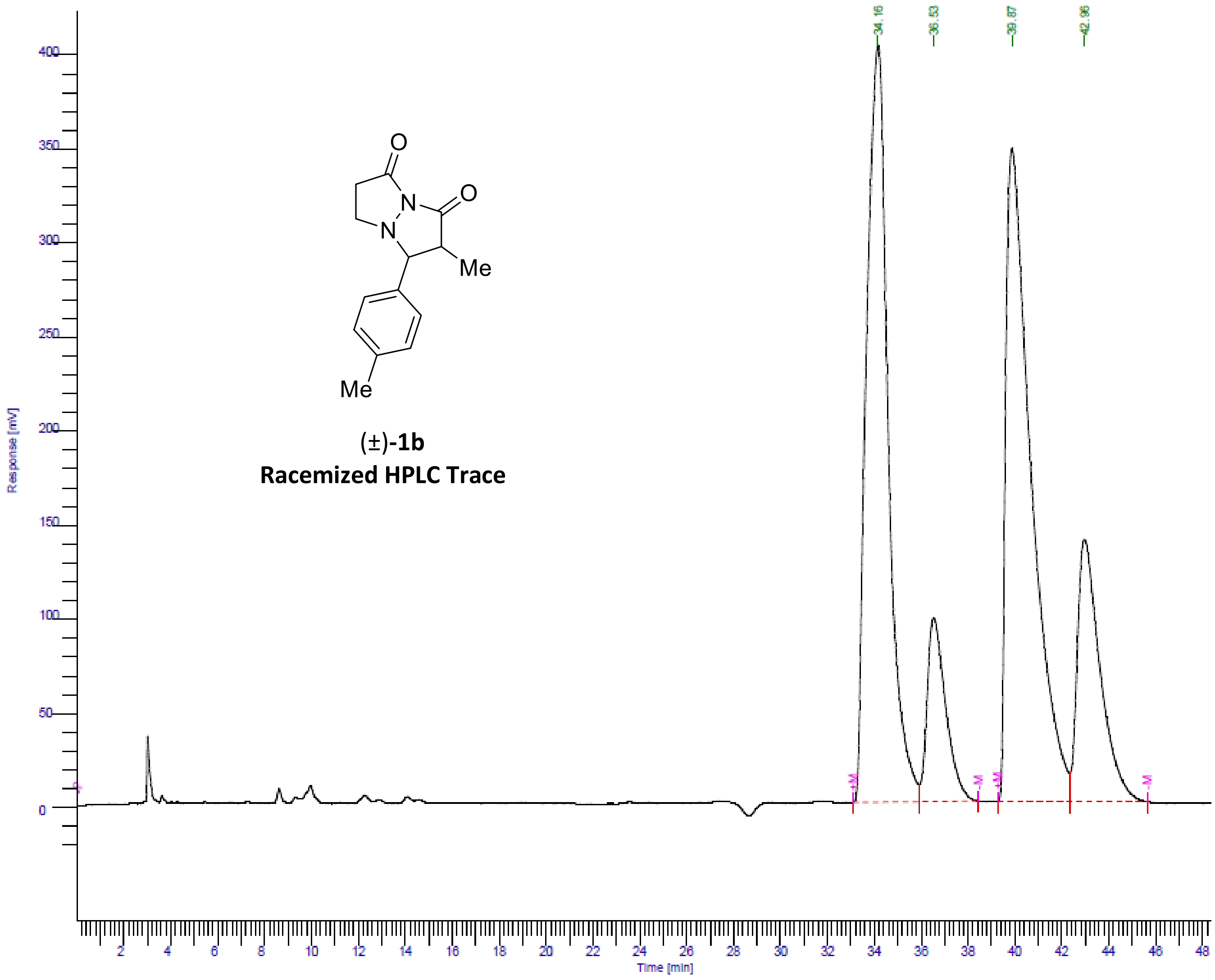

(±)-**1b**
Racemized HPLC Trace

Peak #	Time [min]	Area [µV·s]	Height [µV]	Area [%]	Norm. Area [%]	BL	Area/Height [s]
1	34.160	25706389.53	402358.37	38.51	38.51	*BV	63.8893
2	36.531	5397168.25	97687.76	8.08	8.08	*VB	55.2492
3	39.867	26186004.81	347454.64	39.23	39.23	*BV	75.3652
4	42.965	9467715.69	139476.49	14.18	14.18	*VB	67.8804
		66757278.29	986977.26	100.00	100.00		

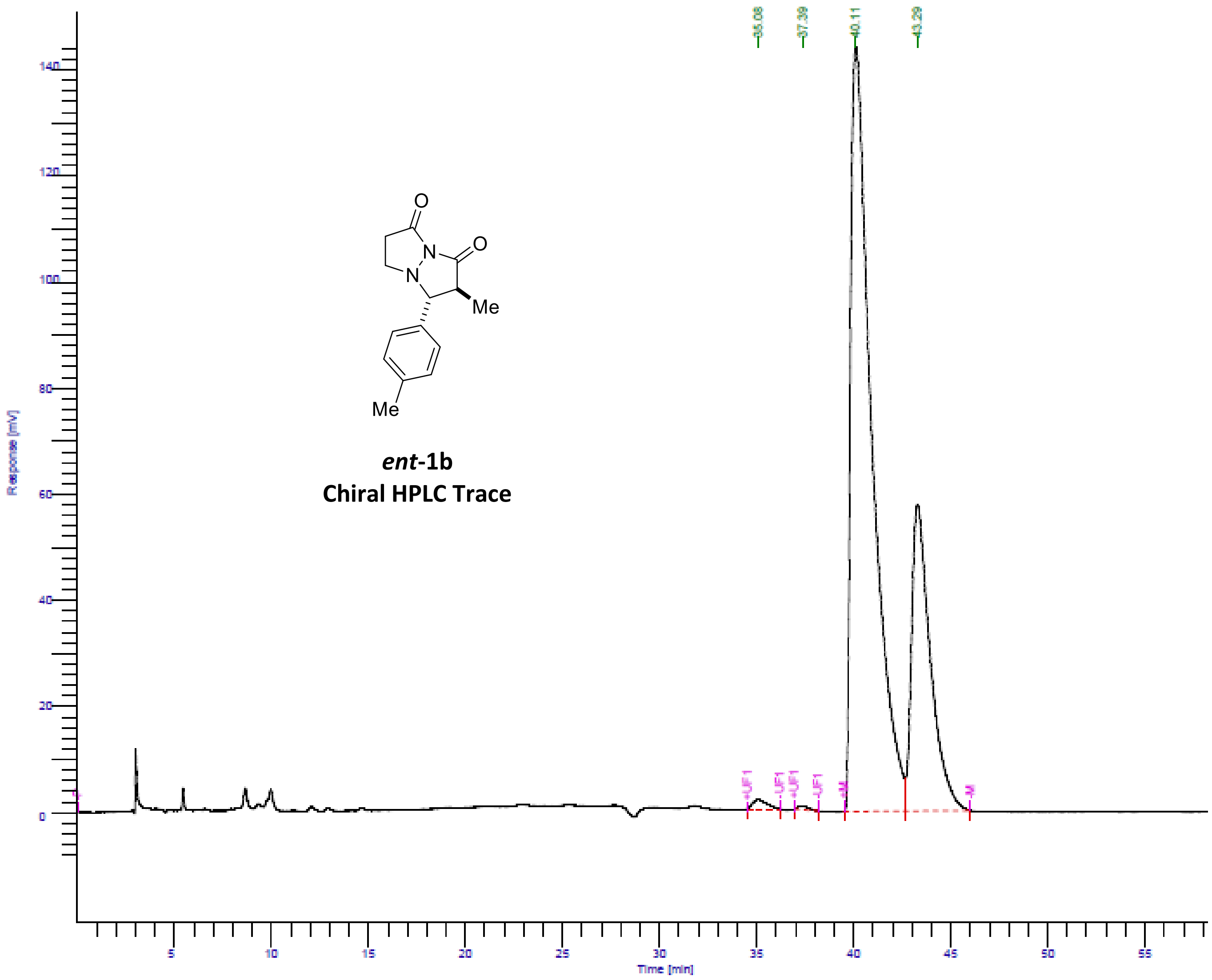

Peak #	Time [min]	Area [µV·s]	Height [µV]	Area [%]	Norm. Area [%]	BL	Area/Height [s]
1	35.075	89304.34	1719.32	0.60	0.60	*MM	51.9418
2	37.385	25535.65	657.67	0.17	0.17	*MM	38.8272
3	40.108	10884413.14	144101.83	73.12	73.12	*BV	75.5328
4	43.286	3885524.10	57761.80	26.10	26.10	*VB	67.2681
		14884777.23	204240.62	100.00	100.00		

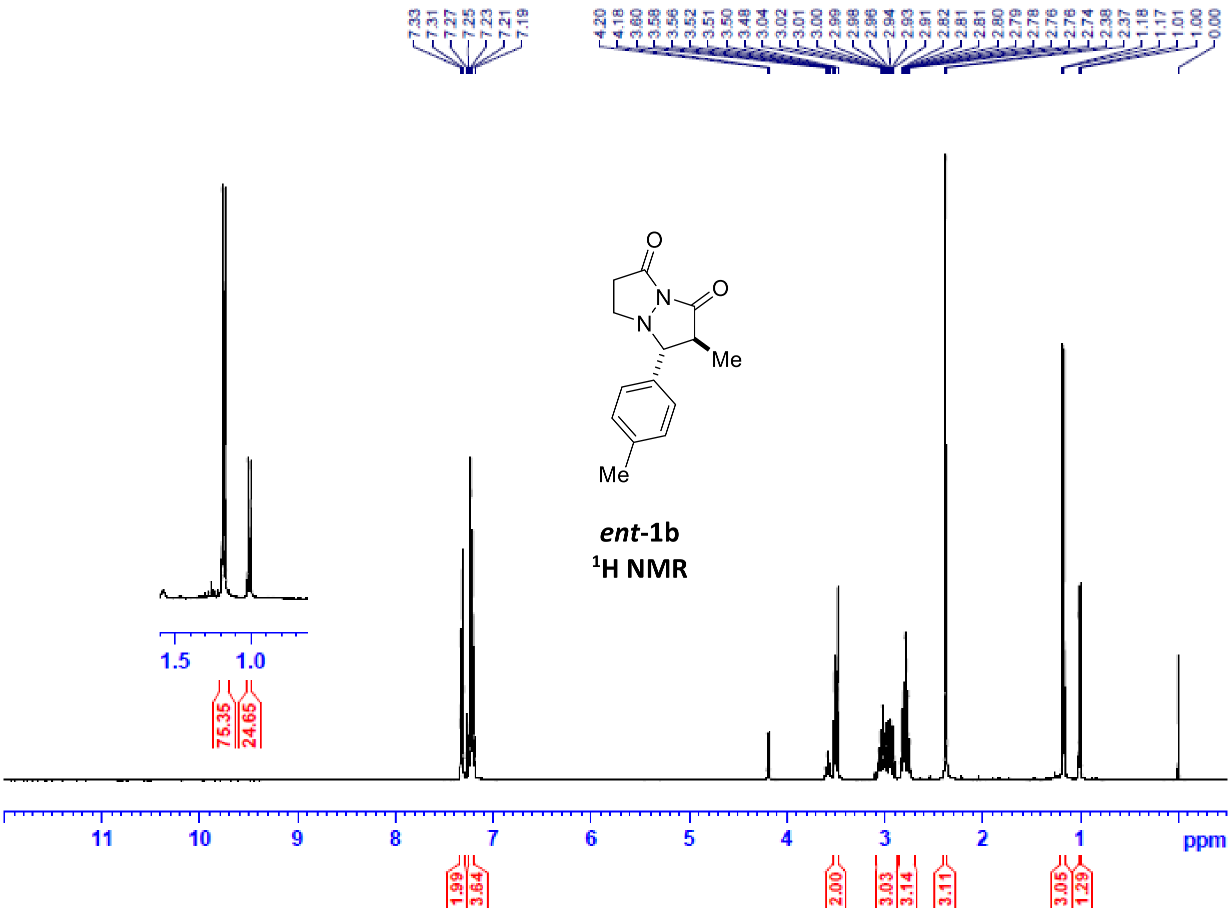
ent-1b
1H NMR
ppm

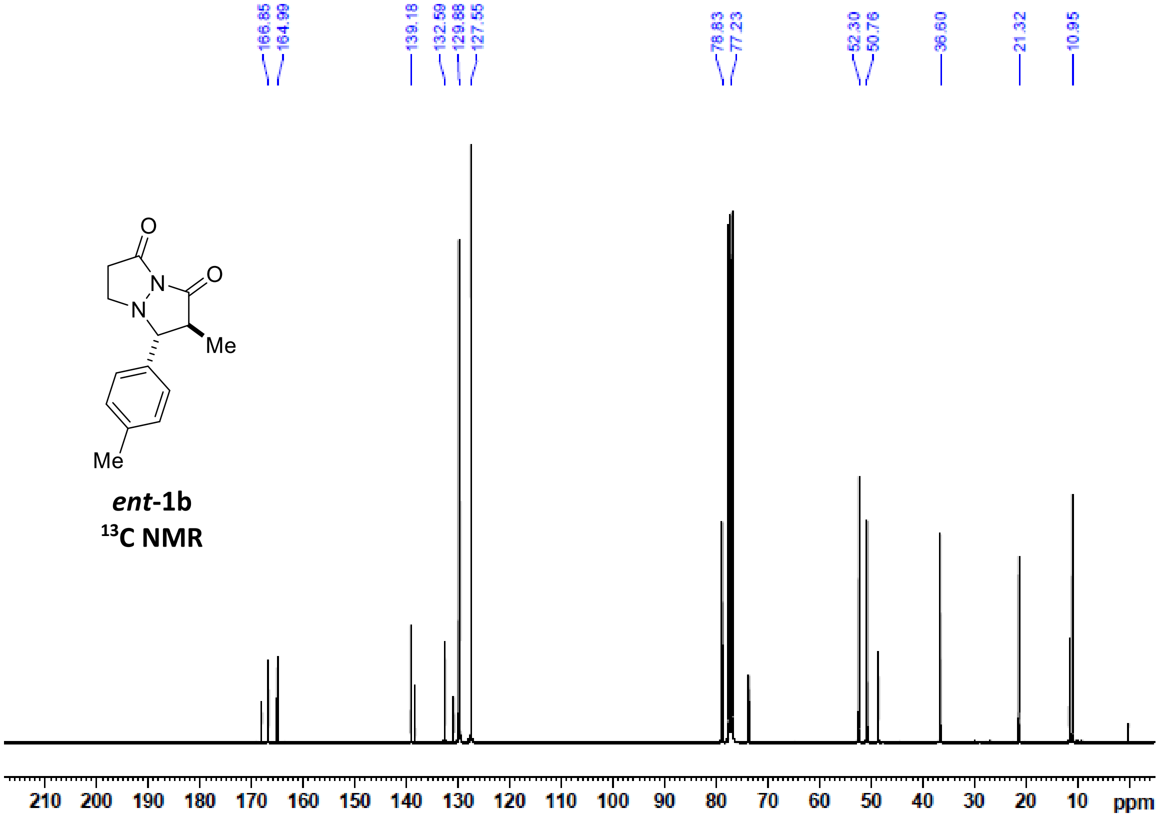
ent-1b
13C NMR
ppm

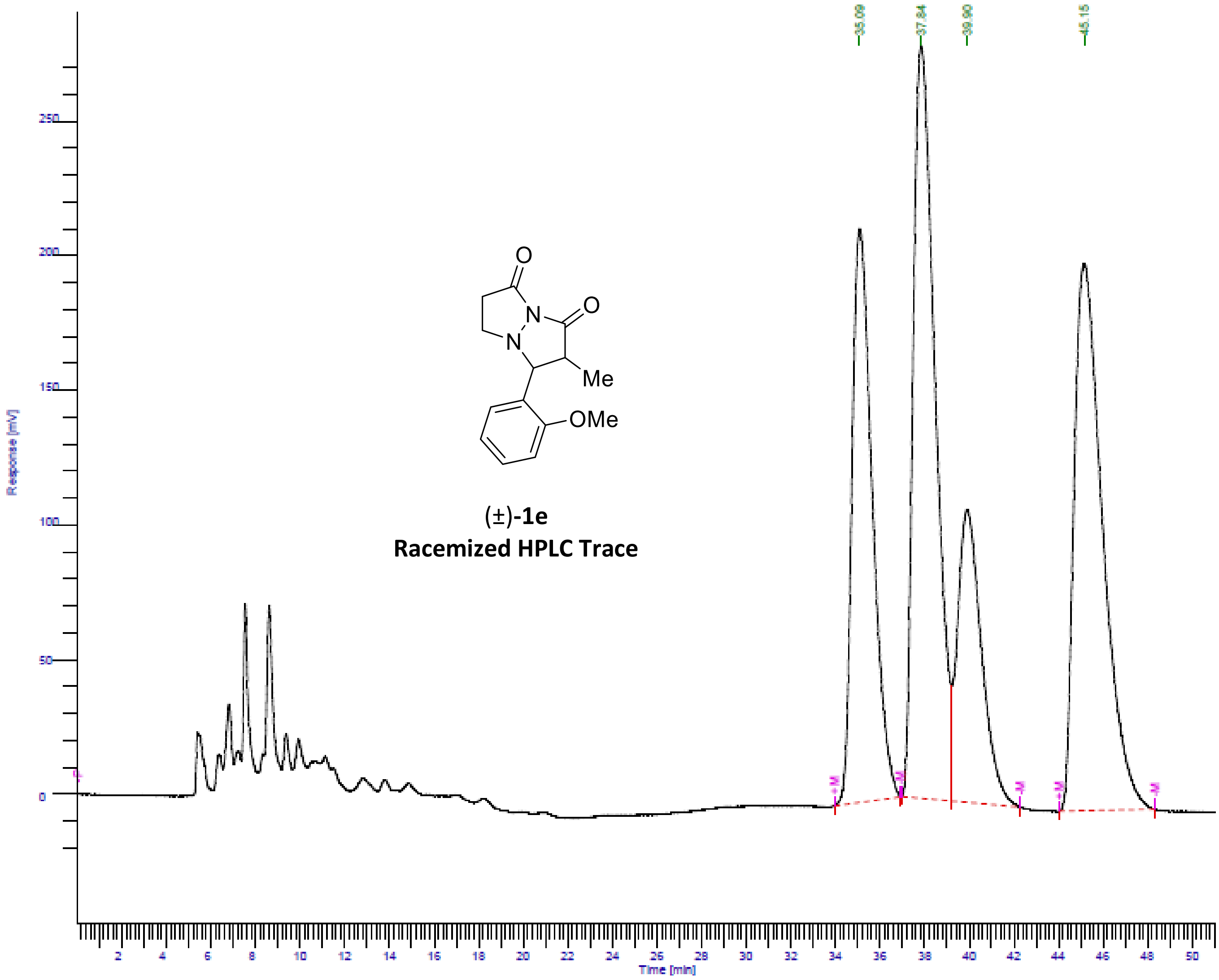

Peak #	Time [min]	Area [µV·s]	Height [µV]	Area [%]	Norm. Area [%]	BL	Area/Height [s]
1	35.089	13447179.71	213184.02	22.89	22.89	*BB	63.0778
2	37.839	19177311.14	279571.89	32.65	32.65	*BV	68.5953
3	39.904	8084623.28	108727.72	13.76	13.76	*VB	74.3566
4	45.146	18031337.27	203307.04	30.70	30.70	*BB	88.6902
		58740451.40	804790.66	100.00	100.00		

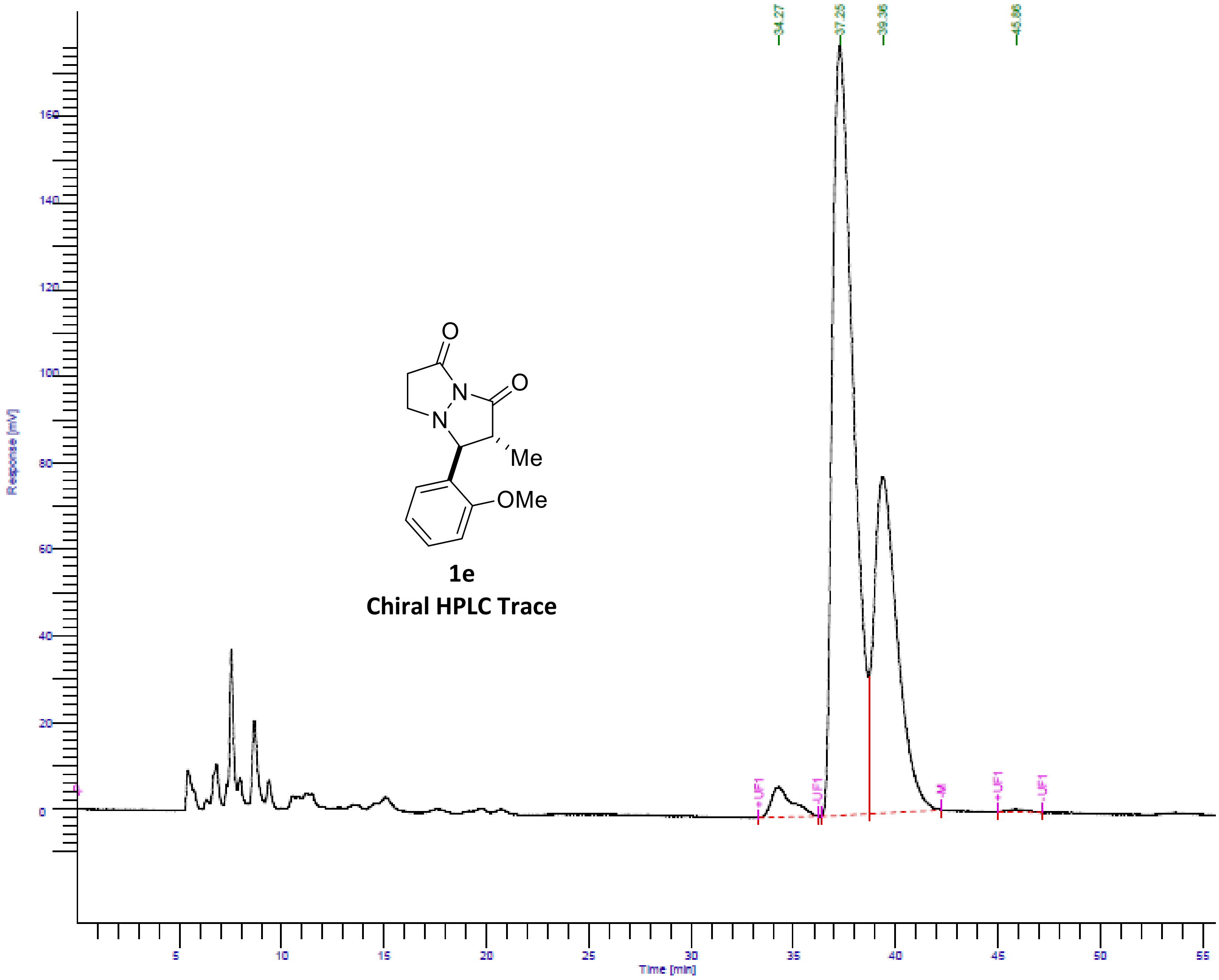

Peak #	Time [min]	Area [µV·s]	Height [µV]	Area [%]	Norm. Area [%]	BL	Area/Height [s]
1	34.269	494994.39	6940.19	2.55	2.55	*MM	71.3229
2	37.255	12675084.91	178052.88	65.21	65.21	*BV	71.1872
3	39.358	6228625.10	77794.44	32.04	32.04	*VB	80.0652
4	45.863	39764.33	644.26	0.20	0.20	*MM	61.7214
		19438468.73	263431.76	100.00	100.00		

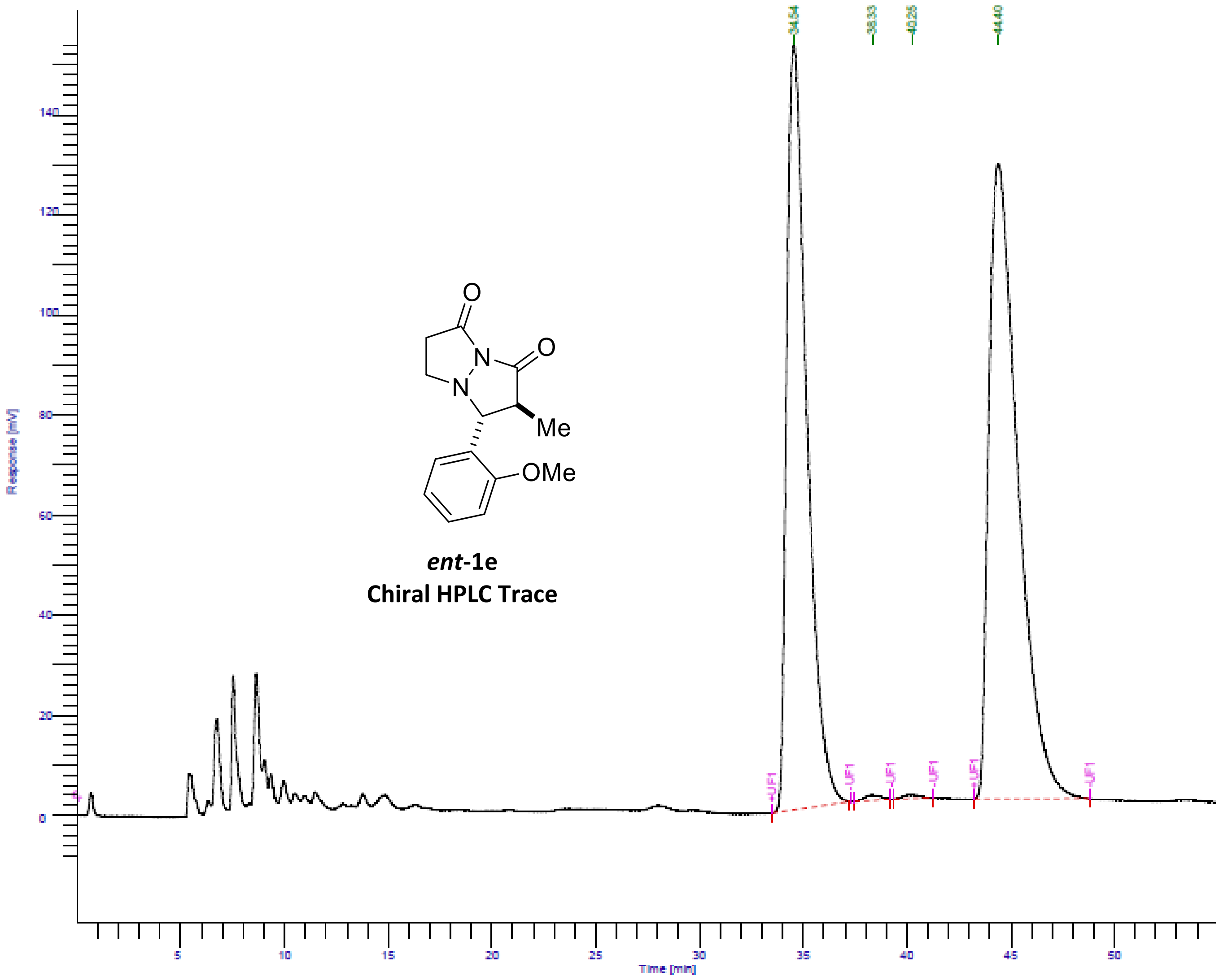

***ent*-1e**
Chiral HPLC Trace

Peak #	Time [min]	Area [µV·s]	Height [µV]	Area [%]	Norm. Area [%]	BL	Area/Height [s]
1	34.540	10319664.74	152975.13	45.37	45.37	*MM	67.4598
2	38.331	55695.89	1082.28	0.24	0.24	*MM	51.4614
3	40.245	43855.89	804.86	0.19	0.19	*MM	54.4887
4	44.396	12328441.74	127094.95	54.20	54.20	*MM	97.0018
		22747658.26	281957.23	100.00	100.00		

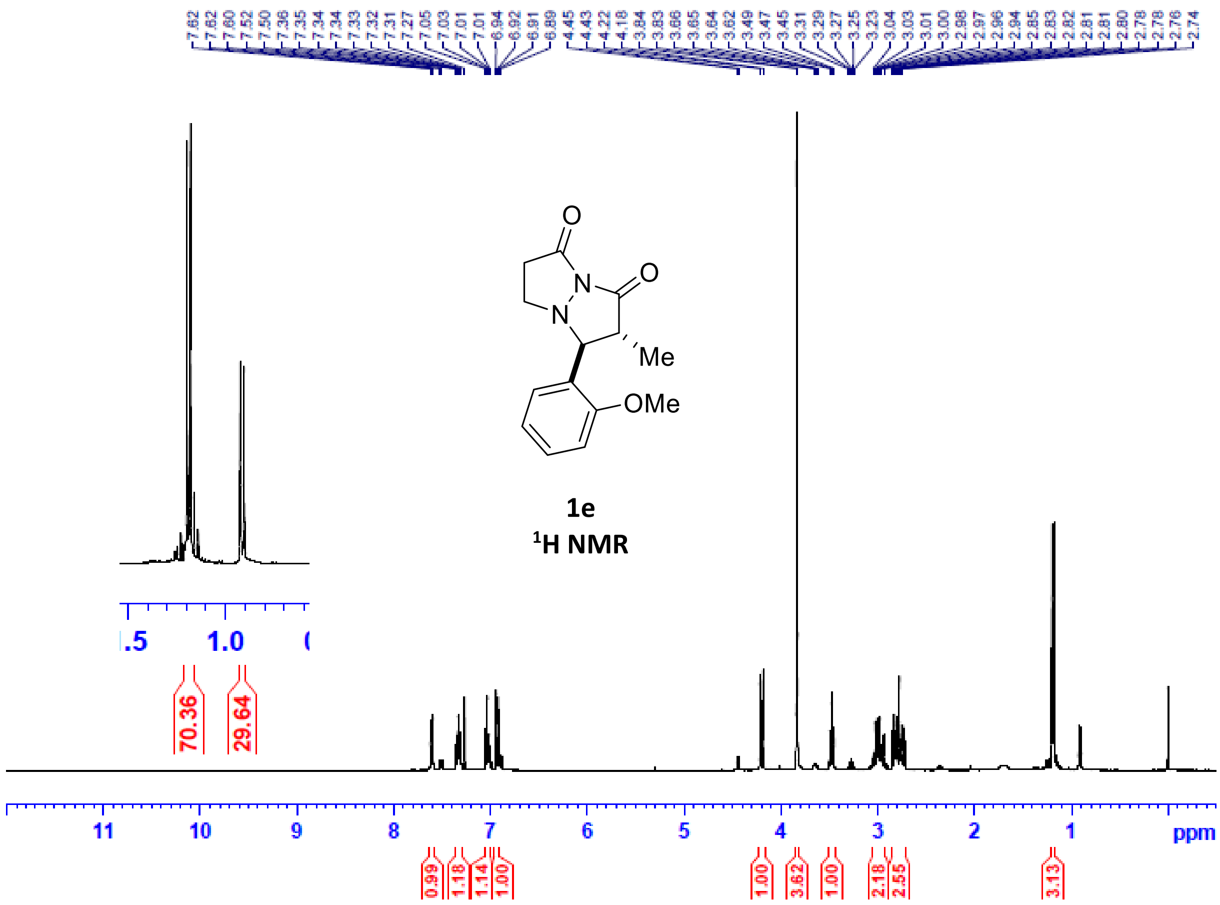
7.62 7.62 7.60 7.52 7.50 7.36 7.35 7.34 7.33 7.32 7.31 7.27 7.05 7.03 7.01 7.01 6.94 6.92 6.91 6.89 4.45 4.43 4.22 4.18 3.84 3.83 3.66 3.65 3.64 3.62 3.49 3.47 3.45 3.31 3.29 3.27 3.25 3.23 3.04 3.03 3.01 3.00 2.98 2.97 2.96 2.94 2.85 2.83 2.82 2.81 2.81 2.80 2.78 2.78 2.76 2.74
O
N
N
O
Me
OMe
1e
¹H NMR
.5
1.0
70.36
29.64
11
10
9
8
7
6
5
4
3
2
1
ppm
0.99
1.18
1.14
1.00
1.00
3.62
1.00
2.18
2.55
3.13

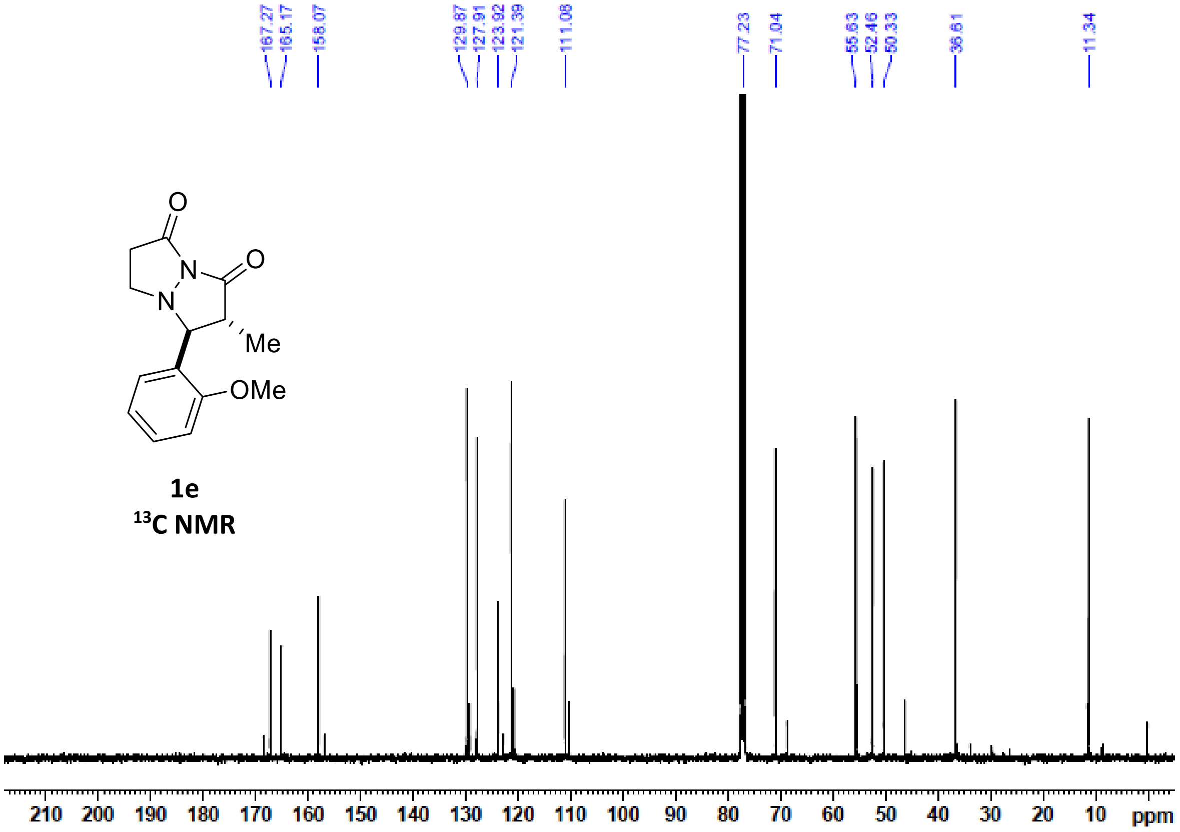
167.27
165.17
158.07
129.87
127.91
123.92
121.39
111.08
77.23
71.04
55.63
52.46
50.33
36.61
11.34
O
N
N
O
Me
OMe
1e
¹³C NMR
210 200 190 180 170 160 150 140 130 120 110 100 90 80 70 60 50 40 30 20 10 ppm

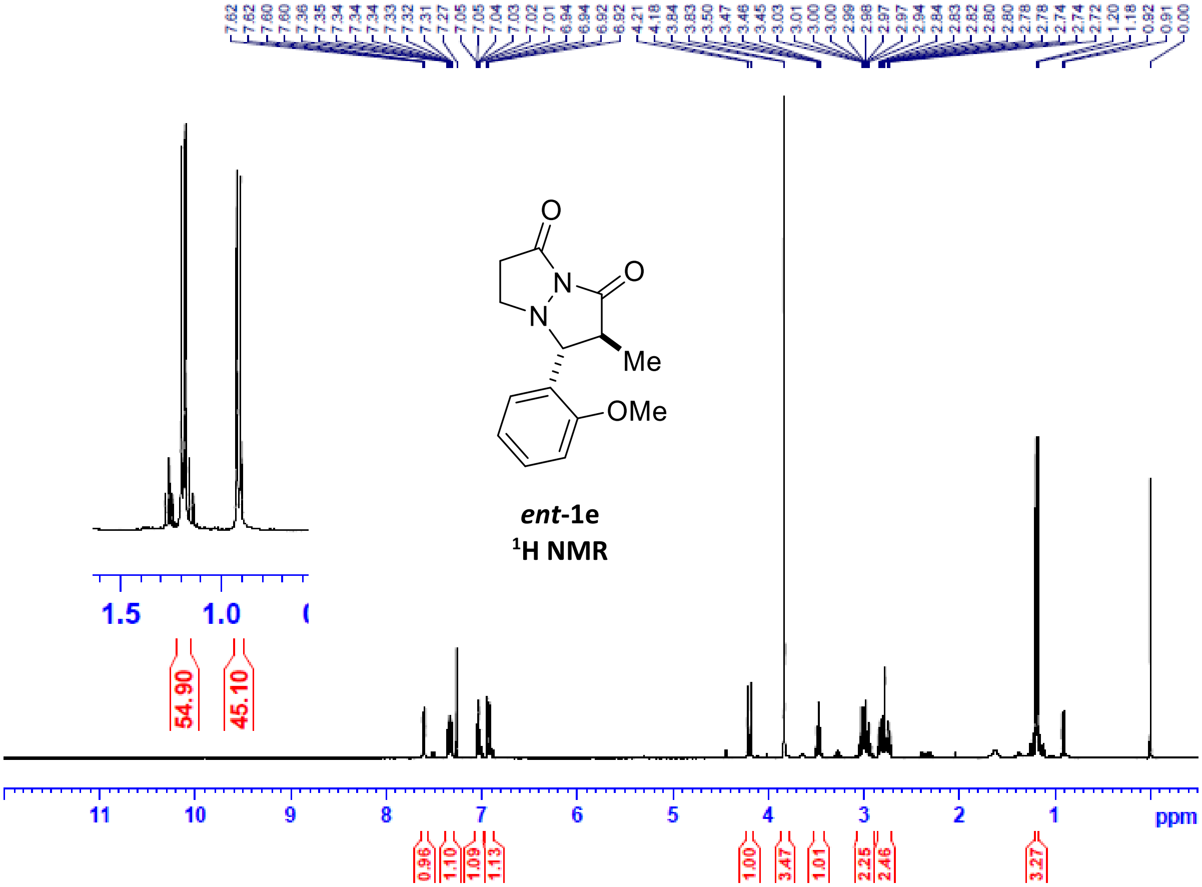

O
N
O
N
Me
OMe
ent-1e
¹H NMR
1.5
1.0
54.90
45.10
11
10
9
8
7
6
5
4
3
2
1
ppm
0.96
1.10
1.09
1.13
1.00
3.47
1.01
2.25
2.46
3.27

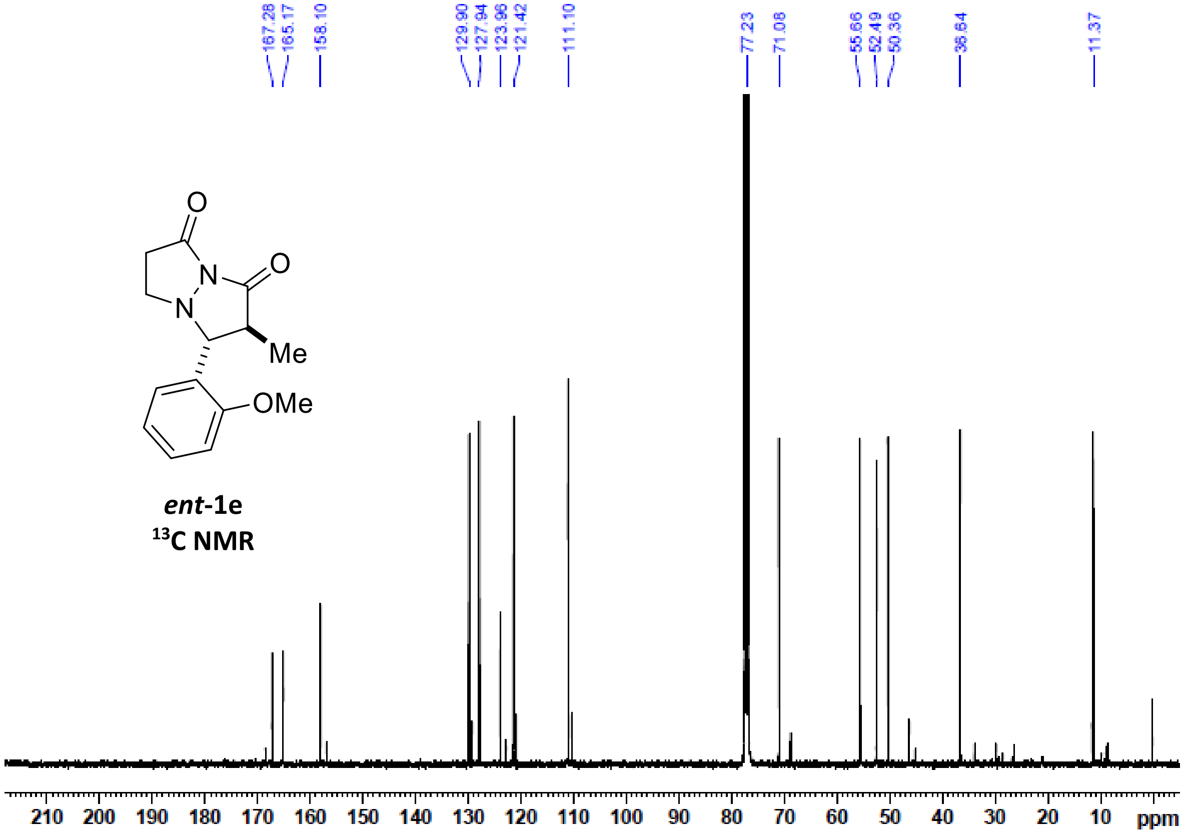

167.28
165.17
158.10
129.90
127.94
123.96
121.42
111.10
77.23
71.08
55.66
52.49
50.36
36.64
11.37
O
N
O
N
Me
OMe
ent-1e
¹³C NMR
210
200
190
180
170
160
150
140
130
120
110
100
90
80
70
60
50
40
30
20
10
ppm

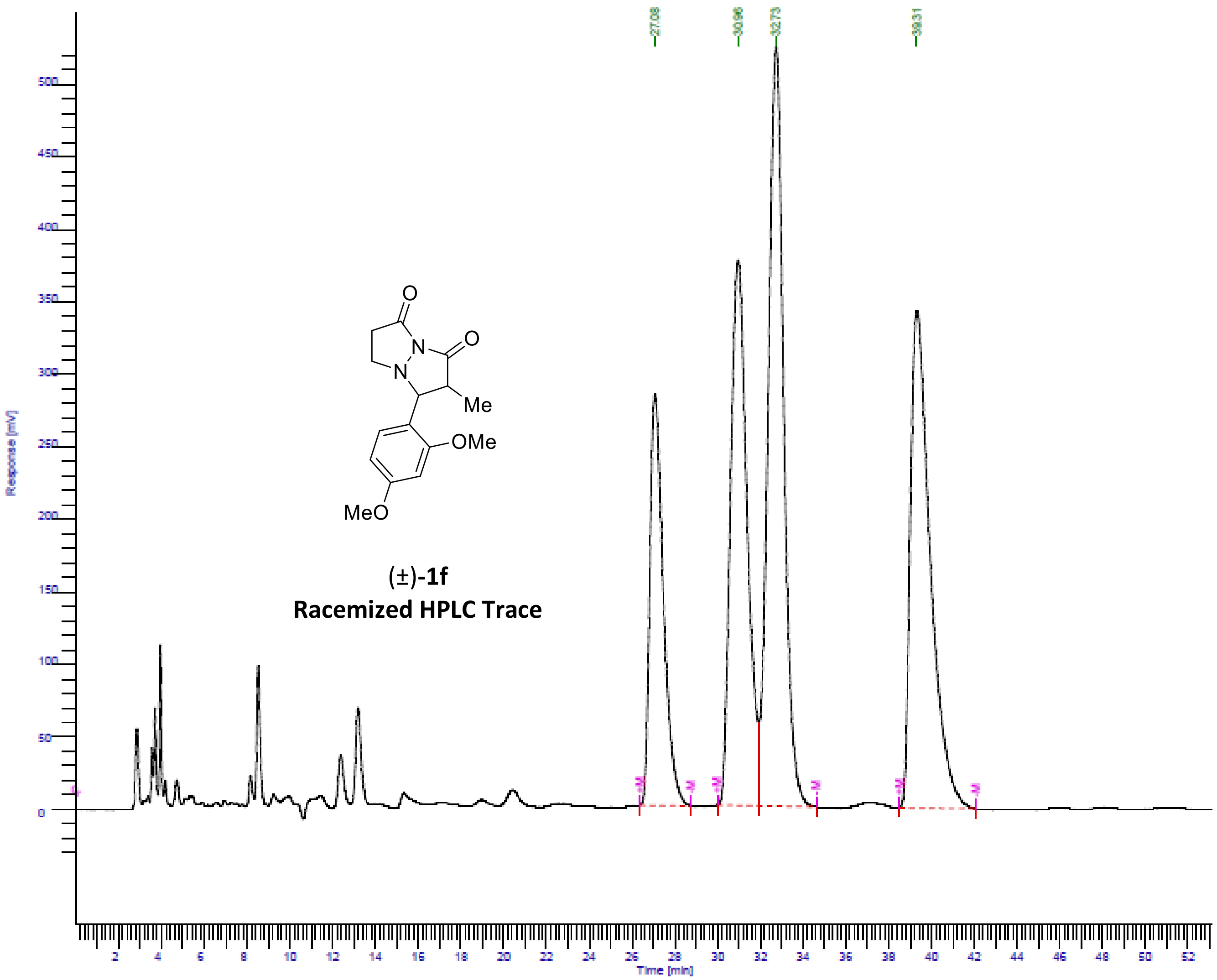

Peak #	Time [min]	Area [µV·s]	Height [µV]	Area [%]	Norm. Area [%]	BL	Area/Height [s]
1	27.077	12348277.23	283625.75	15.13	15.13	*BB	43.5372
2	30.958	20435438.09	376295.04	25.03	25.03	*BV	54.3070
3	32.728	26687879.09	524089.77	32.69	32.69	*VB	50.9223
4	39.313	22159375.11	343283.46	27.15	27.15	*BB	64.5512
		81630969.52	1.53e+06	100.00	100.00		

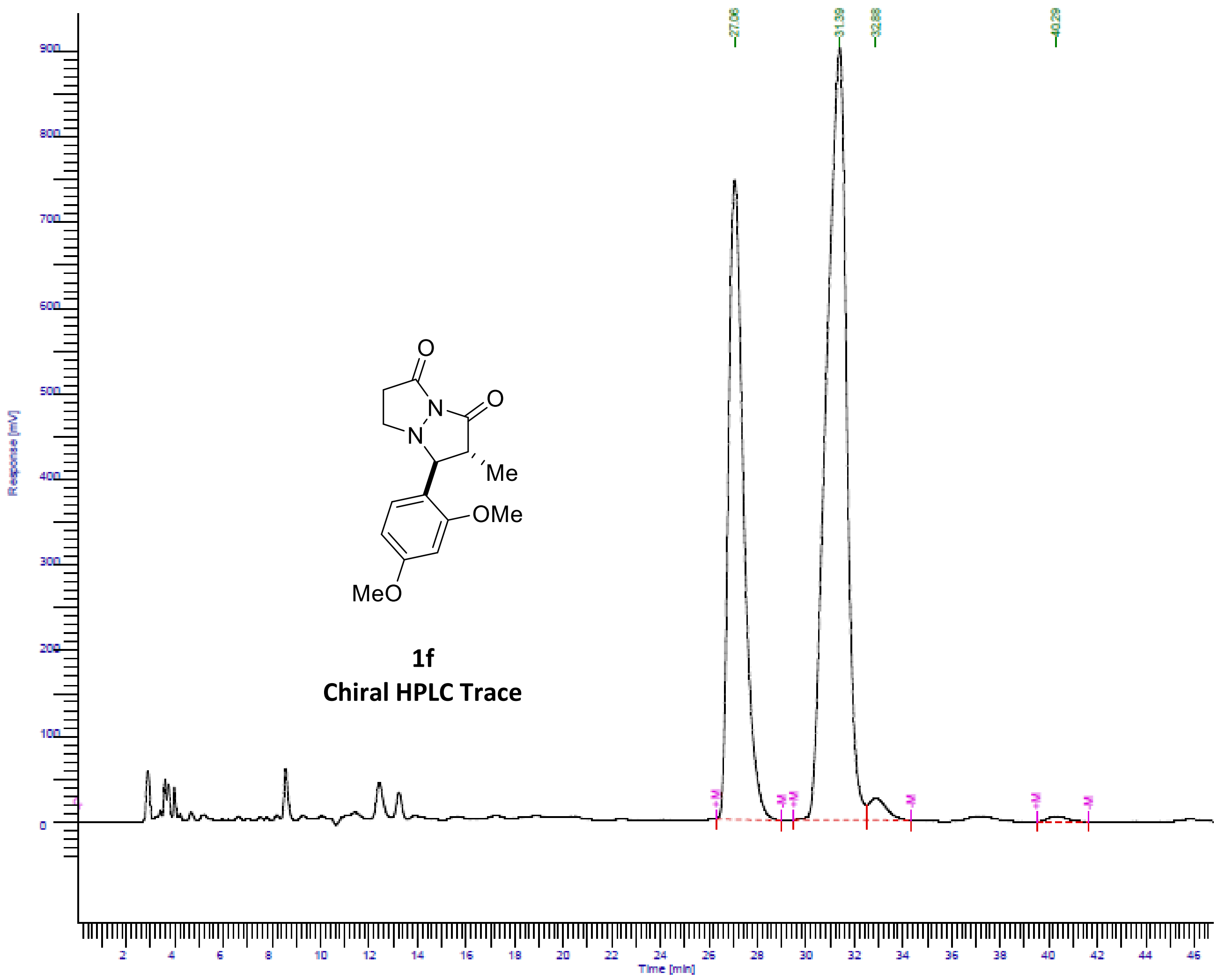

1f
Chiral HPLC Trace

Peak #	Time [min]	Area [µV·s]	Height [µV]	Area [%]	Norm. Area [%]	BL	Area/Height [s]
1	27.059	32214198.25	746584.09	37.46	37.46	*BB	43.1488
2	31.388	52113427.00	902560.40	60.61	60.61	*BV	57.7395
3	32.882	1318684.44	25460.51	1.53	1.53	*VB	51.7933
4	40.292	341790.52	6019.11	0.40	0.40	*BB	56.7842
		85988100.21	1.68e+06	100.00	100.00		

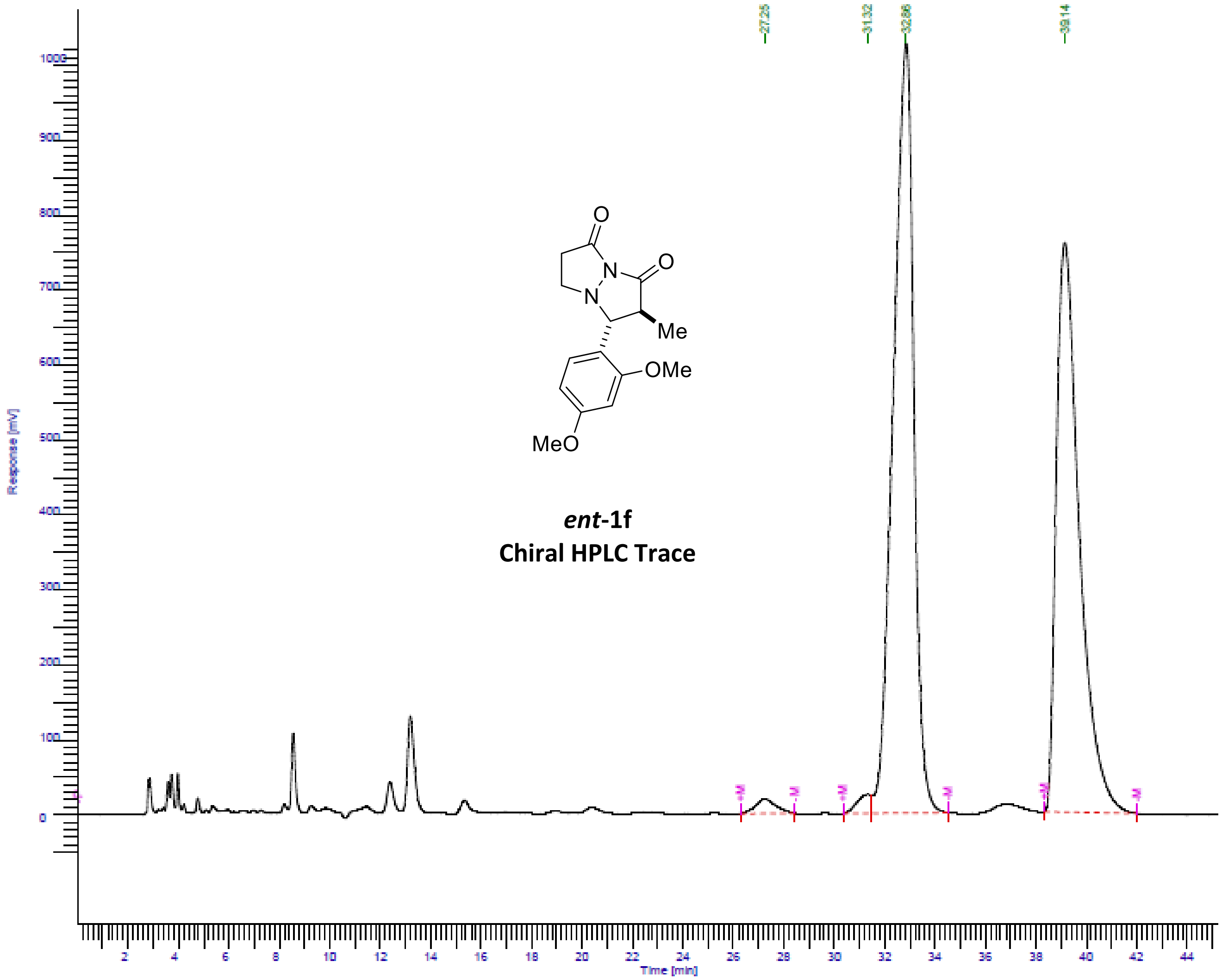

***ent*-1f**
Chiral HPLC Trace

Peak #	Time [min]	Area [µV·s]	Height [µV]	Area [%]	Norm. Area [%]	BL	Area/Height [s]
1	27.247	1098938.91	19541.67	1.02	1.02	*BB	56.2357
2	31.324	992074.57	25177.01	0.92	0.92	*BV	39.4040
3	32.859	57962911.73	1.03e+06	53.55	53.55	*VB	56.4629
4	39.145	48179217.56	759787.20	44.51	44.51	*BB	63.4115
		1.08e+08	1.83e+06	100.00	100.00		

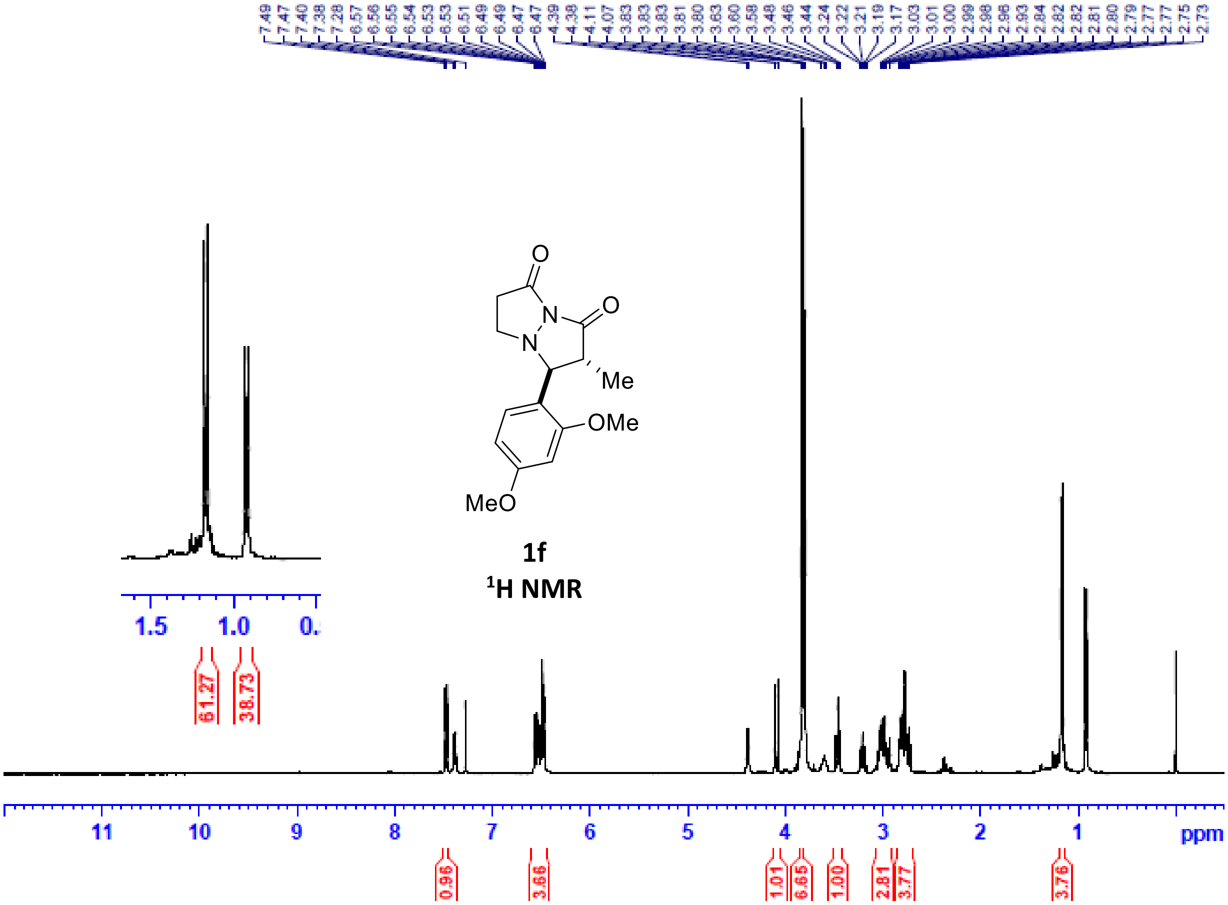
1f
1H NMR
ppm

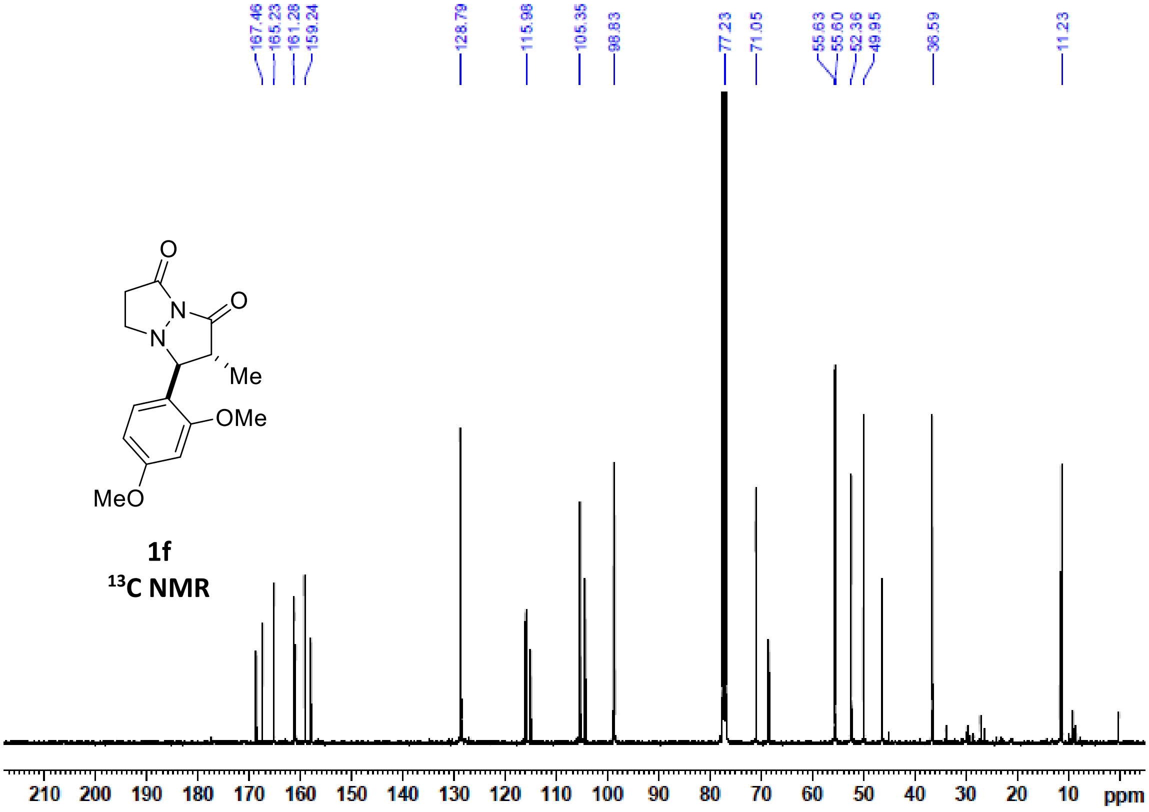
167.46
165.23
161.28
159.24
128.79
115.98
105.35
98.83
77.23
71.05
55.63
55.60
52.36
49.95
36.59
11.23
1f
13C NMR
ppm

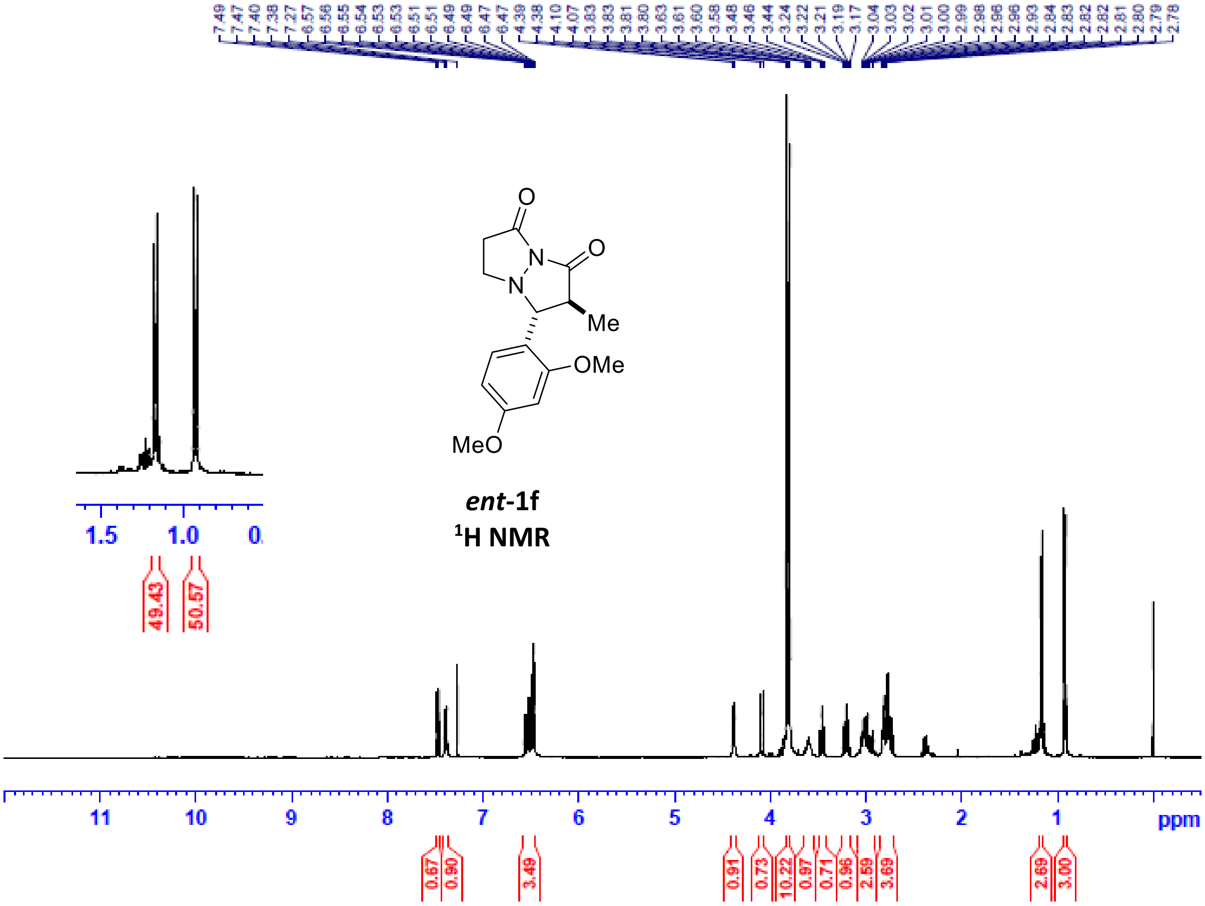
7.49 7.47 7.40 7.38 7.27 6.57 6.56 6.55 6.54 6.53 6.53 6.51 6.51 6.49 6.49 6.47 6.47 4.39 4.38 4.10 4.07 3.83 3.83 3.81 3.80 3.63 3.61 3.60 3.58 3.48 3.46 3.44 3.24 3.22 3.21 3.19 3.17 3.04 3.03 3.02 3.01 3.00 2.99 2.98 2.98 2.96 2.96 2.93 2.84 2.83 2.82 2.82 2.81 2.80 2.79 2.78
O
N
O
N
Me
OMe
MeO
ent-1f
1H NMR
1.5
1.0
49.43
50.57
11 10 9 8 7 6 5 4 3 2 1 ppm
0.67 0.90 3.49 0.91 0.73 10.22 0.97 0.71 0.96 2.59 3.69 2.69 3.00

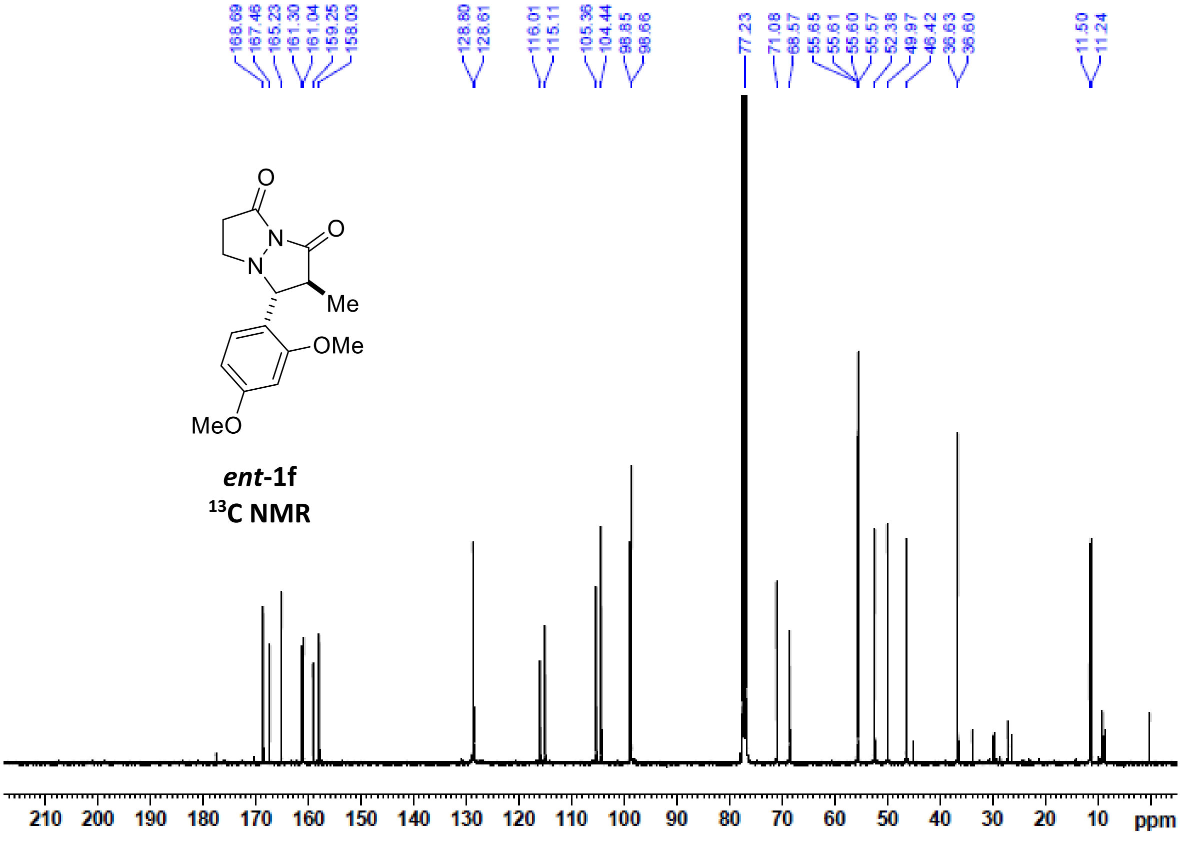
168.69 167.46 165.23 161.30 161.04 159.25 158.03 128.80 128.61 116.01 115.11 105.36 104.44 98.85 98.66 77.23 71.08 68.57 55.65 55.61 55.60 55.57 52.38 49.97 46.42 36.63 36.60 11.50 11.24
O
N
O
N
Me
OMe
MeO
ent-1f
13C NMR
210 200 190 180 170 160 150 140 130 120 110 100 90 80 70 60 50 40 30 20 10 ppm

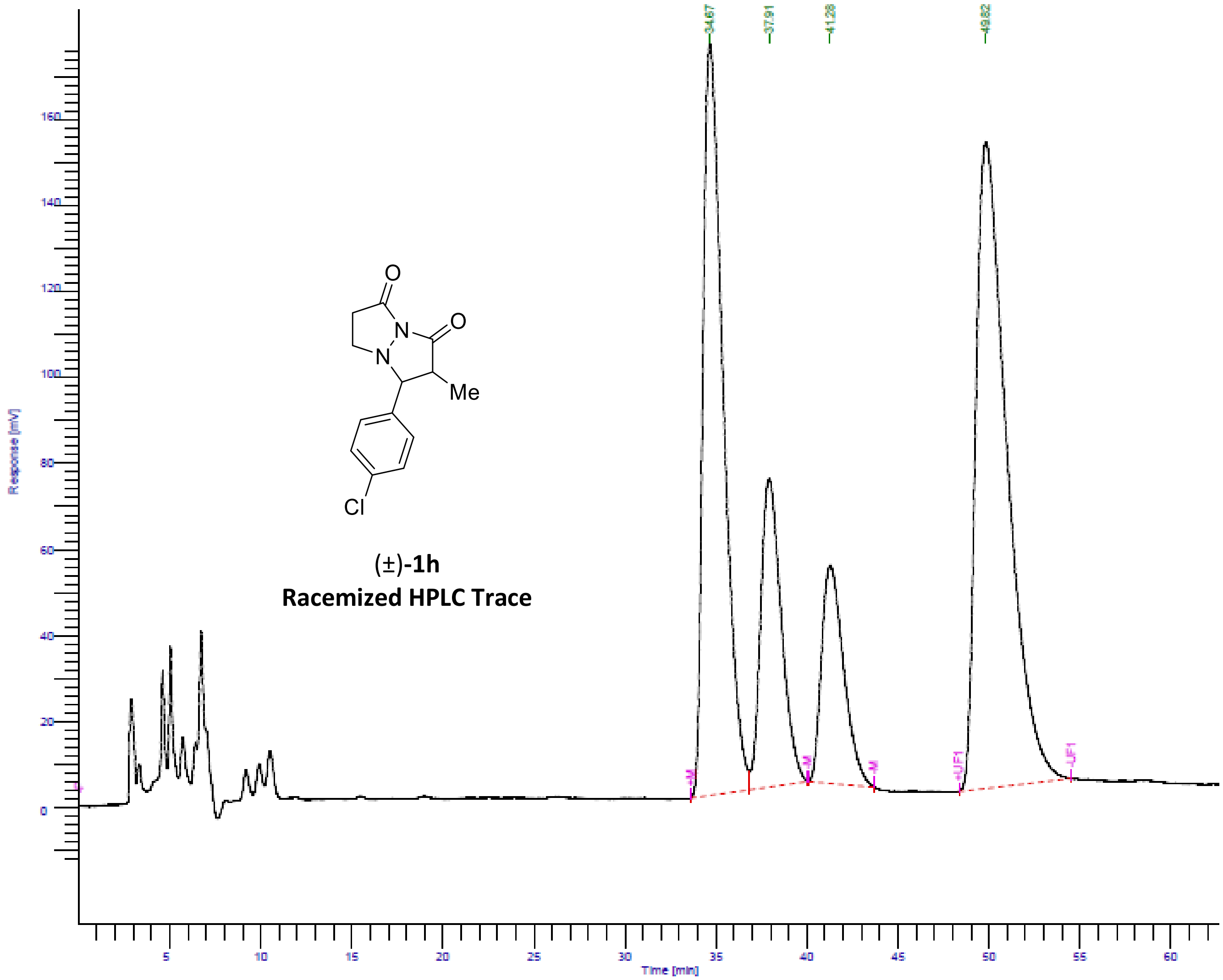

(±)-**1h**
Racemized HPLC Trace

Peak #	Time [min]	Area [µV·s]	Height [µV]	Area [%]	Norm. Area [%]	BL	Area/Height [s]
1	34.667	13504185.55	174935.81	32.66	32.66	*BV	77.1951
2	37.914	5662409.97	71693.00	13.70	13.70	*VB	78.9814
3	41.279	4309752.41	50780.53	10.42	10.42	*BB	84.8702
4	49.823	17868268.34	150452.98	43.22	43.22	*MM	118.7631
		41344616.28	447862.32	100.00	100.00		

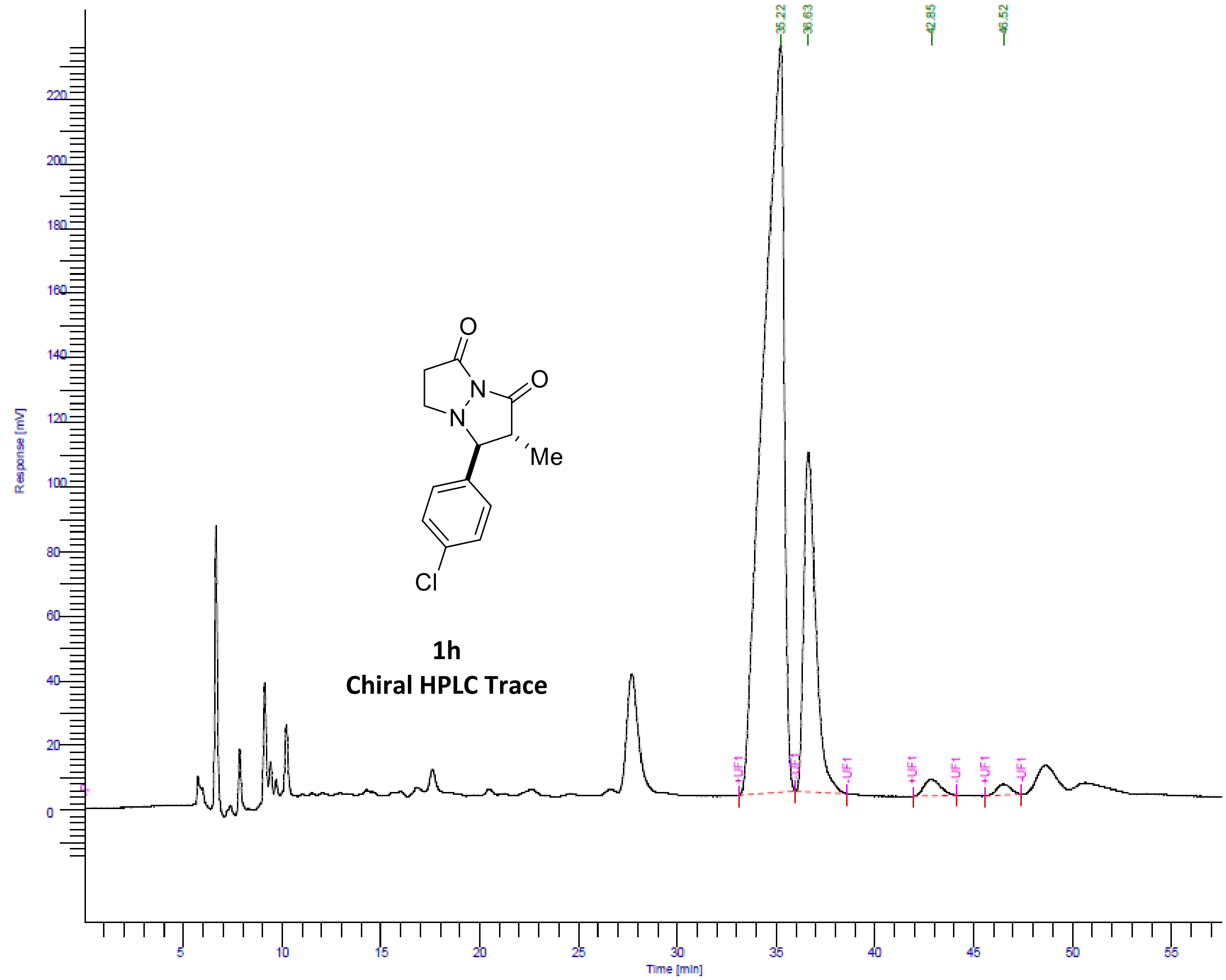

1h
Chiral HPLC Trace

Peak #	Time [min]	Area [µV·s]	Height [µV]	Area [%]	Norm. Area [%]	BL	Area/Height [s]
1	34.137	26070695.49	291217.23	74.08	74.08	*MM	89.5232
2	38.302	2134.32	262.02	0.01	0.01	*MM	8.1455
3	40.927	8987849.42	97052.15	25.54	25.54	*MM	92.6085
4	51.157	129879.63	1412.80	0.37	0.37	*MM	91.9305
		35190558.87	389944.20	100.00	100.00		

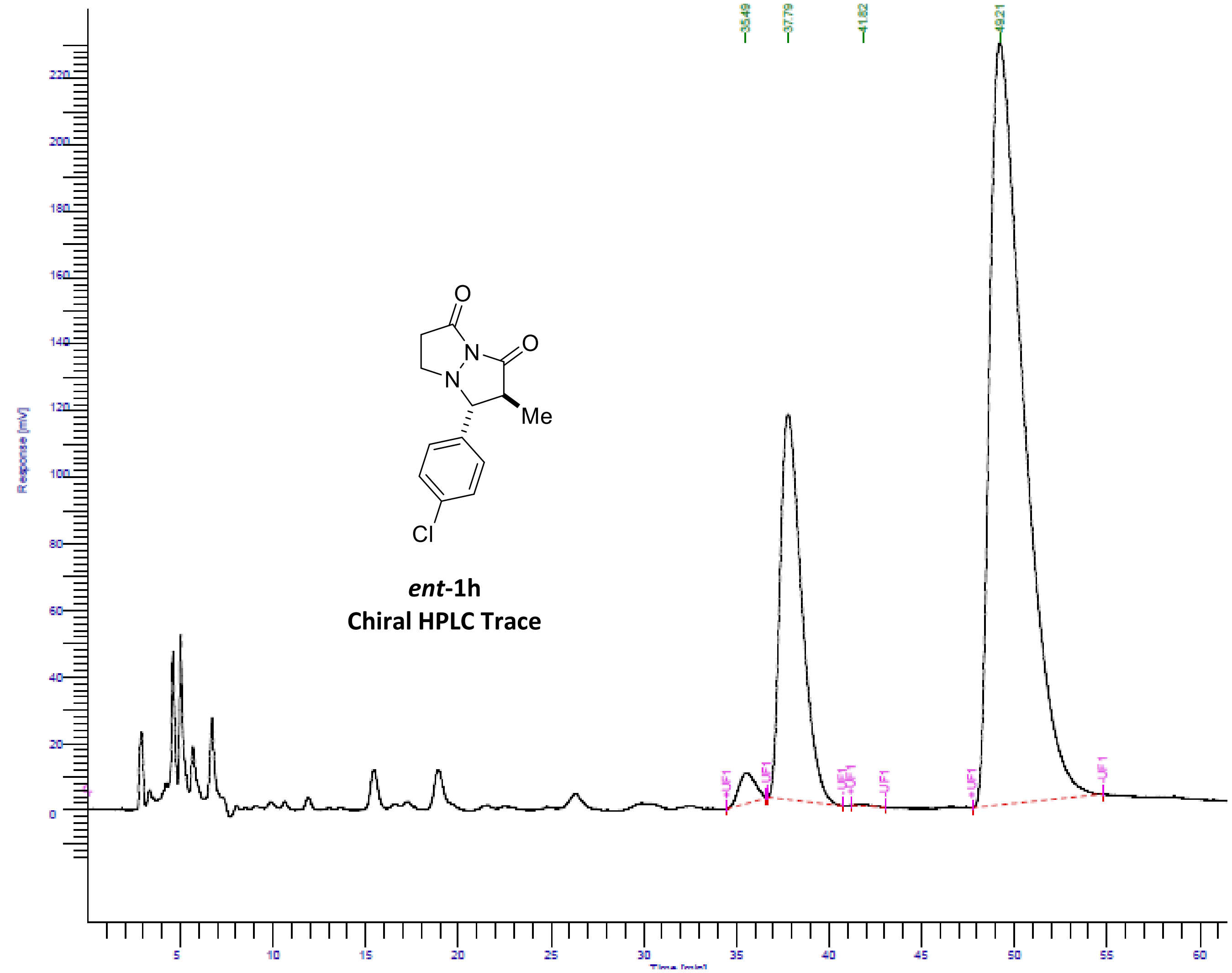

***ent*-1h**
Chiral HPLC Trace

Peak #	Time [min]	Area [µV·s]	Height [µV]	Area [%]	Norm. Area [%]	BL	Area/Height [s]
1	35.486	572680.92	9260.45	1.45	1.45	*MM	61.8416
2	37.789	9327879.79	115742.57	23.65	23.65	*MM	80.5916
3	41.822	22972.09	546.80	0.06	0.06	*MM	42.0119
4	49.214	29523282.19	229032.13	74.84	74.84	*MM	128.9045
		39446814.99	354581.96	100.00	100.00		

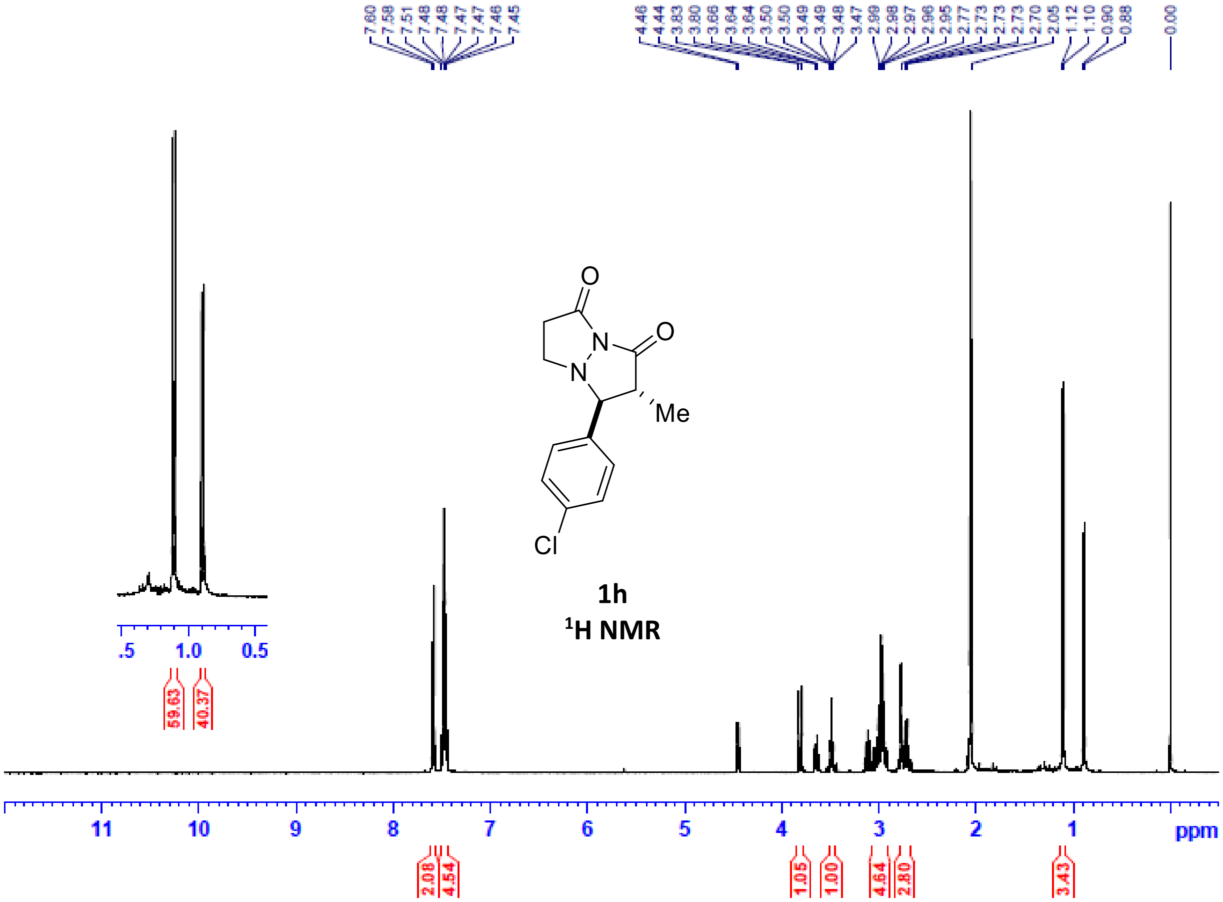
7.60
7.58
7.51
7.48
7.48
7.47
7.47
7.46
7.45
4.46
4.44
3.83
3.80
3.66
3.64
3.64
3.50
3.49
3.49
3.48
3.47
2.99
2.98
2.97
2.96
2.95
2.77
2.73
2.73
2.73
2.70
2.05
1.12
1.10
0.90
0.88
0.00
O
N
N
O
Me
Cl
1h
1H NMR
.5
1.0
0.5
59.63
40.37
11
10
9
8
7
6
5
4
3
2
1
ppm
2.08
4.54
1.05
1.00
4.64
2.80
3.43

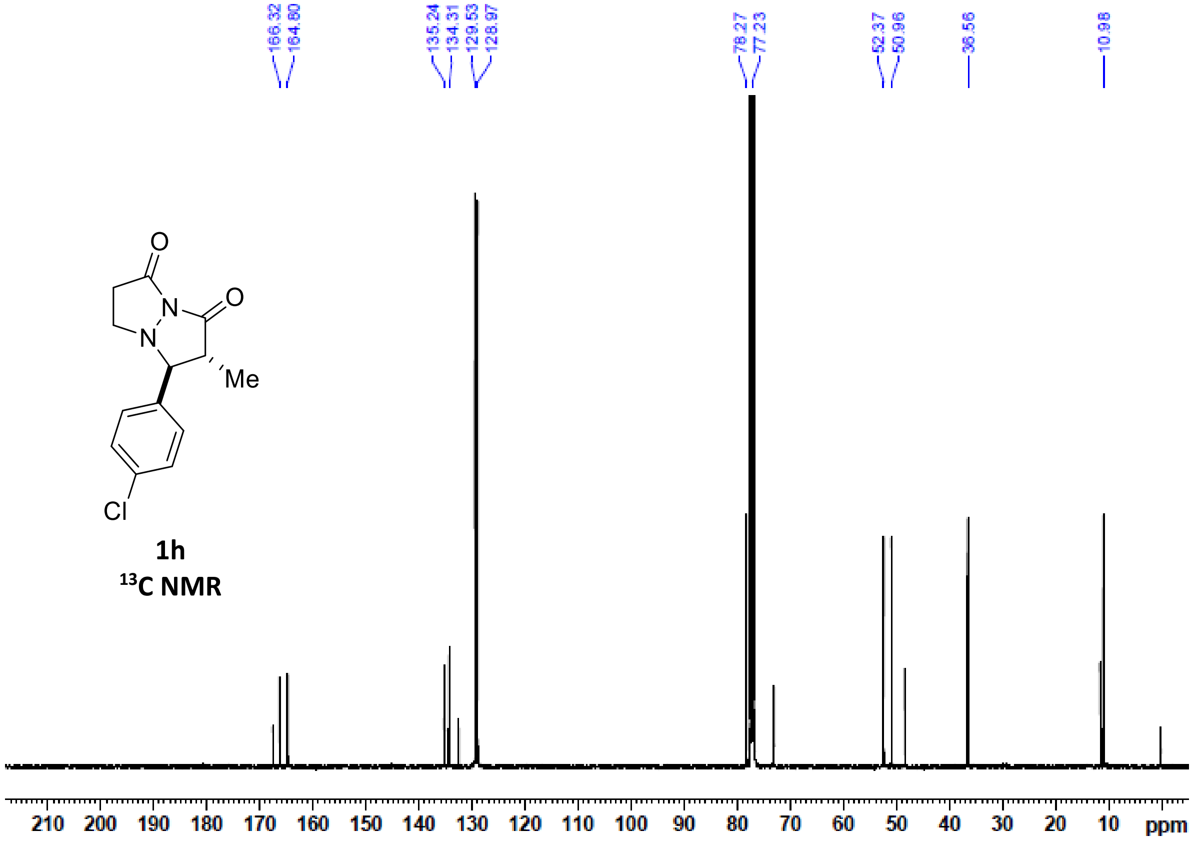
166.32
164.80
135.24
134.31
129.53
128.97
78.27
77.23
52.37
50.96
36.56
10.98
O
N
N
O
Me
Cl
1h
13C NMR
210
200
190
180
170
160
150
140
130
120
110
100
90
80
70
60
50
40
30
20
10
ppm

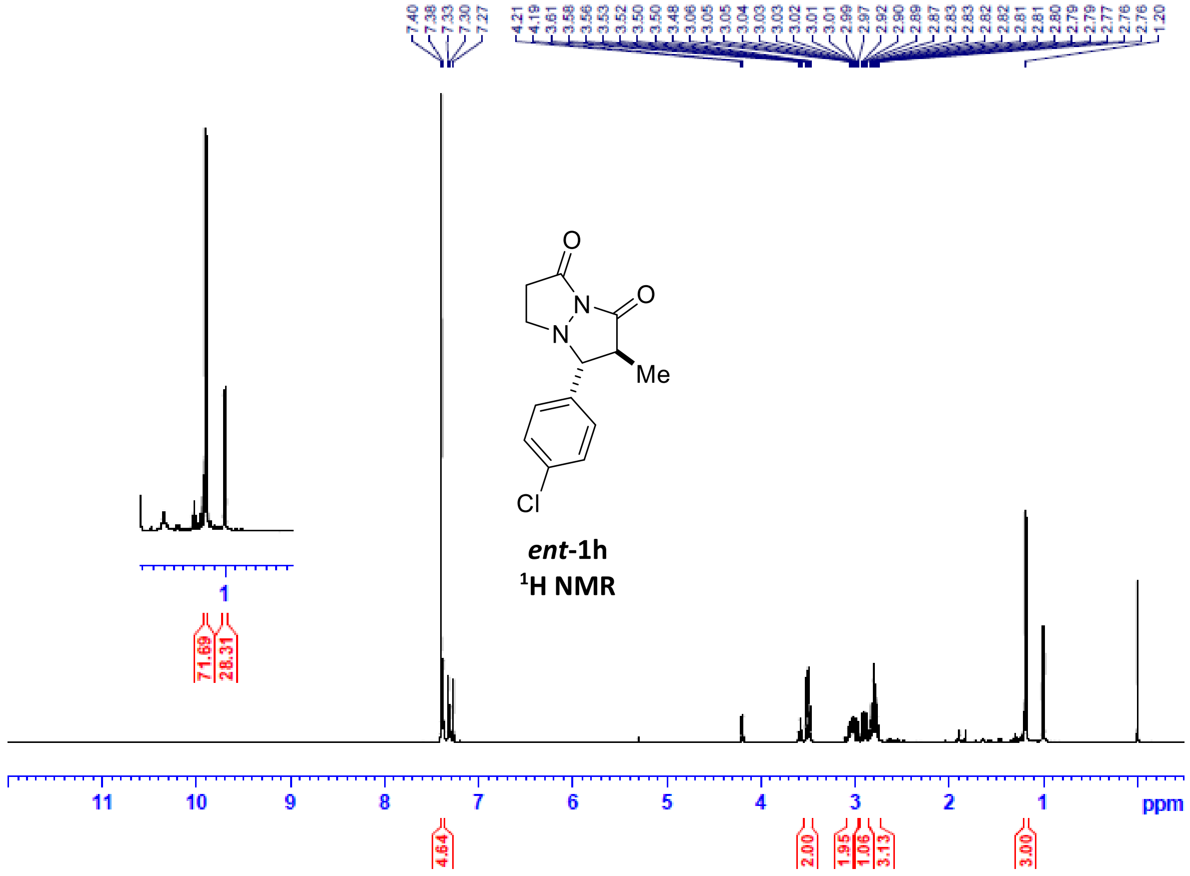

ent-1h
^{1}H NMR

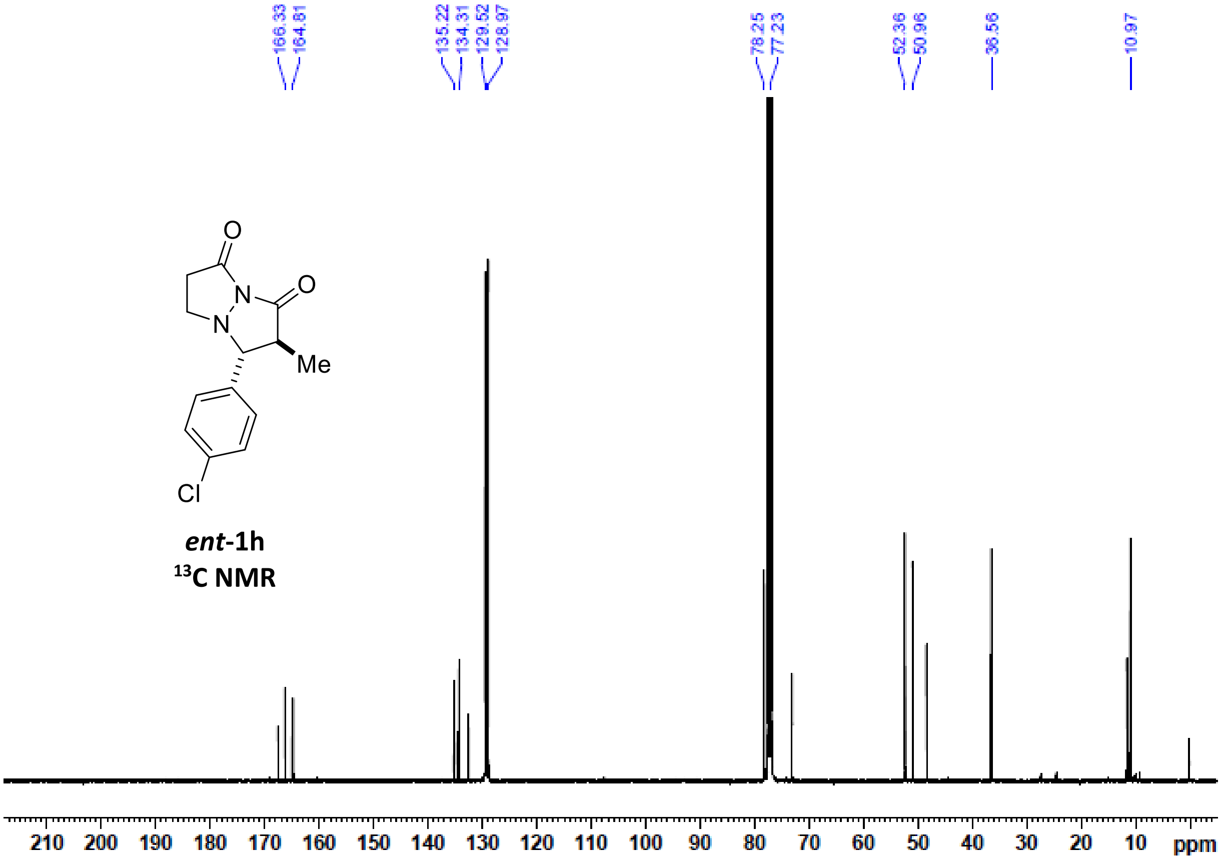

ent-1h
^{13}C NMR

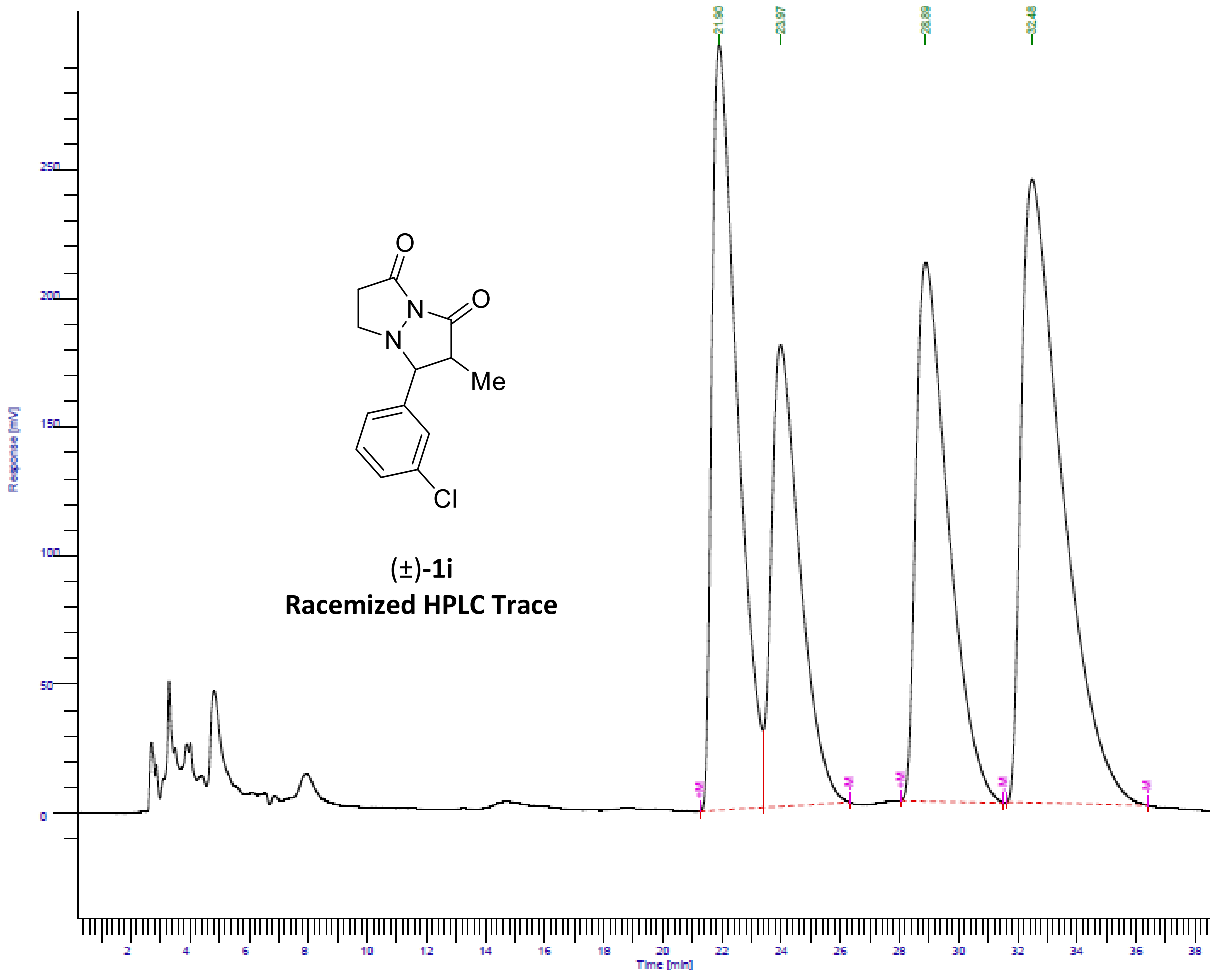

Peak #	Time [min]	Area [µV·s]	Height [µV]	Area [%]	Norm. Area [%]	BL	Area/Height [s]
1	21.898	17842920.18	297540.58	25.84	25.84	*BV	59.9680
2	23.974	12032402.99	179486.08	17.42	17.42	*VB	67.0381
3	28.886	15886112.09	209400.82	23.00	23.00	*BB	75.8646
4	32.481	23295749.66	242229.05	33.73	33.73	*BB	96.1724

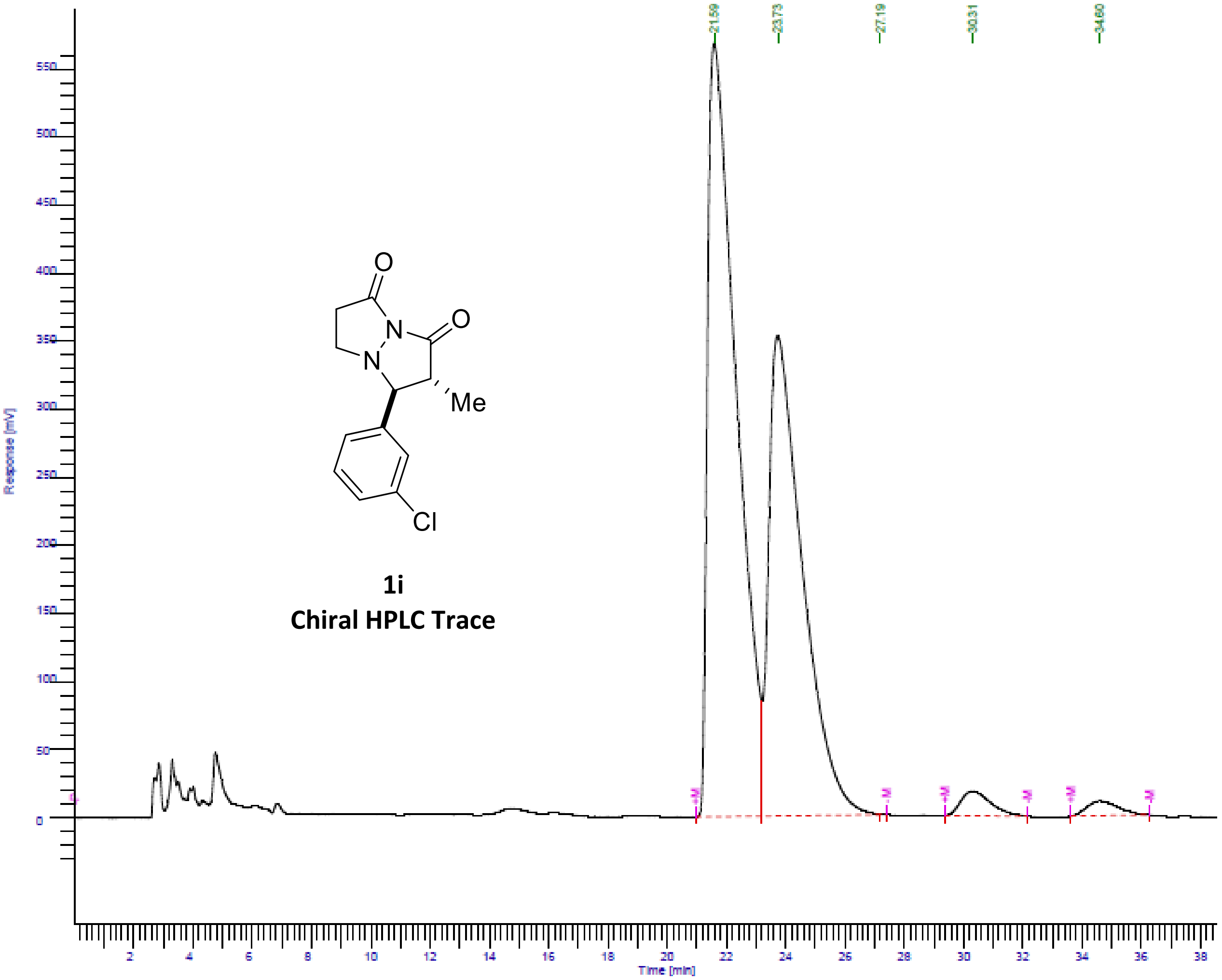

Peak #	Time [min]	Area [µV·s]	Height [µV]	Area [%]	Norm. Area [%]	BL	Area/Height [s]
1	21.586	38546684.96	568137.48	56.19	56.19	*BV	67.8475
2	23.727	27979903.65	352529.93	40.78	40.78	*VV	79.3689
3	27.194	2134.27	360.74	0.00	0.00	*VB	5.9164
4	30.310	1275763.89	18121.64	1.86	1.86	*BB	70.4000
5	34.598	799846.83	10750.51	1.17	1.17	*BB	74.4008

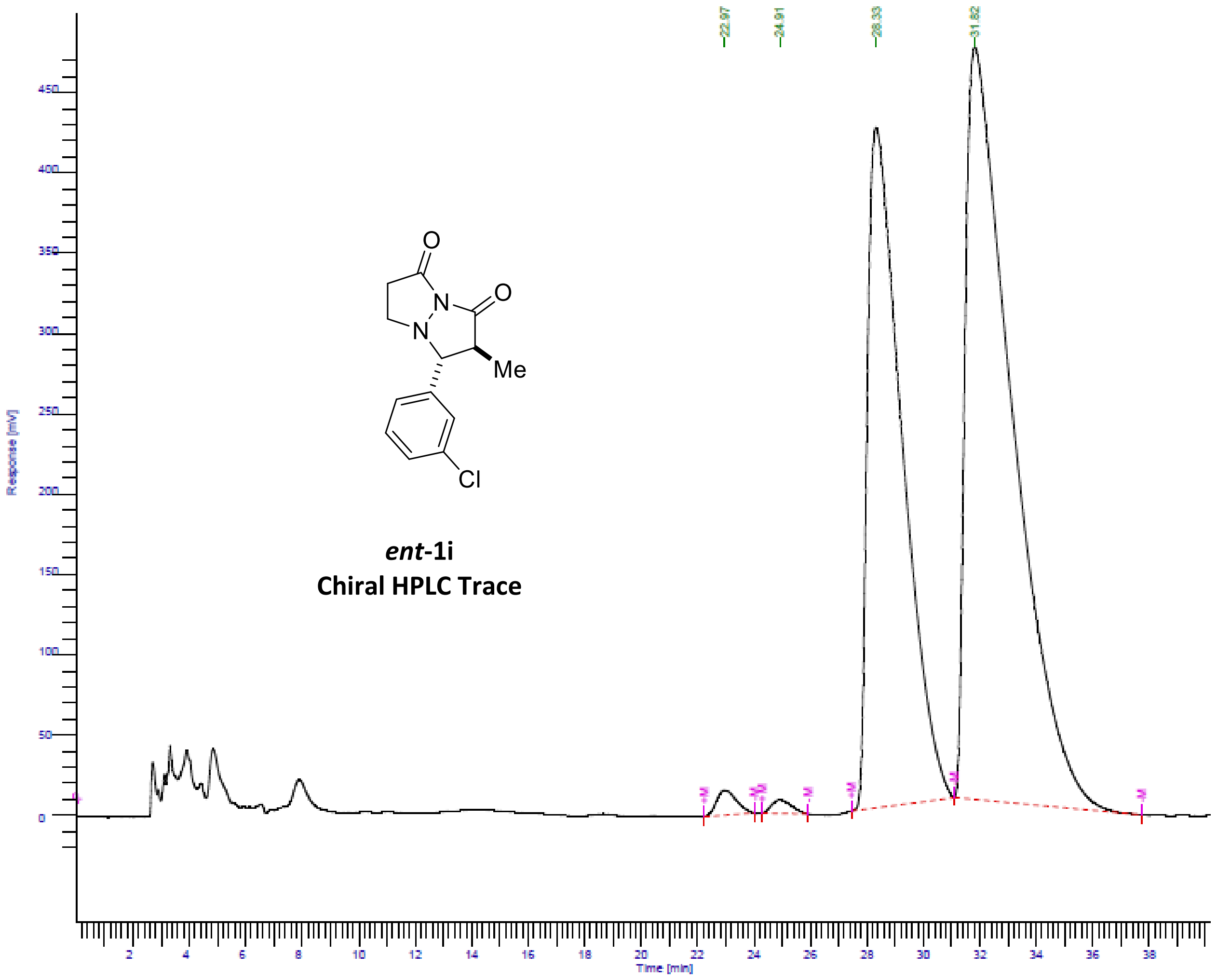

Peak #	Time [min]	Area [µV·s]	Height [µV]	Area [%]	Norm. Area [%]	BL	Area/Height [s]
1	22.969	760692.91	15541.07	0.83	0.83	*BB	48.9473
2	24.907	416462.03	8470.61	0.45	0.45	*BB	49.1655
3	28.327	36227192.55	423396.27	39.53	39.53	*BB	85.5633
4	31.821	54230587.79	468156.18	59.18	59.18	*BB	115.8387

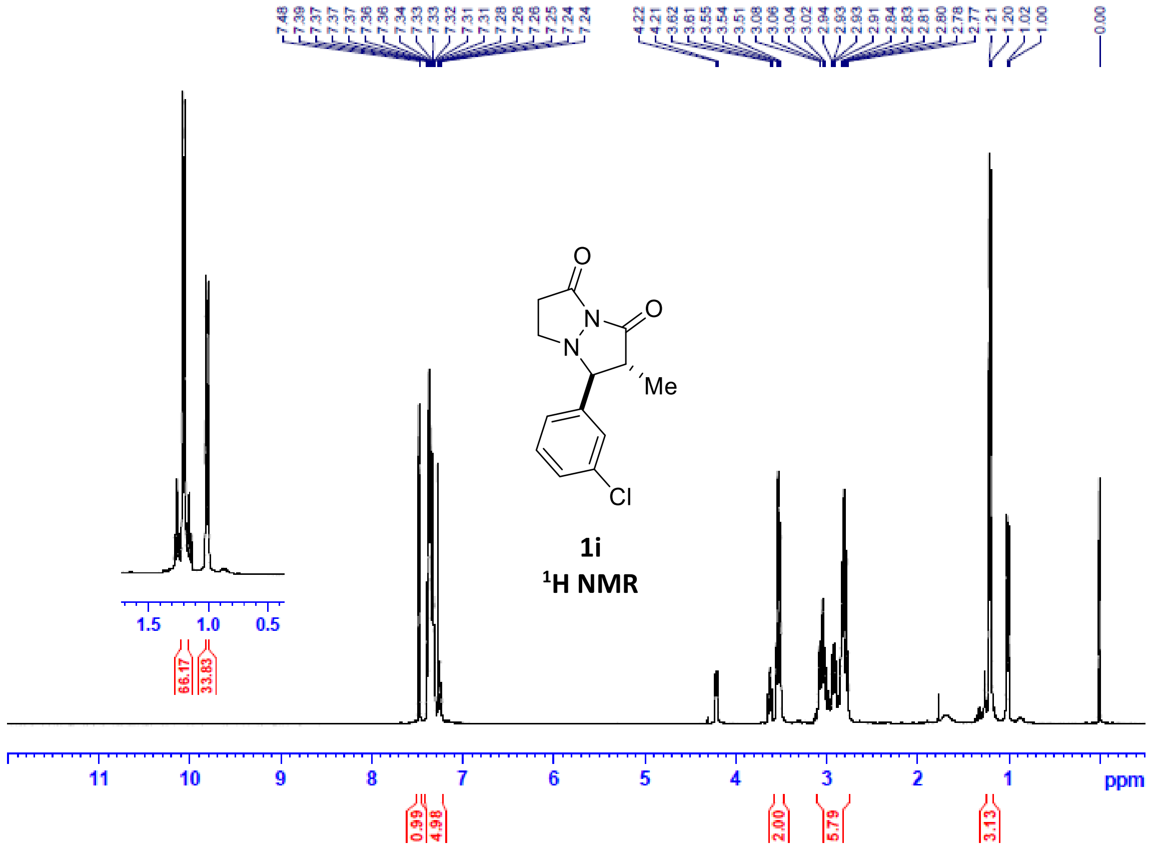
7.48 7.39 7.37 7.37 7.37 7.36 7.36 7.34 7.33 7.33 7.32 7.31 7.31 7.28 7.26 7.26 7.25 7.24 7.24
4.22 4.21 3.62 3.61 3.55 3.54 3.51 3.08 3.06 3.04 3.02 2.94 2.93 2.93 2.91 2.84 2.83 2.81 2.80 2.78 2.77 1.21 1.20 1.02 1.00
0.00
O
N
N
O
Me
Cl
1i
1H NMR
1.5 1.0 0.5
66.17 33.83
11 10 9 8 7 6 5 4 3 2 1 ppm
0.99 4.98 2.00 5.79 3.13

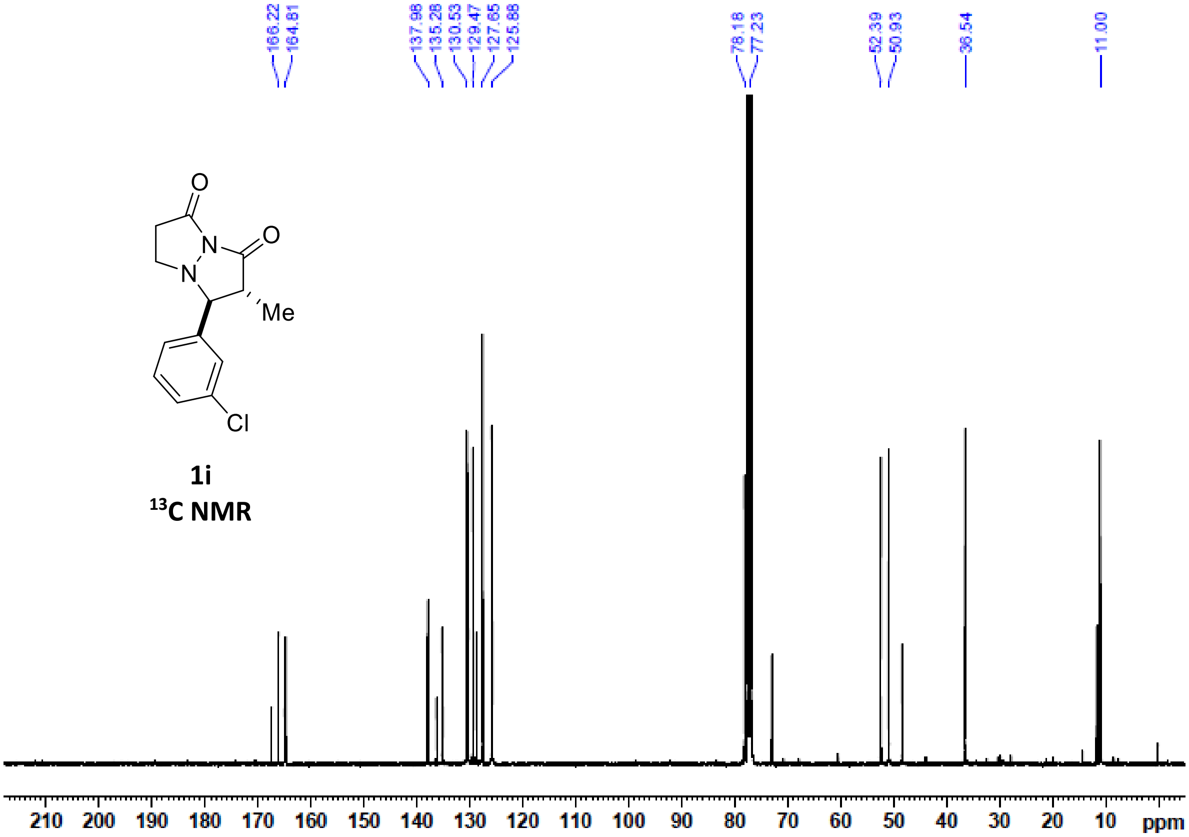
166.22 164.81
137.98 135.28 130.53 129.47 127.65 125.88
78.18 77.23
52.39 50.93
36.54
11.00
O
N
N
O
Me
Cl
1i
13C NMR
210 200 190 180 170 160 150 140 130 120 110 100 90 80 70 60 50 40 30 20 10 ppm

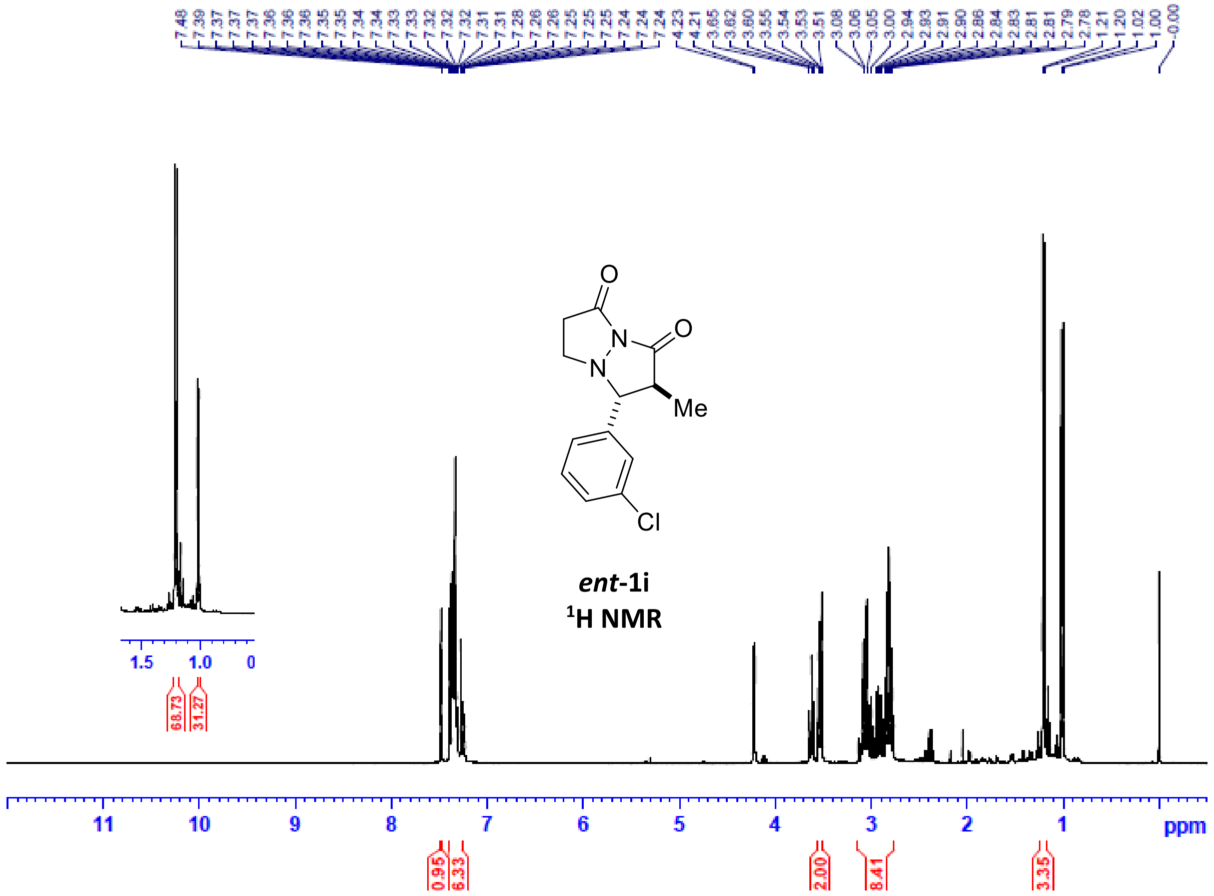
7.48 7.39 7.37 7.37 7.37 7.36 7.36 7.36 7.35 7.35 7.34 7.34 7.33 7.33 7.32 7.32 7.32 7.31 7.31 7.28 7.26 7.26 7.25 7.25 7.25 7.24 7.24 7.24 4.23 4.21 3.65 3.62 3.60 3.55 3.54 3.53 3.51 3.08 3.06 3.05 3.00 2.94 2.93 2.91 2.90 2.86 2.84 2.83 2.81 2.81 2.79 2.78 1.21 1.20 1.02 1.00 -0.00
O
N
N
O
Me
Cl
ent-1i
1H NMR
1.5
1.0
0
68.73
31.27
11
10
9
8
7
6
5
4
3
2
1
ppm
0.95
6.33
2.00
8.41
3.35

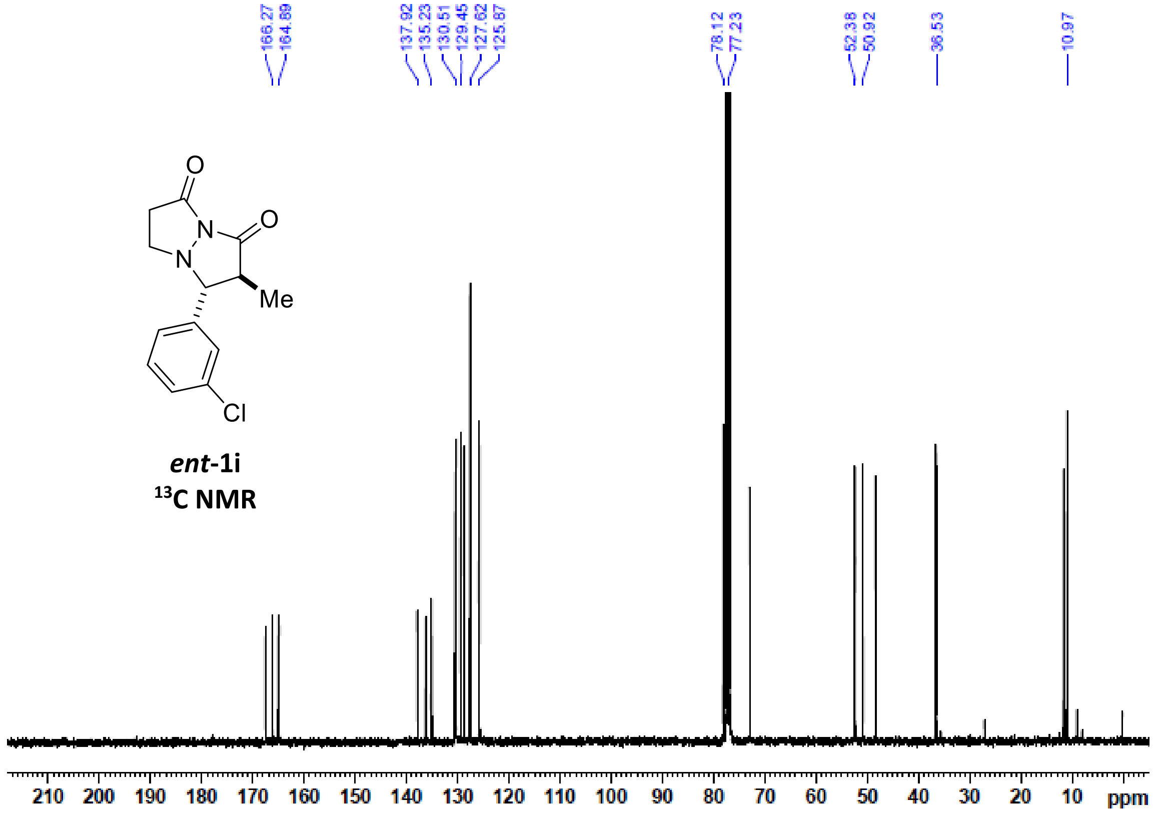
166.27 164.89 137.92 135.23 130.51 129.45 127.62 125.87 78.12 77.23 52.38 50.92 36.53 10.97
O
N
N
O
Me
Cl
ent-1i
13C NMR
210 200 190 180 170 160 150 140 130 120 110 100 90 80 70 60 50 40 30 20 10 ppm

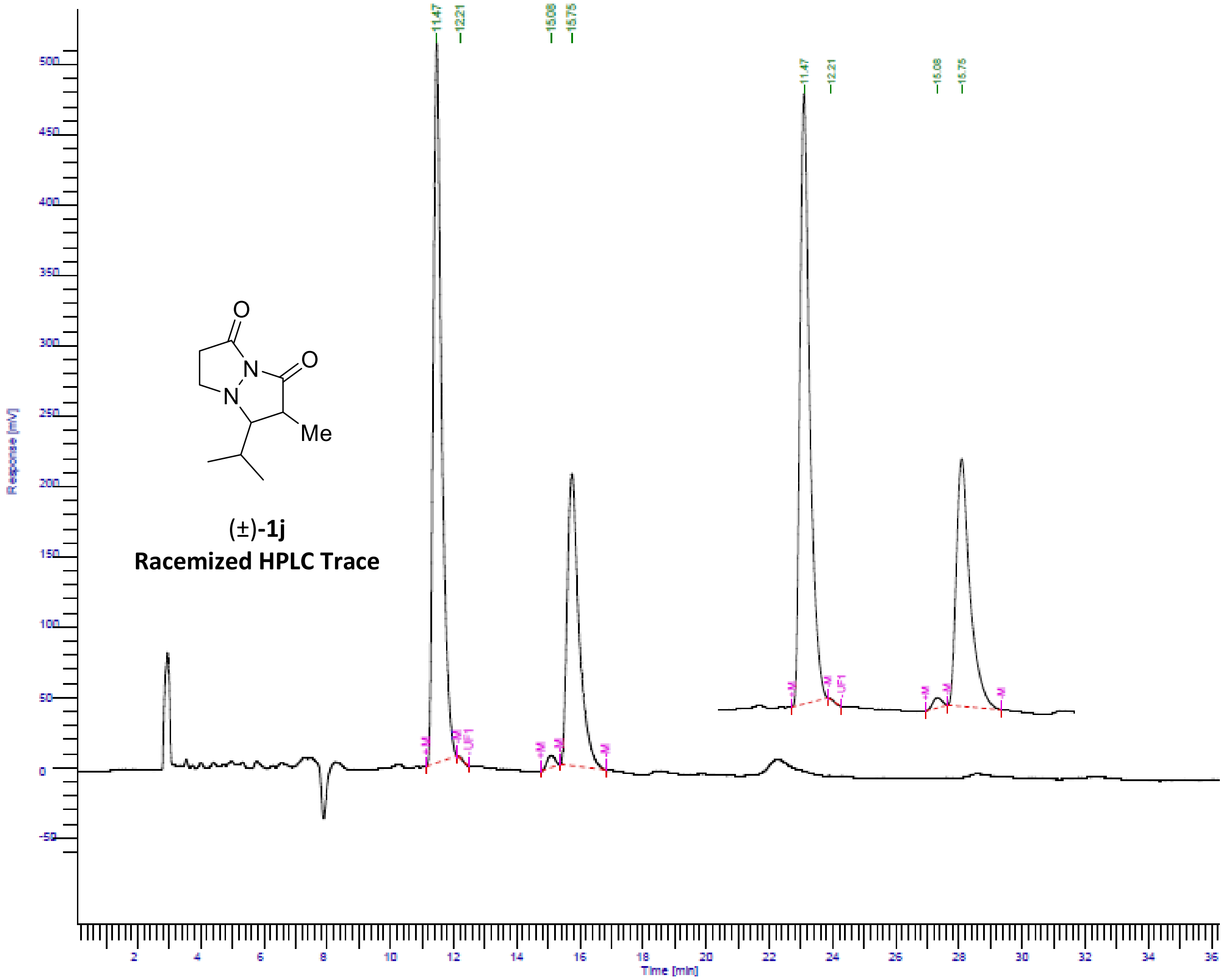

Peak #	Time [min]	Area [µV·s]	Height [µV]	Area [%]	Norm. Area [%]	BL	Area/Height [s]
1	11.467	9484881.47	511779.72	63.34	63.34	*BB	18.5331
2	12.210	4158.72	726.29	0.03	0.03	*MM	5.7260
3	15.084	142955.91	8420.27	0.95	0.95	*BB	16.9776
4	15.745	5343420.22	207388.89	35.68	35.68	*BB	25.7652

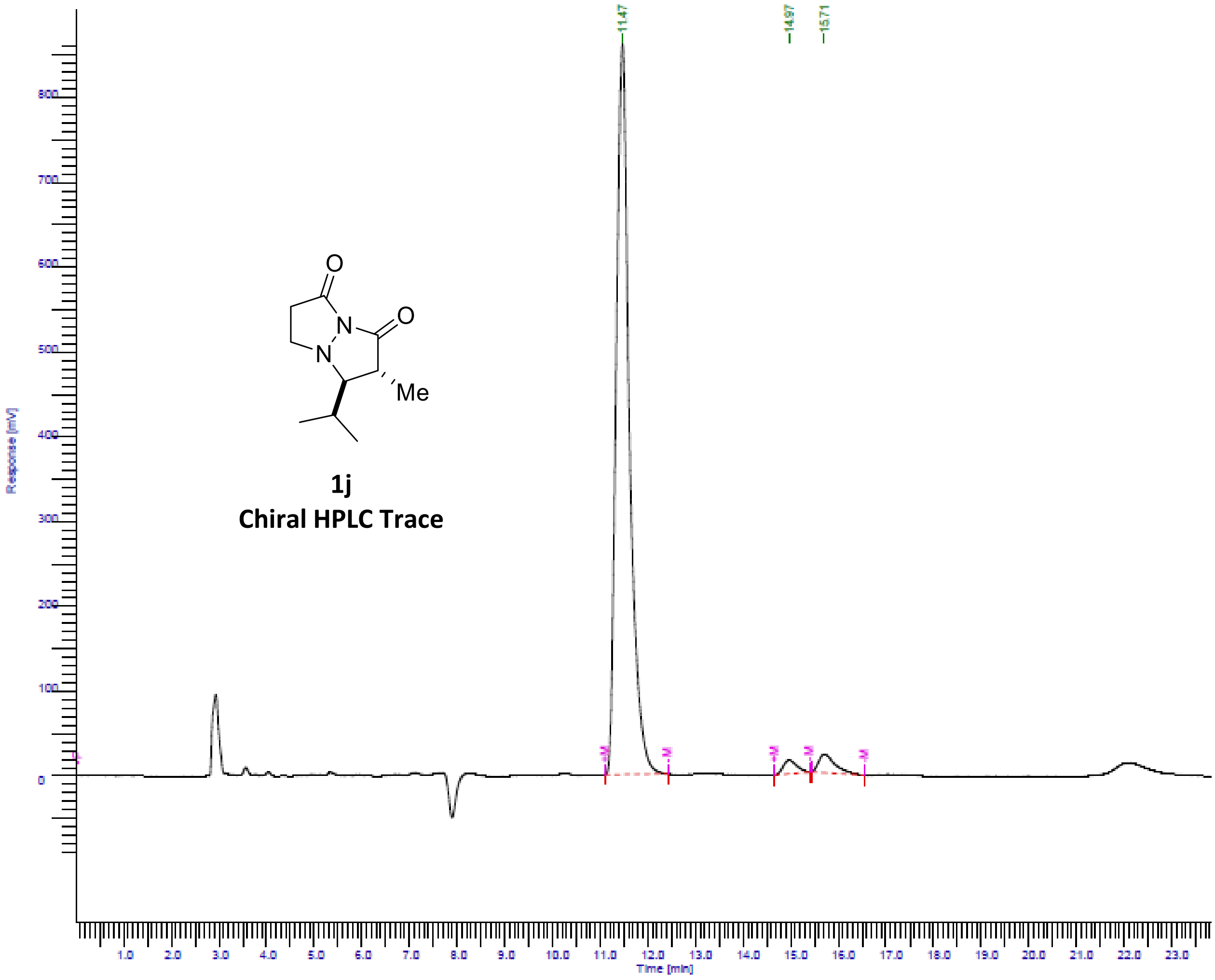

Peak #	Time [min]	Area [µV·s]	Height [µV]	Area [%]	Norm. Area [%]	BL	Area/Height [s]
1	11.467	16552384.75	861996.62	95.10	95.10	*BB	19.2024
2	14.966	324498.15	16252.52	1.86	1.86	*BB	19.9660
3	15.709	527855.18	21603.40	3.03	3.03	*BB	24.4339

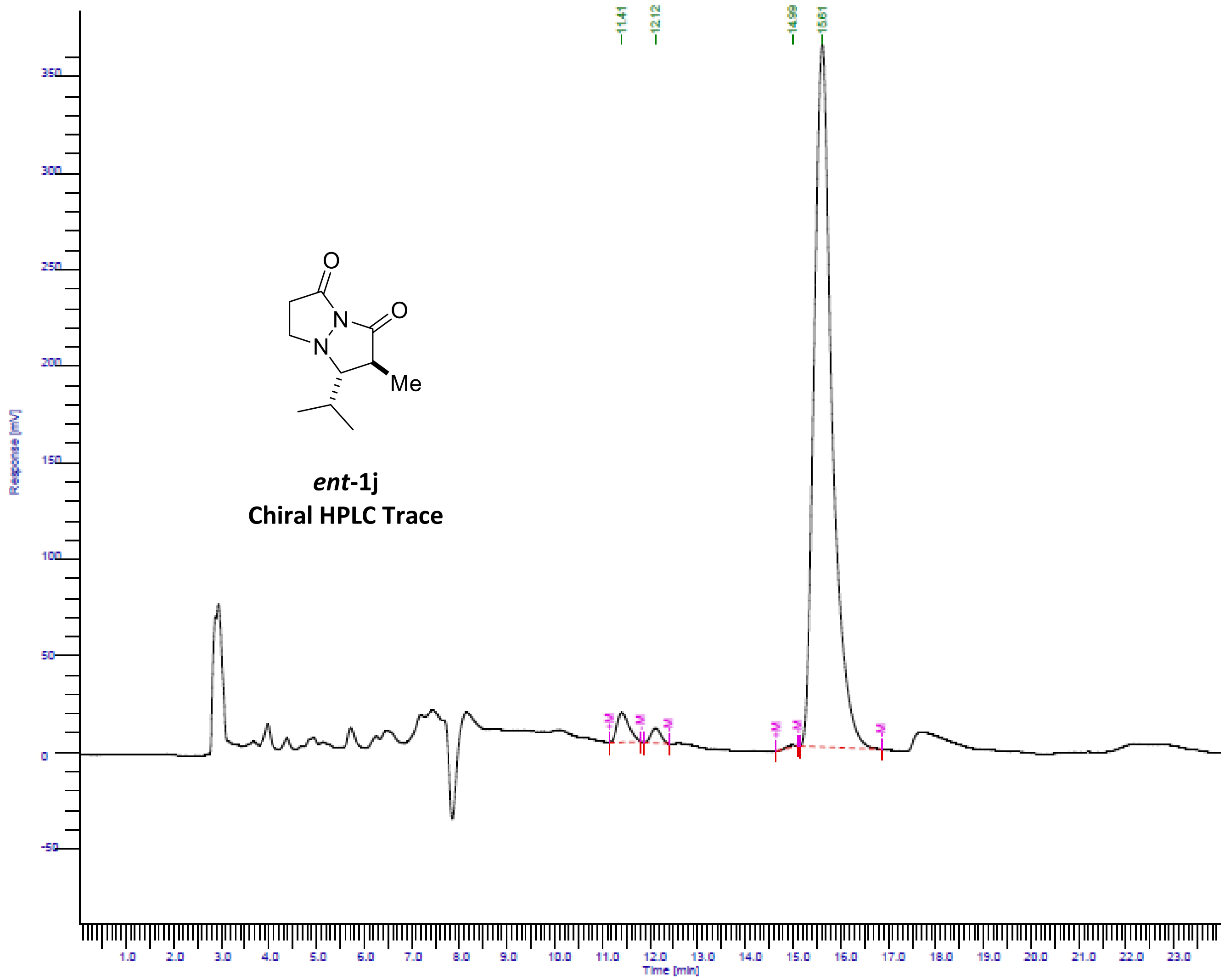

Peak #	Time [min]	Area [µV·s]	Height [µV]	Area [%]	Norm. Area [%]	BL	Area/Height [s]
1	11.406	267212.48	15470.05	2.68	2.68	*BB	17.2729
2	12.119	111176.34	7822.90	1.11	1.11	*BB	14.2117
3	14.990	15202.34	1230.59	0.15	0.15	*BB	12.3537
4	15.613	9594986.66	363797.79	96.06	96.06	*BB	26.3745

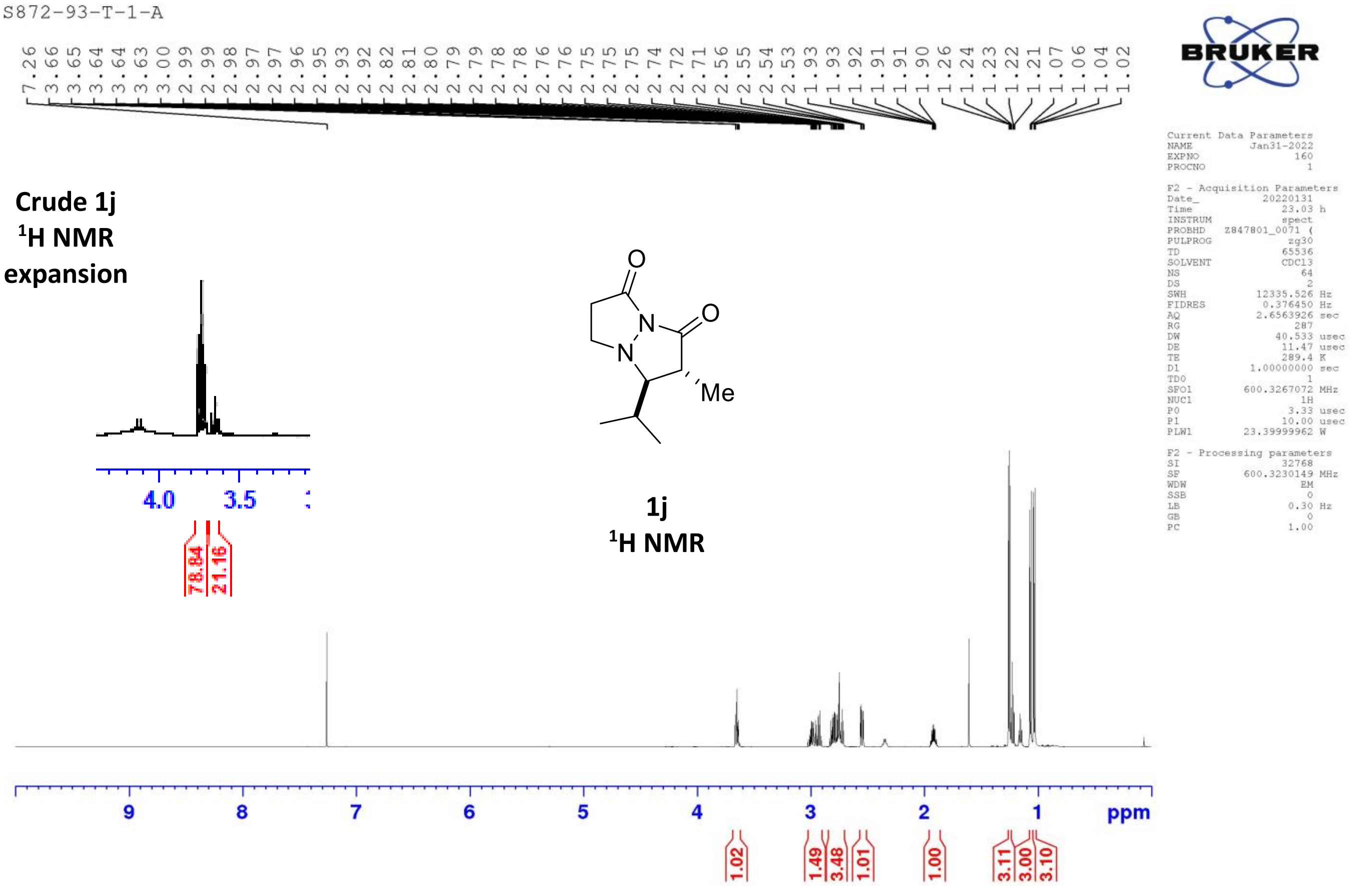
S872-93-T-1-A
Crude 1j
1H NMR
expansion
1j
1H NMR
Me
N
N
O
O
ppm
BRUKER
Current Data Parameters
NAME Jan31-2022
EXPNO 160
PROCNO 1
F2 - Acquisition Parameters
Date_ 20220131
Time 23.03 h
INSTRUM spect
PULPROG zg30
TD 65536
SOLVENT CDCl3
NS 64
DS 2
SWH 12335.526 Hz
FIDRES 0.376450 Hz
AQ 2.6563926 sec
RG 287
DW 40.533 usec
DE 11.47 usec
TE 289.4 K
D1 1.00000000 sec
TD0 1
SFO1 600.3267072 MHz
NUC1 1H
P0 3.33 usec
P1 10.00 usec
PLW1 23.39999962 W
F2 - Processing parameters
SI 32768
SF 600.3230149 MHz
WDW EM
SSB 0
LB 0.30 Hz
GB 0
PC 1.00

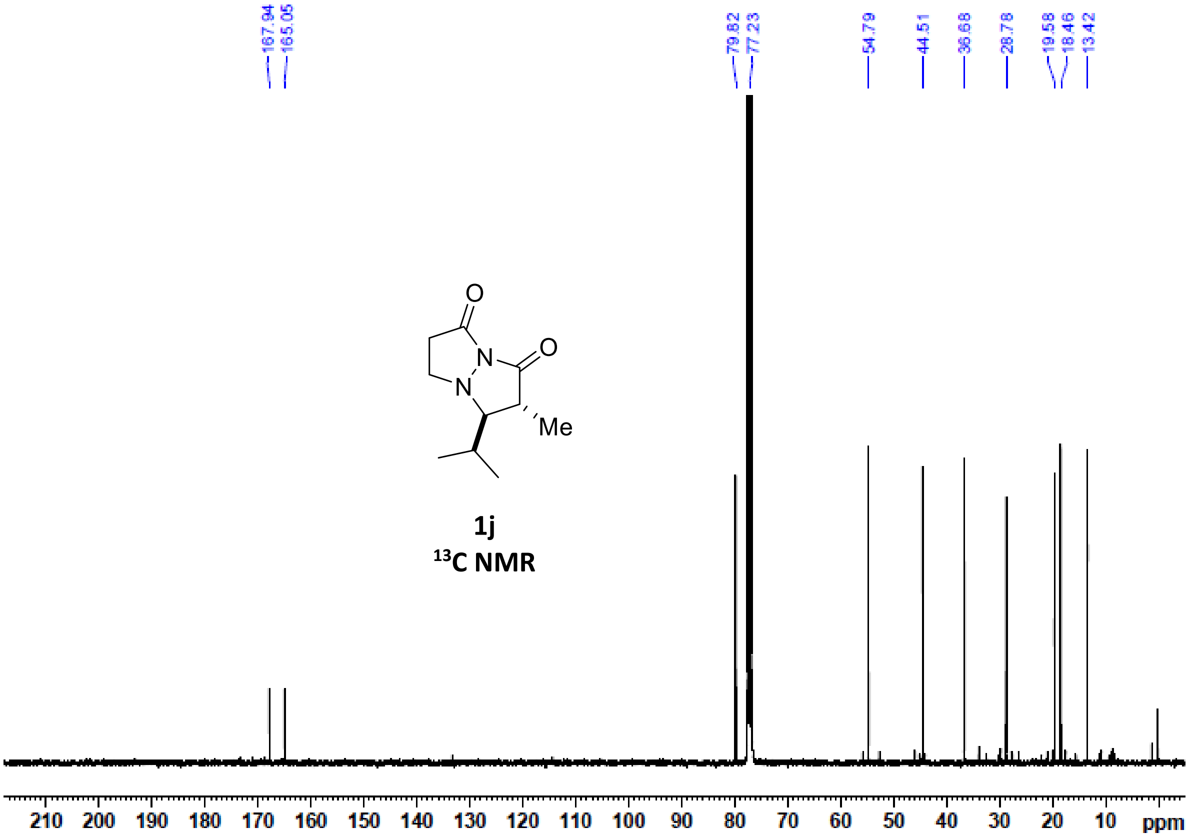
167.94
165.05
79.82
77.23
54.79
44.51
36.68
28.78
19.58
18.46
13.42
O
N
O
N
Me
1j
^{13}C NMR
210 200 190 180 170 160 150 140 130 120 110 100 90 80 70 60 50 40 30 20 10 ppm

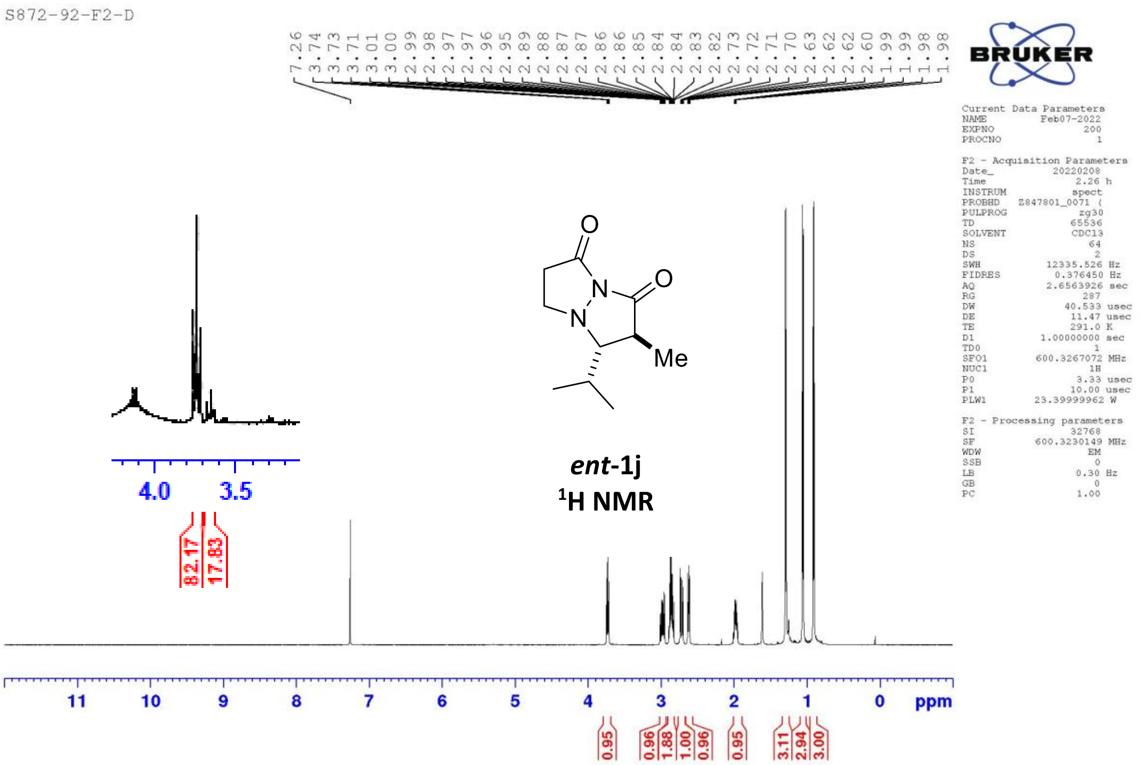

***ent*-1j**
^{1}H NMR

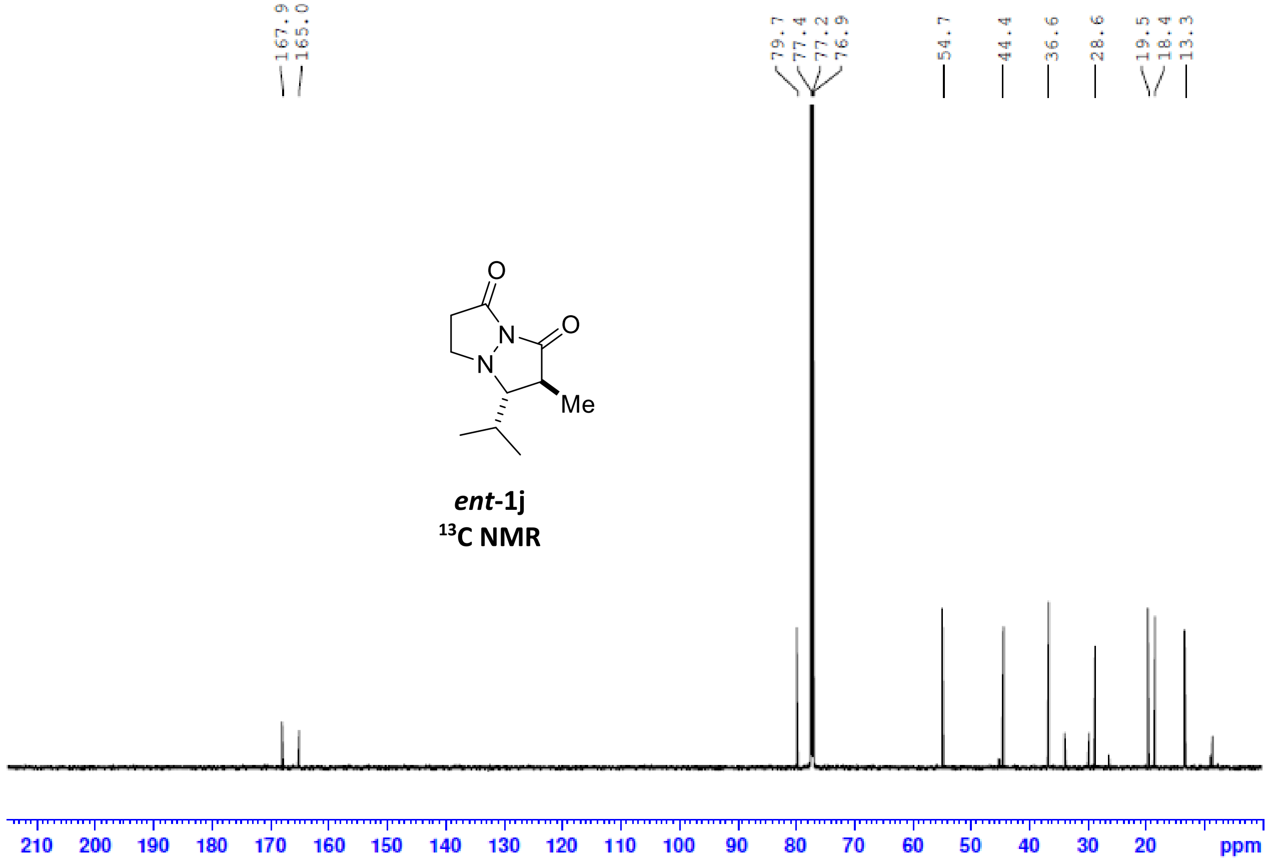

***ent*-1j**
^{13}C NMR

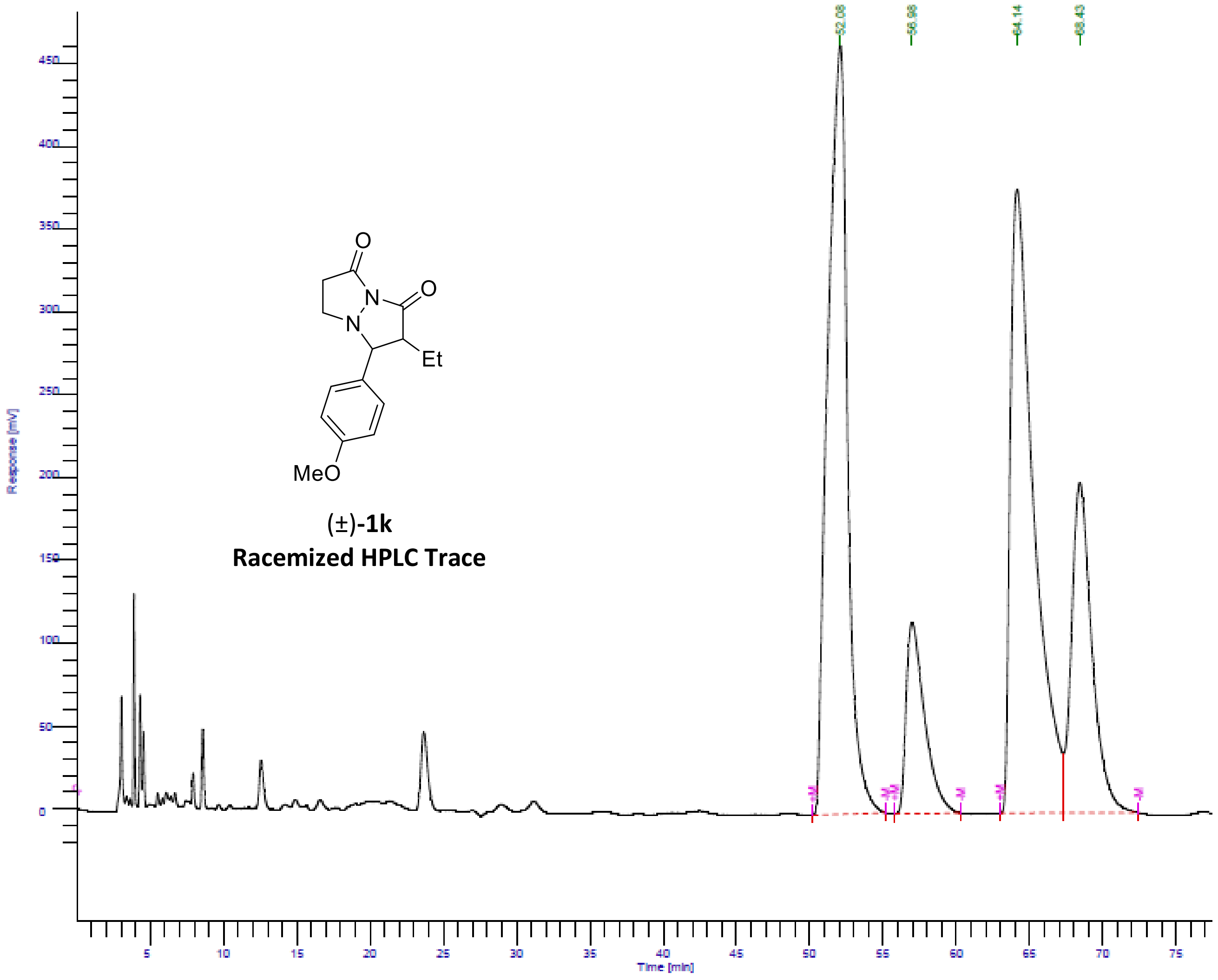

Peak #	Time [min]	Area [µV·s]	Height [µV]	Area [%]	Norm. Area [%]	BL	Area/Height [s]
1	52.076	43249334.36	464330.63	37.68	37.68	*BB	93.1434
2	56.982	10135540.56	115373.91	8.83	8.83	*BB	87.8495
3	64.144	41958322.24	376666.24	36.55	36.55	*BV	111.3939
4	68.435	19444393.18	199446.46	16.94	16.94	*VB	97.4918

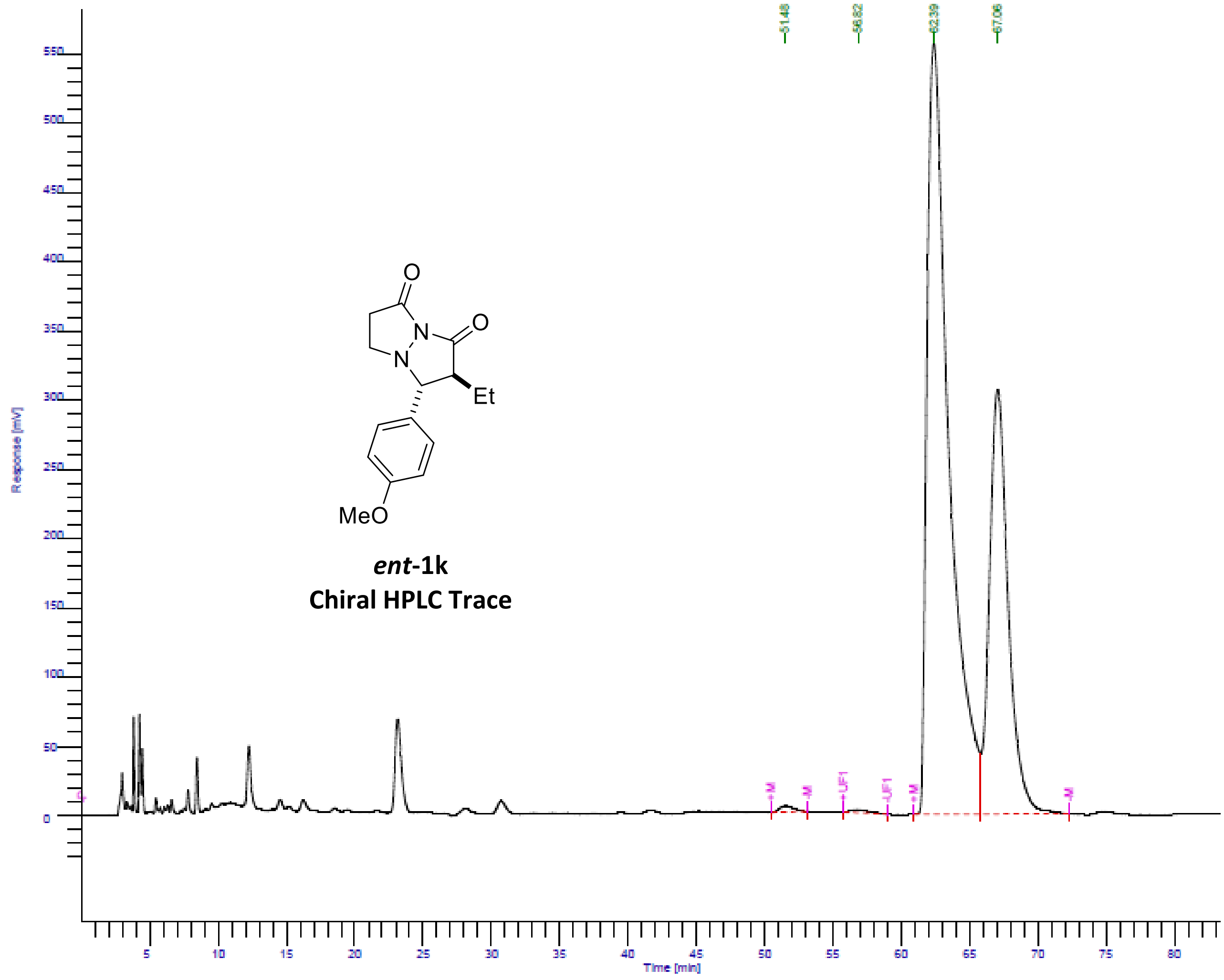

***ent*-1k**
Chiral HPLC Trace

Peak #	Time [min]	Area [µV·s]	Height [µV]	Area [%]	Norm. Area [%]	BL	Area/Height [s]
1	51.476	321483.64	4442.78	0.35	0.35	*BB	72.3609
2	56.819	222708.24	2268.66	0.24	0.24	*MM	98.1675
3	62.387	62521327.39	556983.76	67.63	67.63	*BV	112.2498
4	67.059	29386329.50	306843.97	31.79	31.79	*VB	95.7696

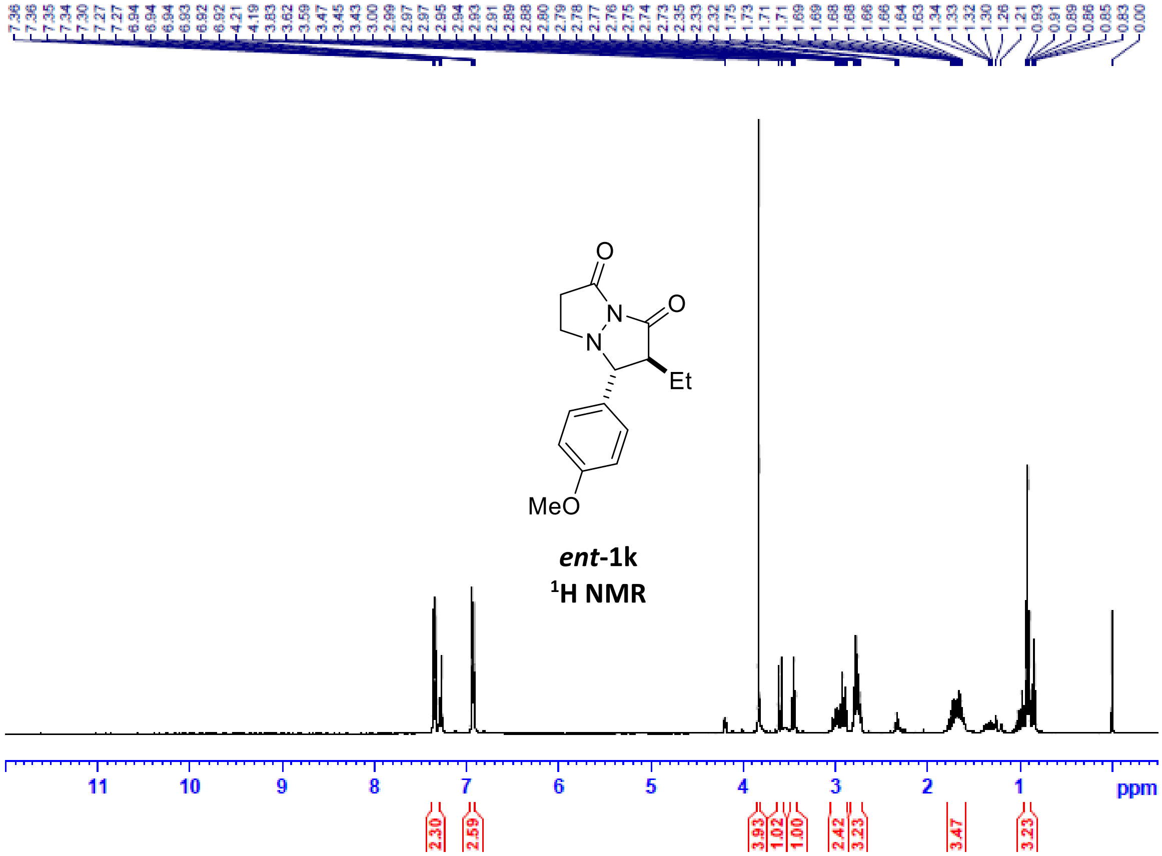

O
N
N
O
Et
MeO
ent-1k
1H NMR
ppm

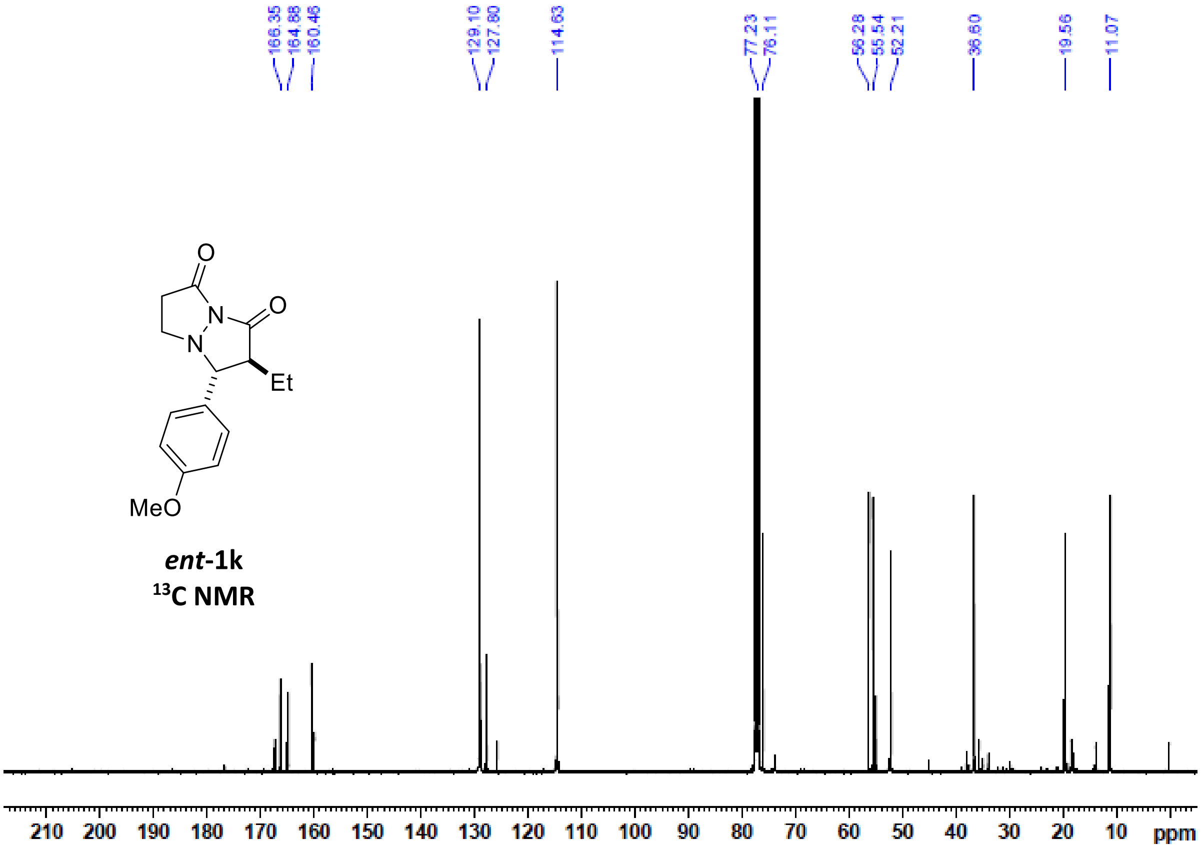

166.35
164.88
160.46
129.10
127.80
114.63
77.23
76.11
56.28
55.54
52.21
36.60
19.56
11.07
O
N
N
O
Et
MeO
ent-1k
13C NMR
ppm

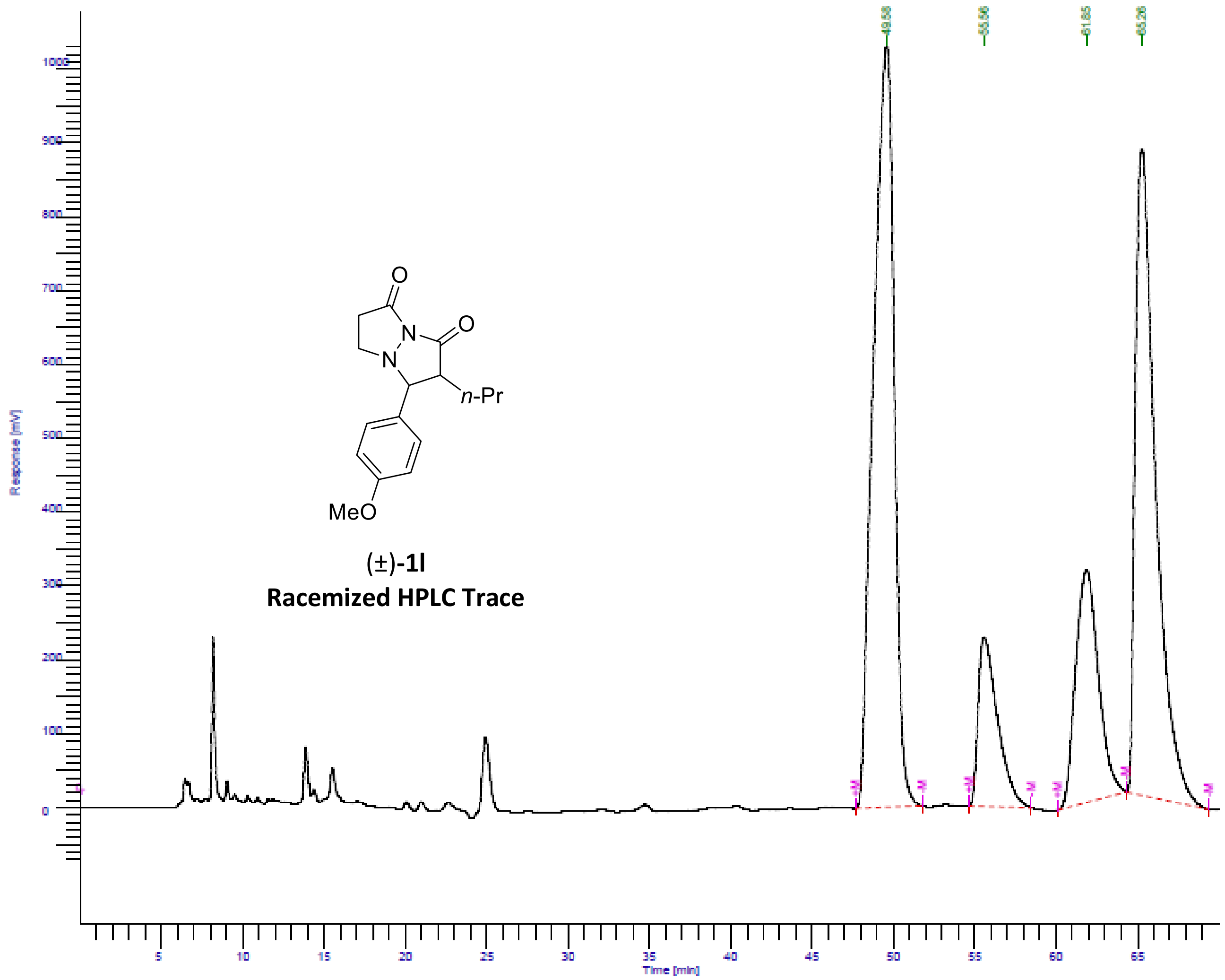

(±)-**1l**
Racemized HPLC Trace

Peak #	Time [min]	Area [µV·s]	Height [µV]	Area [%]	Norm. Area [%]	BL	Area/Height [s]
1	49.579	91077113.01	1.03e+06	41.98	41.98	*BB	88.3186
2	55.564	19063750.39	228840.35	8.79	8.79	*BB	83.3059
3	61.854	30804324.19	315324.11	14.20	14.20	*BB	97.6910
4	65.258	76016959.13	875505.67	35.04	35.04	*BB	86.8263

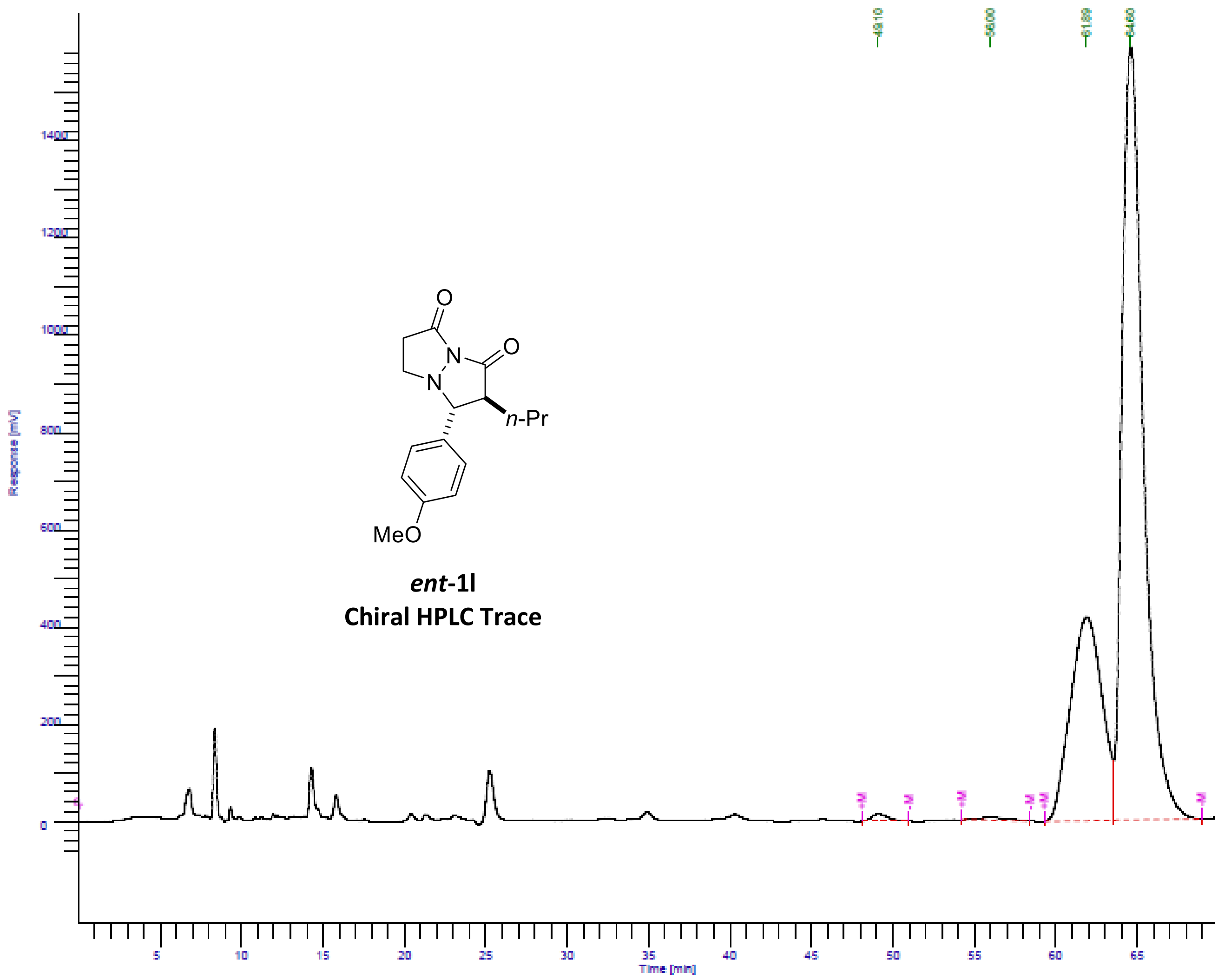

Peak #	Time [min]	Area [µV·s]	Height [µV]	Area [%]	Norm. Area [%]	BL	Area/Height [s]
1	49.098	1042821.92	14548.06	0.51	0.51	*BB	71.6812
2	55.999	759047.17	6737.88	0.37	0.37	*BB	112.6536
3	61.893	56525742.56	419157.99	27.74	27.74	*BV	134.8555
4	64.598	1.45e+08	1.59e+06	71.38	71.38	*VB	91.5193

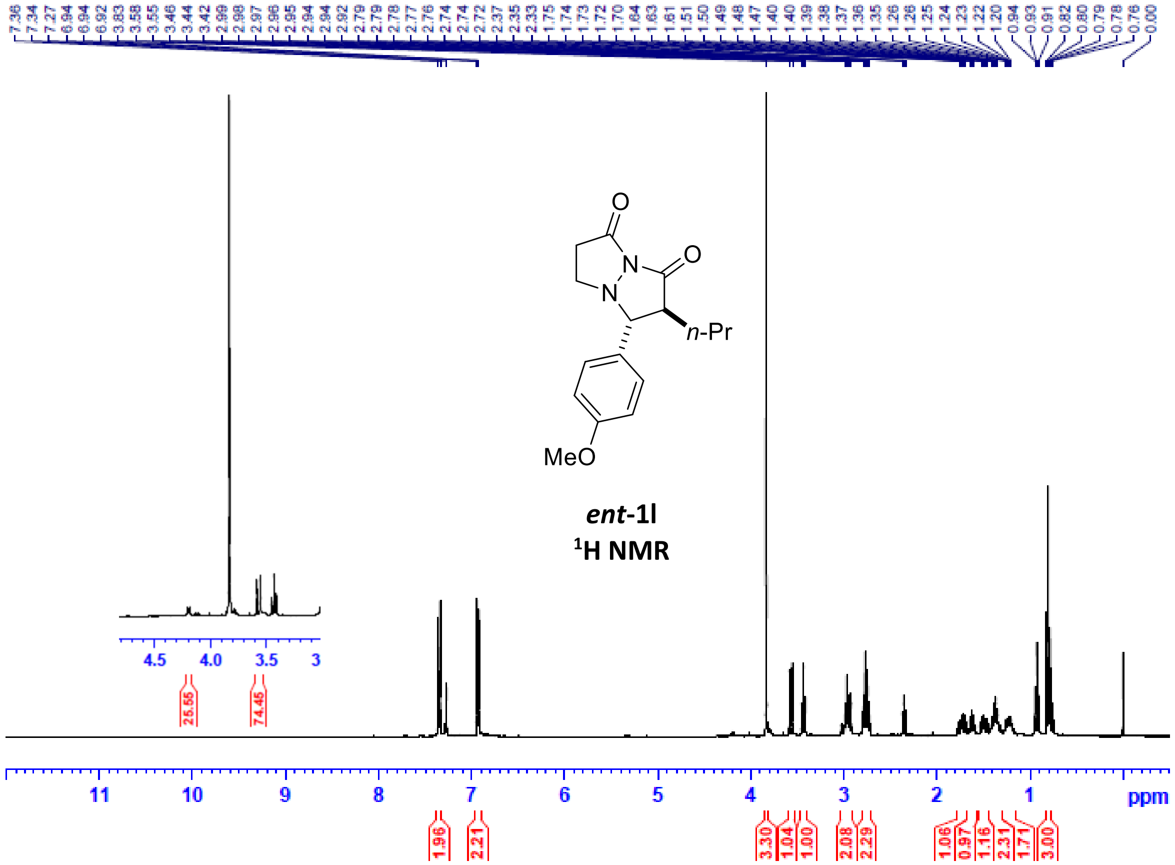
7.36 7.34 7.27 6.94 6.94 6.92 3.83 3.58 3.55 3.46 3.44 3.42 2.99 2.98 2.97 2.96 2.95 2.94 2.94 2.92 2.79 2.79 2.78 2.77 2.76 2.74 2.74 2.72 2.37 2.35 2.33 1.75 1.74 1.73 1.72 1.70 1.64 1.63 1.61 1.51 1.50 1.49 1.48 1.47 1.40 1.40 1.39 1.38 1.37 1.36 1.35 1.26 1.26 1.25 1.24 1.23 1.22 1.20 0.94 0.93 0.91 0.82 0.80 0.79 0.78 0.76 0.00
O
N
N
O
n-Pr
MeO
ent-1l
1H NMR
4.5 4.0 3.5 3
25.55 74.45
11 10 9 8 7 6 5 4 3 2 1 ppm
1.96 2.21 3.30 1.04 1.00 2.08 2.29 1.06 0.97 1.16 2.31 1.71 3.00

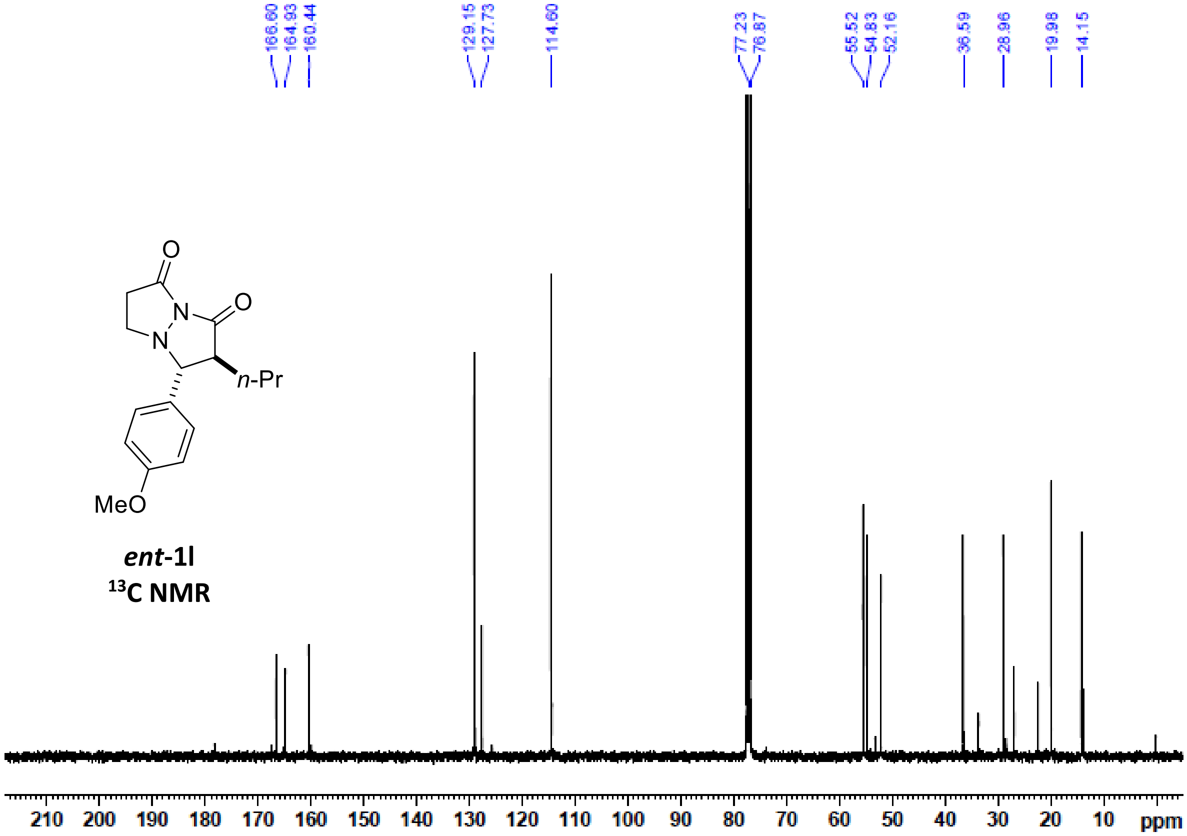
166.60 164.93 160.44 129.15 127.73 114.60 77.23 76.87 55.52 54.83 52.16 36.59 28.96 19.98 14.15
O
N
N
O
n-Pr
MeO
ent-1l
13C NMR
210 200 190 180 170 160 150 140 130 120 110 100 90 80 70 60 50 40 30 20 10 ppm

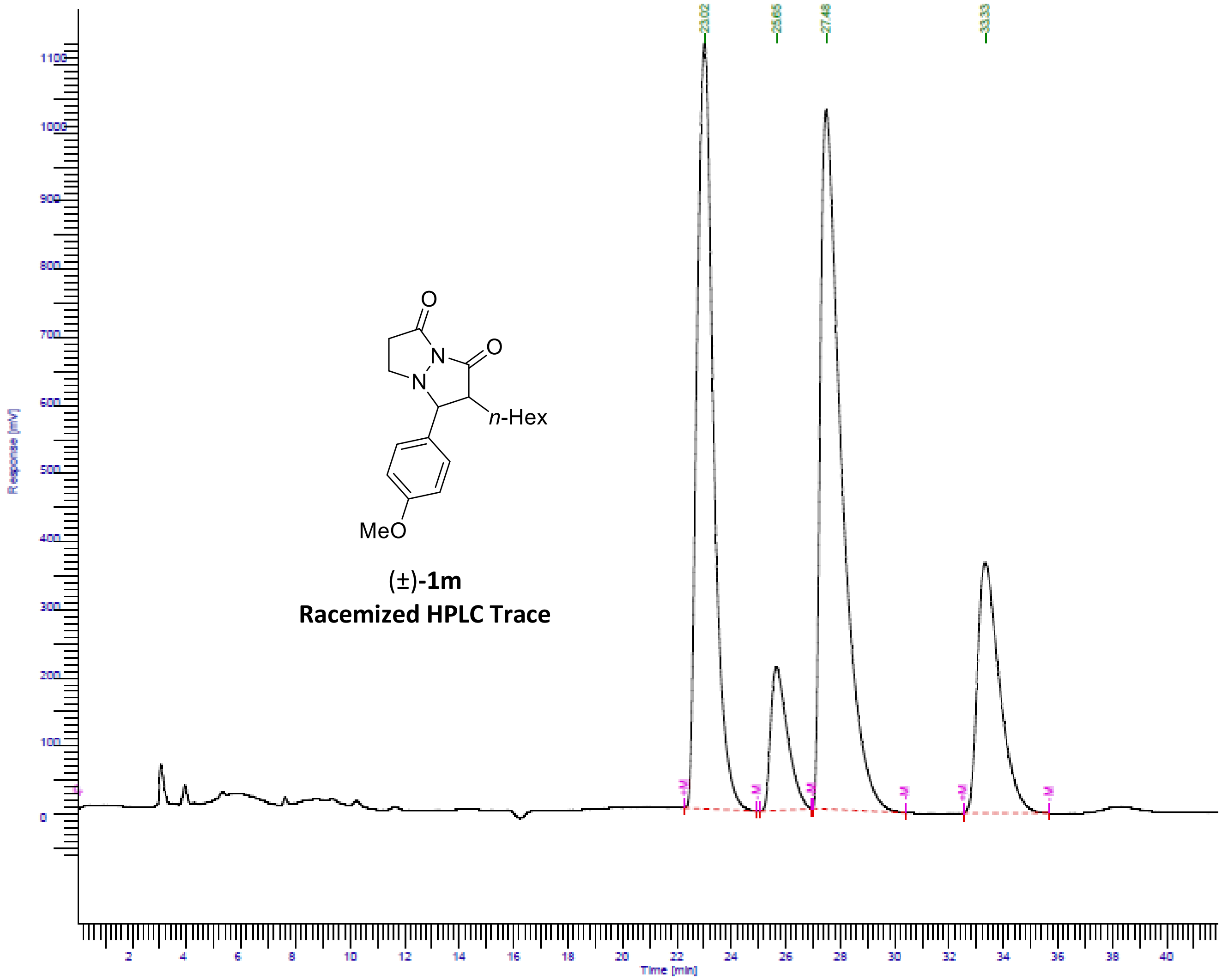

(±)-**1m**
Racemized HPLC Trace

Peak #	Time [min]	Area [µV·s]	Height [µV]	Area [%]	Norm. Area [%]	BL	Area/Height [s]
1	23.020	47463515.66	1.12e+06	35.72	35.72	*BB	42.2347
2	25.654	9066216.40	210405.23	6.82	6.82	*BB	43.0893
3	27.480	55608983.75	1.03e+06	41.84	41.84	*BB	54.1594
4	33.329	20754855.87	367717.41	15.62	15.62	*BB	56.4424

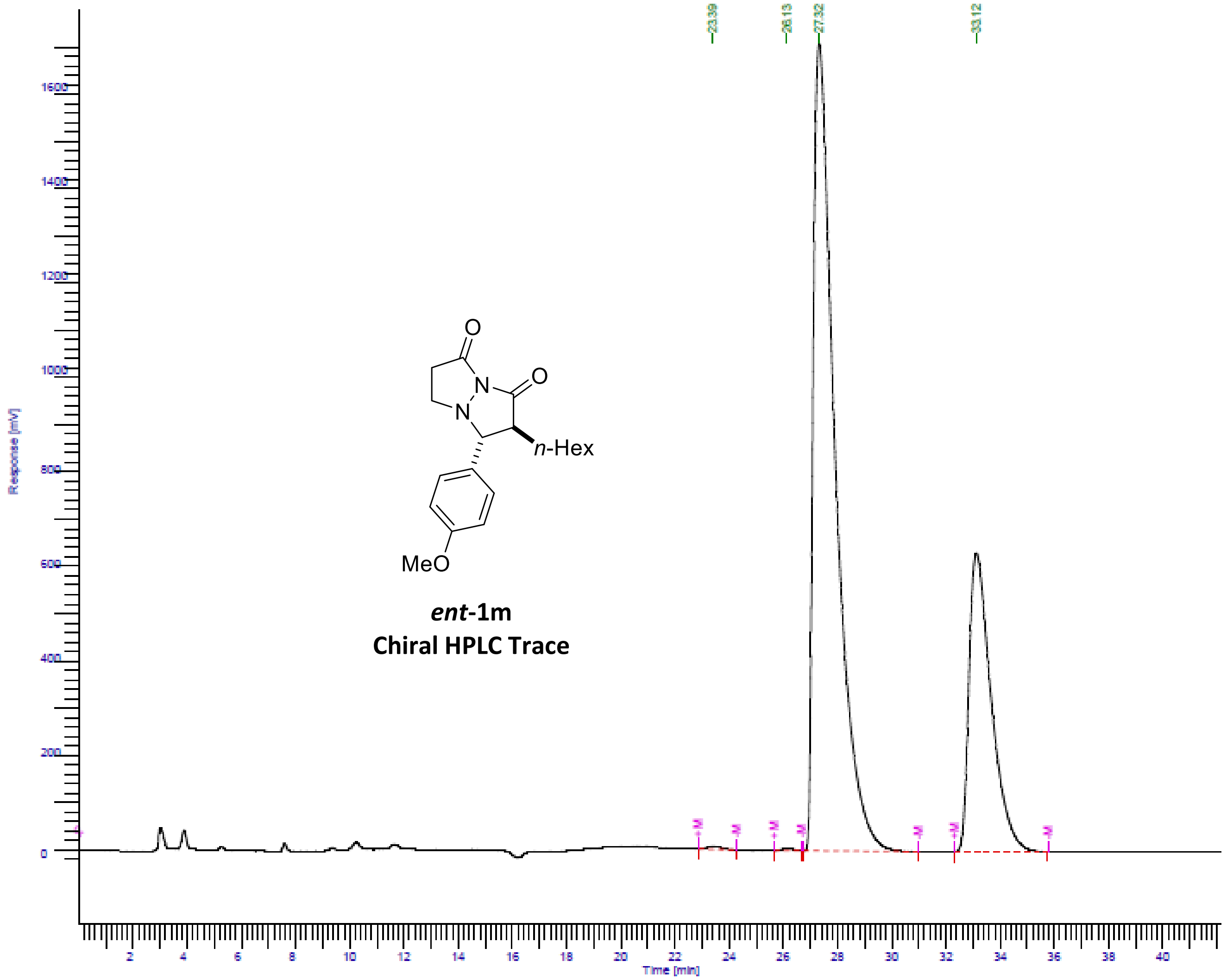

Peak #	Time [min]	Area [µV·s]	Height [µV]	Area [%]	Norm. Area [%]	BL	Area/Height [s]
1	23.393	240339.70	6375.42	0.18	0.18	*BB	37.6978
2	26.130	143629.46	4587.67	0.11	0.11	*BB	31.3077
3	27.322	95501404.74	1.71e+06	72.37	72.37	*BB	55.8487
4	33.124	36085677.71	632841.76	27.34	27.34	*BB	57.0216

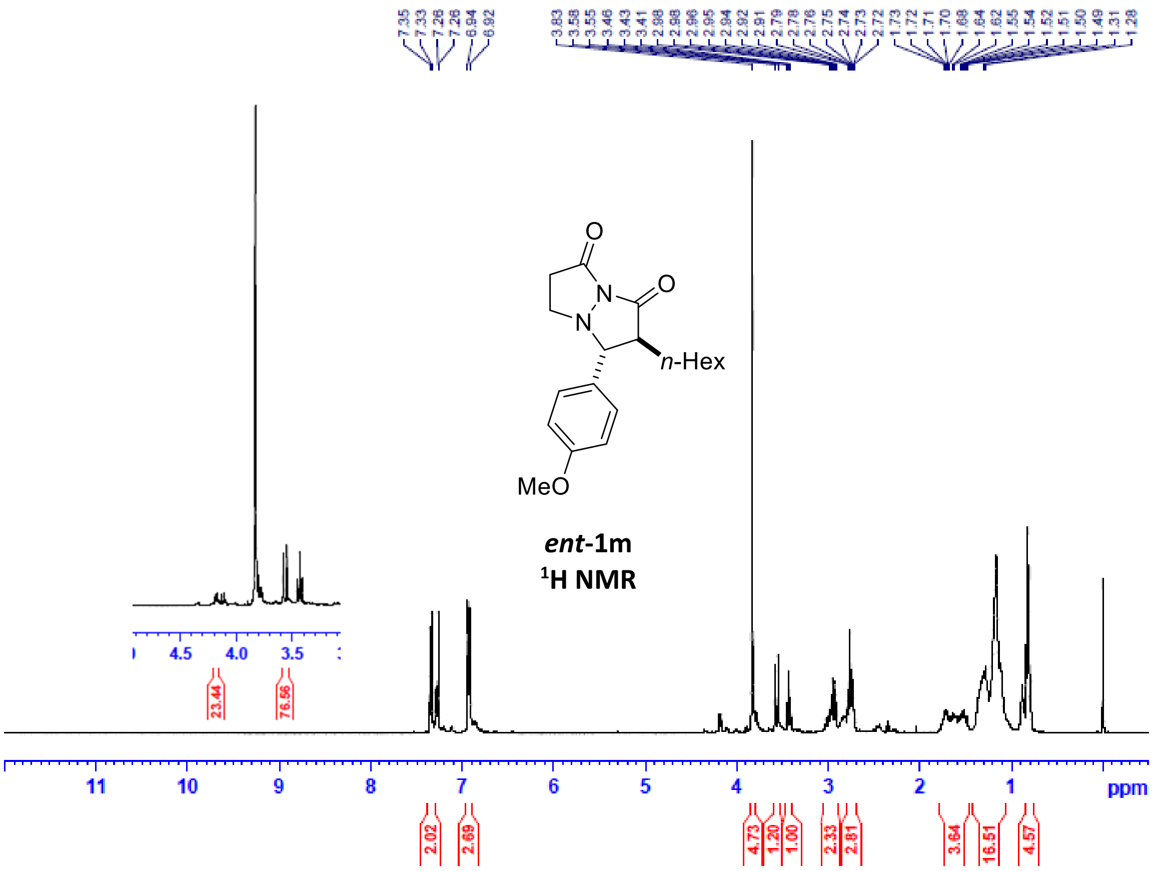

n-Hex
MeO
ent-1m
^{1}H NMR
ppm

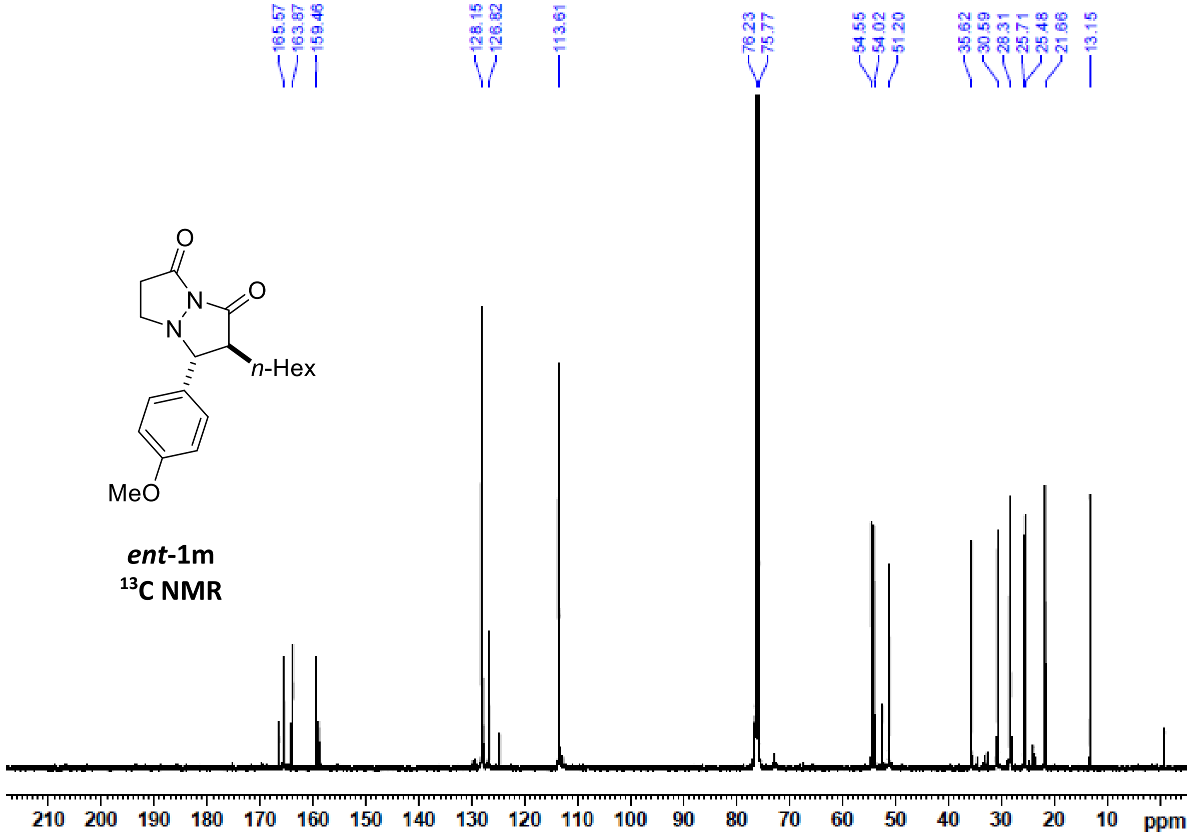

n-Hex
MeO
ent-1m
^{13}C NMR
ppm

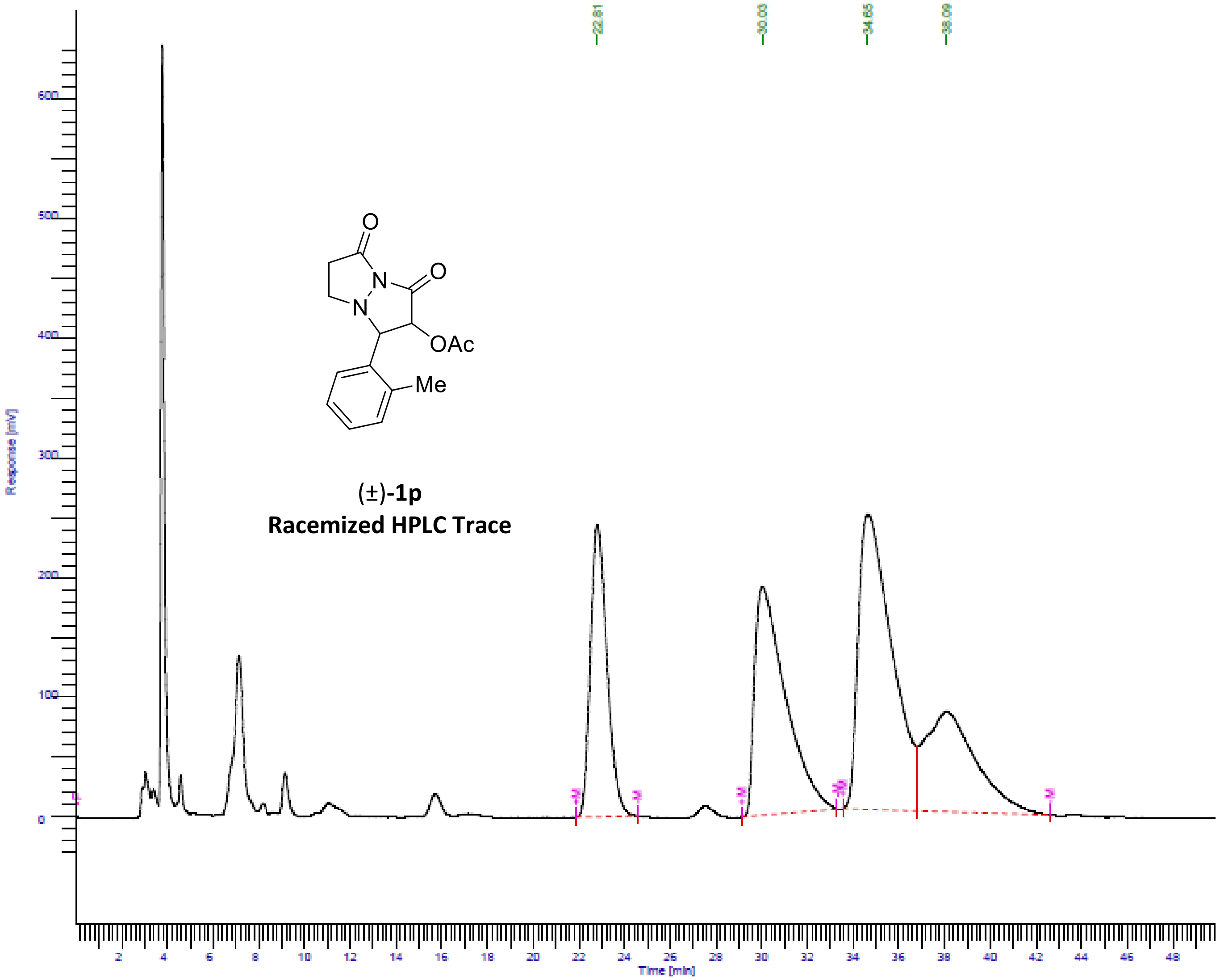

Peak #	Time [min]	Area [µV·s]	Height [µV]	Area [%]	Norm. Area [%]	BL	Area/Height [s]
1	22.811	12246806.86	243967.00	17.93	17.93	*BB	50.1986
2	30.030	17936009.65	190922.08	26.26	26.26	*BB	93.9441
3	34.652	25154846.58	246572.36	36.83	36.83	*BV	102.0181
4	38.085	12963909.06	83948.20	18.98	18.98	*VB	154.4275

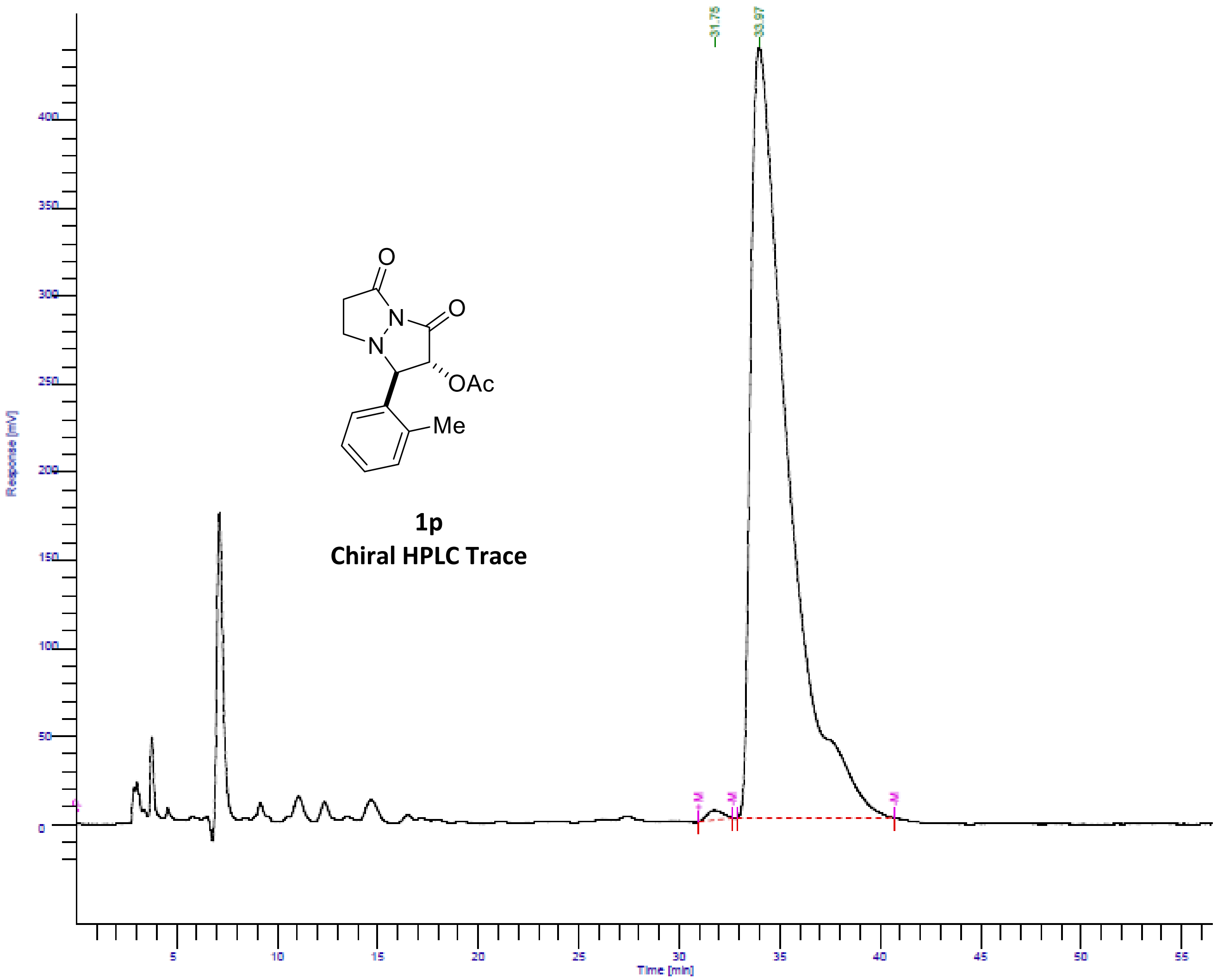

Peak #	Time [min]	Area [µV·s]	Height [µV]	Area [%]	Norm. Area [%]	BL	Area/Height [s]
1	31.748	310094.69	5604.07	0.55	0.55	*BB	55.3338
2	33.972	56136666.26	438011.23	99.45	99.45	*BB	128.1626

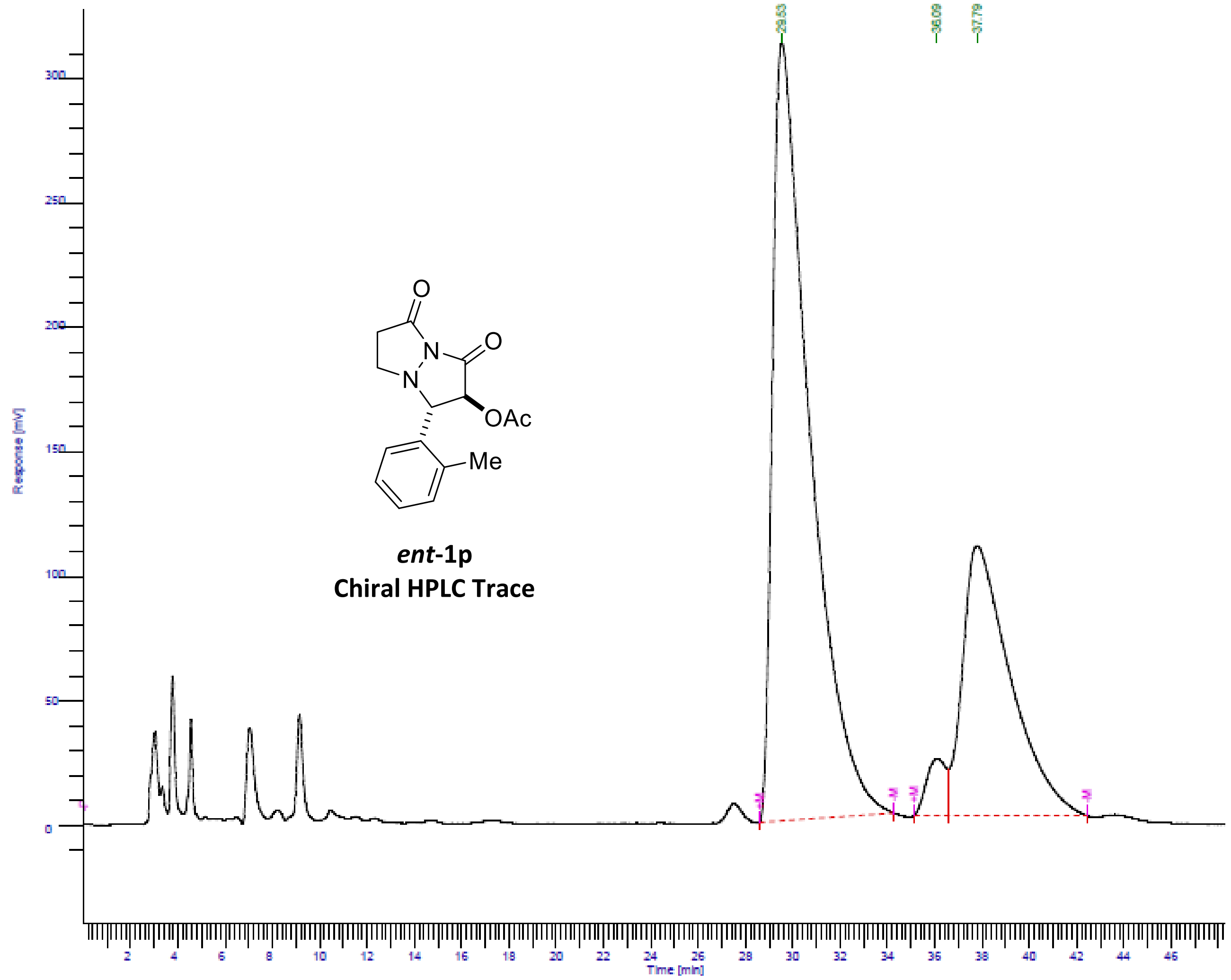

***ent*-1p**
Chiral HPLC Trace

Peak #	Time [min]	Area [µV·s]	Height [µV]	Area [%]	Norm. Area [%]	BL	Area/Height [s]
1	29.535	34326001.26	312557.88	67.44	67.44	*BB	109.8229
2	36.089	1283319.84	22649.38	2.52	2.52	*BV	56.6603
3	37.793	15285489.62	108462.42	30.03	30.03	*VB	140.9289

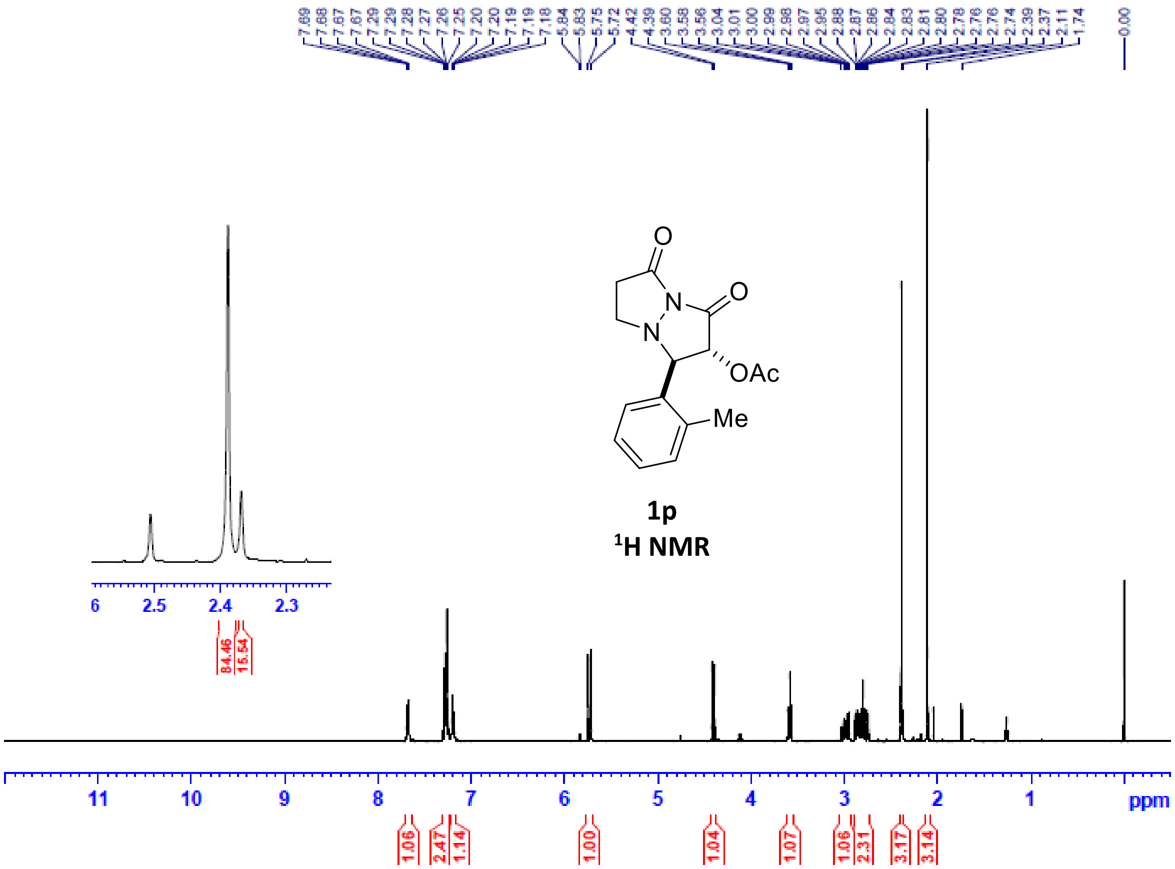
1p
1H NMR
ppm

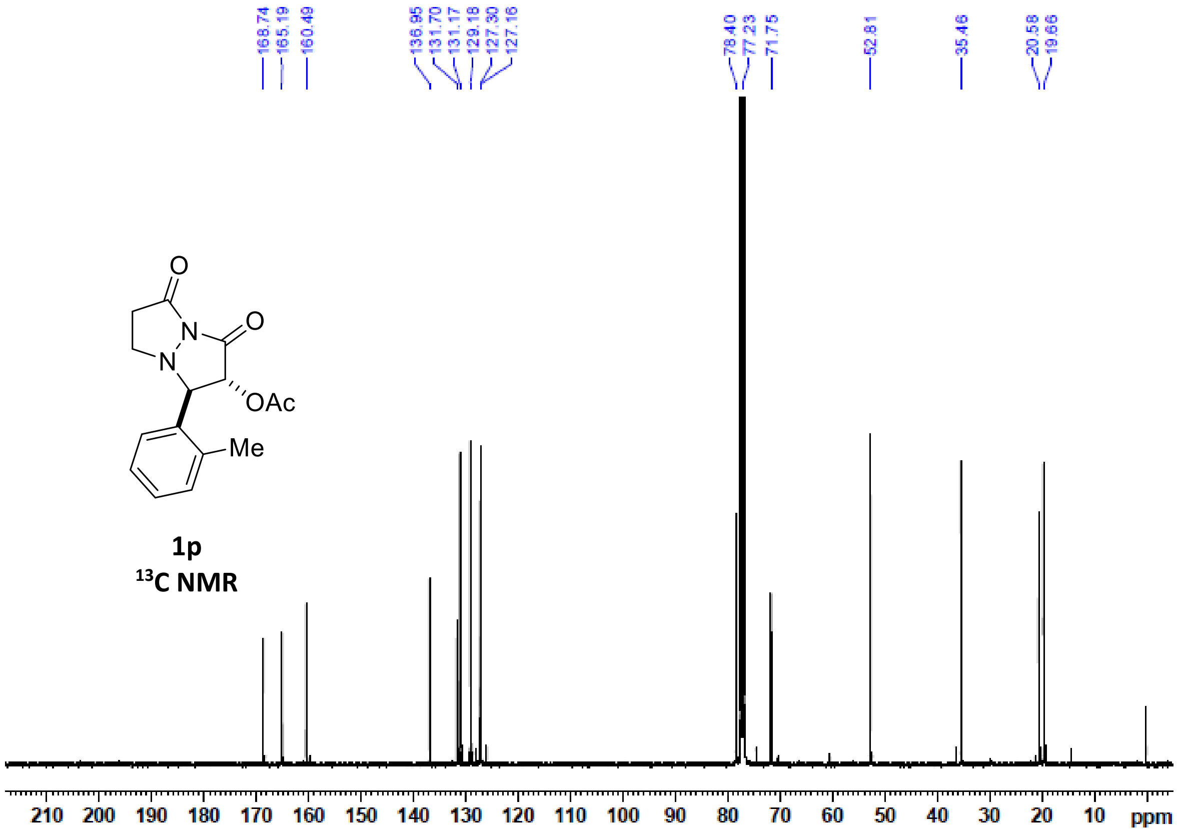
1p
13C NMR
ppm

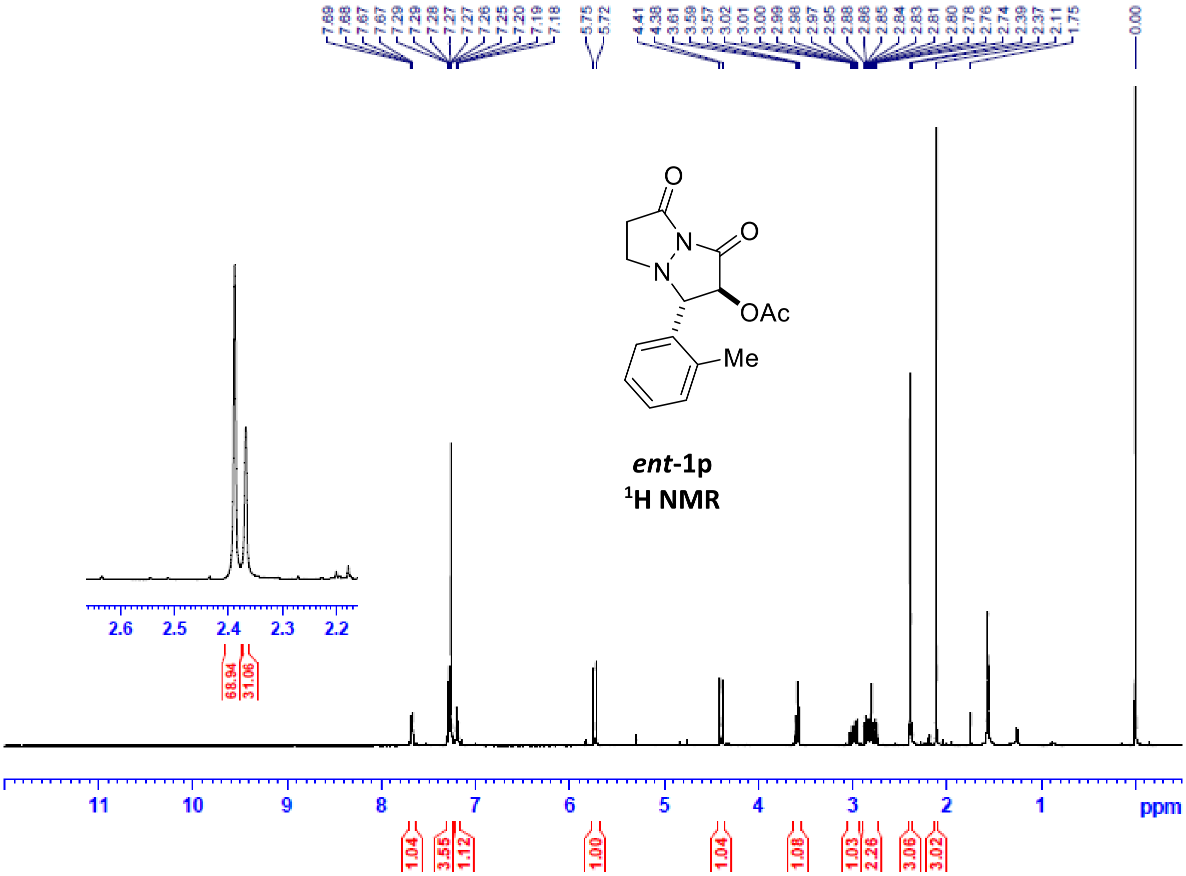
7.69 7.68 7.67 7.67 7.29 7.29 7.28 7.27 7.27 7.26 7.25 7.20 7.19 7.18
5.75 5.72
4.41 4.38 3.61 3.59 3.57 3.02 3.01 3.00 2.99 2.98 2.97 2.95 2.88 2.86 2.85 2.84 2.83 2.81 2.80 2.78 2.76 2.74 2.39 2.37 2.11 1.75
0.00
OAc
Me
ent-1p
1H NMR
2.6 2.5 2.4 2.3 2.2
68.94 31.06
11 10 9 8 7 6 5 4 3 2 1 ppm
1.04 3.55 1.12 1.00 1.04 1.08 1.03 2.26 3.06 3.02

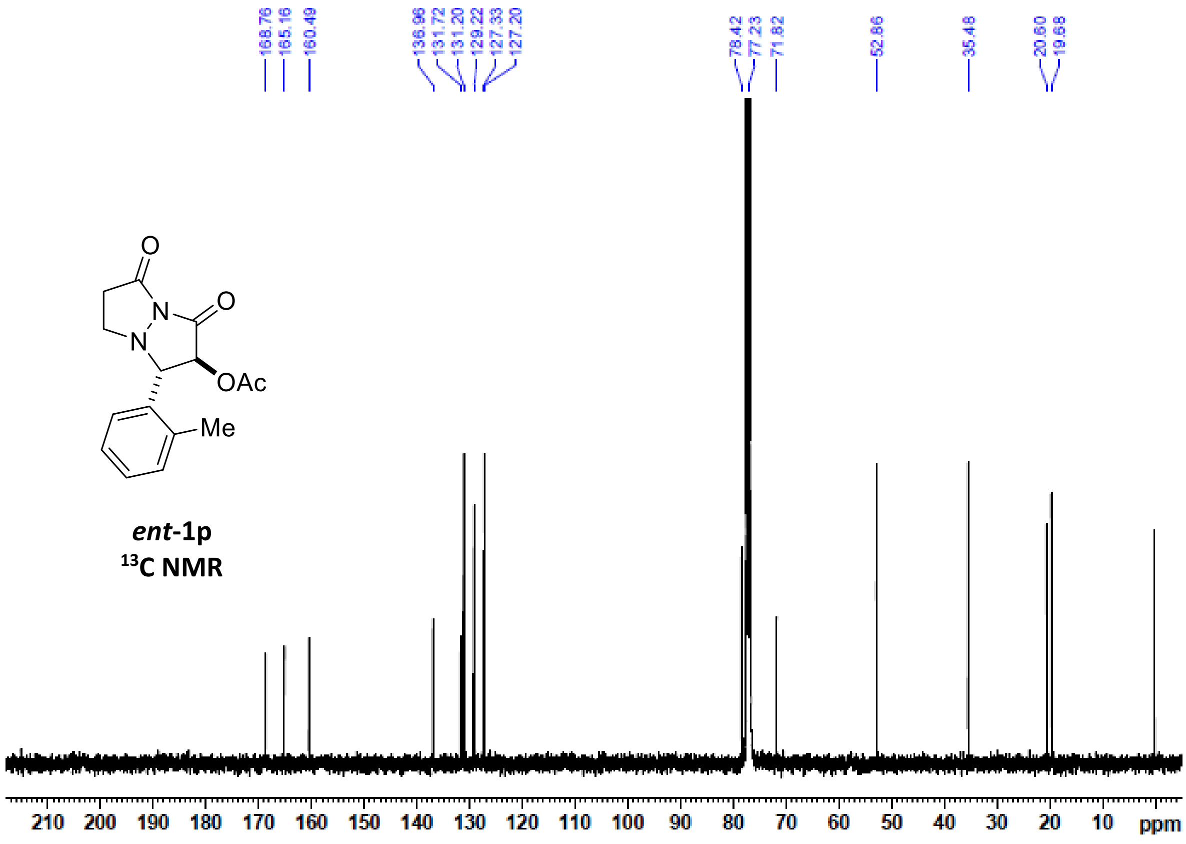
168.76 165.16 160.49
136.96 131.72 131.20 129.22 127.33 127.20
78.42 77.23 71.82
52.86
35.48
20.60 19.68
OAc
Me
ent-1p
13C NMR
210 200 190 180 170 160 150 140 130 120 110 100 90 80 70 60 50 40 30 20 10 ppm

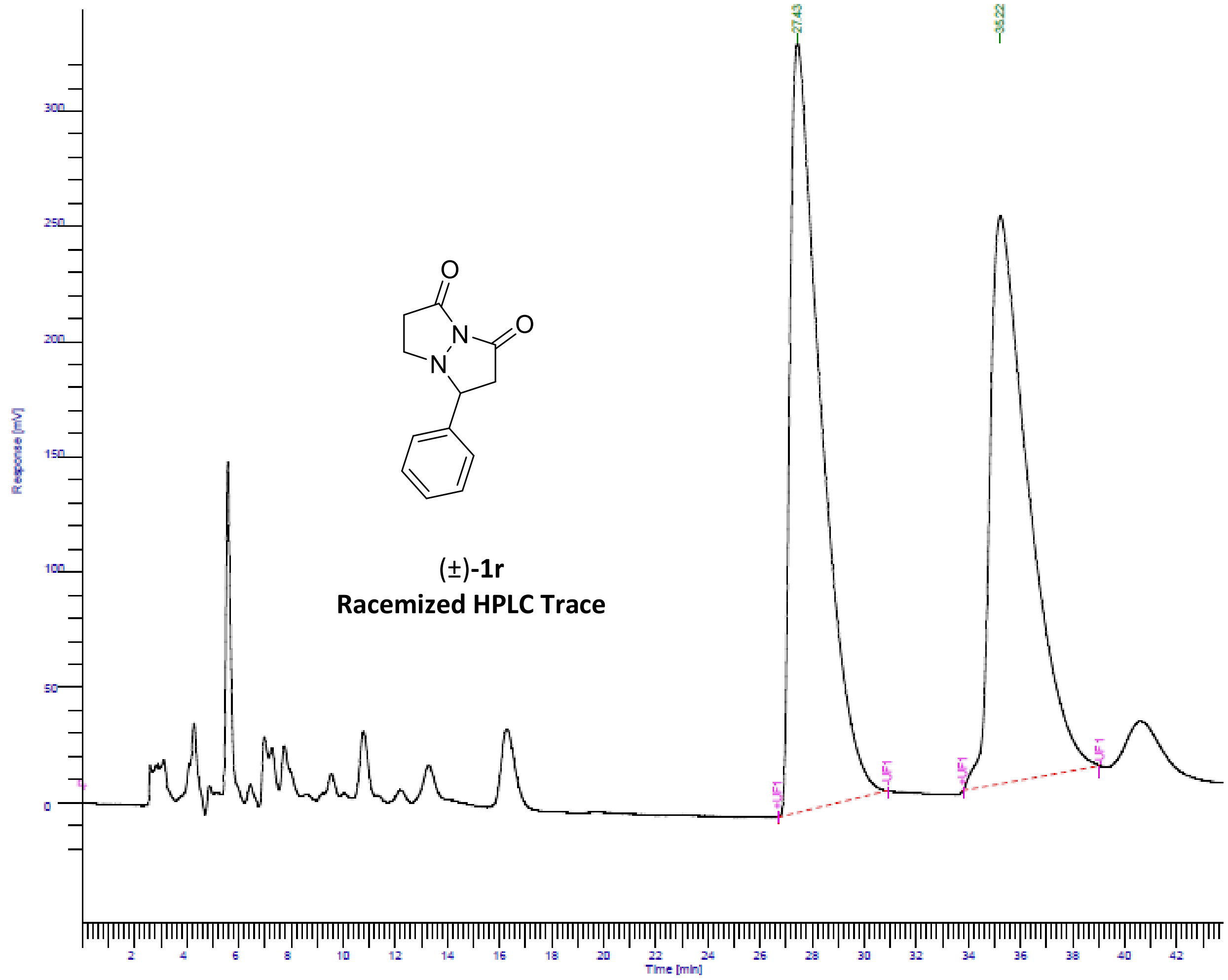

(±)-**1r**
Racemized HPLC Trace

Peak #	Time [min]	Area [µV·s]	Height [µV]	Area [%]	Norm. Area [%]	BL	Area/Height [s]
1	27.434	27730981.10	333592.47	52.25	52.25	*MM	83.1283
2	35.222	25345447.08	246449.36	47.75	47.75	*MM	102.8424

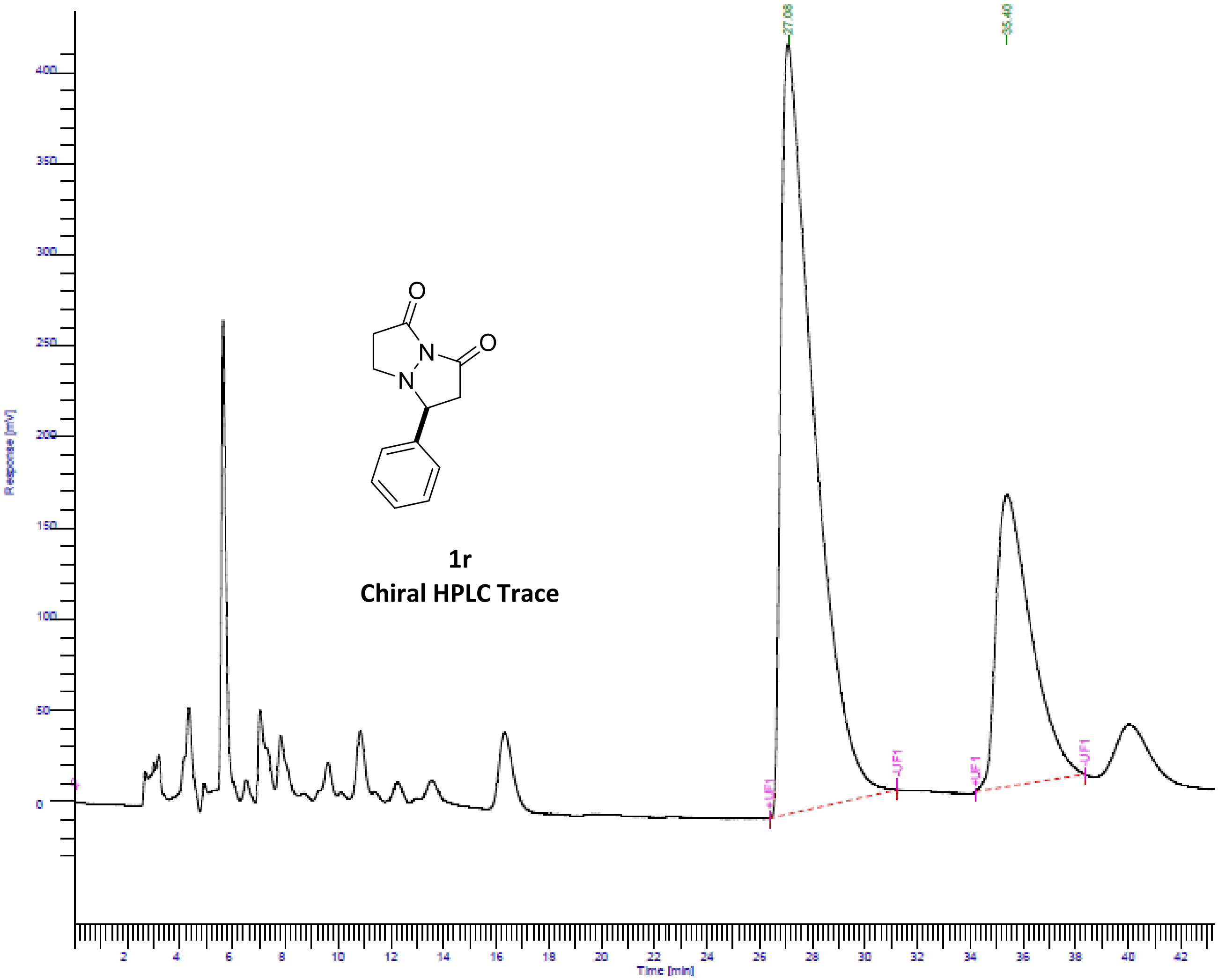

Peak #	Time [min]	Area [µV·s]	Height [µV]	Area [%]	Norm. Area [%]	BL	Area/Height [s]
1	27.082	36973224.23	422079.18	71.42	71.42	*MM	87.5978
2	35.398	14793920.41	160032.84	28.58	28.58	*MM	92.4430

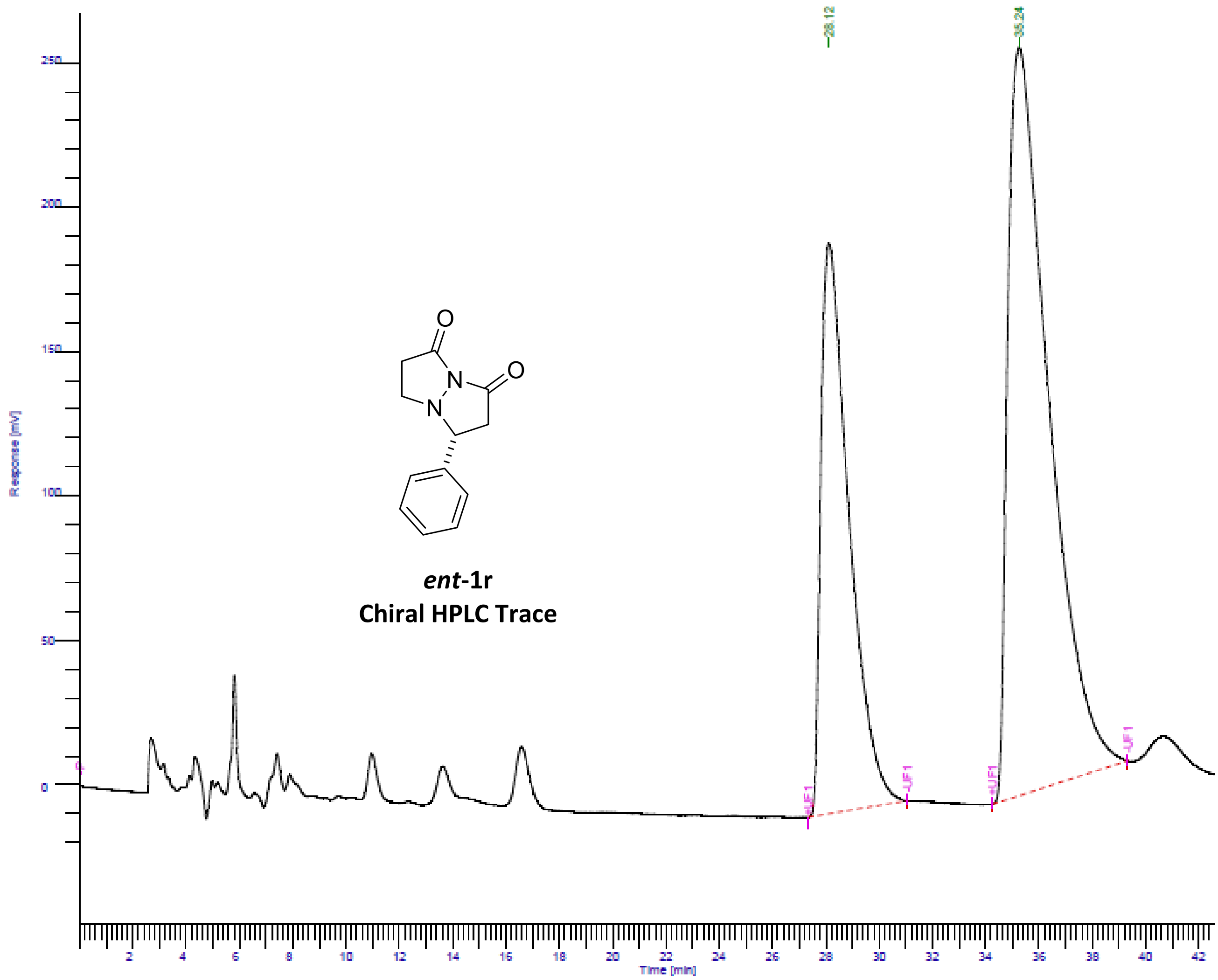

Peak #	Time [min]	Area [µV·s]	Height [µV]	Area [%]	Norm. Area [%]	BL	Area/Height [s]
1	28.116	14450100.37	197504.16	34.22	34.22	*MM	73.1635
2	35.244	27777315.46	259123.75	65.78	65.78	*MM	107.1971

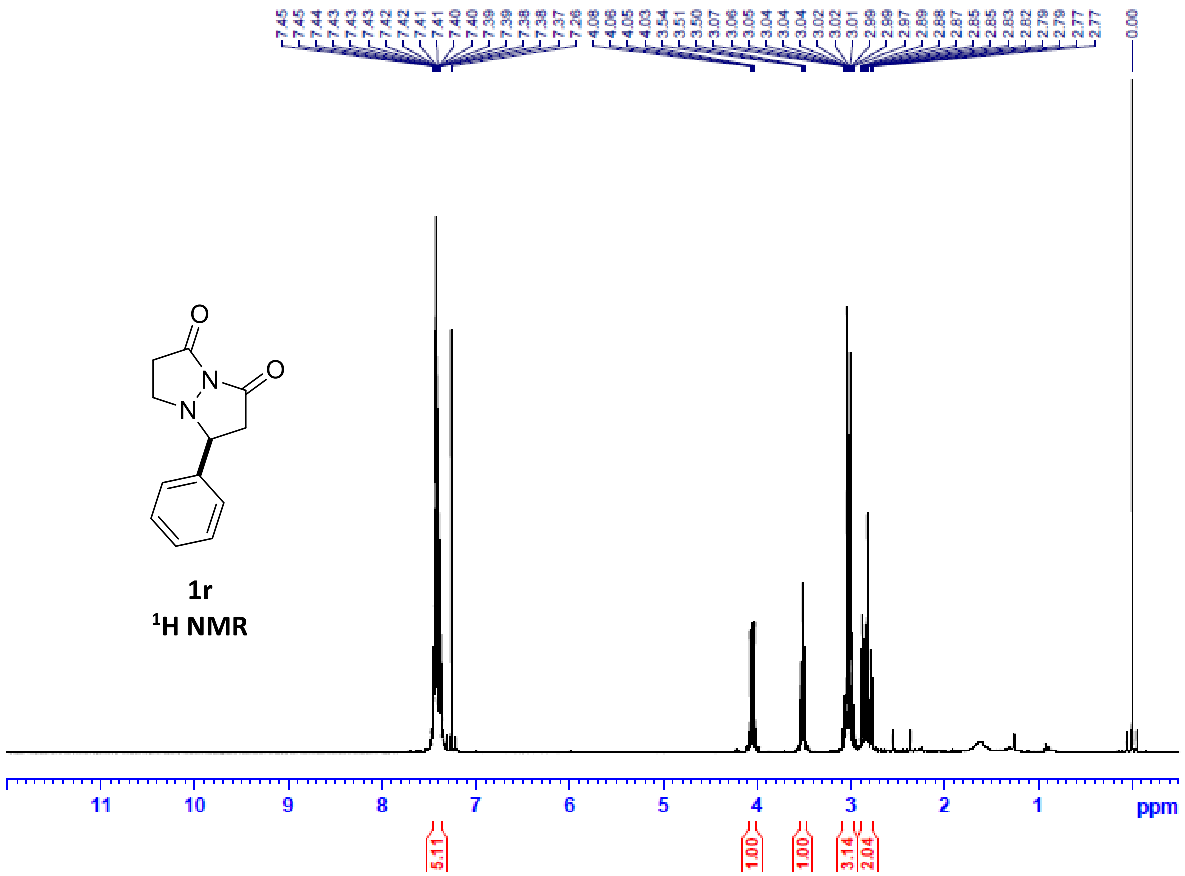

1r
1H NMR
ppm

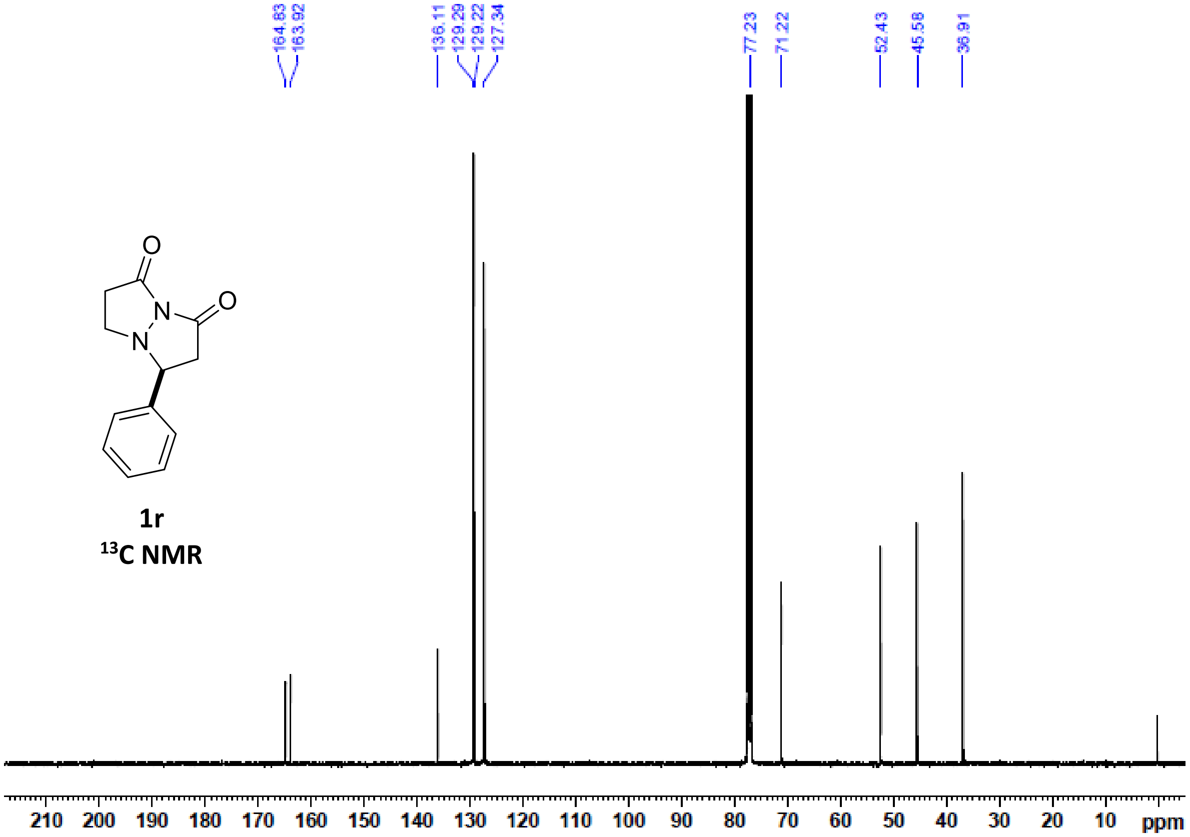

1r
13C NMR
ppm

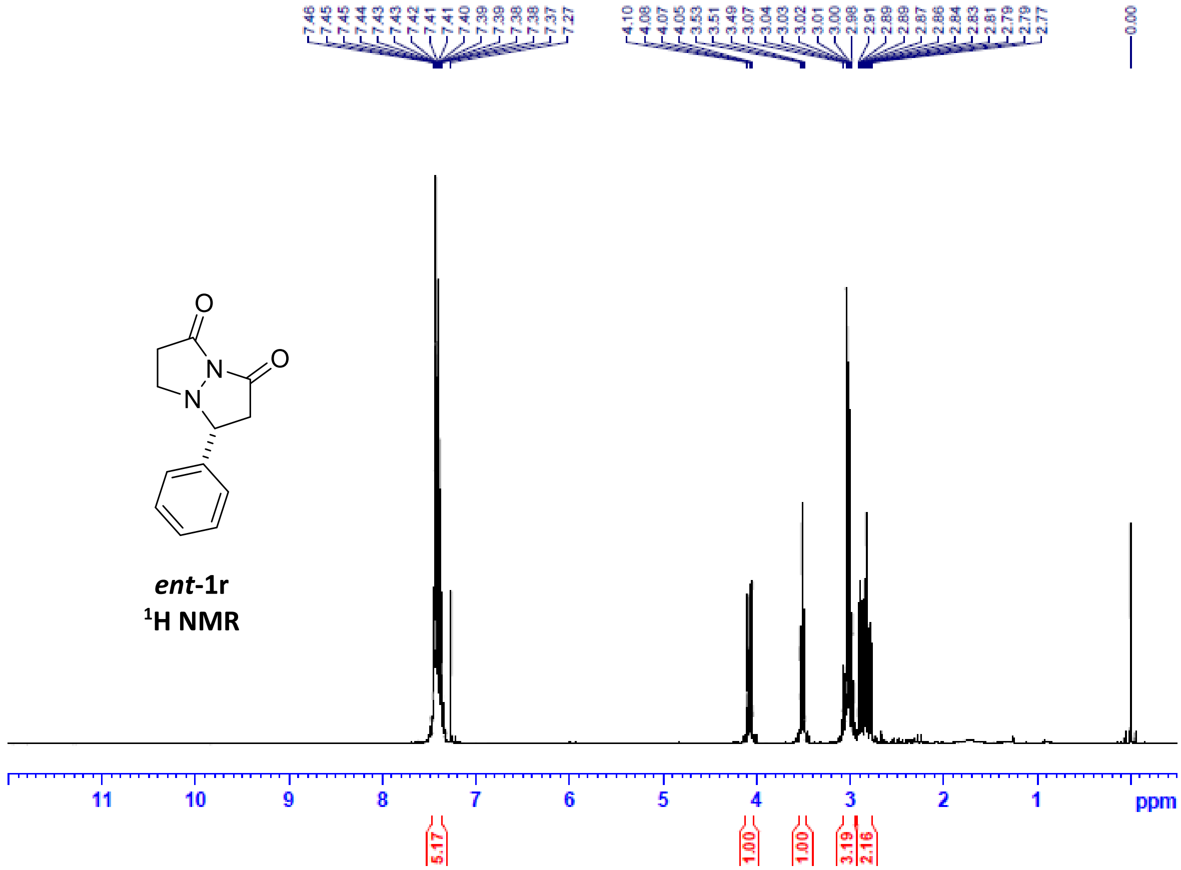
7.46
7.45
7.45
7.44
7.43
7.43
7.42
7.41
7.41
7.40
7.39
7.39
7.38
7.38
7.37
7.27
4.10
4.08
4.07
4.05
3.53
3.51
3.49
3.07
3.04
3.03
3.02
3.01
3.00
2.98
2.91
2.89
2.89
2.87
2.86
2.84
2.83
2.81
2.79
2.79
2.77
0.00
ent-1r
^{1}H NMR
11 10 9 8 7 6 5 4 3 2 1 ppm
5.17
1.00
1.00
3.19
2.16

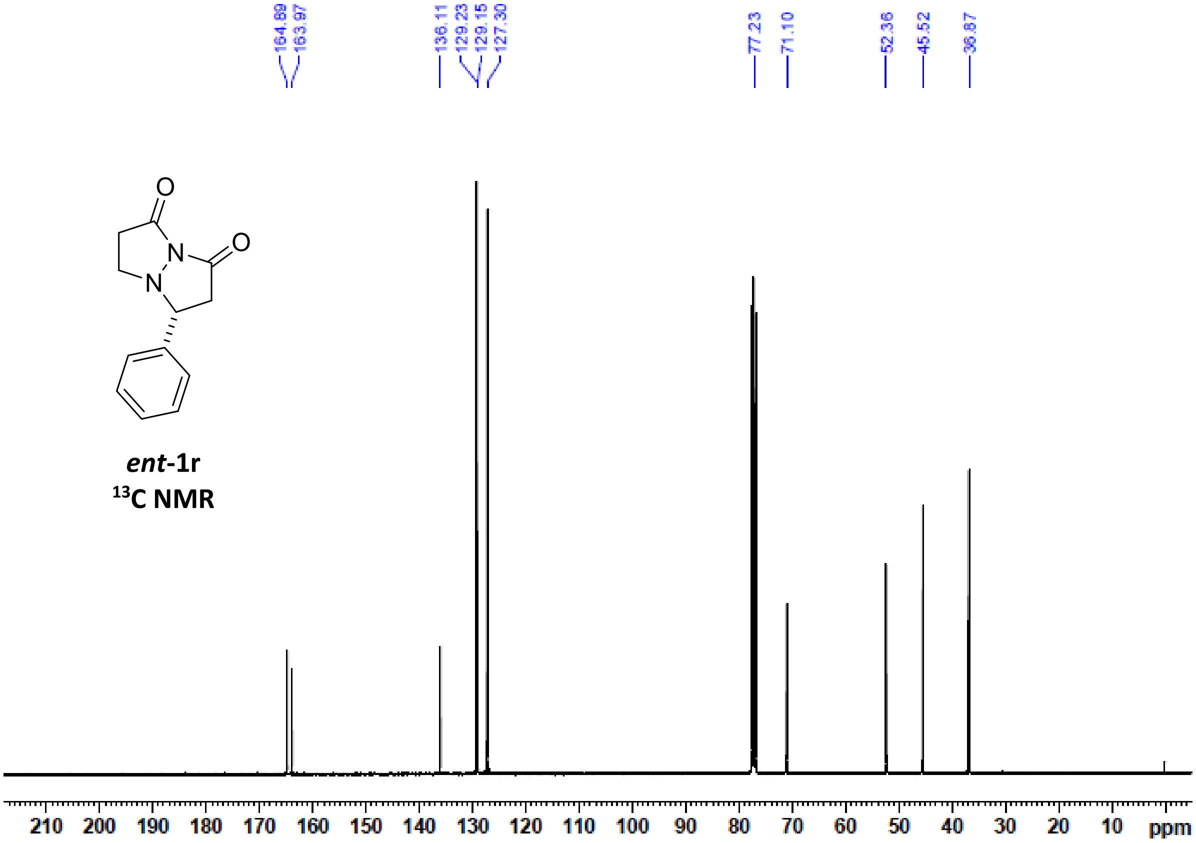
164.89
163.97
136.11
129.23
129.15
127.30
77.23
71.10
52.36
45.52
36.87
ent-1r
^{13}C NMR
210 200 190 180 170 160 150 140 130 120 110 100 90 80 70 60 50 40 30 20 10 ppm

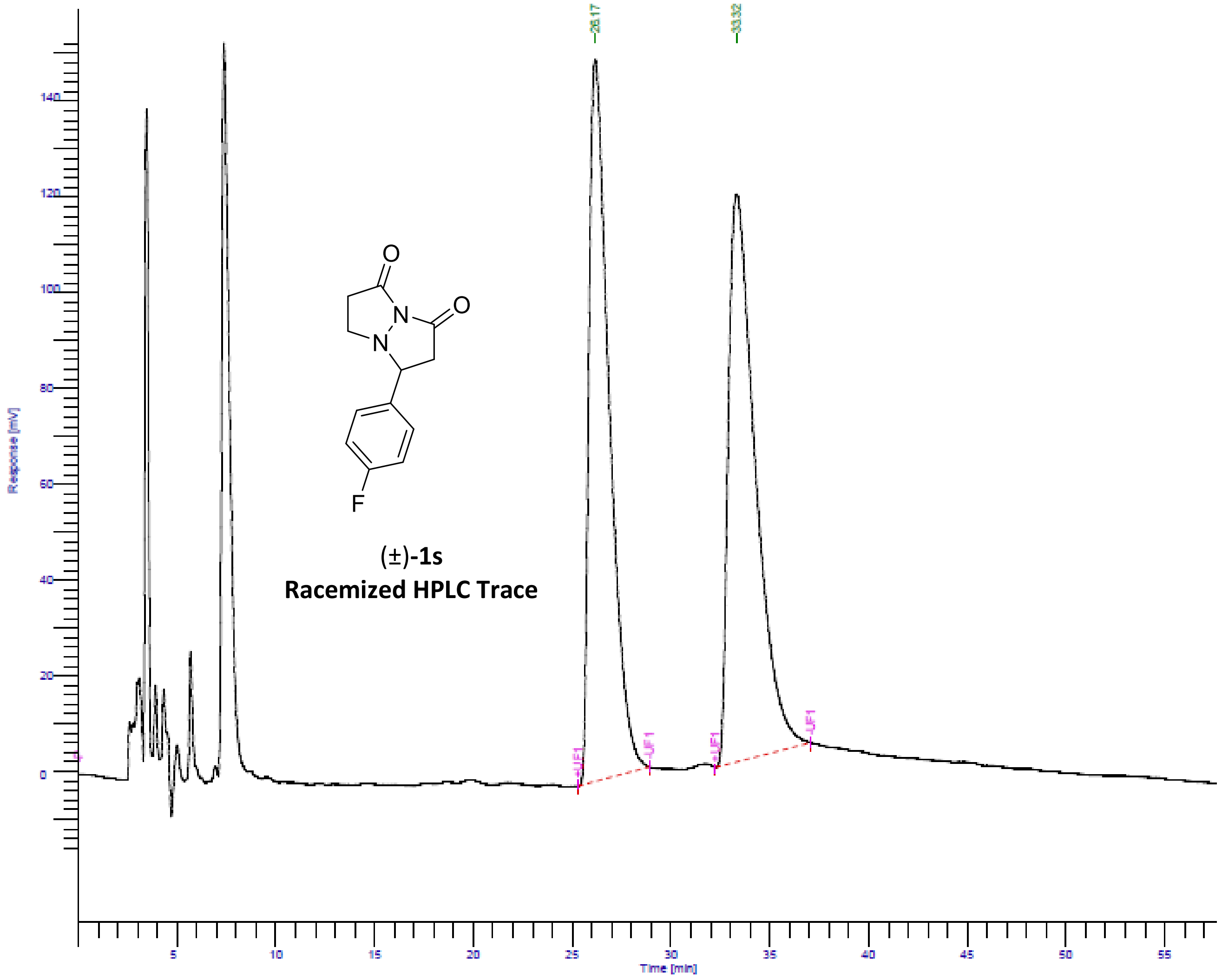

Peak #	Time [min]	Area [µV·s]	Height [µV]	Area [%]	Norm. Area [%]	BL	Area/Height [s]
1	26.165	10756087.48	150793.67	48.86	48.86	*MM	71.3298
2	33.315	11257003.97	118550.84	51.14	51.14	*MM	94.9551

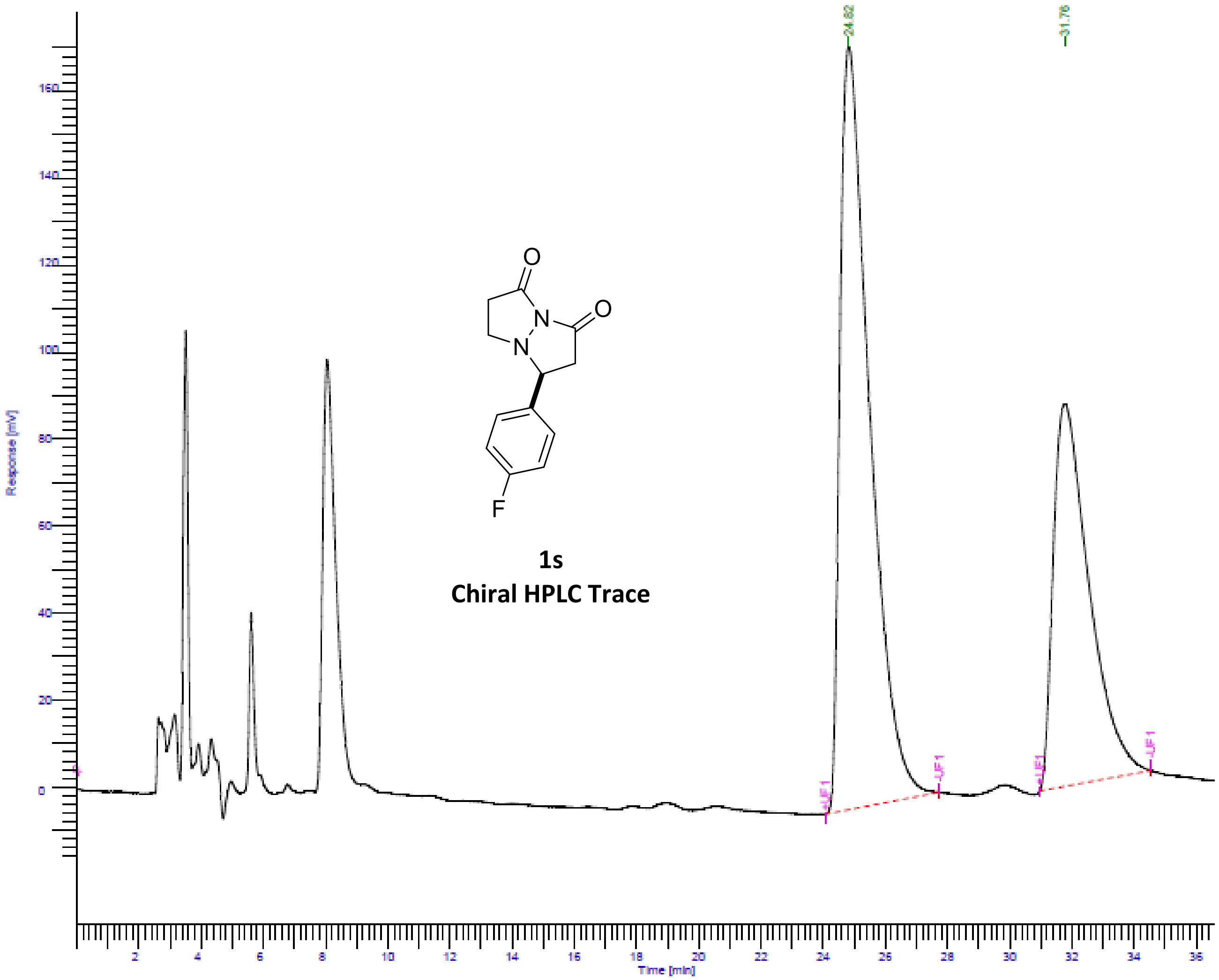

Peak #	Time [min]	Area [µV·s]	Height [µV]	Area [%]	Norm. Area [%]	BL	Area/Height [s]
1	24.816	11710896.41	175581.17	62.57	62.57	*MM	66.6979
2	31.761	7006517.43	87929.57	37.43	37.43	*MM	79.6833

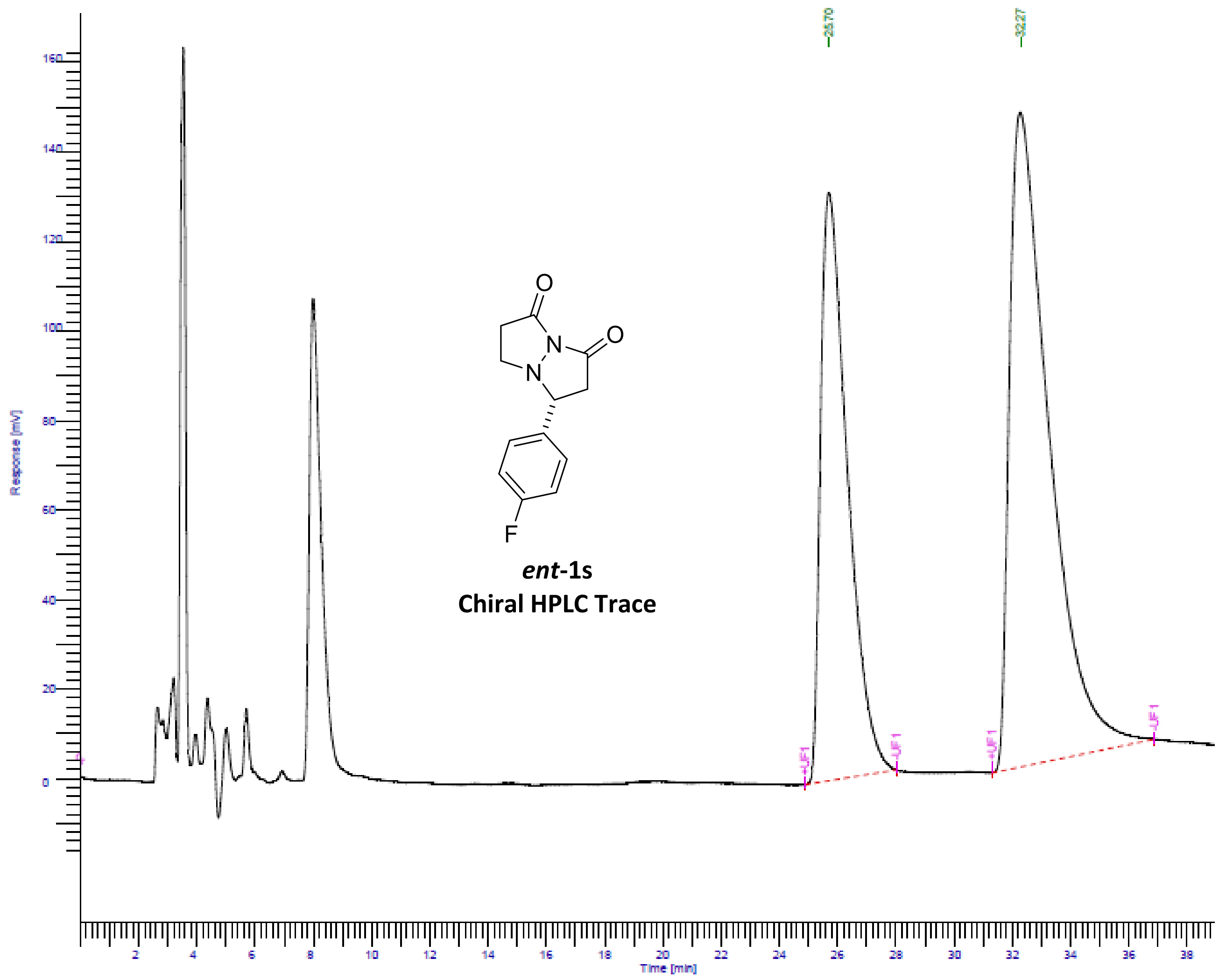

ent-1s
Chiral HPLC Trace

Peak #	Time [min]	Area [µV·s]	Height [µV]	Area [%]	Norm. Area [%]	BL	Area/Height [s]
1	25.696	8444333.34	131311.81	37.90	37.90	*MM	64.3075
2	32.274	13837945.54	146240.21	62.10	62.10	*MM	94.6248

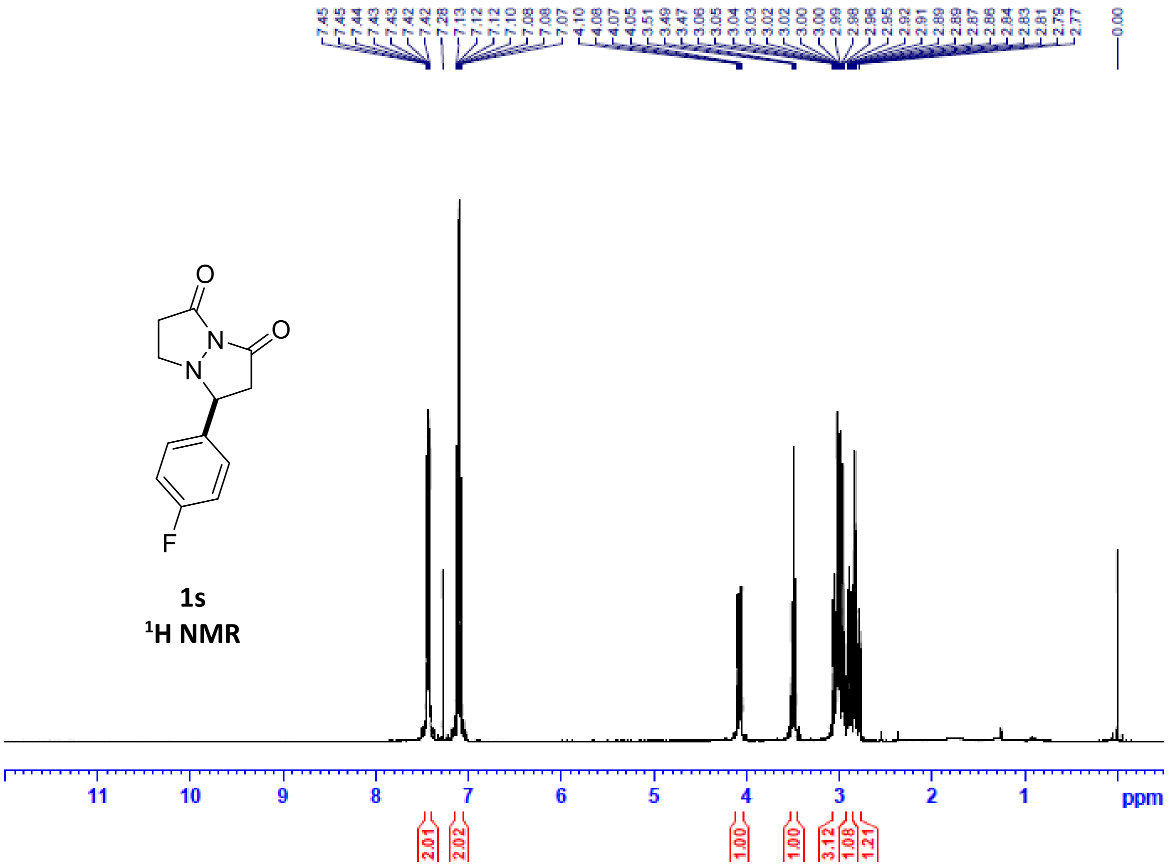

1s
^{1}H NMR

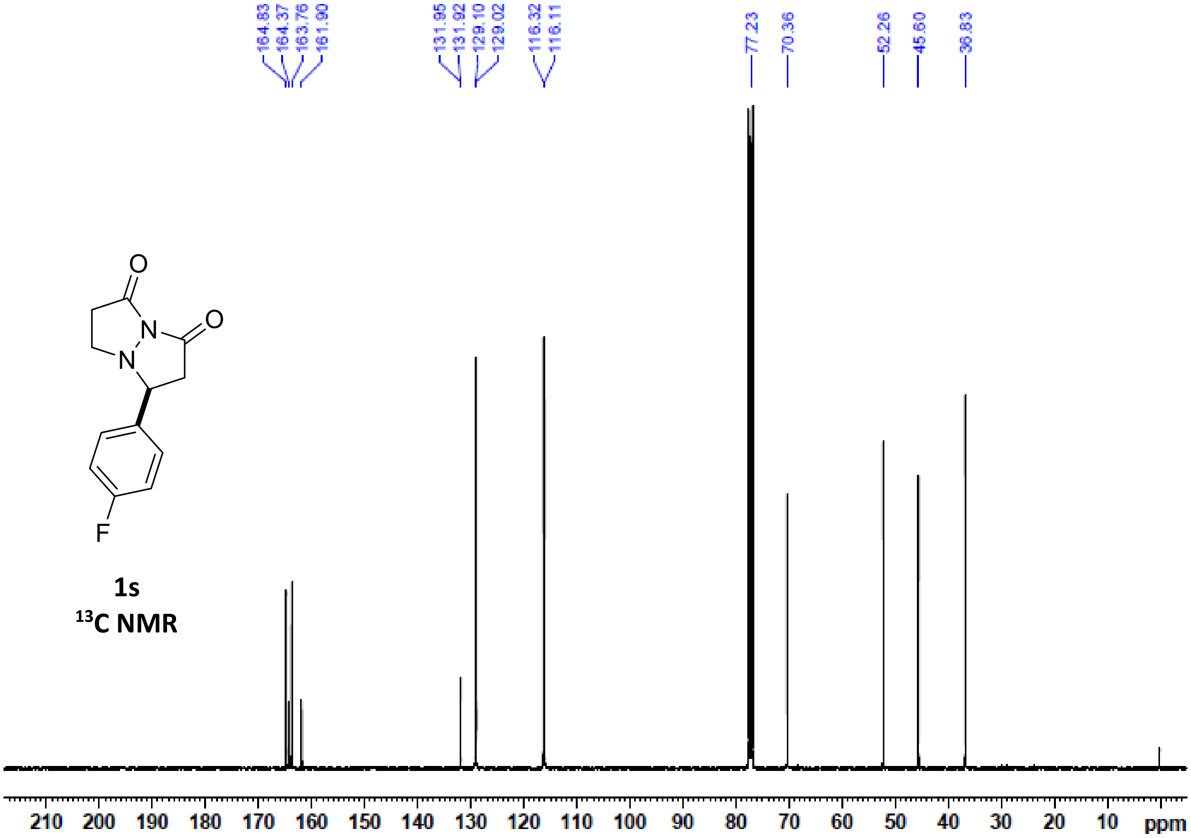

1s
^{13}C NMR

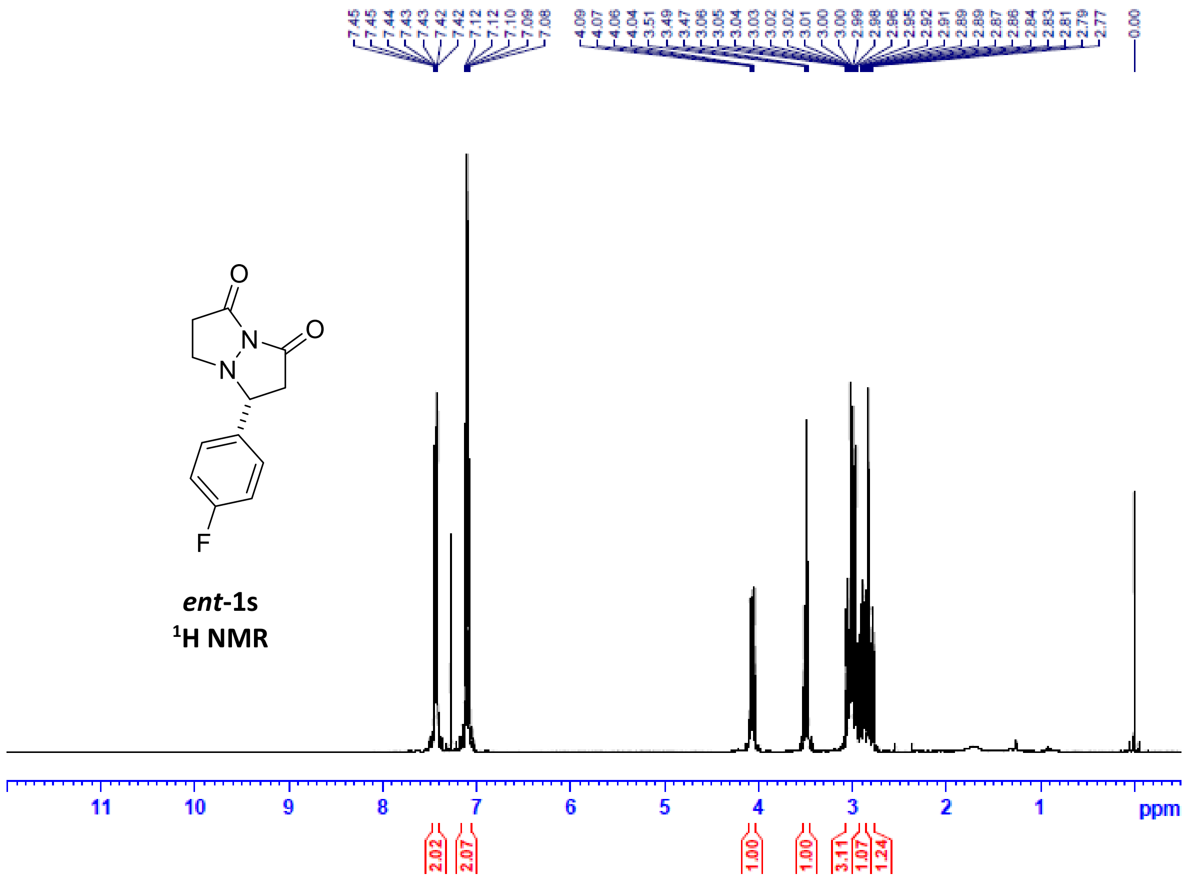

***ent*-1s**
^{1}H NMR

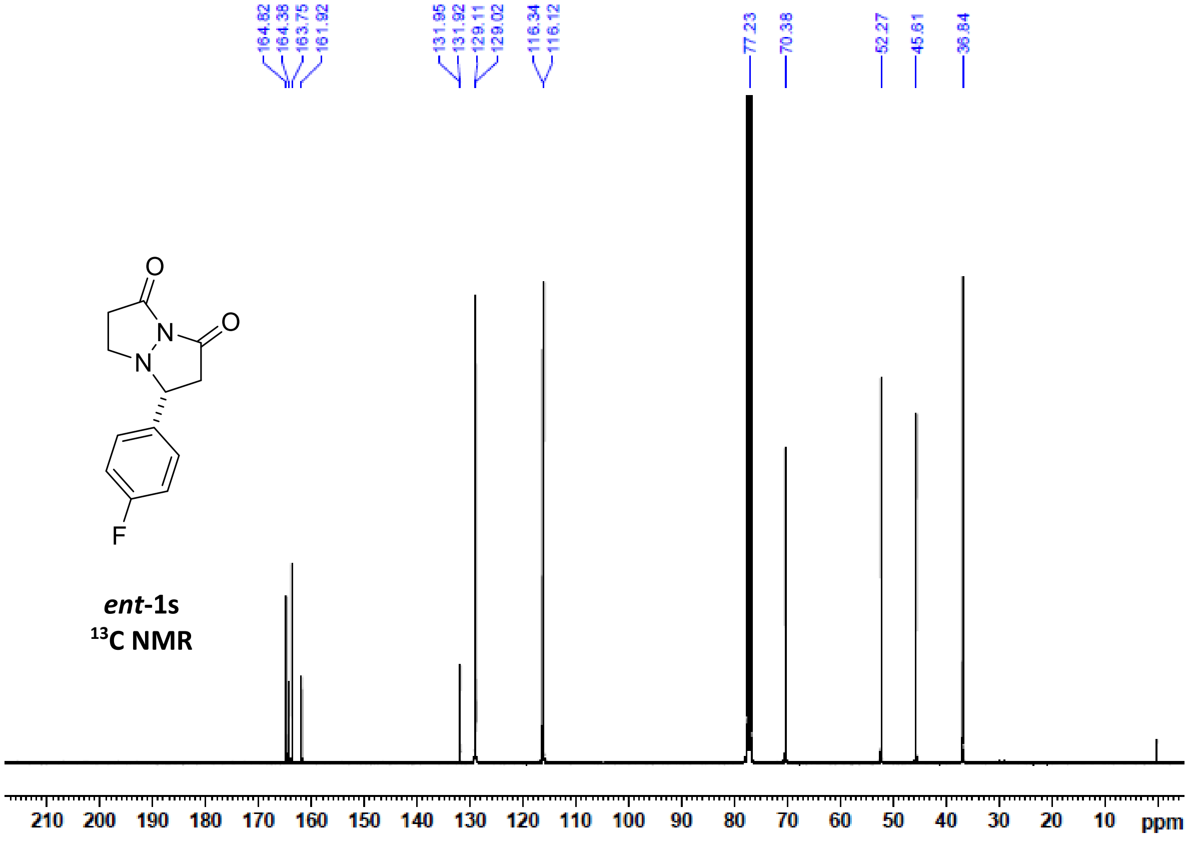

***ent*-1s**
^{13}C NMR

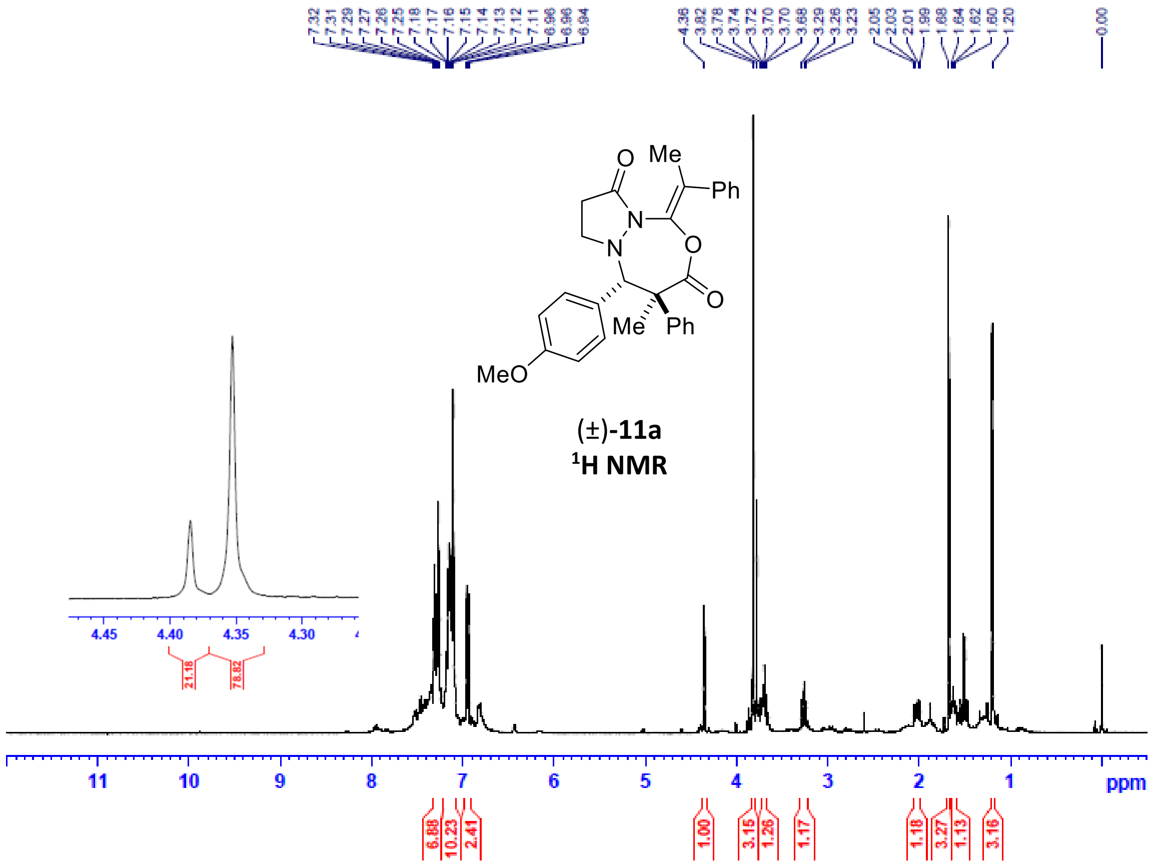
(±)-11a
1H NMR

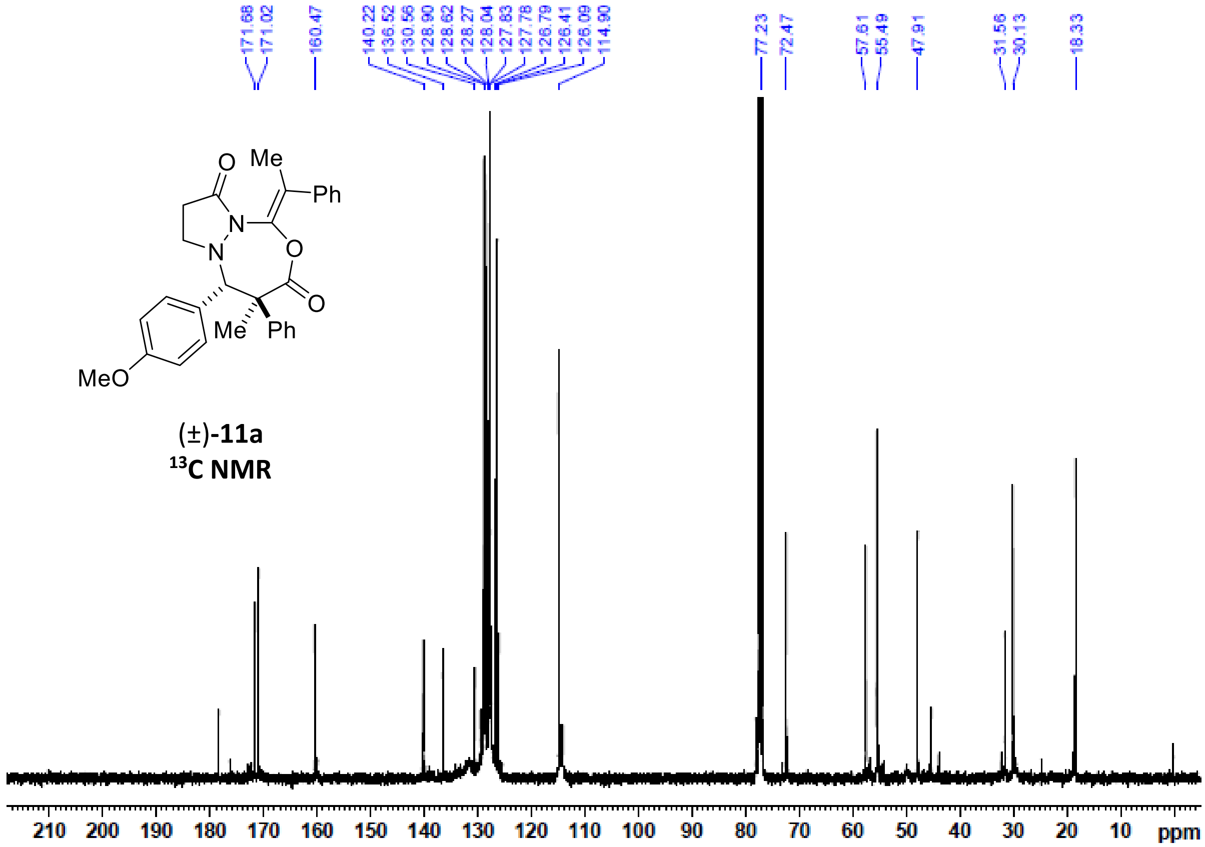
(±)-11a
13C NMR

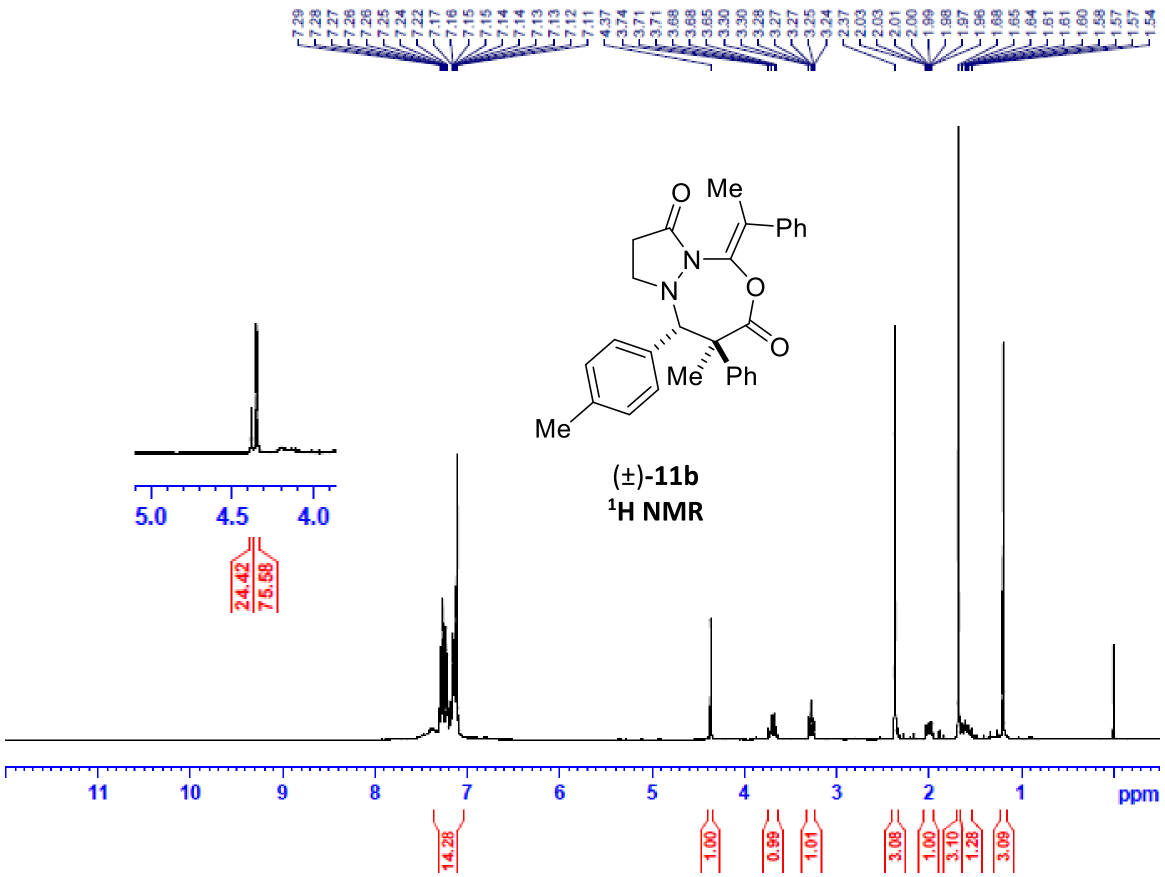
(±)-11b
¹H NMR
ppm

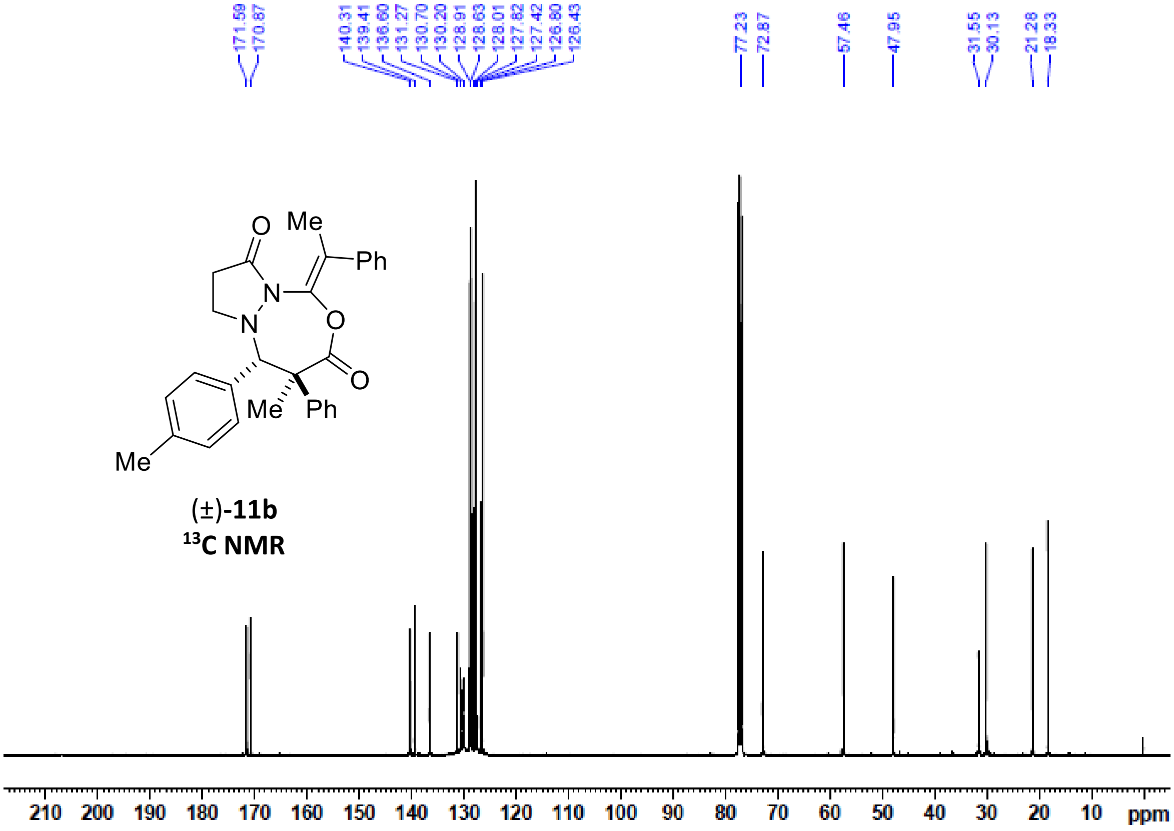
(±)-11b
¹³C NMR
ppm

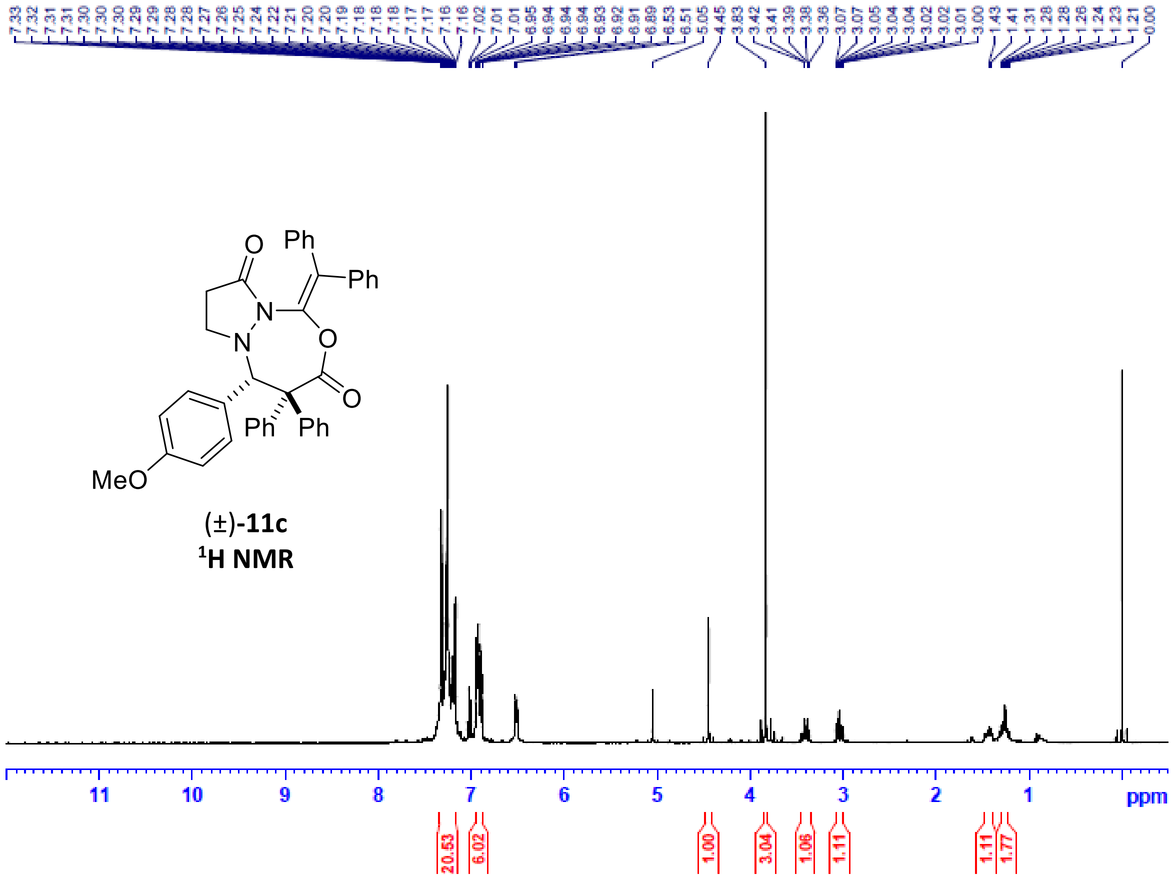
7.33 7.32 7.31 7.31 7.30 7.30 7.29 7.29 7.28 7.28 7.27 7.26 7.25 7.24 7.22 7.21 7.20 7.20 7.19 7.18 7.18 7.18 7.17 7.17 7.16 7.16 7.02 7.01 7.01 6.95 6.94 6.94 6.94 6.93 6.92 6.91 6.89 6.53 6.51 5.05 4.45 3.83 3.42 3.41 3.39 3.38 3.36 3.07 3.07 3.05 3.04 3.04 3.02 3.02 3.01 3.00 1.43 1.41 1.31 1.28 1.28 1.26 1.24 1.23 1.21 0.00
O
Ph
Ph
N
N
O
O
Ph
Ph
MeO
(±)-11c
^{1}H NMR
11 10 9 8 7 6 5 4 3 2 1 ppm
20.53 6.02 1.00 3.04 1.06 1.11 1.11 1.77

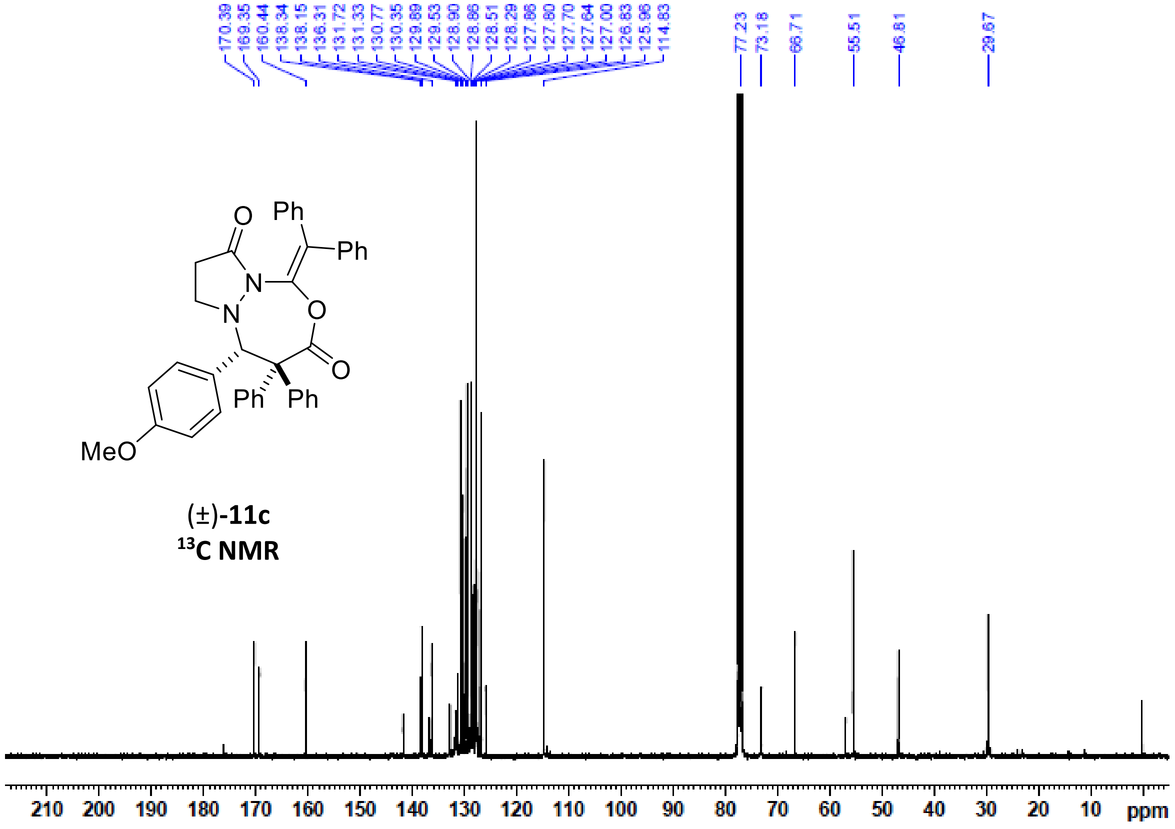
170.39 169.35 160.44 138.34 138.15 136.31 131.72 131.33 130.77 130.35 129.89 129.53 128.90 128.86 128.51 128.29 127.86 127.80 127.70 127.64 127.00 126.83 125.96 114.83
77.23 73.18 66.71 55.51 46.81 29.67
O
Ph
Ph
N
N
O
O
Ph
Ph
MeO
(±)-11c
^{13}C NMR
210 200 190 180 170 160 150 140 130 120 110 100 90 80 70 60 50 40 30 20 10 ppm

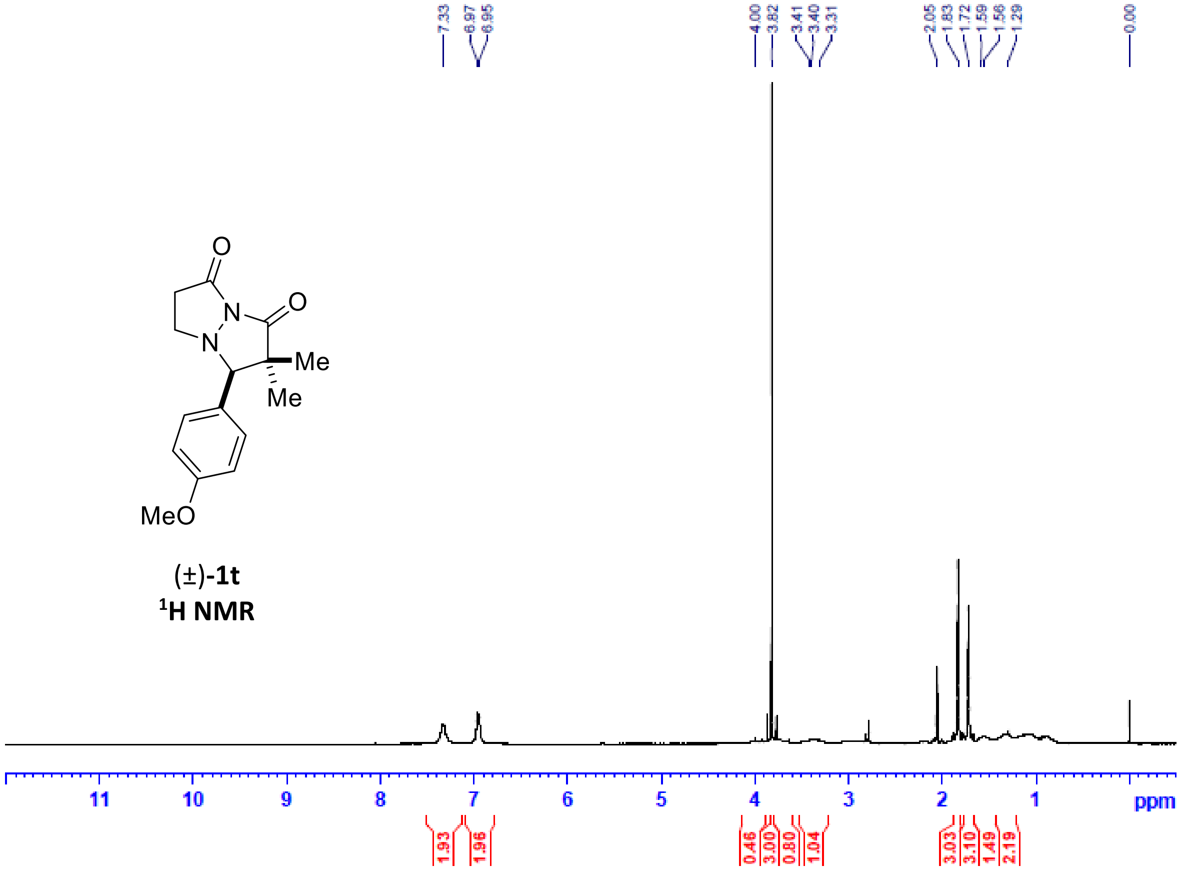

(±)-**1t**
^{1}H NMR

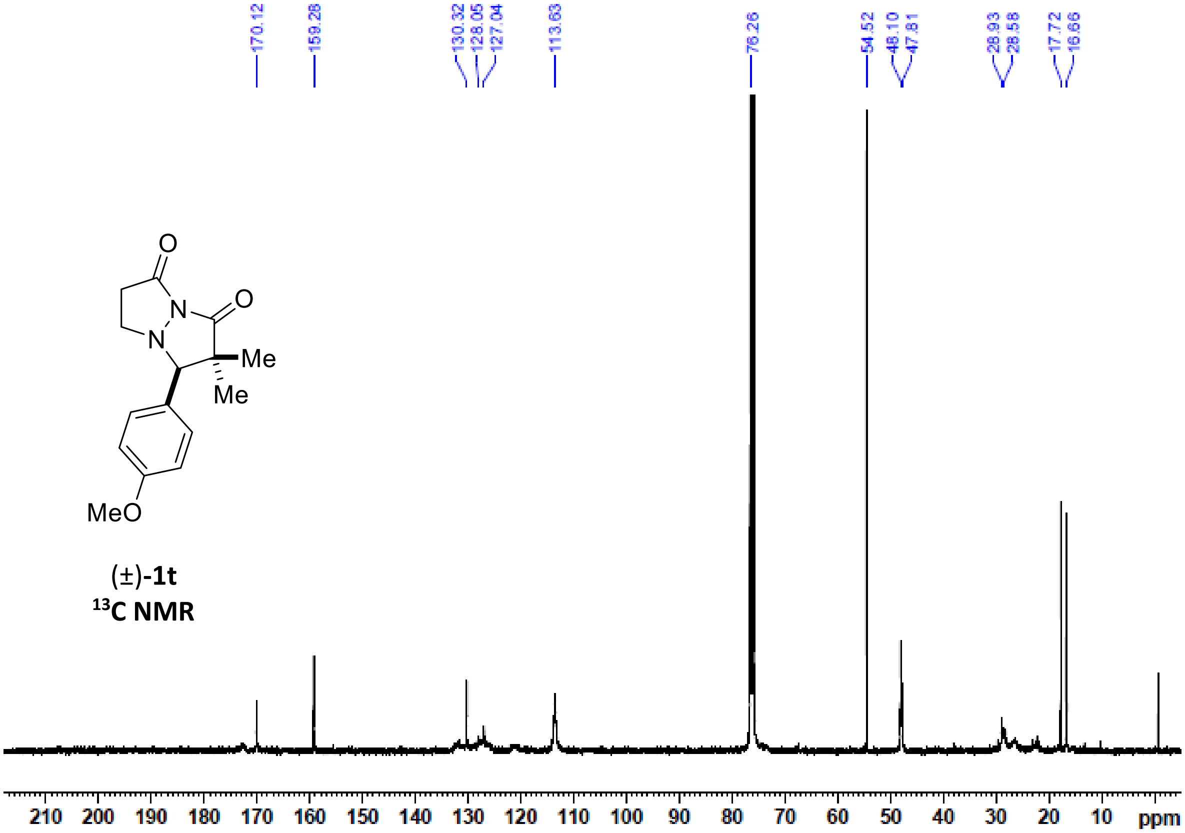

(±)-**1t**
^{13}C NMR

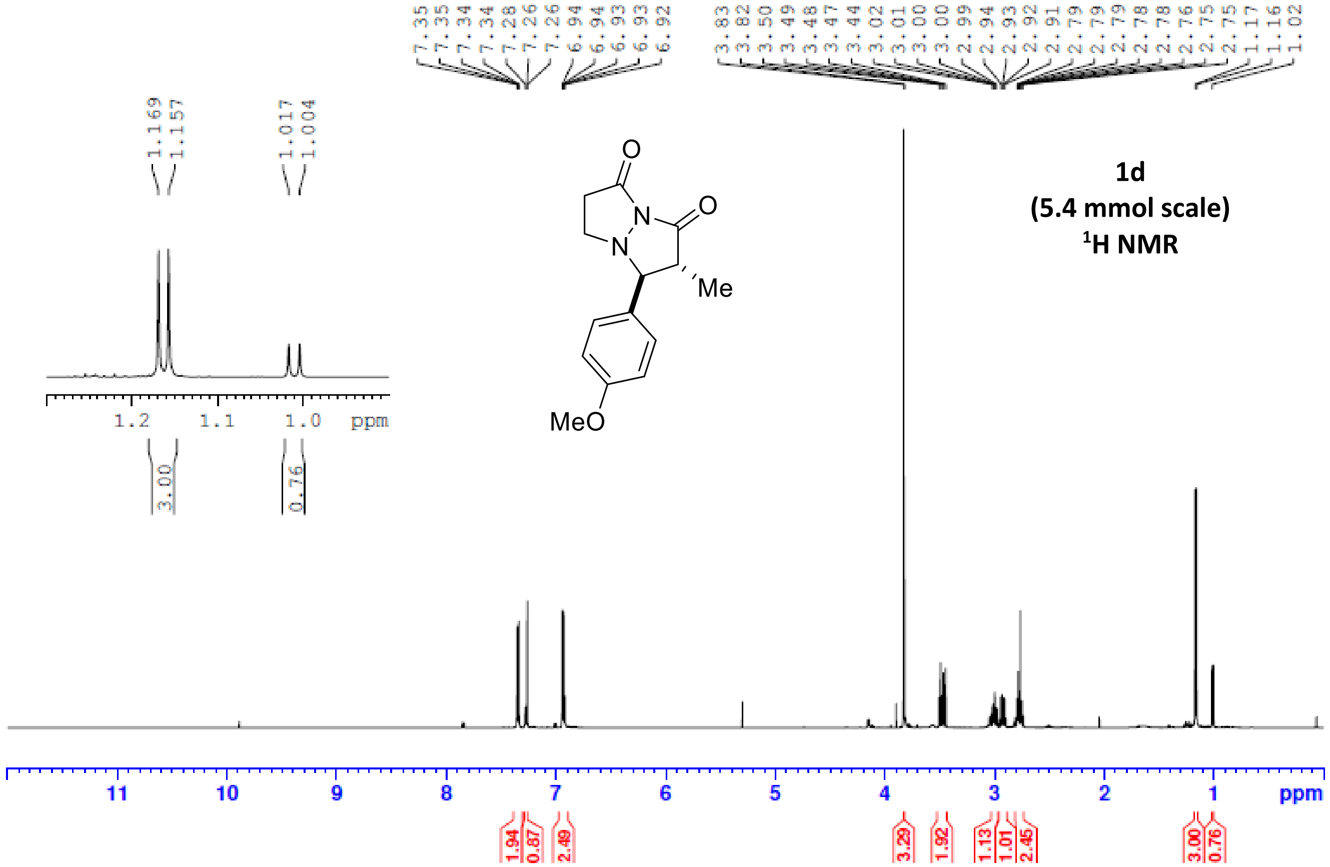
1d
(5.4 mmol scale)
^{1}H NMR
MeO
Me
ppm

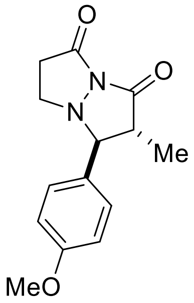

1d
(5.4 mmol scale)
^{13}C NMR

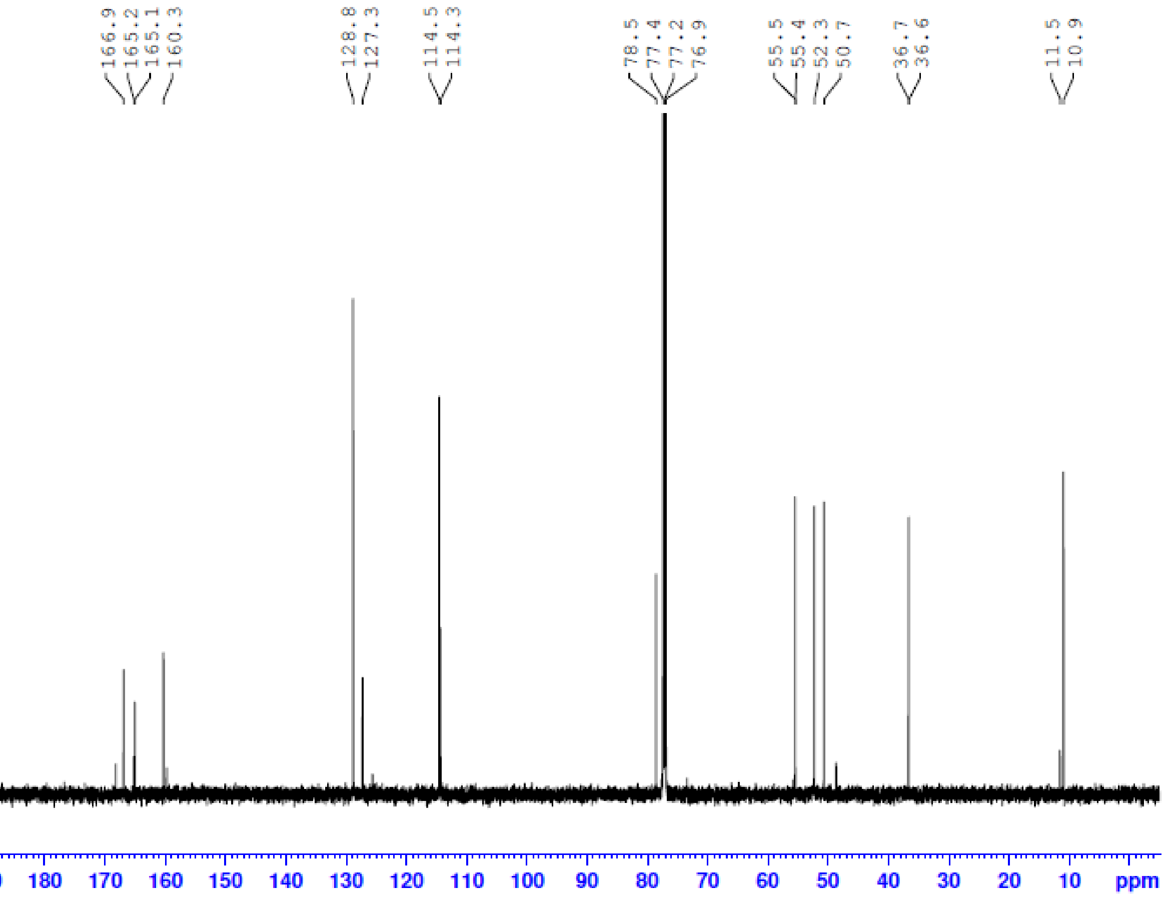

Experimental Section

General

THF was freshly distilled from benzophenone ketyl radical under nitrogen prior to use, while Hünig's base (diisopropylethylamine) was distilled from calcium hydride.[1] Most anhydrous solvents (dichloromethane and diethyl ether) were obtained by passing through activated alumina columns on a solvent purification system. Hydrazine monohydrate, methyl acrylate, benzaldehyde, *p*-anisaldehyde, *o*-tolualdehyde, *p*-tolualdehyde, 4-fluorobenzaldehyde, lithium perchlorate, copper(II) triflate, erbium(III) triflate, ytterbium(III) triflate, copper(I) iodide, quinine, quinidine and dinc dust (<10 μm), were purchased from Aldrich Chemical Co. $(DHQ)_2$PHAL and $(DHQD)_2$PHAL were purchased from AK Scientific, Inc. Propionyl chloride, butyryl chloride, valeroyl chloride, acetoxyacetyl chloride and octanoyl chloride were purchased from Aldrich Chemical Co. and distilled prior to use.[1] TLC plates (Sorbent Technologies, UV254, 250μM) were used as received. TMS-quinine, Me-quinidine and *epi*-quinine thiourea were synthesized according to literature procedure.[2] Azomethine imines **4a**-**4k** (Table 2 graphic, **4a**: R^2 = Ph; **4b**: R^2 = 4-MeC_6H_4; **4c**: R^2 = 2-MeC_6H_4; **4d**: R^2 = 4-$MeOC_6H_4$; **4e**: R^2 = 2-$MeOC_6H_4$; **4f**: R^2 = 2,4-di$MeOC_6H_4$; **4g**: R^2 = 4-FC_6H_4; **4h**: R^2 = 4-ClC_6H_4; **4i**: R^2 = 3-ClC_6H_4; **4j**: R^2 = *i*-Pr; **4k**: R^2 = *c*-Hex) were prepared according to literature procedures.[3] Disubstituted ketenes were prepared according to literature procedures.[4-6]

NMR spectra were recorded on a Bruker DPX Avance 200 spectrometer (200 MHz for ^{1}H and 50 MHz for ^{13}C) and on a Bruker Biospin AG 400 spectrometer (400 MHz for ^{1}H and 100 MHz for ^{13}C). NMR chemical shifts were reported relative to TMS (0 ppm) for ^{1}H and to $CDCl_3$ (77.23 ppm) for ^{13}C spectra. High resolution mass spectra were recorded on Agilent Technologies 6520 Accurate Mass Q-TOF LC-MS instrument at Oakland University. Low resolution mass spectra were recorded on a GC/MS Hewlett Packard HP 6890 GC instrument with a 5973 mass selective detector. IR spectra were recorded on a Bio Rad FTS-175C spectrometer. Optical rotations were measured on a Rudolph DigiPol 781 TDV automatic polarimeter. Chiral high performance liquid chromatography analysis (HPLC) was

performed using Daicel Chiralpak AD, Chiralpak AD-H, Chiralpak OD and Chiralpak OD-H (25 × 0.46 cm) (Daicel chemical Ind., Ltd.) on a Perkin Elmer Flexar instrument attached with diode array detector (deuterium lamp, 190-600 nm) with HPLC-grade isopropanol and hexanes as the eluting solvents. Enantiomeric excesses were determined at λ = 254 or 225 nm (details given for each compound).

Diastereomeric ratios for bicyclic pyrazolidinones **1** were determined by ^{1}H NMR analysis of the crude product in each case and corroborated by HPLC analysis in some cases. Products **1a**-**1d,** *ent*-**1c,** *ent*-**1d**, **1g**, *ent*-**1g**, **1k**-**1o**, *ent*-**1n**, *ent*-**1o**, **1q**, and *ent*-**1q** were prepared and characterized as previously reported.[7]

General procedure for alkaloid-catalyzed [3 + 2] cycloaddition of ketene and azomethine imine: To a stirring solution of azomethine imine **4a**-**4k** (0.30 mmol, 1 equiv) and catalyst (0.03 mmol, 0.1 equiv) in dichloromethane (2.0 mL) at –25 °C, Hünig's base (0.10 mL, 0.60 mmol, 2 equiv) was added. To this stirring reaction mixture, a solution of acid chloride (0.60 mmol, 2 equiv) in dichloromethane (1.0 mL) was added over a period of 10 h via syringe pump. The reaction was stirred at this temperature for another 6 h and then poured into cold water (15 mL), extracted with dichloromethane (20 mL × 3). The combined organic layers were washed with water (50 mL), and brine (50 mL), and dried over sodium sulfate. The solvent was removed under reduced pressure. The residue was then dissolved in dichloromethane and passed through a plug of regular silica gel (10 g, 2 × 2 cm) using 10% EtOAc/dichloromethane as elutant to afford crude product (free from catalyst) for diastereomeric ratio and enantiomeric excess measurement. Pure product was isolated after further regular silica gel column chromatographic purification using EtOAc/dichloromethane as eluent (mentioned details below).

(2*S*,3*R*)-2-Methyl-3-phenyltetrahydro-1*H*,7*H*-pyrazolo[1,2-a]pyrazole-1,7-dione (*ent*-1a): Following general procedure, propionyl chloride (0.053 mL, 0.58 mmol) in dichloromethane (1.0 mL) was added over 10 h to a solution of **4a** (50 mg, 0.29 mmol), Hünig's base (0.10 mL, 0.58 mmol) and $(DHQD)_2PHAL$ (22 mg, 0.03 mmol) in dichloromethane (1.9 mL) at –25 °C. Elution with 3% EtOAc/dichloromethane through silica gel column afforded *ent*-**1a** as a light yellowish solid (57 mg, 86%), dr = 1.9:1 (by ^{1}H NMR); HPLC analysis: 97% ee [Daicel Chiralcel AD-H column; 1.2 mL/min; solvent system: 2% isopropanol in hexane; retention times: 45.1 min (minor), 54.6 min (major)]; Mp: 136-140 ^oC; IR (thin film) 2984, 2921, 2885, 1770, 1701, 1455, 1319, 1294, 1275, 699 cm^{-1}; ^{1}H NMR (400 MHz, $CDCl_3$, TMS, Major isomer): δ 7.47-7.33 (m, 5H), 3.57-3.48 (m, 2H), 3.10-2.90 (m, 2H), 2.87-2.74 (m, 2H), 1.19 (d, *J* = 7.1 Hz, 3H); ^{13}C NMR (100 MHz, $CDCl_3$, Major isomer): δ 166.7, 165.0, 135.7, 129.2, 129.0, 127.7, 79.0, 52.4, 50.9, 36.6, 11.0; $(M + H)^+$ HRMS m/z calcd for $(C_{13}H_{15}N_2O_2)^+$: 231.1134; found: 231.1131.

(2*S*,3*R*)-2-Methyl-3-(p-tolyl)tetrahydro-1*H*,7*H*-pyrazolo[1,2-a]pyrazole-1,7-dione (*ent*-1b): Following general procedure, propionyl chloride (0.059 mL, 0.64 mmol) in dichloromethane (1.0 mL) was added over 10 h to a solution of **4b** (60 mg, 0.32 mmol), Hünig's base (0.11 mL, 0.64 mmol) and $(DHQD)_2PHAL$ (25 mg, 0.03 mmol) in dichloromethane (2.2 mL) at –25 °C. Elution with 2.5% EtOAc/dichloromethane through silica gel column afforded *ent*-**1b** as a white solid (71 mg, 91%), dr = 3:1 (by ^{1}H NMR and HPLC); HPLC analysis: 98% ee [Daicel

Chiralcel AD-H column; 1 mL/min; solvent system: 3% isopropanol in hexane; retention times: 35.1 min (minor), 40.1 min (major)]; Mp: 215-218 °C; IR (thin film) 2974, 2917, 2884, 1771, 1699, 1520, 1327, 1294, 1275, 831 cm^{-1}; ^1H NMR (400 MHz, $CDCl_3$, TMS, Major isomer): δ 7.35-7.29 (m, 2H), 7.26-7.18 (m, 2H), 3.54-3.46 (m, 2H), 3.08-2.88 (m, 2H), 2.85-2.73 (m, 2H), 2.37 (s, 3H), 1.18 (d, *J* = 7.0 Hz, 3H); ^{13}C NMR (100 MHz, $CDCl_3$, Major isomer): δ 166.9, 165.0, 139.2, 132.6, 129.9, 127.6, 78.8, 52.3, 50.8, 36.6, 21.3, 11.0; $(M + H)^+$ HRMS m/z calcd for $(C_{14}H_{17}N_2O_2)^+$: 245.1290; found: 245.1287.

(2*R*,3*S*)-3-(2-Methoxyphenyl)-2-methyltetrahydro-1H,7H-pyrazolo[1,2-a]pyrazole-1,7-dione (1e): Following general procedure, propionyl chloride (0.045 mL, 0.49 mmol) in dichloromethane (0.6 mL) was added over 10 h to a solution of **4e** (50 mg, 0.25 mmol), Hünig's base (0.09 mL, 0.49 mmol) and $(DHQ)_2PHAL$ (19 mg, 0.02 mmol) in dichloromethane (1.9 mL) at –25 °C. Elution with 0.75%-3% EtOAc/dichloromethane through silica gel column afforded **1e** as a light yellow sticky solid (59 mg, 93%), dr = 2.8:1 (by ^1H NMR); HPLC analysis (Major isomer): 99% ee [Daicel Chiralcel OD column; 0.5 mL/min; solvent system: 15% isopropanol in hexane; retention times: 37.3 min (major), 45.9 min (minor)]; IR (thin film) 2977, 2935, 2840, 1775, 1702, 1326, 1308, 1276, 1244, 1023, 755 cm^{-1}; ^1H NMR (400 MHz, $CDCl_3$, TMS, Major isomer): δ 7.61 (d, *J* = 7.5 Hz, 1H), 7.34 (t, *J* = 8.5 Hz, 1H), 7.03 (t, *J* = 7.5 Hz, 1H), 6.93 (d, *J* = 8.3 Hz, 1H), 4.20 (d, *J* = 12.3 Hz, 1H), 3.84 (s, 3H), 3.47 (t, *J* = 9.0 Hz, 1H), 3.06-2.92 (m, 2H), 2.86-2.71 (m, 2H), 1.19 (d, *J* = 7.1 Hz, 3H); ^{13}C NMR (100 MHz, $CDCl_3$, Major isomer): δ 167.3, 165.2, 158.1, 129.9, 127.9, 123.9, 121.4, 111.1, 71.0, 55.6, 52.5, 50.3, 36.6, 11.3; $(M + H)^+$ HRMS m/z calcd for $(C_{14}H_{17}N_2O_3)^+$: 261.1239; found: 261.1239.

(2*S*,3*R*)-3-(2-Methoxyphenyl)-2-methyltetrahydro-1H,7H-pyrazolo[1,2-a]pyrazole-1,7-dione (*ent*-1e): Following general procedure, propionyl chloride (0.045 mL, 0.49 mmol) in dichloromethane (0.6 mL) was added over 10 h to a solution of **4e** (50 mg, 0.25 mmol), Hünig's base (0.09 mL, 0.49 mmol) and $(DHQ)_2PHAL$ (19 mg, 0.02 mmol) in dichloromethane (1.9 mL) at –25 °C. Elution with 3% EtOAc/dichloromethane through silica gel column afforded *ent*-**1e** as a light yellow sticky solid (57 mg, 89%), dr = 1.2:1 (by ^1H NMR); HPLC analysis (Major isomer): 99% ee [Daicel Chiralcel OD column; 0.5 mL/min; solvent system: 15% isopropanol in hexane; retention times: 38.3 min (minor), 44.4 min (major)]; IR (thin film) 2977, 2934, 2840, 1775, 1702, 1326, 1308, 1278, 1244, 1023, 756 cm^{-1}; ^1H NMR (400 MHz, $CDCl_3$, TMS, Major isomer): δ 7.61 (d, *J* = 7.5 Hz, 1H), 7.34 (t, *J* = 8.4 Hz, 1H), 7.04 (t, *J* = 7.5 Hz, 1H), 6.93 (d, *J* = 8.3 Hz, 1H), 4.20 (d, *J* = 12.3 Hz, 1H), 3.84 (s, 3H), 3.47 (t, *J* = 8.7 Hz, 1H), 3.06-2.91 (m, 2H), 2.86-2.71 (m, 2H), 1.19 (d, *J* = 7.1 Hz, 3H); ^{13}C NMR (100 MHz, $CDCl_3$, Major isomer): δ 167.3, 165.2, 158.1, 129.9, 127.9, 124.0, 121.4, 111.1, 71.1, 55.7, 52.5, 50.4, 36.6, 11.4; $(M + H)^+$ HRMS m/z calcd for $(C_{14}H_{17}N_2O_3)^+$: 261.1239; found: 261.1238.

(2*R*,3*S*)-3-(2,4-Dimethoxyphenyl)-2-methyltetrahydro-1H,7H-pyrazolo[1,2-a]pyrazole-1,7-dione (1f): Following general procedure, propionyl chloride (0.039 mL, 0.43 mmol) in dichloromethane (0.6 mL) was added over 10 h to a solution of **4f** (50 mg, 0.21 mmol), Hünig's base (0.08 mL, 0.43 mmol) and $(DHQ)_2PHAL$ (17 mg, 0.02 mmol) in dichloromethane (1.5 mL) at –25 °C. Elution with 2.5% EtOAc/dichloromethane through silica gel column afforded **1f** as a light yellow sticky solid (28 mg, 45%), dr = 1.6:1 (by ^1H NMR); HPLC

analysis (Major isomer): 95% ee [Daicel Chiralcel AD-H column; 1 mL/min; solvent system: 7% isopropanol in hexane; retention times: 31.4 min (major), 32.9 min (minor)]; IR (thin film) 2939, 2841, 1776, 1699, 1612, 1507, 1322, 1292, 1227, 1031, 827 cm^{-1}; ^{1}H NMR (400 MHz, $CDCl_3$, TMS, Major isomer): δ 7.48 (d, *J* = 8.5 Hz, 1H), 6.59-6.44 (m, 2H), 4.09 (d, *J* = 12.3 Hz, 1H), 3.83 (s, 3H), 3.81 (s, 3H), 3.46 (t, *J* = 8.9 Hz, 1H), 3.08-2.90 (m, 2H), 2.85-2.70 (m, 2H), 1.17 (d, *J* = 7.0 Hz, 3H); ^{13}C NMR (100 MHz, $CDCl_3$, Major isomer): δ 167.5, 165.2, 161.3, 159.2, 128.8, 116.0, 105.4, 98.8, 71.1, 55.63, 55.60, 52.4, 50.0, 36.6, 11.2; $(M + H)^+$ HRMS m/z calcd for $(C_{15}H_{19}N_2O_4)^+$: 291.1345; found: 291.1341.

(2*S*,3*R*)-3-(2,4-Dimethoxyphenyl)-2-methyltetrahydro-1H,7H-pyrazolo[1,2-a]pyrazole-1,7-dione (*ent*-1f): Following general procedure, propionyl chloride (0.039 mL, 0.43 mmol) in dichloromethane (0.6 mL) was added over 10 h to a solution of **4f** (50 mg, 0.21 mmol), Hünig's base (0.08 mL, 0.43 mmol) and $(DHQD)_2PHAL$ (17 mg, 0.02 mmol) in dichloromethane (1.5 mL) at –25 °C. Elution with 2.5% EtOAc/dichloromethane through silica gel column afforded *ent*-**1f** as a light yellow sticky solid (25 mg, 40%), dr = 1:1.04 (in this entry the usual *trans*-isomer became minor isomer) (by ^{1}H NMR and HPLC); HPLC analysis (Minor isomer): 96% ee [Daicel Chiralcel AD-H column; 1 mL/min; solvent system: 7% isopropanol in hexane; retention times: 31.3 min (minor), 32.9 min (major)]; IR (thin film) 2938, 2842, 1776, 1699, 1612, 1507, 1321, 1292, 1227, 1031, 827 cm^{-1}; ^{1}H NMR (400 MHz, $CDCl_3$, TMS, Minor isomer): δ 7.48 (d, *J* = 8.5 Hz, 1H), 6.59-6.45 (m, 2H), 4.09 (d, *J* = 12.3 Hz, 1H), 3.83 (s, 3H), 3.81 (s, 3H), 3.46 (t, *J* = 8.9 Hz, 1H), 3.08-2.90 (m, 2H), 2.85-2.70 (m, 2H), 1.17 (d, *J* = 6.9 Hz, 3H); ^{13}C NMR (100 MHz, $CDCl_3$, Minor isomer): δ 167.5, 165.2, 161.3, 159.3, 128.8, 116.0, 105.4, 98.9, 71.1, 55.7, 55.6, 52.4, 50.0, 36.6, 11.2; $(M + H)^+$ HRMS m/z calcd for $(C_{15}H_{19}N_2O_4)^+$: 291.1345; found: 291.1342.

(2*R*,3*S*)-3-(4-Chlorophenyl)-2-methyltetrahydro-1*H*,7*H*-pyrazolo[1,2-a]pyrazole-1,7-dione (1h): Following general procedure, propionyl chloride (0.053 mL, 0.58 mmol) in dichloromethane (1.0 mL) was added over 10 h to a solution of **4h** (60 mg, 0.29 mmol), Hünig's base (0.10 mL, 0.58 mmol) and $(DHQ)_2PHAL$ (22 mg, 0.03 mmol) in dichloromethane (1.9 mL) at –25 °C. Elution with 2.5% EtOAc/dichloromethane through silica gel column afforded **1h** as a light yellow solid (70 mg, 92%), dr = 1.5:1 (by ^{1}H NMR and HPLC); HPLC analysis: 99% ee [Daicel Chiralcel OD column; 1.0 mL/min; solvent system: 12% isopropanol in hexane; retention times: 34.1 min (major), 51.2 min (minor)]; IR (thin film) 2977, 2923, 1770, 1698, 1294, 1087, 842 cm^{-1}; ^{1}H NMR (400 MHz, Acetone-d_6, TMS, Major isomer): δ 7.61-7.56 (m, 2H), 7.50-7.44 (m, 2H), 3.82 (d, *J* = 12.3 Hz, 1H), 3.49 (t, *J* = 5.2 Hz, 1H), 3.16-2.90 (m, 2H), 2.82-2.65 (m, 2H), 1.11 (d, *J* = 7.0 Hz, 3H); ^{13}C NMR (100 MHz, $CDCl_3$, Major isomer): δ 166.3, 164.8, 135.2, 134.3, 129.5, 129.0, 78.3, 52.4, 51.0, 36.6, 11.0; $(M + H)^+$ HRMS m/z calcd for $(C_{13}H_{14}ClN_2O_2)^+$: 265.0744; found: 265.0741.

(2*S*,3*R*)-3-(4-Chlorophenyl)-2-methyltetrahydro-1*H*,7*H*-pyrazolo[1,2-a]pyrazole-1,7-dione (*ent*-1h): Following general procedure, propionyl chloride (0.053 mL, 0.58 mmol) in dichloromethane (1.0 mL) was added over 10 h to a solution of **4h** (60 mg, 0.29 mmol), Hünig's base (0.10 mL, 0.58 mmol) and $(DHQ)_2PHAL$ (22 mg, 0.03 mmol) in dichloromethane (1.9 mL) at –25 °C. Elution with 2.5% EtOAc/dichloromethane through silica gel column afforded *ent*-**1h** as a light yellow solid (72 mg, 95%), dr = 2.6:1 (by ^{1}H NMR and HPLC); HPLC analysis: 96% ee [Daicel Chiralcel OD column; 1.0 mL/min; solvent system: 12%

isopropanol in hexane; retention times: 35.5 min (minor), 49.2 min (major)]; IR (thin film) 2979, 2923, 1770, 1701, 1290, 1089, 851 cm^{-1}; ^{1}H NMR (400 MHz, $CDCl_3$, TMS, Major isomer): δ 7.42-7.29 (m, 4H), 3.54-3.46 (m, 2H), 3.11-2.86 (m, 2H), 3.11-2.86 (m, 2H), 1.19 (d, *J* = 7.0 Hz, 3H); ^{13}C NMR (100 MHz, $CDCl_3$, Major isomer): δ 166.3, 164.8, 135.2, 134.3, 129.5, 129.0, 78.3, 52.4, 51.0, 36.6, 11.0; (M + H)$^+$ HRMS m/z calcd for $(C_{13}H_{14}ClN_2O_2)^+$: 265.0744; found: 265.0740.

(2*S*,3*R*)-3-(3-Chlorophenyl)-2-methyltetrahydro-1*H*,7*H*-pyrazolo[1,2-a]pyrazole-1,7-dione (1i): Following general procedure, propionyl chloride (0.058 mL, 0.63 mmol) in dichloromethane (1.0 mL) was added over 10 h to a solution of **4i** (65 mg, 0.31 mmol), Hünig's base (0.11 mL, 0.63 mmol) and $(DHQ)_2$PHAL (24 mg, 0.03 mmol) in dichloromethane (2.1 mL) at –25 °C. Elution with 2.5% EtOAc/dichloromethane through silica gel column afforded **1i** as a light yellow sticky solid (72 mg, 87%), dr = 2:1 (by ^{1}H NMR); HPLC analysis (Major isomer): 96% ee [Daicel Chiralcel OD-H column; 1 mL/min; solvent system: 20% isopropanol in hexane; retention times: 21.6 min (major), 34.6 min (minor)]; IR (thin film) 2980, 2936, 1772, 1698, 1370, 1328, 1172, 793 cm^{-1}; ^{1}H NMR (400 MHz, $CDCl_3$, TMS, Major isomer): δ 7.48 (bs, 1H), 7.41-7.23 (m, 3H), 3.57-3.48 (m, 2H), 3.13-2.88 (m, 2H), 2.87-2.74 (m, 2H), 1.21 (d, *J* = 7.0 Hz, 3H); ^{13}C NMR (100 MHz, $CDCl_3$, Major isomer): δ 166.2, 164.8, 138.0, 135.3, 130.5, 129.5, 127.7, 125.9, 78.2, 52.4, 50.9, 36.5, 11.0; (M + H)$^+$ HRMS m/z calcd for $(C_{13}H_{14}ClN_2O_2)^+$: 265.0744; found: 265.0741.

(2*R*,3*S*)-3-(3-Chlorophenyl)-2-methyltetrahydro-1*H*,7*H*-pyrazolo[1,2-a]pyrazole-1,7-dione (*ent*-1i): Following general procedure, propionyl chloride (0.058 mL, 0.63 mmol) in dichloromethane (1.0 mL) was added over 10 h to a solution of **4i** (65 mg, 0.31 mmol), Hünig's base (0.11 mL, 0.63 mmol) and $(DHQD)_2$PHAL (24 mg, 0.03 mmol) in dichloromethane (3.1 mL) at –25 °C. Elution with 2.5% EtOAc/dichloromethane through silica gel column afforded *ent*-**1i** as a light yellow sticky solid (76 mg, 92%), dr = 2.2:1 (by ^{1}H NMR); HPLC analysis (Major isomer): 97% ee [Daicel Chiralcel OD-H column; 1 mL/min; solvent system: 20% isopropanol in hexane; retention times: 23.0 min (minor), 31.8 min (major)]; IR (thin film) 2979, 2935, 1775, 1703, 1370, 1325, 1173, 789 cm^{-1}; ^{1}H NMR (400 MHz, $CDCl_3$, TMS, Major isomer): δ 7.48 (bs, 1H), 7.41-7.23 (m, 3H), 3.57-3.49 (m, 2H), 3.14-2.88 (m, 2H), 2.88-2.75 (m, 2H), 1.21 (d, *J* = 7.0 Hz, 3H); ^{13}C NMR (100 MHz, $CDCl_3$, Major isomer): δ 166.3, 164.9, 137.9, 135.2, 130.5, 129.5, 127.6, 125.9, 78.1, 52.4, 50.9, 36.5, 11.0; (M + H)$^+$ HRMS m/z calcd for $(C_{13}H_{14}ClN_2O_2)^+$: 265.0744; found: 265.0741.

(2*R*,3*R*)-3-Isopropyl-2-methyltetrahydro-1*H*,7*H*-pyrazolo[1,2-a]pyrazole-1,7-dione (1j): Following general procedure, propionyl chloride (0.066 mL, 0.71 mmol) in dichloromethane (1.0 mL) was added over 10 h to a solution of **4j** (50 mg, 0.36 mmol), Hünig's base (0.13 mL, 0.71 mmol) and $(DHQ)_2$PHAL (28 mg, 0.04 mmol) in dichloromethane (2.6 mL) at –25 °C. Elution with 2% EtOAc/dichloromethane through silica gel column afforded **1j** as a colorless oil (69 mg, 99%), dr = 3.7:1 (by ^{1}H NMR); HPLC analysis: 94% ee [Daicel Chiralcel AD-H column; 1.0 mL/min; solvent system: 10% isopropanol in hexane; retention times: 11.5 min (major), 15.7 min (minor)]; IR (thin film) 2968, 2941, 2878, 1777, 1700, 1325, 1306, 1174, 1095, 842 cm^{-1}; ^{1}H NMR (400 MHz, $CDCl_3$, TMS, Major isomer): δ 3.69-3.61 (m, 1H), 3.05-2.91 (m, 1H), 2.86-2.69 (m, 3H), 2.55 (dd, *J* = 12.3 & 3.9 Hz, 1H), 1.98-1.87 (m, 1H), 1.26 (d, *J* = 7.0 Hz, 3H), 1.07 (d, *J* = 7.0 Hz, 3H), 1.04 (d, *J* = 7.0 Hz, 3H); ^{13}C NMR (100 MHz, $CDCl_3$,

Major isomer): δ 167.9, 165.1, 79.8, 54.8, 44.5, 36.7, 28.8, 19.6, 18.5, 13.4; $(M + H)^+$ HRMS m/z calcd for $(C_{10}H_{17}N_2O_2)^+$: 197.1290; found: 197.1287.

(2*S*,3*S*)-3-Isopropyl-2-methyltetrahydro-1*H*,7*H*-pyrazolo[1,2-a]pyrazole-1,7-dione (*ent*-1j): Following general procedure, propionyl chloride (0.066 mL, 0.71 mmol) in dichloromethane (1.0 mL) was added over 10 h to a solution of **4j** (50 mg, 0.36 mmol), Hünig's base (0.13 mL, 0.71 mmol) and $(DHQD)_2PHAL$ (28 mg, 0.04 mmol) in dichloromethane (2.5 mL) at –25 °C. Elution with 2% EtOAc/dichloromethane through silica gel column afforded *ent*-**1j** as a colorless oil (69 mg, 99%), dr = 4.6:1 (by ^1H NMR); HPLC analysis: 95% ee [Daicel Chiralcel AD-H column; 1.0 mL/min; solvent system: 10% isopropanol in hexane; retention times: 11.4 min (minor), 15.6 min (major)]; IR (thin film) 2974, 2942, 2880, 1777, 1700, 1326, 1306, 1174, 1094, 842 cm^{-1}; ^1H NMR (400 MHz, $CDCl_3$, TMS, Major isomer): δ 3.69-3.61 (m, 1H), 3.05-2.91 (m, 1H), 2.86-2.69 (m, 3H), 2.55 (dd, J = 12.4 & 3.8 Hz, 1H), 1.97-1.88 (m, 1H), 1.26 (d, J = 7.0 Hz, 3H), 1.07 (d, J = 7.0 Hz, 3H), 1.04 (d, J = 7.0 Hz, 3H); ^{13}C NMR (100 MHz, $CDCl_3$, Major isomer): δ 167.9, 165.0, 79.7, 54.7, 44.4, 36.6, 28.6, 19.5, 18.4, 13.3; $(M + H)^+$ HRMS m/z calcd for $(C_{10}H_{17}N_2O_2)^+$: 197.1290; found: 197.1285.

(2*S*,3*R*)-2-Ethyl-3-(4-methoxyphenyl)tetrahydro-1*H*,7*H*-pyrazolo[1,2-a]pyrazole-1,7-dione (*ent*-1k): Following general procedure, butyryl chloride (0.063 mL, 0.59 mmol) in dichloromethane (1.0 mL) was added over 10 h to a solution of **4d** (60 mg, 0.29 mmol), Hünig's base (0.10 mL, 0.59 mmol) and $(DHQD)_2PHAL$ (23 mg, 0.029 mmol) in dichloromethane (2.0 mL) at –25 °C. Elution with 2% EtOAc/dichloromethane through silica gel column afforded *ent*-**1k** as a yellowish oil (54 mg, 67%), dr = 2.1:1 (by HPLC); HPLC analysis: 99% ee [Daicel Chiralcel AD-H column; 1.0 mL/min; solvent system: 3% isopropanol in hexane; retention times: 51.5 min (minor), 62.4 min (major)]; IR (thin film) 2965, 2935, 2876, 2838, 1775, 1701, 1514, 1282, 1246, 838 cm^{-1}; ^1H NMR (400 MHz, $CDCl_3$, TMS, Major isomer): δ 7.39-7.32 (m, 2H), 6.97-6.89 (m, 2H), 3.83 (s, 3H), 3.61 (d, J = 12.3 Hz, 1H), 3.45 (t, J = 8.5 Hz, 1H), 3.06-2.86 (m, 2H), 2.83-2.70 (m, 2H), 2.80-2.57 (m, 2H), 0.91 (t, J = 7.5 Hz, 3H); ^{13}C NMR (100 MHz, $CDCl_3$, Major isomer): δ 166.4, 164.9, 160.5, 129.1, 127.8, 114.6, 76.1, 56.3, 55.5, 52.2, 36.6, 19.6, 11.1; $(M + H)^+$ HRMS m/z calcd for $(C_{15}H_{19}N_2O_3)^+$: 275.1396; found: 275.1392.

(2*S*,3*R*)-3-(4-Methoxyphenyl)-2-propyltetrahydro-1*H*,7*H*-pyrazolo[1,2-a]pyrazole-1,7-dione (*ent*-1l): Following general procedure, valeroyl chloride (0.07 mL, 0.59 mmol) in dichloromethane (1.0 mL) was added over 10 h to a solution of **4d** (60 mg, 0.29 mmol), Hünig's base (0.10 mL, 0.59 mmol) and $(DHQD)_2PHAL$ (23 mg, 0.03 mmol) in dichloromethane (2.0 mL) at –25 °C. Elution with 2% EtOAc/dichloromethane through silica gel column afforded *ent*-**1l** as a yellowish oil (60 mg, 71%), dr = 2.9:1 (by HPLC); HPLC analysis: 99% ee [Daicel Chiralcel AD-H column; 0.5 mL/min; solvent system: 7% isopropanol in hexane; retention times: 49.1 min (minor), 64.6 min (major)]; IR (thin film) 2958, 2933, 2872, 2839, 1776, 1702, 1514, 1283, 1246, 836 cm^{-1}; ^1H NMR (400 MHz, $CDCl_3$, TMS, Major isomer): δ 7.38-7.32 (m, 2H), 6.97-6.90 (m, 2H), 3.83 (s, 3H), 3.57 (d, J = 12.3 Hz, 1H), 3.44 (t, J = 8.6 Hz, 1H), 3.04-2.91 (m, 2H), 2.81-2.71 (m, 2H), 1.80-1.66 (m, 1H), 1.56-1.44 (m, 1H), 1.44-1.29 (m, 1H), 1.29-1.14 (m, 1H), 0.80 (t, J = 7.3 Hz, 3H); ^{13}C NMR (100 MHz, $CDCl_3$, Major isomer): δ 166.6, 164.9, 160.4, 129.2, 127.7, 114.6, 76.9, 55.5, 54.8,

52.2, 36.6, 29.0, 20.0, 14.2; $(M + H)^+$ HRMS m/z calcd for $(C_{16}H_{21}N_2O_3)^+$: 289.1552; found: 289.1552.

(2*S*,3*R*)-2-Hexyl-3-(4-methoxyphenyl)tetrahydro-1*H*,7*H*-pyrazolo[1,2-a]pyrazole-1,7-dione (*ent*-1m): Following general procedure, octanoyl chloride (0.10 mL, 0.59 mmol) in dichloromethane (1.0 mL) was added over 10 h to a solution of **4d** (60 mg, 0.29 mmol), Hünig's base (0.10 mL, 0.59 mmol) and $(DHQD)_2$PHAL (23 mg, 0.03 mmol) in dichloromethane (2.0 mL) at –25 °C. Elution with 1.5% EtOAc/dichloromethane through silica gel column afforded *ent*-**1m** as a yellowish oil (78 mg, 80%), dr = 3.3:1 (by ^{1}H NMR and HPLC); HPLC analysis: >99% ee [Daicel Chiralcel AD-H column; 1.0 mL/min; solvent system: 5% isopropanol in hexane; retention times: 23.4 min (minor), 27.3 min (major)]; IR (thin film) 2954, 2926, 2856, 1777, 1703, 1513, 1304, 1282, 1247, 836 cm^{-1}; ^{1}H NMR (400 MHz, $CDCl_3$, TMS, Major isomer): δ 7.37-7.31 (m, 2H), 6.96-6.90 (m, 2H), 3.83 (s, 3H), 3.57 (d, *J* = 12.3 Hz, 1H), 3.43 (t, *J* = 8.4 Hz, 1H), 3.04-2.89 (m, 2H), 2.81-2.69 (m, 2H), 1.79-1.46 (m, 2H), 1.41-1.04 (m, 8H), 0.82 (t, *J* = 7.0 Hz, 3H); ^{13}C NMR (100 MHz, $CDCl_3$, Major isomer): δ 166.6, 164.9, 160.5, 129.2, 127.8, 114.6, 76.8, 55.6, 55.0, 52.2, 36.6, 31.6, 29.3, 26.7, 26.5, 22.7, 14.2; $(M + H)^+$ HRMS m/z calcd for $(C_{19}H_{27}N_2O_3)^+$: 331.2022; found: 331.2019.

(1*R*,2*R*)-3,5-Dioxo-1-(*p*-tolyl)tetrahydro-1*H*,5*H*-pyrazolo[1,2-a]pyrazol-2-yl acetate (1p): Following general procedure, acetoxyacetyl chloride (0.070 mL, 0.64 mmol) in dichloromethane (1.0 mL) was added over 10 h to a solution of **4c** (60 mg, 0.32 mmol), Hünig's base (0.11 mL, 0.64 mmol) and $(DHQ)_2$PHAL (25 mg, 0.03 mmol) in dichloromethane (2.2 mL) at –25 °C. Elution with 3% EtOAc/dichloromethane through silica gel column afforded **1p** as a yellowish oil (25 mg, 27%), dr = 5.4:1 (by ^{1}H NMR and HPLC); HPLC analysis: 99% ee [Daicel Chiralcel OD-H column; 1.0 mL/min; solvent system: 18% isopropanol in hexane; retention times: 31.8 min (minor), 34.0 min (major)]; IR (thin film) 2850, 1793, 1748, 1706, 1436, 1420, 1210, 1099, 763 cm^{-1}; ^{1}H NMR (400 MHz, $CDCl_3$, TMS, Major isomer): δ 7.71-7.65 (m, 1H), 7.32-7.24 (m, 2H), 7.22-7.16 (m, 1H), 5.74 (d, *J* = 11.2 Hz, 1H), 4.41 (d, *J* = 11.2 Hz, 1H), 3.58 (t, *J* = 8.5 Hz, 1H), 3.05-2.93 (m, 1H), 2.89-2.73 (m, 2H), 2.39 (s, 3H), 2.11 (s, 3H); ^{13}C NMR (100 MHz, $CDCl_3$, Major isomer): δ 168.7, 165.2, 160.5, 137.0, 131.7, 131.2, 129.2, 127.3, 127.2, 78.4, 71.8, 52.8, 35.5, 20.6, 19.7; $(M + H)^+$ HRMS m/z calcd for $(C_{15}H_{17}N_2O_4)^+$: 289.1188; found: 289.1185.

(1*S*,2*S*)-3,5-Dioxo-1-(*p*-tolyl)tetrahydro-1*H*,5*H*-pyrazolo[1,2-a]pyrazol-2-yl acetate (*ent*-1p): Following general procedure, acetoxyacetyl chloride (0.057 mL, 0.53 mmol) in dichloromethane (1.0 mL) was added over 10 h to a solution of **4c** (50 mg, 0.27 mmol), Hünig's base (0.093 mL, 0.53 mmol) and $(DHQD)_2$PHAL (21 mg, 0.03 mmol) in dichloromethane (1.7 mL) at –25 °C. Elution with 3% EtOAc/dichloromethane through silica gel column afforded *ent*-**1p** as a yellowish oil (20 mg, 26%), dr = 2.2:1 (by ^{1}H NMR); HPLC analysis: 93% ee [Daicel Chiralcel OD-H column; 1.0 mL/min; solvent system: 18% isopropanol in hexane; retention times: 29.5 min (major), 36.1 min (minor)]; IR (thin film) 2850, 1793, 1748, 1706, 1436, 1420, 1210, 1099, 763 cm^{-1}; ^{1}H NMR (400 MHz, $CDCl_3$, TMS, Major isomer): δ 7.71-7.65 (m, 1H), 7.32-7.24 (m, 2H), 7.22-7.17 (m, 1H), 5.74 (d, *J* = 11.2 Hz, 1H), 4.40 (d, *J* = 11.2 Hz, 1H), 3.59 (t, *J* = 8.6 Hz, 1H), 3.05-2.94 (m, 1H), 2.89-2.73 (m, 2H), 2.39 (s, 3H), 2.11 (s, 3H); ^{13}C NMR (100 MHz, $CDCl_3$, Major isomer): δ 168.8, 165.2,

160.5, 137.0, 131.7, 131.2, 129.2, 127.3, 127.2, 78.4, 71.8, 52.9, 35.5, 20.6, 19.7; $(M + H)^+$ HRMS m/z calcd for $(C_{15}H_{17}N_2O_4)^+$: 289.1188; found: 289.1187.

(*S*)-3-Phenyltetrahydro-1*H*,7*H*-pyrazolo[1,2-a]pyrazole-1,7-dione (1r): Following general procedure, acetyl chloride (0.056 mL, 0.72 mmol) in dichloromethane (1.0 mL) was added over 10 h to a solution of **4a** (50 mg, 0.29 mmol), Hünig's base (0.13 mL, 0.72 mmol), CuI (55 mg, 0.29 mmol) and TMSQ **6** (11 mg, 0.03 mmol) in dichloromethane (1.8 mL) at –25 °C. Elution with 0.75-3.5% EtOAc/dichloromethane through silica gel column afforded **1r** as a colorless gum (14 mg, 23%), HPLC analysis: 43% ee [Daicel Chiralcel OD column; 1.0 mL/min; solvent system: 20% isopropanol in hexane; retention times: 27.1 min (major), 35.4 min (minor)]; [*α*]D24 = -63.3 (c = 0.8, CH_2Cl_2); IR (thin film) 2987, 2868, 1767, 1698, 1379, 1298, 703 cm^{-1}; ^1H NMR (400 MHz, $CDCl_3$, TMS): δ 7.49-7.33 (m, 5H), 4.06 (dd, *J* = 11.5 & 8.2 Hz, 1H), 3.51 (t, *J* = 8.9 Hz, 1H), 3.10-2.95 (m, 3H), 2.92-2.75 (m, 2H); ^{13}C NMR (100 MHz, $CDCl_3$): δ 164.8, 163.9, 136.1, 129.3, 129.2, 127.3, 71.2, 52.4, 45.6, 36.9; $(M + H)^+$ HRMS m/z calcd for $(C_{12}H_{13}N_2O_2)^+$: 217.0977; found: 217.0975.

(*R*)-3-Phenyltetrahydro-1*H*,7*H*-pyrazolo[1,2-a]pyrazole-1,7-dione (*ent*-1r): Following general procedure, acetyl chloride (0.056 mL, 0.72 mmol) in dichloromethane (1.0 mL) was added over 10 h to a solution of **4a** (50 mg, 0.29 mmol), Hünig's base (0.13 mL, 0.72 mmol), CuI (55 mg, 0.29 mmol) and MeQd **7** (27 mg, 0.03 mmol) in dichloromethane (1.8 mL) at –25 °C. Elution with 0.75-3.5% EtOAc/dichloromethane through silica gel column afforded *ent*-**1r** as a colorless gum (39 mg, 64%), HPLC analysis: 32% ee [Daicel Chiralcel OD column; 1.0 mL/min; solvent system: 20% isopropanol in hexane; retention times: 35.2 min (major), 28.1 min (minor)]; [*α*]D24 = 25.6 (c = 2.5, CH_2Cl_2); IR (thin film) 2980, 2870, 1769, 1696, 1379, 1301, 700 cm^{-1}; ^1H NMR (400 MHz, $CDCl_3$, TMS): δ 7.50-7.32 (m, 5H), 4.08 (dd, *J* = 11.7 & 7.9 Hz, 1H), 3.51 (t, *J* = 8.4 Hz, 1H), 3.10-2.95 (m, 3H), 2.92-2.75 (m, 2H); ^{13}C NMR (100 MHz, $CDCl_3$): δ 164.9, 164.0, 136.1, 129.2, 129.15, 127.3, 71.1, 52.4, 45.5, 36.9; $(M + H)^+$ HRMS m/z calcd for $(C_{12}H_{13}N_2O_2)^+$: 217.0977; found: 217.0974.

(*S*)-3-(4-Fluorophenyl)tetrahydro-1*H*,7*H*-pyrazolo[1,2-a]pyrazole-1,7-dione (1s): Following general procedure, acetyl chloride (0.074 mL, 1.04 mmol) in dichloromethane (1.0 mL) was added over 10 h to a solution of **4g** (50 mg, 0.26 mmol), Hünig's base (0.18 mL, 1.04 mmol), CuI (50 mg, 0.26 mmol) and TMSQ **6** (10 mg, 0.03 mmol) in dichloromethane (1.6 mL) at –25 °C. Elution with 0.75-3.5% EtOAc/dichloromethane through silica gel column afforded **1s** as a colorless gum (32 mg, 53%), HPLC analysis: 25% ee [Daicel Chiralcel OD column; 1.0 mL/min; solvent system: 20% isopropanol in hexane; retention times: 24.8 min (major), 31.8 min (minor)]; [*α*]D24 = -35.6 (c = 3.1, CH_2Cl_2); IR (thin film) 2998, 2874, 1780, 1696, 1511, 1306, 1222, 1160, 832 cm^{-1}; ^1H NMR (400 MHz, $CDCl_3$, TMS): δ 7.43 (dd, *J* = 8.6 & 3.4 Hz, 2H), 7.10 (t, *J* = 8.6 Hz, 2H), 4.08 (dd, *J* = 12.5 & 5.4 Hz, 1H), 3.49 (t, *J* = 8.6 Hz, 1H), 3.1-2.94 (m, 3H), 2.93-2.76 (m, 2H); ^{13}C NMR (100 MHz, $CDCl_3$): δ 164.8, 163.8, 163.1 (d, *J* = 248 Hz, 1C), 131.9 (d, *J* = 3 Hz, 1C), 129.1 (d, *J* = 8 Hz, 1C), 116.2 (d, *J* = 22 Hz, 1C), 70.4, 52.3, 45.6, 36.8; $(M + H)^+$ HRMS m/z calcd for $(C_{12}H_{12}FN_2O_2)^+$: 235.0883; found: 235.0883.

(*R*)-3-(4-Fluorophenyl)tetrahydro-1*H*,7*H*-pyrazolo[1,2-a]pyrazole-1,7-dione (*ent*-1s): Following general procedure, acetyl chloride (0.074 mL, 1.04 mmol) in dichloromethane (1.0 mL) was added over 10 h to a solution of **4g** (50 mg, 0.26 mmol), Hünig's base (0.18 mL,

1.04 mmol), CuI (50 mg, 0.26 mmol) and MeQd **7** (9 mg, 0.03 mmol) in dichloromethane (1.6 mL) at –25 °C. Elution with 0.75-3.5% EtOAc/dichloromethane through silica gel column afforded *ent*-**1s** as a colorless gum (36 mg, 59%), HPLC analysis: 24% ee [Daicel Chiralcel OD column; 1.0 mL/min; solvent system: 20% isopropanol in hexane; retention times: 32.3 min (major), 25.7 min (minor)]; [*α*]D24 = 20.9 (c = 2.6, CH_2Cl_2); IR (thin film) 2998, 2872, 1777, 1699, 1509, 1305, 1221, 1159, 829 cm^{-1}; ^{1}H NMR (400 MHz, $CDCl_3$, TMS): δ 7.43 (dd, *J* = 8.7 & 3.4 Hz, 2H), 7.10 (t, *J* = 8.6 Hz, 2H), 4.07 (dd, *J* = 12.5 & 5.4 Hz, 1H), 3.49 (t, *J* = 8.7 Hz, 1H), 3.1-2.93 (m, 3H), 2.93-2.76 (m, 2H); ^{13}C NMR (100 MHz, $CDCl_3$): δ 164.8, 163.8, 163.2 (d, *J* = 248 Hz, 1C), 131.9 (d, *J* = 3 Hz, 1C), 129.1 (d, *J* = 8 Hz, 1C), 116.2 (d, *J* = 22 Hz, 1C), 70.4, 52.3, 45.6, 36.8; $(M + H)^+$ HRMS m/z calcd for $(C_{12}H_{12}FN_2O_2)^+$: 235.0883; found: 235.0882.

5-(4-Methoxyphenyl)-4-methyl-4-phenyl-1-(1-phenylethylidene)tetrahydro-1*H*,3*H*,9*H*-pyrazolo[1,2-c][1,3,4]oxadiazepine-3,9-dione (11a): To a stirring solution of **4d** (60 mg, 0.29 mmol) and $(DHQ)_2$PHAL (23 mg, 0.03 mmol) in dichloromethane (2.0 mL) at –25 °C, a solution of methylphenylketene (78 mg, 0.59 mmol) in dichloromethane (1.0 mL) was added over a period of 10 h via syringe pump. The reaction was stirred at this temperature for another 6 h and then poured into cold water (15 mL), and extracted with dichloromethane (20 mL × 3). The combined organic layers were washed with water, and brine, and dried over sodium sulfate. Removal of the solvent under reduced pressure followed by plug of regular silica gel column chromatographic purification using 0.5-2% EtOAc/dichloromethane afforded **11a** as a yellow sticky solid (102 mg, 74%); dr = 3.7:1 (by ^{1}H NMR); IR (thin film) 2992, 2932, 2837, 1747, 1710, 1511, 1250, 1117, 1027, 696 cm^{-1}; ^{1}H NMR (400 MHz, $CDCl_3$, TMS, Major isomer): δ 7.35-7.21 (m, 4H), 7.21-7.06 (m, 8H), 6.99-6.90 (m, 2H), 4.36 (s, 1H), 3.82 (s, 3H), 3.75-3.64 (m, 1H), 3.26 (t, *J* = 9.9 Hz, 1H), 2.02 (dd, *J* = 16.9 & 9.0 Hz, 1H), 1.68 (s, 3H), 1.67-1.56 (m, 1H), 1.20 (s, 3H); ^{13}C NMR (100 MHz, $CDCl_3$, Major isomer): δ 171.7, 171.0, 160.5, 140.2, 136.5, 130.6, 128.9, 128.6, 128.3, 128.0, 127.83, 127.78, 126.8, 126.4, 126.1, 114.9, 72.5, 57.6, 55.5, 47.9, 31.6, 30.1, 18.3; $(M + H)^+$ HRMS m/z calcd for $(C_{29}H_{29}N_2O_4)^+$: 469.2127; found: 469.2127.

4-Methyl-4-phenyl-1-(1-phenylethylidene)-5-(*p*-tolyl)tetrahydro-1*H*,3*H*,9*H*-pyrazolo[1,2-c][1,3,4]oxadiazepine-3,9-dione (11b): To a stirring solution of **4b** (60 mg, 0.32 mmol) and $(DHQ)_2$PHAL (25 mg, 0.03 mmol) in dichloromethane (2.2 mL) at –25 °C, a solution of methylphenylketene (84 mg, 0.64 mmol) in dichloromethane (1.0 mL) was added over a period of 10 h via syringe pump. The reaction was stirred at this temperature for another 6 h and then poured into cold water (15 mL), extracted with dichloromethane (20 mL × 3). The combined organic layers were washed with water, and brine, and dried over sodium sulfate. Removal of the solvent under reduced pressure followed by plug of regular silica gel column chromatographic purification using 6% EtOAc/dichloromethane afforded **11b** as a yellow sticky solid (102 mg, 71%); dr = 3.1:1 (by ^{1}H NMR). Recrystallization from pentane/CH_2Cl_2 provided crystals suitable for X-ray crystal structure analysis; IR (thin film) 3024, 2989, 2940, 1749, 1713, 1358, 1330, 1118, 696 cm^{-1}; ^{1}H NMR (400 MHz, $CDCl_3$, TMS, Major isomer): δ 7.32-7.08 (m, 14H), 4.37 (s, 1H), 3.70 (q, *J* = 11.9 Hz, 1H), 3.27 (dt, *J* = 10.9 & 2.2 Hz, 1H), 2.37 (s, 3H), 2.00 (dd, *J* = 16.6 & 8.5 Hz, 1H), 1.68 (s, 3H), 1.66-1.53 (m, 1H), 1.20 (s, 3H);

^{13}C NMR (100 MHz, $CDCl_3$, Major isomer): δ 171.6, 170.9, 140.3, 139.4, 136.6, 131.3, 130.7, 130.2, 128.9, 128.6, 128.0, 127.8 (2-carbons), 127.4, 126.8, 126.4, 72.9, 57.5, 48.0, 31.6, 30.1, 21.3, 18.3; $(M + H)^+$ HRMS m/z calcd for $(C_{29}H_{29}N_2O_3)^+$: 453.2178; found: 453.2176.

1-(Diphenylmethylene)-5-(4-methoxyphenyl)-4,4-diphenyltetrahydro-1*H*,3*H*,9*H*-pyrazolo[1,2-c][1,3,4]oxadiazepine-3,9-dione (11c): To a stirring solution of **4d** (50 mg, 0.25 mmol) and $(DHQ)_2$PHAL (19 mg, 0.02 mmol) in dichloromethane (1.5 mL) at –25 °C, a solution of diphenylketene (95 mg, 0.49 mmol) in dichloromethane (1.0 mL) was added over a period of 10 h via syringe pump. The reaction was stirred at this temperature for another 6 h and then poured into cold water (15 mL), extracted with dichloromethane (20 mL × 3). The combined organic layers were washed with water, and brine, and dried over sodium sulfate. Removal of the solvent under reduced pressure followed by plug of regular silica gel column chromatographic purification using 8% EtOAc/dichloromethane afforded **11c** as a yellow sticky solid (42 mg, 29%); HPLC analysis: 0% ee [Daicel Chiralcel AD-H column; 1.0 mL/min; solvent system: 10% isopropanol in hexane; retention times: 16.4 min (one isomer), 20.0 min (one isomer)]; IR (thin film) 2927, 2904, 1750, 1702, 1512, 1496, 1186, 1095, 732 cm^{-1}; ^1H NMR (400 MHz, $CDCl_3$, TMS): δ 7.39-7.14 (m, 20H), 6.98-6.87 (m, 4H), 4.45 (s, 1H), 3.83 (s, 3H), 3.40 (q, J = 9.5 Hz, 1H), 3.04 (dt, J = 11.9 & 2.3 Hz, 1H), 1.49-1.38 (m, 1H), 1.32-1.19 (m, 1H); ^{13}C NMR (100 MHz, $CDCl_3$): δ 170.4, 169.4, 160.4, 138.3, 138.2, 136.3, 131.7, 131.3, 130.8, 130.4, 129.9, 129.5, 128.90, 128.86, 128.5, 128.3, 127.9, 127.8, 127.7, 127.6, 127.0, 126.8, 126.0, 114.8, 73.2, 66.7, 55.5, 46.8, 29.7; $(M + H)^+$ HRMS m/z calcd for $(C_{39}H_{33}N_2O_4)^+$: 593.2440; found: 593.2438.

2,2-Dimethyl-3-phenyltetrahydro-1H,7H-pyrazolo[1,2-a]pyrazole-1,7-dione (1t): A solution of dimethylketene in THF was prepared from 2-bromo-2-methylpropionyl bromide (0.12 mL, 0.98 mmol) and Zn dust (170 mg, 2.60 mmol) at –78 °C. To a stirring solution of **4d** (50 mg, 0.25 mmol), CuI (47 mg, 0.25 mmol) and $(DHQD)_2$PHAL (19 mg, 0.02 mmol) in dichloromethane (2.5 mL) at –78 °C, a solution of dimethylketene (0.98 mmol) in THF (1.0 mL) was added in one portion. The reaction was stirred at this temperature for another 6 h and then poured into cold water (15 mL), extracted with dichloromethane (20 mL × 3). The combined organic layers were washed with water and brine, and dried over sodium sulfate. Removal of the solvent under reduced pressure followed by plug of regular silica gel column chromatographic purification using 1.5-6% EtOAc/dichloromethane afforded **1t** as a colorless solid (50 mg, 74%); Mp = 217-219 °C; HPLC analysis: 0% ee [Daicel Chiralcel OD-H column; 1.0 mL/min; solvent system: 10% isopropanol in hexane; retention times: 20.8 min (one isomer), 25.1 min (one isomer)]; IR (thin film) 2990, 2938, 2846, 1738, 1711, 1693, 1579, 1307, 1132, 814 cm^{-1}; ^1H NMR (400 MHz, Acetone-D_6): δ 7.43-7.20 (m, 2H), 6.96 (d, J = 7.7 Hz, 2H), 4.15-3.90 (m, 1H), 3.82 (s, 3H), 3.80-3.65 (m, 1H), 3.53-3.25 (m, 1H), 1.83 (s, 3H), 1.72 (s, 3H), 1.65-1.45 (m, 1H), 1.43-1.20 (m, 1H); ^{13}C NMR (100 MHz, $CDCl_3$): δ 170.1, 159.3, 130.3, 128.1, 127.0, 113.6, 76.3, 48.1, 47.8, 28.9, 28.6, 17.7, 16.7; $(M + H)^+$ HRMS m/z calcd for $(C_{15}H_{19}N_2O_3)^+$: 275.1390; found: 275.1391.

Isomerization experiments:

*Treatment of crude product (ent-**1e**) with silica:*

To a solution of *ent*-**1e** (100 mg, 0.38 mmol) in CH_2Cl_2 (10 mL) was added silica (2 g) and stirred for 1.5 h at 50-55 °C. Then the mixture was cooled to room temperature and filtered. The residue was washed with CH_2Cl_2 (20 mL × 3). The combined filtrate was evaporated to afford the crude product for dr measurement by ^{1}H NMR (dr 1.6:1).

*Treatment of crude product (ent-**1e**) with (DHQD)$_2$PHAL:*

To a solution of *ent*-**1e** (48 mg, 0.21 mmol) in CH_2Cl_2 (4 mL) was added (DHQD)$_2$PHAL (14 mg, 10 mol%). The reaction was stirred at -25 °C for 16 h. Then, at rt, the reaction mixture was passed through a plug of regular silica gel (3 g, 2 × 2 cm) using 10% EtOAc/CH_2Cl_2 as eluent to afford the crude product (free from catalyst) for dr measurement by ^{1}H NMR (dr 1.2:1).

*Treatment of crude product (ent-**1e**) with $ZnBr_2$:*

To a solution of *ent*-**1e** (43 mg, 0.17 mmol) in CH_2Cl_2 (4 mL) was added $ZnBr_2$ (74 mg, 2 equiv). The reaction was stirred at -25 °C for 16 h. Then, at rt, the reaction mixture was filtered and the filtrate was evaporated to afford the crude product for dr measurement by ^{1}H NMR (dr 1.1:1).

*Treatment of crude product (ent-**1e**) with KO^{t}Bu:*

To a solution of *ent*-**1e** (23 mg, 0.09 mmol) in THF (37 M) at 0 °C, was added KO^{t}Bu (1 M in hexane, 0.022 mL, 0.25 equiv) dropwise. The mixture was heated to 50 °C, and stirred for 30 min. The reaction was quenched with HCl (0.1 M, ~5 mL) added dropwise, and extracted into dichloromethane. The solvent was evaporated to afford the crude product for dr measurement by ^{1}H NMR (dr 3.4:1).

*Treatment of crude product (ent-**1e**) with DBU:*

To a solution of *ent*-**1e** (92 mg, 0.35 mmol) in CH_2Cl_2 (2 mL, 0.03 M) at 0 °C, was added DBU (0.026 mL, 0.5 equiv). The solution was stirred for 15 min and then was quenched with 1 M HCl/H_2O. The organic and aqueous layers were separated, and the aqueous layer was back-extracted twice with CH_2Cl_2. The combined organic layers were dried over Na_2SO_4 and concentrated under vacuum. The solvent was evaporated to afford the crude product for dr measurement by ^{1}H NMR (dr 2.4:1).

Procedure for scale up (5.4 mmol) synthesis of 1d: To a stirring solution of azomethine imine **4d** (1.10 gm, 5.39 mmol, 1.0 equiv) and (DHQ)$_2$PHAL (0.42 g, 0.54 mmol, 0.10 equiv) in dichloromethane (36.0 mL) at –25 °C, Hünig's base (1.88 mL, 10.8 mmol, 2.0 equiv) was added. To this stirring reaction mixture, a solution of propionyl chloride (0.94 mL, 10.8 mmol, 2.0 equiv) in dichloromethane (18.0 mL) was added over a period of 10 h via syringe pump. The reaction was stirred at this temperature for another 6 h and then poured into cold water (150 mL), and extracted with dichloromethane (60 mL × 3). The combined organic layers were washed with water (80 mL), and brine (80 mL), and dried over sodium sulfate. The solvent was removed under reduced pressure. The residue was then dissolved in dichloromethane and passed through a plug of regular silica gel using 10% EtOAc/dichloromethane as eluent to afford crude **1d** (1.39 gm, 99%, free from catalyst) for diastereomeric ratio measurement (dr 4:1 by ^{1}H NMR). Further purification using regular silica gel column chromatography and eluting with 2.5% EtOAc/dichloromethane provided **1d** as a

pale yellow solid (1.33 gm, 95%). Analytical data for **1d** agreed with that of previously prepared material on 0.29 mmol scale.[7]

Attempted Conversion of 11a to [3.3.0] pyrazolidinone 1u:

A solution of **11a** (72 mg, 0.15 mmol) in dichloromethane (8.0 mL) was stirred at 40 °C for 5 h, and then cooled to room temperature, poured into cold water (20 mL), extracted with dichloromethane (20 mL × 3). The combined organic layers were washed with water (20 mL), and brine (20 mL), and dried over sodium sulfate. The solvent was removed under reduced pressure. The crude residue was compared with the starting material **11a** by TLC and ^{1}H NMR analysis, and it was confirmed that the starting material remained intact.

Attempted Conversion of 11b to [3.3.0] pyrazolidinone 1v:

A solution of **11b** (60 mg, 0.13 mmol), $(DHQ)_2$PHAL (10 mg, 0.01 mmol) in dichloromethane (8.0 mL) was stirred at 40 °C for 5 h, and then cooled to room temperature, poured into cold water (20 mL), extracted with dichloromethane (20 mL × 3). The combined organic layers were washed with water (20 mL) and brine (20 mL), and then dried over sodium sulfate. The solvent was removed under reduced pressure. The crude residue was compared with the starting material **11b** by TLC and ^{1}H NMR analysis, and it was confirmed that the starting material remained intact.

Attempted Synthesis of [3.3.0] pyrazolidinone 1u:

To a stirring solution of **4d** (60 mg, 0.29 mmol) and $(DHQ)_2$PHAL (23 mg, 0.03 mmol) in dichloromethane (2.0 mL) at room temperature, a solution of methylphenylketene (50 mg, 0.38 mmol) in dichloromethane (1.0 mL) was added over a period of 10 h via syringe pump. The reaction was stirred at room temperature for another 6 h and then poured into cold water (15 mL) and extracted with dichloromethane (20 mL × 3). The combined organic layers were washed with water, and brine, and dried over sodium sulfate. The solvent was removed under reduced pressure to provide crude **11a**, with no pyrazolidinone **1u** being formed.

Determination of Absolute and Relative Stereochemistry:

For **1**: X-ray crystallographic analysis of *ent*-**1d** revealed the relative configuration to be *trans* (*anti*), while the absolute configuration, was determined to be the (2*S*,3*R*)-enantiomer.[7]

For **11**: Relative stereochemistry of **11b** was determined to be *trans* (*anti*) by X-ray crystallography. Deposition Number 2073137 contains the supplementary crystallographic data for this paper. These data are provided free of charge by the joint Cambridge Crystallographic Data Centre and Fachinformationszentrum Karlsruhe Access Structures service www.ccdc.cam.ac.uk/structures

References:

1) W. L. F. Armarego, D. D. Perrin, *Purification of Laboratory Chemicals*, 4th Ed. Butterworth Heinemann, **2002**.
2) (a) C. D. Papageorgiou, S. V. Ley, M. J. Gaunt, *Angew. Chem.* **2003**, *115*, 852-855; *Angew. Chem. Int. Ed.* **2003**, *42*, 828-831; (b) M. A. Calter, *J. Org. Chem.* **1996**, *61*, 8006; (c) B. Vakulya, S. Varga, A.

Csámpai, T. Soós, *Org. Lett.* **2005**, *7*, 1967-1969; (d) P. Hammar, T. Marcelli, H. Hiemstra, F. Himo, *Adv. Synth. Catal.* **2007**, 349, 2537.

3) (a) R. Shintani, G. C. Fu, *J. Am. Chem. Soc.* **2003**, *125*, 10778-10779; (b) A.; Suárez, C. W. Downey, G. C. Fu, *J. Am. Chem. Soc.* **2005**, *127*, 11244; (c) R. Shintani, T. Hayashi, *J. Am. Chem. Soc.* **2006**, *128*, 6330; (d) H.-W. Zhao, B. Li, H.-L. Pang, T. Tian, X.-Q. Chen, X.-Q. Song, W. Meng, Z. Yang, Y.-D. Zhao, Y.-Y. Liu, *Org. Lett*. **2016**, *18*, 848-851; (e) S.-M. Li, B. Yu, J. Liu, H.-L. Li, R. Na, *Synlett* **2016**; *27*, 282-286.
4) S. Chen, A. A. Ibrahim, N. J. Peraino, D. Nalla, M. Mondal, M. Van Raaphorst, N. J. Kerrigan, *J. Org. Chem.* **2016**, *81*, 7824-7837.
5) J. E. Wilson, G. C. Fu, *Angew. Chem.* **2004**, *116,* 6518-6520; *Angew. Chem. Int. Ed.* **2004**, *43*, 6358-6360.
6) (a) B. L. Hodous, G. C. Fu, *J. Am. Chem. Soc.* **2002**, *124*, 10006-10007; (b) S. L. Wiskur, G. C. Fu, *J. Am. Chem. Soc.* **2005**, *127*, 6176-6177; (c) A. A. Ibrahim, D. Nalla, M. Van Raaphorst, N. J. Kerrigan, *J. Am. Chem. Soc.* **2012**, *134*, 2942-2945; (d) M. Panda, M. Mondal, S. Chen, A. A. Ibrahim, D. J. Twardy, N. J. Kerrigan, *Eur. J. Org. Chem.* **2020**, 5752-5764.
7) M. Mondal, K. A. Wheeler, N. J. Kerrigan, *Org. Lett.* **2016**, *18*, 4108-4111.
